# Satellitome Analysis of *Adalia bipunctata* (Coleoptera): Revealing Centromeric Turnover and Potential Chromosome Rearrangements in a Comparative Interspecific Study

**DOI:** 10.3390/ijms25179214

**Published:** 2024-08-25

**Authors:** Pablo Mora, José M. Rico-Porras, Teresa Palomeque, Eugenia E. Montiel, Sebastián Pita, Diogo C. Cabral-de-Mello, Pedro Lorite

**Affiliations:** 1 Department of Experimental Biology, Genetics Area, University of Jaén, Paraje las Lagunillas s/n, 23071 Jaén, Spain; pmora@ujaen.es (P.M.); jmrico@ujaen.es (J.M.R.-P.); tpalome@ujaen.es (T.P.); 2 Department of Biology, Genetics, Faculty of Sciences, Autonomous University of Madrid, 28049 Madrid, Spain; eugenia.montiel@uam.es; 3 Biodiversity and Global Change Research Centre (CIBC-UAM), Autonomous University of Madrid, 28049 Madrid, Spain; 4 Section Evolutive Genetics, Faculty of Sciences, University of the Republic, Iguá 4225, Montevideo 11400, Uruguay; spita@fcien.edu.uy; 5 Department of General and Applied Biology, Institute of Biosciences/IB, UNESP—São Paulo State University, Rio Claro 13506-900, SP, Brazil; cabral.mello@unesp.br

**Keywords:** Coleoptera, Coccinellidae, *Adalia bipunctata*, *Adalia decempunctata*, ladybird beetle, RepeatExplorer2, satellite DNA, satellitome, fluorescence in situ hybridization (FISH), Chromosome In Silico Mapping (CHRISMAPP), karyotype evolution

## Abstract

Eukaryotic genomes exhibit a dynamic interplay between single-copy sequences and repetitive DNA elements, with satellite DNA (satDNA) representing a substantial portion, mainly situated at telomeric and centromeric chromosomal regions. We utilized Illumina next-generation sequencing data from *Adalia bipunctata* to investigate its satellitome. Cytogenetic mapping via fluorescence in situ hybridization was performed for the most abundant satDNA families. In silico localization of satDNAs was carried out using the CHRISMAPP (Chromosome In Silico Mapping) pipeline on the high-fidelity chromosome-level assembly already available for this species, enabling a meticulous characterization and localization of multiple satDNA families. Additionally, we analyzed the conservation of the satellitome at an interspecific scale. Specifically, we employed the CHRISMAPP pipeline to map the satDNAs of *A. bipunctata* onto the genome of *Adalia decempunctata*, which has also been sequenced and assembled at the chromosome level. This analysis, along with the creation of a synteny map between the two species, suggests a rapid turnover of centromeric satDNA between these species and the potential occurrence of chromosomal rearrangements, despite the considerable conservation of their satellitomes. Specific satDNA families in the sex chromosomes of both species suggest a role in sex chromosome differentiation. Our interspecific comparative study can provide a significant advance in the understanding of the repeat genome organization and evolution in beetles.

## 1. Introduction

Repetitive DNA elements are one of the main components in the eukaryotic genomes [1]. The total abundance of the repetitive portion in the genomes can vary from one species to another and is mainly composed by the transposable elements and satellite DNA (satDNA) or tandem repeats of a monomeric sequence [2]. Satellite DNA is commonly located in the telomeric and centromeric chromosomal regions [3,4] being an important component of the constitutive heterochromatin [5]. The pericentromeric satDNA is considered important in chromatin maintenance, sister chromatid cohesion, chromosome pairing, formation of the centromere or the kinetochores, chromosome segregation and in genome stability [6,7,8,9,10,11]. For example, in humans, the highly studied α-Sat is transcribed and plays a relevant role in many important centromeric functions through the cell cycle [12,13,14]. In fact, amplification of several satDNAs has also been observed in different types of human cancer, probably linked to the alterations in the cell cycle that occur in these pathologies [15,16].

The repetitive DNA fraction of a genome is called the repeatome [17], and the fraction corresponding to satDNA is defined as the satellitome [18]. The emergence of new bioinformatics tools for data from next-generation sequencing (NGS) has opened new ways to determine and characterize the repeatome and satellitome from a genome [19,20]. In this way, RepeatExplorer and TAREAN, which use a graph-based methodology to determine the repeatome [19,20,21], have been extensively used. The great advantage of these pipelines is the ability to characterize the repeatome and the satellitome from a genome without having a reference genome using not-assembled genome data and low coverage sequencing data. Using all of those approaches, the analysis of the repetitive content present in a genome can be performed more deeply than using the restriction endonuclease technique, which generally only allows the isolation of abundant satDNAs. These approaches contribute also to the performance of comparative and phylogenomic studies [22,23].

The Coccinellidae beetle family belongs to the Polyphaga (Coleoptera) suborder and comprises around 6000 species distributed across six subfamilies (Sticholotidinae, Coccidulinae, Scymninae, Chilocorinae, Coccinellinae, and Epilachininae) and 360 genera [24]. Almost 90% of all ladybird beetles are carnivorous, feeding on aphids and other small insects [25,26]. Consequently, they are of economic interest as biological control agents due to their predation on agricultural pests. The two-spotted ladybird beetle *Adalia bipunctata* (Coccinellidae) typically has two red forewings with two black spots, although this species sometimes exhibits polymorphism in the color pattern and can present black elytra with two red spots [27]. *A. bipunctata* is considered one of the most valuable biological control agents for many crops [28], and has been marketed in Europe since 1999 [29,30].

Despite the fact that ladybird beetles are one of the most numerous groups of animals, satDNA has been studied in only about 50 species from five families within the order Coleoptera, mostly in the families Tenebrionidae, Chrysomelidae and Coccinellidae. Almost all studies utilized restriction endonucleases [31]. Research on Coccinellidae has been conducted on species from three genera, *Henosepilachna*, *Epilachna*, and *Hippodamia* [32,33,34]. Two families of satDNA have been isolated in the species *Henosepilachna argus* using classical methodologies, such as the use of restriction enzymes [32] and the construction of C0t-1 DNA libraries [33]. Mora et al. [32] identified a satDNA family with a monomer length of 658 bp, present in the subtelomeric regions of all the chromosomes, except for the long arm of the X chromosome, which lacks a positive hybridization signal. This satDNA was also reported to be transcribed [32]. Later, another satDNA was isolated in this species [33], which is the main component of the pericentromeric heterochromatin in *H. argus* as well as in another related species, *Epilachna paenulata*. This satDNA is the shortest described to date in Coleoptera, composed of the repetition of a hexanucleotide (TTAAAA). The only complete satellitome that has been characterized in Coccinellidae was from the species *Hippodamia variegata* [34] using NGS data. This satellitome comprises at least 30 families of satDNA, with almost all of them (27 out of 30) having a repeat unit longer than 120 bp.

In the present study, the newly generated NGS data from *A. bipunctata* were used to describe its satellitome. Apart from the advance in the understanding of genome structure in beetles, we detected turnover of satDNAs between related species, mainly for the centromeric region, and traced the occurrence of chromosome rearrangements. Initially, we mapped the most abundant satDNAs using cytogenetic tools. Subsequently, we employed the CHRISMAPP pipeline [35] to map all the satDNA families using the chromosome-level assembly of *A. bipunctata* (GenBank assembly no. GCA_910592335.1) [36] as a reference. In addition, we analyzed the conservation of the satellitome at interspecific scale. The presence of *A. bipunctata* satDNAs has been determined in the genome of *Adalia decempunctata*, which has already been sequenced and assembled at the chromosomal level (GenBank assembly no. GCA_951802165.1) [37], using the CHRISMAPP pipeline. This analysis, coupled with the creation of a synteny map between the genomes of both species, suggests a rapid turnover of the centromeric satDNA and the possible occurrence of chromosomal rearrangements, despite the conservation in their satellitomes. This interspecific comparative study can provide a significant advance in the understanding of genome structure and evolution in beetles.

## 2. Results and Discussion

### 2.1. Adalia bipunctata Satellitome Description

Most of the bioinformatics tools currently used for the characterization and analysis of the satellitome require short sequences generated by NGS. For this reason, the genome has been sequenced using Illumina technology. Data obtained through Illumina sequencing indicate that the genome of *A. bipunctata* has an A+T richness of 62.66%. This A+T richness appears to be a common feature among insect genomes. According to a recent study compiling available high-quality genome sequences and other large-scale genome projects across 150 insect species, the A+T content varies from 54.46% to 74.26% [38]. This study includes 15 beetle species, with A+T content ranging from 61.89% to 69.15%. Notably, *A. bipunctata* shows an A+T content of 62.87%, closely resembling the 62.66% calculated from our Illumina data. Furthermore, this study includes data from another ladybird beetle, *Coccinella septempuctata*, with an A+T content of 63.45%, slightly higher than that of *A. bipunctata*, but very similar to another ladybird beetle species, *H. variegata*, which has an A+T genome content of 63.6% [34].

Upon detailed analysis of the RepeatExplorer2 output, 85 distinct satDNA families were identified (Table 1). However, the sequence containing the most common insect telomeric repeat (TTAGG) was not detected using the RepeatExplorer2 pipeline. This limitation is often observed with RepeatExplorer, which has difficulty clustering and detecting similarities among low-complexity sequences such as telomeric repeats [39]. Nevertheless, this telomeric repeat was included in the RepeatMasker analysis of the Illumina reads. This analysis revealed the presence of the telomeric repeat TTAGG (AbipSat24-5-TEL), which constitutes approximately 0.04% of the genome (Table 1). Therefore, the *A. bipunctata* satellitome has, at least, 86 satDNA families. Among Coleoptera species, the satellitomes were better characterized in six other species belonging to four families [34,35,40,41,42,43], revealing distinct numbers of satDNA families, from 11 in *Tenebrio molitor* [42] to 165 in *Chrysolina americana* [35]. In *Rhyzopertha dominica*, the TRDB clustering tool resulted in the formation of 315 clusters, representing potential satDNA families [41]. However, only the 10 most abundant clusters, which comprised 37% of the clustered arrays, were analyzed in detail.

There is considerable variation in the monomer length among the satDNA families of *A. bipunctata*, ranging from 5 bp (telomeric repetition) to 2435 bp (Table 1, Supplementary Figure S1). The size distribution pattern of the repeat unit is similar to that found in other insects, where the most common monomer length is less than 500 bp [31]. Among the 86 satDNA families identified in *A. bipunctata*, six have monomers exceeding 1 kb, with the longest being AbipSat38-2435. Similar satDNAs, and even larger ones, have been found in the satellite DNA of other coleopterans, such as the 1169 bp *Pst*I satDNA from *Misolpampus goudoti* [3], the HvarSat07-2000 family of *H. variegata* [34], or the families CameSat051-3051 and CameSat010-3664 of *C. americana* [35].

According to RepeatMasker results, only two satDNA families in *A. bipunctata* exhibit abundances exceeding 1%: the main satDNA family, AbipSat01-187, accounting for a total of 9.98%, and AbipSat02-497, which constitutes 1.68% of the genome. Altogether, the different satDNA families comprise approximately 14.67% of the genome. The satellitome has been only analyzed in another Coccinellidae beetle, *H. variegata*, where satDNA constitutes 14.93% of its genome [34]. Remarkably, despite the significant disparity in genome sizes, 475 Mb for *A. bipunctata* [36] and 284 Mb for *H. variegata* [44], the proportion of satDNA in these two species remains very similar. Studies on the proportion of the satellitome in the genome from other Coleoptera families show variable results. Similar percentages have been found in species of other families such as the leaf beetle, *C. americana* (Chrysomelidae), in which the set of satDNA families represents 17.97% of its genome [35], although its genome is much larger (980 Mb) (NCBI accession number GCA_958502065.1) than in the analyzed Coccinellidae species. In the red palm weevil *Rhynchophorus ferrugineus* (Curculionidae), the satellitome represents 24.91% of its 779 Mb genome length [40,45]. Conversely, in *R. dominica* (Bostrichidae), the main 10 satDNA families altogether represent only 0.5% of its 479 Mb genome assembly [41]. Finally, the results for Tenebrionidae species are contradictory. Recently, Gržan et al. [43] estimated that the *Tribolium castaneum* satellitome represents 23.8% of its genomic sequence (204 Mb). However, previous studies indicated that the major satDNA alone comprises 35% of the genome [46]. Likewise, it has been determined that the satellitome of *T. molitor* represents 28% of the genome and only 0.79% of its 756.8 Mb genome assembly [42]. On the other hand, previous analysis determined that the main satDNA alone represents over 50% of the genome in this species [47]. According to the authors, these discrepancies could be due to both the imprecision of the older evaluations and the saturation of the new pipelines with highly amplified satDNAs. Nevertheless, despite the limited number of satellitomes currently characterized in Coleoptera, there is no clear correlation between genome size and the percentage of satDNA in the genome.

The assembled genome of *A. bipunctata* is currently available at the chromosome level [36]. The masking of satDNA sequences on these assemblies revealed a significantly lower amount of satDNA (8.13%) compared to the estimate obtained directly from Illumina reads (14.67%). These differences are not unexpected, as it has been previously observed that satDNA is underrepresented in the genome assemblies. The repetitive nature of satDNA sequences causes them to collapse in assemblies, leading to the underestimation or even the exclusion from the assembly altogether [48,49]. There are several examples of satDNA underestimation in insect genome assemblies. For example, in the beetle *T. castaneum*, it was estimated that the satDNA families TCAST1 and TCAST2 represent 35% of the genome, but they only accounted for 0.3% of the assembled genome [50,51]. Similar observations were made in the hemipteran *Rhodnius prolixus*, where satellitome represents 5.6% of the assembled genome but 8% in estimations using unassembled reads [52]. In the red palm weevil beetle (*R. ferrugineus*), the percentage of satDNA estimated using Illumina reads was 24.91% of the genome compared to 11.44% in the assembled chromosomes [40]. This study analyzes the correlation between the underestimation of a satDNA family and the degree of tandem structure, revealing that underestimation tends to be more pronounced when satDNA sequences are organized in large arrays compared to those dispersed in small arrays. In this way, a large cluster of satDNA is likely to be collapsed just in a few monomers, since satDNA families composed by small clusters scattered in the genome appear to be better assembled. In *A. bipunctata*, a significant discrepancy is noted specifically for the most abundant satDNA family, AbipSat01-187. This family constitutes only 3.29% of the genome in the assembled chromosomes, whereas it was estimated to represent 9.98% of the genome based on unassembled Illumina reads.

The divergence, estimated using the Kimura 2-parameter (K2P) genetic distance, ranges from 0.57% (AbipSat84-169) to 23.48% (AbipSat27-84) across all satDNA families, with an average divergence of 7.6%. In the assembled genome, the values ranged from 0.72% (AbipSat84-169) to 28.16% (AbipSat29-179), with an average of 9.02%. This wide range of variation has also been observed in the analysis of the satellitomes of other beetle species, such as *C. americana* (from 1.77% to 26.18%) [35], *H. variegata* (0.23% to 25.74%) [34] or *R. ferrugineus* (0.48% to 25.36%) [40]. Similar patterns of divergence have been reported in other insects using comparable methodologies [18,53,54,55]. Divergence values are inversely related to the homogenization process and the amplification of each satDNA but directly related to the mutation rate [56,57]. The low divergence values observed in *A. bipunctata* genome may reflect a homogenization process that can be seen in the satellitome landscape, where the abundance versus the divergence (against the consensus) of each satDNA family was plotted (Supplementary Figure S2). The mean peak of the distribution shows that most of the sequences have a value of 5%. This pattern is generally consistent across individual landscapes of satDNA families, although some families, such as AbipSat17-86 or AbipSat28-165, exhibit exceptions to this trend. Interestingly, certain satDNA families display a double peak pattern, which likely corresponds to both recent and older expansions of these sequences (e.g., AbipSat09-473). This phenomenon is not uncommon and has been observed in other insect genomes, including *H. variegata* [34].

The possible evolutionary relationship among all the satDNA families has also been studied. We found that five different satDNA families can be grouped into two different superfamilies (Supplementary Figure S3). In this context, a superfamily refers to a group of satDNA families exhibiting significant sequence similarity, suggesting the existence of a common ancestry from which they have diverged through the accumulation of mutations over time. The first superfamily (SF-1) includes three different families, AbipSat15-179, AbipSat25-176 and AbipSat29-174. Among them, the families AbipSat15-179 and AbipSat25-176 showed the highest identity percentage (75%), whereas the families AbipSat25-176 and AbipSat29-174 showed the lowest homology percentage (68.42%). Regarding the SF-2, it comprises two different families (AbipSat52-177 and AbipSat61-175), with a lower identity percentage between both sequences (67.43%). The existence of satDNA superfamilies has been described in other coleopteran species [40], in other insect groups such as Orthoptera [16,54,58], as well as in distant zoological groups such as fishes [59]. As the satellitome of a larger number of species is analyzed, it will be possible to determine whether the existence of superfamilies is a common feature in eukaryotes.

### 2.2. SatDNA Location in Adalia bipunctata Chromosomes by FISH and CHRISMAPP

**Figure 1:**
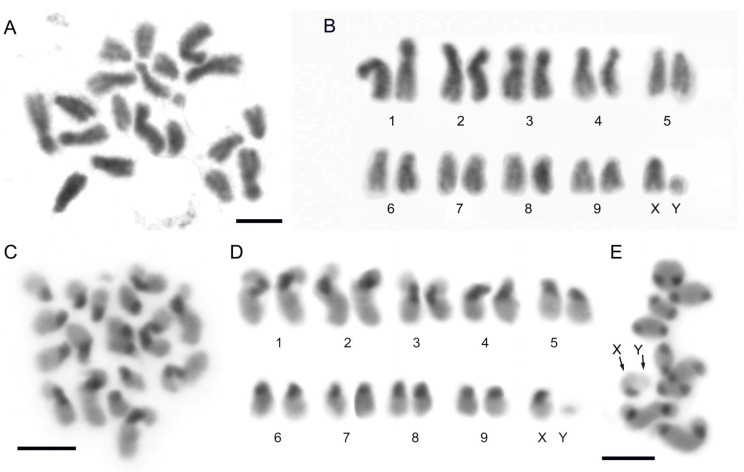
(**A**) Male mitotic metaphase and (**B**) karyotype of *A. bipunctata*. The karyotype consists of nine pairs of autosomes and the sex pair, an X chromosome and a dot-shaped Y chromosome. (**C**) Male mitotic metaphase and (**D**) karyotype after C-banding technique. Heterochromatin blocks are observed in the pericentromeric regions of all chromosomes except for the Y chromosome. (**E**) Meiotic metaphase after C-banding technique and subsequent DAPI staining (inverted image), with nine autosomal bivalents and the sex chromosomes in an Xy_p_ “parachute” shape. Arrows indicate the sex chromosomes. Bar = 5 μm.

The karyotype of *A. bipunctata* was first analyzed by Stevens [60] and consists of nine pairs of autosomes and the sexual pair XX/XY (Figure 1A,B). The three largest autosome pairs are submetacentric, with the remaining autosomes and the X chromosome being acrocentric (Figure 1B). The Y chromosome is notably small, and its morphology cannot be clearly determined. C-banding revealed the presence of large heterochromatic blocks in the pericentromeric regions of all autosomes and the X chromosome, whereas the Y chromosome seems to be euchromatic (Figure 1C,D), as was previously observed [61]. During meiosis, the sex chromosomes form the Xy_p_ “parachute” association (Figure 1E) [62]. In meiosis, the heterochromatic regions are distinctly visible due to their strong staining with DAPI (Figure 1E).

**Figure 2:**
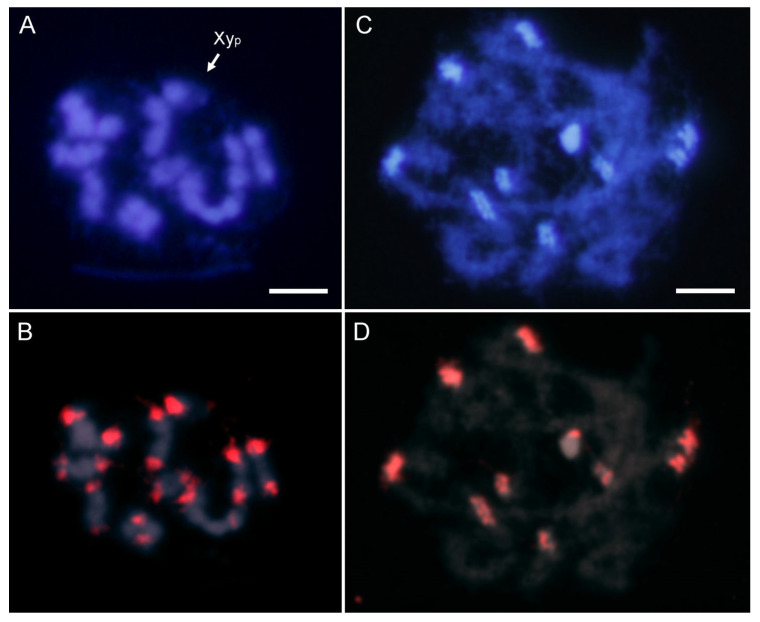
DAPI staining (**A**) and FISH (**B**) on male meiotic metaphase I of *A. bipunctata* using a specific probe for the AbipSat01-187 satDNA, revealing positive hybridization signals (in red) on all autosomes and the two sex chromosomes. DAPI staining (**C**) and FISH (**D**) on male meiotic prophase showing the coincidence of the hybridization signals and the location of the DAPI-positive heterochromatic blocks. Bar = 5 μm.

The most abundant satDNA families were located by FISH. Hybridization with an AbipSat01-187 probe showed strong positive signals on all the chromosomes including the sex chromosomes (Figure 2). These hybridization signals co-localized with the pericentromeric DAPI positive heterochromatin. Interestingly, although the Y chromosome is entirely euchromatic, it also displayed a weak hybridization signal (Figure 2B).

**Figure 3:**
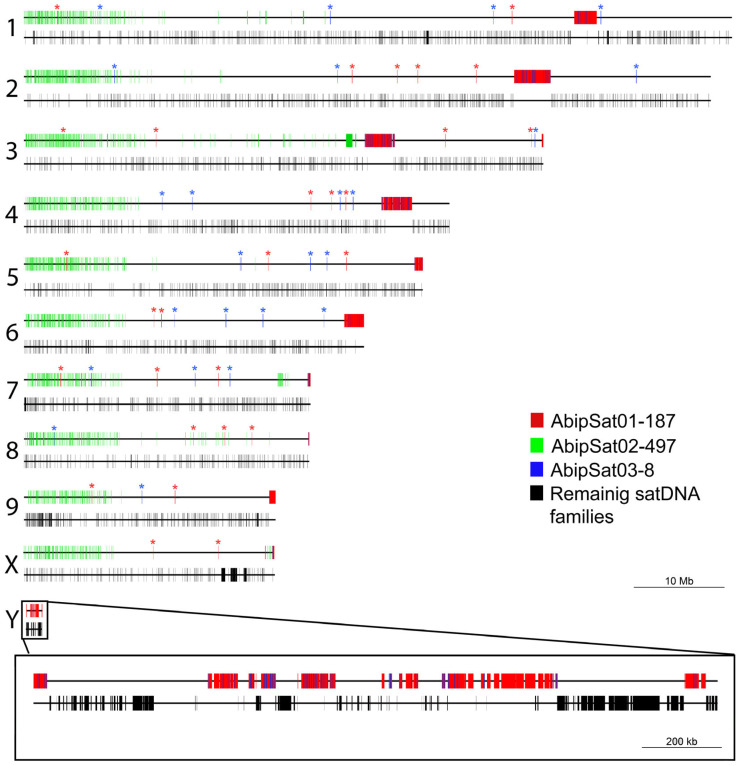
*A. bipunctata* pseudochromosomes showing the distribution of different satDNA families obtained through the CHRISMAPP approach. For each chromosome, two schemes are displayed. The top one illustrates the distribution of the three most abundant satDNA families, while the bottom one shows the distribution of the remaining satDNA families. Asterisks indicate the presence of short arrays of the AbipSat01-187 and AbipSat03-8 out of the heterochromatic blocks.

As above commented, a chromosome-level genome assembly of *A. bipunctata* is available [36]. Recently, a user-friendly pipeline (CHRISMAPP) was developed to map satDNAs in chromosome-level assembled genomes [35]. This pipeline has been applied to the *A. bipunctata* chromosomes (Figure 3, Supplementary Figure S4). CHRISMAPP analysis shows that the most abundant satDNA in the genome, AbipSat01-187, appears accumulated in large blocks. According to the FISH results, these regions correspond to pericentromeric heterochromatin blocks of the autosomes and the X chromosome. This satDNA is also present on the Y chromosome, although the small size of this chromosome does not allow determining if any of the AbipSat01-187 blocks on this chromosome correspond to the centromere position. The size of these regions, especially for chromosomes 7, 8, and X, is very small when compared to the signals obtained by FISH using this satellite as a probe. This point will be further discussed later. In addition to these regions, there are short arrays of this satDNA along the chromosome, in regions that are euchromatic, according to the results obtained by C-banding.

**Figure 4:**
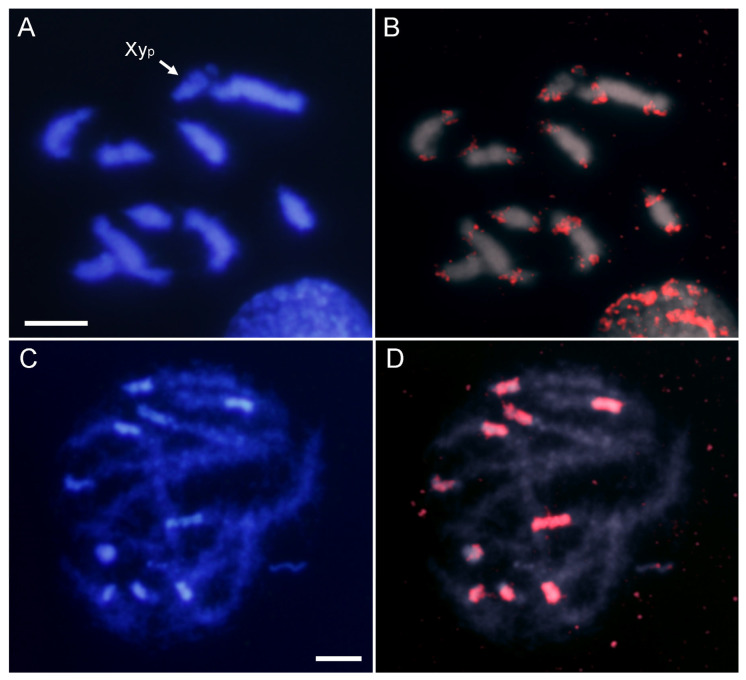
DAPI staining (**A**) and FISH (**B**) on male meiotic metaphase I of *A. bipunctata* using a probe specific to the AbipSat03-8 satDNA, revealing positive hybridization signals (in red) on all chromosomes and the two sex chromosomes. DAPI staining (**C**) and FISH (**D**) on male meiotic prophase showing the coincidence of the hybridization signals and the location of the DAPI positive heterochromatic blocks. Bar = 5 μm.

**Figure 5:**
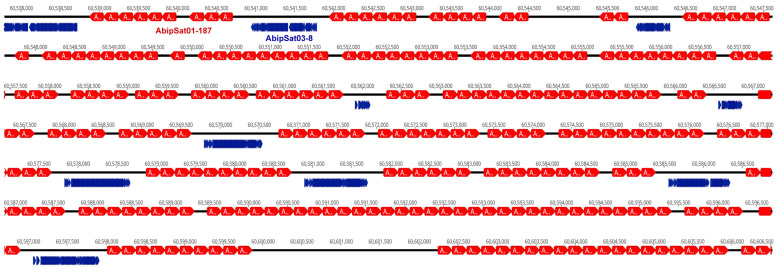
A region of the *A. bipunctata* chromosome 1 heterochromatin block showing alternative arrays of AbipSat01-187 (red) and AbipSat03-8 (blue).

The CHRISMAPP results also show that there is another satDNA family located in pericentromeric regions, AbipSat03-8. Similarly to what was observed with the AbipSat01-187 family, there are also short arrays of AbipSat03-8 satDNA in euchromatic regions. FISH using a probe for AbipSat03-8 reveals that the localization of this satDNA coincides with the heterochromatic regions of autosomes and the X chromosome, as well as the Y chromosome (Figure 4). Sequence analysis of the chromosomes shows that the pericentromeric regions are mainly composed of alternating arrays of both satDNAs in the two different orientations (Figure 5). 

**Figure 6:**
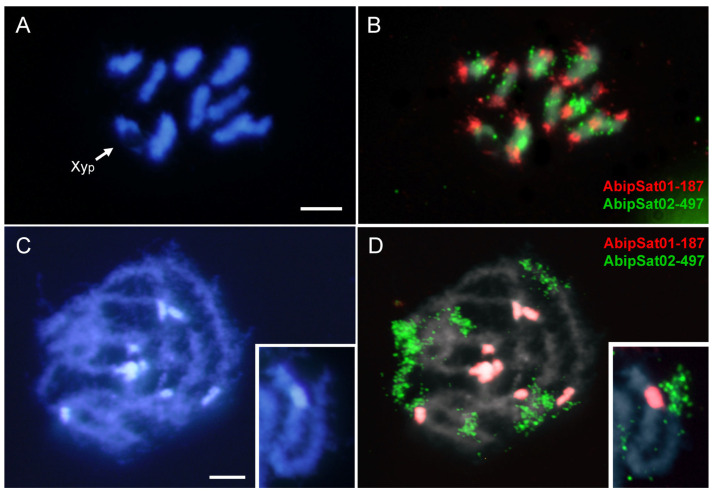
Double FISH using the AbipSat01-187 satDNA (labeled in red) and the satDNA AbipSat02-497 (labeled in green) as probes on *A. bipunctata* meiotic chromosomes. DAPI staining (**A**,**C**) and double FISH (**B**,**D**). On male meiotic metaphase I (**B**), positive hybridization signals with AbipSat01-187 are visible on the extremes of the bivalents (pericentromeric regions), the X and the Y chromosome. Hybridization signals with AbipSat02-497 are visible in the middle of the bivalents. On male meiotic prophase (**D**), hybridization signals with AbipSat01-187 are coincident with the DAPI positive heterochromatin, and the hybridization signals with AbipSat02-497 are located at one of the ends of the bivalents. Insert in C and D depicts an isolated bivalent showing the distribution of both satDNAs. Bar = 5 μm.

The second most abundant satDNA family in the genome, AbipSat02-497 (1.68%), shows a completely different distribution pattern compared to AbipSat01-187 and AbipSat03-8. This satDNA is located in the euchromatic regions, mainly accumulated at the terminal regions of the long arms of the autosomes and the X chromosome, and is not present on the Y chromosome. Double hybridization using this satDNA and AbipSat01-187 (to locate the pericentromeric regions) confirmed the locations predicted with the CHRISMAPP pipeline (Figure 6).

**Figure 7:**
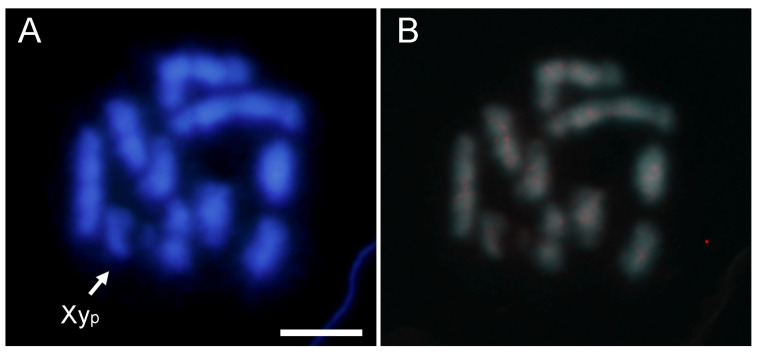
DAPI staining (**A**) and FISH (**B**) on male meiotic metaphase I of *A. bipunctata* using a probe for AbipSat04-193 (labeled in red) with two amplification rounds, revealing a diffuse labeling along the chromosomes. Bar = 5 μm.

Due to the resolution limitations of the FISH technique, for those satDNA families that were not highly abundant, FISH experiments were performed with amplification for the fourth and fifth most abundant satDNAs (AbipSat04-193 and AbipSat05-258). However, even with amplification, clear hybridization signals were not observed with either of the two probes, and only a diffuse labeling was visible along all chromosomes. Figure 7 shows the results obtained using a probe for AbipSat04-193. Based on these results and because the remaining satDNAs constitute a lower percentage of the genome, they have only been localized by CHRISMAPP (Supplementary Figure S4). In general, CHRISMAPP shows that except for the families AbipSat01-187 and AbipSat03-8, which are mainly located in pericentromeric regions, the remaining satDNA families are organized into short arrays distributed along the chromosomes.

### 2.3. Satellitome Representation in the Adalia bipunctata Genome Assembly

As previously indicated, estimation of satDNA using the sequence of the assembled chromosomes revealed that satDNA was underrepresented in the assembled chromosomes in comparison with Illumina reads. Genome assembly typically extends up to the limits of heterochromatic blocks. Within these regions, achieving adequate assembly becomes challenging due to the similarity between monomer units, resulting in collapse within this area. CHRISMAPP analysis indicated that pericentromeric regions of certain chromosomes, particularly chromosomes 7, 8, and X, contain small blocks of AbipSat01-187, the primary component of heterochromatin. However, these findings are not consistent with the results obtained from FISH or C-banding techniques, both of which demonstrated that the amount of heterochromatin in these chromosomes is similar to that of the remaining chromosomes.

The genome assembly of *A. bipunctata* encompasses a total of approximately 475 Mb, with 94.87% of the assembly allocated to 11 chromosomal-level scaffolds. These scaffolds represent the nine autosomes, as well as the X and Y sex chromosomes. In addition to these chromosomal scaffolds, there are 103 unplaced scaffolds within the genome assembly [36]. The CHRISMAPP pipeline was also applied to these scaffolds, confirming that they are mainly constituted by satDNA, predominantly by the two satDNA sequences that make up the heterochromatin in *A. bipunctata*, AbipSat01-187 and AbipSat03-8 (Supplementary Figure S5). These DNA segments are most likely part of pericentromeric heterochromatic regions. This observation helps to explain several discrepancies: first, the differences in heterochromatin quantity observed between the assembled chromosomes and the results of FISH and C-banding; second, the disparities between satDNA estimates derived from Illumina raw reads (14.67% of the genome) versus those from assembled chromosomes (8.13%). The abundance of all satDNAs has been recalculated using both assembled chromosomes and unplaced scaffolds, resulting in a value (12.20%) closer to the estimate obtained with raw reads.

**Figure 8:**
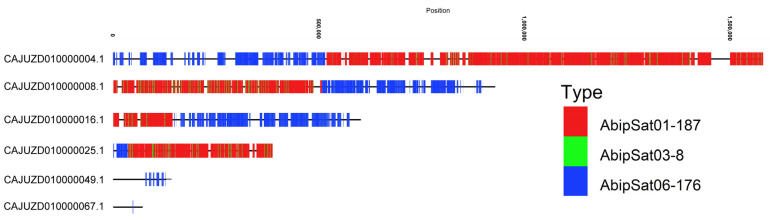
CHRISMAPP analysis on unplaced scaffolds of the *A. bipunctata* genome, showing the presence of six scaffolds with satDNA AbipSat06-176. This satDNA is associated with satDNAs AbipSat01-178 and AbipSat03-8 in four of the unplaced scaffolds.

**Figure 9:**
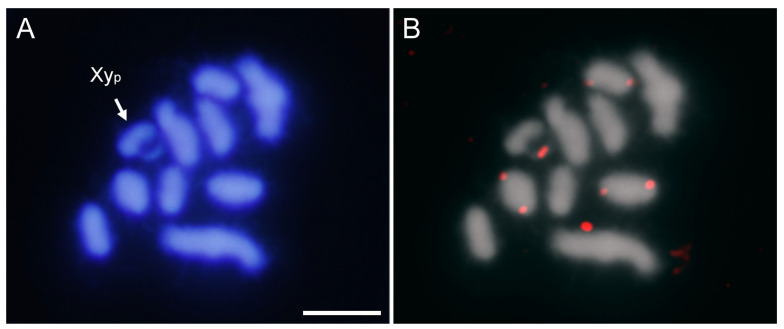
DAPI staining (**A**) and FISH (**B**) on male meiotic metaphase I of *A. bipunctata* using AbipSat06-176 as a probe. Hybridization signals (in red) are visible on three autosomal bivalents and the Y chromosome. Bar = 5 μm.

Among the most abundant satDNAs, significant differences were also observed for AbipSat06-176, which represents 0.23% of the genome (estimated from raw reads) but only 0.002% in the assembled chromosomes. When considering also unplaced scaffolds, the percentage of this satDNA increases to 0.20% of the genome (Table 1), a value close to the estimate obtained from Illumina reads. Therefore, this satDNA has been virtually eliminated in the chromosome assembly. Analysis of unplaced scaffolds revealed that the AbipSat06-176 satDNA is present in six scaffolds (Figure 8). In four of these scaffolds, AbipSat06-176 repeats are accumulated, whereas in two of them, they are dispersed. Interestingly, these arrays of AbipSat06-176 are found alongside arrays of AbipSat01-187 and AbipSat03-8, which are primarily accumulated in pericentromeric regions. This co-localization suggests that AbipSat06-176 may also be localized in these chromosomal regions. To confirm this, FISH was performed using a probe for AbipSat06-176. The results show that this satDNA is localized in three pairs of autosomes and in the Y chromosome (Figure 9), suggesting that each of these scaffolds could belong to one of these four chromosomal locations.

All data indicate that out of the 86 characterized satDNA families in the *A. bipunctata* genome, one of them is particularly amplified, constituting the pericentromeric heterochromatin. This satDNA, AbipSat01-178, is the most abundant in the genome (9.98%). Two other satDNA families are also preferentially amplified in these chromosomal regions but with significantly lower abundance: AbipSat03-8 (0.39%) and AbipSat06-176 (0.23%).

Until recently, the most common technique to isolate satDNA was based on the digestion of genomic DNA with restriction endonucleases. However, the use of restriction enzymes is generally not effective when the satDNA has a low copy number in the genome or when there is no restriction enzyme with a target present in the repetitive sequence [63]. Another used technique was the generation of *C0t*-1 libraries [32,64], but this also has a limitation in finding satDNAs with low copy numbers. Through these methodologies, the characterization of one or a few satDNA families per species has been possible. In general, these isolated satDNA families were the most abundant in the genome of the species and were mainly located in the pericentromeric constitutive heterochromatin [31]. Nowadays, the study of satDNA is more commonly conducted using data generated by NGS and the application of bioinformatics programs designed to find repeated sequences without the need to assemble the reads [65,66]. This methodology has enabled the characterization of numerous satDNA families in eukaryotic genome, collectively referred to as the “satellitome”. The amount of satDNA can vary greatly, even among closely related species [67], and, in some insects, the amount of satDNA can reach 50% of the genome, as in the beetle *T. molitor* [68] or the hemipteran *Triatoma delpontei* [69].

Although the number of satellitomes analyzed in insects so far is not very high, it is possible to observe that satDNAs can have three different distribution patterns. The most abundant satDNAs are part of the heterochromatin of all or most chromosomes. Other satDNAs may accumulate in specific regions of one or several chromosomes. The less abundant satDNAs generally seem to be organized into short arrays scattered throughout the genome.

### 2.4. Comparative Analysis with Adalia decempunctata

The genus *Adalia* is represented in southern Europe by two species: *A. bipunctata* and *A. decempunctata* [70]. The genome of *A. decempunctata* has also been sequenced and assembled at the chromosome level [37]. To conduct a comparative study of the satellitome between both species, the presence of the satDNAs of *A. bipunctata* in *A. decempunctata* has been determined. Given that our results in *A. bipunctata* show that satDNA is underrepresented in the assembled genome, this analysis was carried out using *A. decempunctata* raw reads available in GenBank. The analysis shows that out of the 86 satDNA families of *A. bipunctata*, 70 of them are also present in the genome of *A. decempunctata* (Supplementary Table S1). An interesting result is that the most abundant satDNA and main component of heterochromatin in *A. bipunctata* (AbipSat01-187) does not appear in the genome of *A. decempunctata*. This result is in accordance with the “library hypothesis”, which indicates that related species share a common library of satDNA sequences inherited from a common ancestor, and that subsequently certain variants are amplified and even eliminated, generating different collections of satDNAs in related species, while new families of satDNA can also be generated [71,72,73].

**Figure 10:**
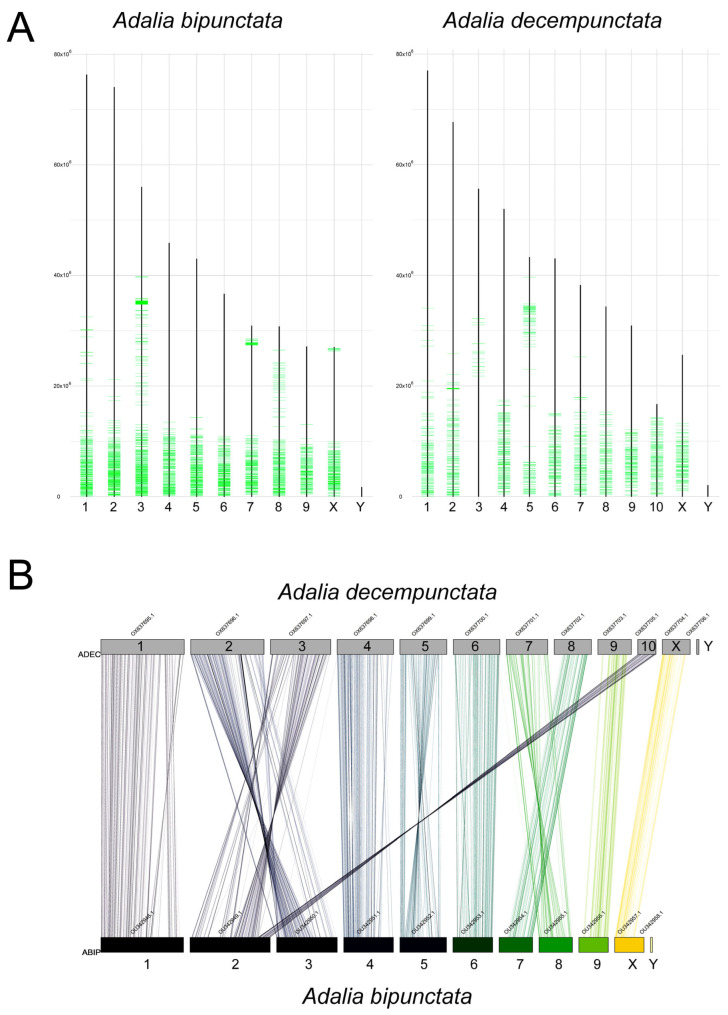
(**A**) Location of the satDNA AbipSat02-497 in the *A. bipunctata* and in the *A. decempunctata* genome assembly using CHRISMAPP. (**B**) Synteny map of the *A. bipunctata* and *A. decempunctata* genomes.

Using the CHRISMAPP pipeline, we located the satDNAs of *A. bipunctata* in *A. decempunctata* (Supplementary Figure S6). All these satDNAs show a dispersed distribution pattern in all chromosomes. In *A. decempunctata*, none of these satDNAs are organized in large blocks similar to those found in *A. bipunctata*. The satDNA AbipSat03-8 in *A. bipunctata* is mainly accumulated in the heterochromatic regions, although it is also dispersed in the euchromatin. This satDNA also appears in the genome of *A. decempunctata*, but in much smaller proportions (0.3864% of the genome in *A. bipunctata* and less than 0.0001% in *A. decempunctata*) and dispersed throughout the genome. Due to the lack of cytogenetic information on *A. decempunctata* [74], it is unclear whether this species has heterochromatic pericentromeric blocks similar to those found in *A. bipunctata*. If it does, it seems that the satDNAs of this heterochromatin are not present in *A. bipunctata*. This suggests a rapid turnover of centromeric satDNAs between these related species, despite the level of conservation of the satellitome between them. Rapid turnover of major satDNAs has been described in other insect groups, such as in the *Drosophila obscura* group species [75]. This process has been associated with the presence of chromosomal rearrangements involving centromeric regions. However, this does not seem to be the case in *Adalia*. In the family Coccinellidae, most species have a chromosome number of 2n = 20 [74]. It is assumed that 2n = 20 is the ancestral karyotypic condition in Coleoptera, consisting of nine pairs of autosomes and the X and Y chromosomes. *A. bipunctata* exhibits this ancestral karyotype. Although cytogenetic data for *A. decempunctata* are currently unavailable, the recent genome assembly suggests that this species has 10 pairs of autosomes plus the X and Y chromosomes. Karyotypes with 2n = 20 and 2n = 22 can be easily related through a single chromosomal fusion or fission event. However, we consider the fission hypothesis to be more plausible since *A. bipunctata* has a karyotype of 2n = 20, which is considered ancestral in Coleoptera. We analyzed the distribution of satDNA families between both species to see if it could help to explain the evolutionary relationship between their karyotypes. The most interesting data were provided by the distribution pattern of the AbipSat02-497 family (Figure 10A). As previously mentioned, in *A. bipunctata* this satDNA accumulates mainly in the terminal regions of the long arms of all autosomes and the X chromosome. In the chromosomes of *A. decempunctata*, one of the largest chromosomes (chromosome 3) lacks this accumulation of AbipSat02-497. Additionally, *A. decempunctata* presents a small chromosome (chromosome 10) with a huge amount of this satDNA. It is plausible to hypothesize that one of the largest chromosomes underwent fission at the terminal region, generating the chromosomes 10 and 3. Chromosome 10 would retain the region where the AbipSat02-497 satDNA accumulates, while chromosome 3 would lack this region at its end, which matches the distribution pattern of this satellite observed using the CHRISMAPP pipeline.

Moreover, in order to address the chromosomal fusion/fission events between both *Adalia* species, we applied the syntenyPlotteR pipeline [76]. The results obtained (Figure 10B) fully support the previous hypothesis. This analysis shows the homology between chromosome 2 of *A. bipunctata* and chromosomes 3 and 10 of *A. decempunctata*, with chromosome 10 showing homology with the terminal region of chromosome 2 of *A. bipunctata*. In addition to this chromosomal rearrangement, the synteny analysis revealed the existence of two extensive chromosomal inversions between chromosome 2 of *A. bipunctata* and chromosome 3 of *A. decempunctata*. It seems that there have been no additional chromosomal fusion or fission events between the two karyotypes, as each chromosome of *A. bipunctata* presents homology with a single chromosome of *A. decempunctata*. However, it is possible to observe the existence of chromosomal inversions between some pairs of orthologous chromosomes, such as between the chromosomes 1s of both species.

The results from CHRISMAPP analysis were also utilized to compare the sex chromosomes in both species. In the Y chromosome of *A. bipunctata*, 12 satDNA families were identified (Supplementary Figure S7). Additionally, this chromosome presents another satDNA, AbipSat06-176, which was not detected in the Y chromosome assembly but whose presence was determined by FISH (Figure 9). Among these 13 satDNAs, two form the heterochromatin of the autosomes and the X chromosome (AbipSat01-187, AbipSat03-8), 10 are shared between the Y chromosome and the autosomes or the X chromosome (AbipSat06-176, AbipSat08-316, AbipSat18-14, AbipSat23-376, AbipSat26-2235, AbipSat31-145, AbipSat32-163, AbipSat43-282, AbipSat58-518, and AbipSat65-286). One satDNA family seems to be present exclusively on the Y chromosome (AbipSat10-1902) (Supplementary Figure S4). Using CHRISMAPP, five of the satDNA families present in *A. bipunctata* were detected on the Y chromosome of *A. decempunctata* (Supplementary Figure S7). Four of these are shared between the Y chromosomes of both species (AbipSat08-316, AbipSat10-1902, AbipSat18-14, AbipSat23-376). The fifth family present in the Y chromosome of *A. decempunctata* is AbipSat34-137. This satDNA is present on the autosomes and the X chromosome of both species but does not appear to be present on the Y chromosome of *A. bipunctata*. The AbipSat10-1902 family is highly amplified in the Y chromosome of *A. decempunctata*, although in this species, it is not specific to the Y chromosome since short arrays of this satDNA can be found on some autosomes (Supplementary Figure S6).

Additionally, AbipSat19-1237, which is specific to the X chromosomes in *A. bipunctata*, is also specific to the X chromosome in *A. decempunctata*. It is widely believed that sex chromosomes originated from a pair of autosomes. Over time, these chromosomes began to differentiate, especially the Y chromosome (or the W in the ZZ/ZW system) due to the accumulation of repetitive sequences [77]. This process is facilitated by the complete lack of recombination in the Y chromosome, causing it to differentiate more rapidly than autosomes. This observation may explain why more satDNA families in *A. bipunctata* are conserved among the autosomes of both species (69 out of 85) compared to the Y chromosomes (4 out of 13). The X chromosomes in these species recombine in females, which helps them avoid the mutational degradation experienced by Y chromosomes, thus maintaining greater similarity to autosomes. However, their different dosage between sexes can influence their evolutionary dynamics differently from autosomes [78]. The presence of an X chromosome-specific satDNA family in both *Adalia* species could be a sign of this differentiation process compared to autosomes, although both X chromosomes are highly conserved among the two *Adalia* species, as demonstrated by the synteny analysis of the genomes of both species. Nevertheless, further studies on these species and related ones are needed to confirm this possible evolutionary pathway.

## 3. Materials and Methods

### 3.1. Insects, Preparation of Chromosome Spreads, and C-Banding

*A. bipunctata* samples were provided by the company Control Bio (https://controlbio.es/es/, accessed on 3 April 2024). Testes were removed from the males, immersed in distilled water for 45 min, fixed in ethanol and glacial acetic acid (3:1, *v*/*v*) and stored at −20 °C until use. Chromosome preparations were obtained as described in Lorite et al. [79]. The insect bodies were preserved in 100% ethanol at −20 °C for the DNA extraction.

The C-banding technique was performed according to the method described by Sumner [80], with small modifications [35]. The preparations were stained with 5% Giemsa or with 4′-6-diamino-2-phenylindole (DAPI) (Roche, Applied Science, Basel, Switzerland), at a concentration of 0.75 µg/mL.

### 3.2. Satellitome Analysis

*A. bipunctata* total genomic DNA (about 4 μg) was extracted from males using the NucleoSpin Tissue kit (Macherey-Nagel GmbH & Co., Düren, Germany), following the manufacturer’s instructions. The genome was sequenced using the Illumina^®^ Hiseq™ 2000 platform at Macrogen Inc. (Seoul, Republic of Korea), with a paired-end sequencing of 101 bp reads. It yielded a total of 23,103,102 paired-end reads that corresponded to more than 2.3 Gb of raw data. Illumina Raw reads were quality trimmed, and the adaptors from the sequencing were also removed using Trimmomatic v0.39 [81]. The cleaned and trimmed reads were analyzed using RepeatExplorer2 v2.3.7 [19,20], implemented within the Galaxy platform (https://galaxyelixir.vm.cesnet.cz/galaxy/, accessed on 10 June 2024) which includes TAREAN [21].

From the total of obtained sequences, a set of 12,000,000 paired-end reads (about 1.2 Gb) were randomly selected and used as input for RepeatExplorer2 pipeline. From those 1.2 Gb as input, RepeatExplorer used in the analysis a total of 3,880,249 (about 392 Mb). The genome size of *A. bipunctata* is about 475 Mb [36]. Therefore, the 392 Mb used by RepeatExplorer2 comprised a coverage of about 0.8×, near the recommended coverage (at least 0.1×–0.5×, or up to the 1.5× coverage for repeat-poor species) [39]. To run RepeatExplorer, default options were used, i.e., a minimum overlap of 55% and a similarity higher than 90%. We selected the long computing time and a percentage of abundance of at least 0.001% of the genome. To identify all the potential satellite repeats, we analyzed those clusters with sphere or ring-like shapes within the top clusters. In order to identify the specific clusters that contain satellite satDNA sequences, and to determine the size of the repeat unit (or monomer), as well as the consensus sequence for each family, Geneious Pro v.4.8.5 [82] was used. The final consensus was obtained by assembling all reads of the cluster. A BLAST all-to-all analysis with blastn and an e-value of 0.001 options was performed in order to identify similarities between all satDNA families. All the consensus sequences from all the potential satDNAs were used as query in Repbase using CENSOR (http://www.girinst.org/, accessed on 10 June 2024) [83] and were analyzed and compared with the GenBank/NCBI DNA databases using the BLAST network service and the EMBL database [84].

The satDNA families were named following a nomenclature similar to that proposed by Ruiz-Ruano et al. [18], including the first letter of the genus name, the first three letters of the species name, followed by ‘Sat’, a number indicating the family in order of abundance, and the monomer size for each satDNA family.

The abundance and divergence for each satDNA were determined using RepeatMasker v4.1.4 [85] with the “-a” option and the RMBlast search engine. To perform the analysis, we randomly selected five million raw reads, then we aligned them against the whole collection of satDNA dimers (for those families with a Repetition Unit Length (RUL) of more than 100 bp) or monomer concatenations until 200 bp in length (for those families with a RUL shorter than 100 bp). The average divergence and the satDNA landscape were generated considering distances from the sequences by applying the Kimura 2-parameter model with the Perl script calcDivergenceFromAlign.pl from the RepeatMasker suite. The same process was also utilized to mask the satDNA sequences in the chromosome-level genome assembly published for *A. bipunctata*, with the objective of determining their presence, abundance, and divergence. Then, both general and individual landscapes were plotted using a custom script in Rstudio v4.1.0 [86] and the ggplot2 package v3.4.4 [87].

In order to compare and analyze the presence of all the satDNAs of *A. bipunctata* in *A. decempunctata*, the profile of each satDNA family was generated using the RepeatProfiler tool (https://github.com/johnssproul/RepeatProfiler, accessed on 18 June 2024) [88]. This tool is used to create the coverage and base pair composition profile using Illumina sequencing reads. As reference, we concatenated monomers of *A. bipunctata* into trimers (or at least monomers up to 200 bp for those satDNA sequences shorter than 100 bp). The default options were selected, with the “-p” option in order to take as input the paired-end reads. After this analysis, the proportion of each shared satDNA family was calculated using RepeatMasker, following the approach mentioned previously. Illumina reads were retrieved from NCBI accession number SRX18975475.

### 3.3. Cytogenetic Mapping by Fluorescence In Situ Hybridization (FISH) and by Chromosome In Silico Mapping (CHRISMAPP) and Synteny Analysis

Specific oligonucleotides were designed based on the most abundant satDNA families (Table 2) and used as probes for FISH. Oligonucleotides were labelled with either biotin-16-dUTP (Roche) or digoxigenin-11-dUTP (Roche) using terminal transferase (Roche) and following the instructions provided by the supplier.

FISH was performed as described in Cabral-de-Mello and Marec [89], with some modifications [35]. The immunological detection was performed with Streptavidin-Alexa Fluor™ 488 (Thermo Fisher Scientific, Waltham, MA, USA) (10 μg/mL) for those satDNAs labelled with Biotin, and with Anti-Digoxigenin-Rhodamine (Roche) (1 μg/mL) for those labelled with Digoxigenin. Slides were mounted with VECTASHIELD–DAPI (Vector Laboratories, Burlingame, CA, USA). DAPI in the antifade solution was used to counterstain the chromosomes. Images were made and analyzed using a BX51 Olympus^®^ fluorescence microscope (Olympus, Hamburg, Germany) equipped with a CCD camera (Olympus^®^ DP70). Image acquisition and processing were carried out using DP Manager software v1.1.1.71 and Adobe Photoshop CS4 software v11.0 (Adobe Systems, San Jose, CA, USA).

For the amplification of hybridization signals from the AbipSat04-193 and AbipSat05-258 satDNA families, which did not show a distinct visible hybridization signal with the initial protocol, the avidin-FITC/antiavidin-biotin system [90] was utilized, with two rounds of amplification [35].

The chromosomal locations of all 86 satDNA families were determined in silico using the CHRISMAPP pipeline [35] (https://github.com/LoriteLab/CHRISMAPP, accessed on 10 June 2024). We used the available chromosome-level genome assembly of *A. bipunctata* [36] and the unassembled scaffolds as reference, and we mapped the satDNAs using their consensus sequences. In order to obtain an overview about the chromosome rearrangements in the *Adalia* genus, we also applied the CHRISMAPP pipeline in *A. decempunctata* genome assembly. Briefly, we took the whole collection of satDNA sequences from *A. bipunctata* to obtain their chromosomal positions in *A. decempunctata* chromosomes. For this analysis, we took only complete pseudochromosomes and not unplaced scaffolds.

Moreover, in order to address the chromosomal fusion/fission events between species, we applied the syntenyPlotteR v1.0.0 pipeline [76]. We retrieved the BUSCO tables from A3Cat [91] using the Arthropoda database from BUSCO. In order to generate the linear plot graph, the tables from BUSCO containing the chromosomal positions of each BUSCO gene were used. The chromosomal positions of each single copy gene from each species were merged by gene name, taking only the “complete” genes in each species.

## 4. Conclusions

The satellitome of *A. bipunctata* has been characterized using sequencing data, identifying 86 different satDNA families. Bioinformatics localization showed that all satDNA families are present in short arrays in euchromatin, while only two or three satDNA families are found in long arrays that form heterochromatin.

Estimations based on Illumina reads and assembled chromosome sequences revealed an underestimation of satDNA quantity in the assembled chromosomes, especially those forming heterochromatin, as conclusively demonstrated in our study. Cytogenetic analysis using FISH supports this finding, emphasizing its importance in molecular genome assembly studies.

Genomic analysis of two *Adalia* species revealed high conservation of their satDNA families, although with differing amounts for the shared satDNAs. The most notable difference was the absence in *A. decempunctata* of the most abundant satDNA of *A. bipunctata*, which in this last species is the main component of its heterochromatin, suggesting rapid turnover in centromeric heterochromatin satDNA composition. Specific satDNA families found in sex chromosomes of both species suggest a role in sex chromosome differentiation, instead of the gene conservation on the X chromosome. Comparative analyses of satDNA and synteny indicated that chromosomal rearrangements, likely fission events, have played a role in the evolutionary changes in their karyotypes.

In summary, the integrated cytogenetic and bioinformatics analysis enabled a detailed characterization of satellitomes in related *Adalia* species, thereby advancing the understanding of repeated genome organization in beetles.

## Figures and Tables

**Table 1:** Names, A+T content, abundances and divergence values from *A. bipunctata* satellitome using Illumina raw reads, chromosome assembly and chromosome assembly plus unplaced scaffolds. ND = non-detected. NCBI accession numbers PP944632–PP944717.

SatDNA Families	% A+T	Illumina Reads		Genome Assembly	
		Genome (%)	Divergence (Kimura-2p)	Chromosomes (%)	Chromosomes + Unplaced Scaffolds (%)
AbipSat01-187	70.1	9.9808	7.91	3.2895	7.0636
AbipSat02-497	64.8	1.6789	15.99	1.8672	1.7659
AbipSat03-8	87.5	0.3864	2.57	0.2758	0.5723
AbipSat04-193	55.4	0.2678	6.62	0.2257	0.2134
AbipSat05-258	65.9	0.2383	6.91	0.2325	0.2199
AbipSat06-176	74.4	0.2328	7.01	0.0019	0.1960
AbipSat07-579	63.4	0.1054	6.22	0.1396	0.1323
AbipSat08-316	64.9	0.1017	8.56	0.1349	0.1276
AbipSat09-473	68.7	0.0916	18.10	0.1705	0.1613
AbipSat10-1902	57.1	0.0874	1.52	0.1012	0.1100
AbipSat11-151	62.3	0.0816	9.31	0.1029	0.0976
AbipSat12-233	62.2	0.0810	2.16	0.0268	0.0254
AbipSat13-1767	66.3	0.0799	7.13	0.1924	0.1823
AbipSat14-53	73.6	0.0751	3.25	0.0001	0.0008
AbipSat15-179	65.4	0.0712	17.93	0.0957	0.0905
AbipSat16-443	56.7	0.0660	10.05	0.0946	0.0920
AbipSat17-86	69.8	0.0642	22.80	0.0826	0.0781
AbipSat18-14	64.3	0.0636	6.85	0.0664	0.0641
AbipSat19-1237	63.3	0.0618	3.28	0.1174	0.1110
AbipSat20-176	61.9	0.0550	10.94	0.0540	0.0543
AbipSat21-1899	65.4	0.0537	8.61	0.0796	0.0759
AbipSat22-148	57.4	0.0527	4.00	0.0455	0.0431
AbipSat23-376	58.8	0.0474	11.11	0.1068	0.1024
AbipSat24-5-TEL	60.0	0.0434	3.63	0.0068	0.0137
AbipSat25-176	63.6	0.0416	18.96	0.0369	0.0349
AbipSat26-2235	60.1	0.0398	3.47	0.0549	0.0544
AbipSat27-84	61.9	0.0352	23.48	0.0007	0.0006
AbipSat28-165	67.9	0.0330	16.69	0.0432	0.0408
AbipSat29-174	64.9	0.0282	23.19	0.0369	0.0349
AbipSat30-212	59.0	0.0278	21.05	0.0047	0.0057
AbipSat31-145	63.4	0.0241	8.06	0.0353	0.0335
AbipSat32-163	60.1	0.0237	8.55	0.0310	0.0321
AbipSat33-284	62.7	0.0228	11.54	0.0339	0.0322
AbipSat34-137	70.1	0.0223	10.47	0.0354	0.0337
AbipSat35-141	56.0	0.0218	4.68	0.0167	0.0158
AbipSat36-157	47.8	0.0194	4.49	0.0364	0.0345
AbipSat37-297	65.3	0.0153	11.24	0.0221	0.0209
AbipSat38-2435	58.2	0.0141	4.60	0.0210	0.0204
AbipSat39-159	60.4	0.0132	6.50	0.0103	0.0097
AbipSat40-152	61.8	0.0131	11.87	0.0109	0.0105
AbipSat41-69	53.6	0.0117	4.52	0.0069	0.0067
AbipSat42-21	61.9	0.0116	18.70	0.0330	0.0313
AbipSat43-282	69.9	0.0111	7.37	0.0086	0.0092
AbipSat44-148	56.8	0.0110	8.71	0.0138	0.0130
AbipSat45-307	64.5	0.0109	5.95	0.0088	0.0083
AbipSat46-126	50.8	0.0089	2.36	0.0061	0.0058
AbipSat47-399	60.7	0.0080	5.88	0.0162	0.0153
AbipSat48-18	50.0	0.0080	7.15	ND	ND
AbipSat49-148	60.8	0.0075	8.48	0.0091	0.0086
AbipSat50-199	55.8	0.0072	12.04	0.0042	0.0040
AbipSat51-128	60.9	0.0071	4.80	0.0055	0.0052
AbipSat52-177	66.7	0.0064	8.37	0.0089	0.0084
AbipSat53-22	50.0	0.0059	15.97	0.0035	0.0033
AbipSat54-271	65.3	0.0058	6.28	0.0002	0.00350
AbipSat55-145	62.1	0.0054	4.12	0.0085	0.0082
AbipSat56-30	46.7	0.0051	9.38	ND	ND
AbipSat57-144	58.3	0.0049	14.89	0.0040	0.0039
AbipSat58-518	60.0	0.0047	7.88	0.0060	0.0062
AbipSat59-105	55.2	0.0039	1.37	0.0030	0.0026
AbipSat60-160	60.0	0.0038	3.13	0.0020	0.0020
AbipSat61-175	68.0	0.0038	8.32	0.0070	0.0064
AbipSat62-144	55.6	0.0037	2.52	0.0020	0.0019
AbipSat63-186	61.8	0.0031	4.08	0.0020	0.0017
AbipSat64-201	62.2	0.0031	5.24	0.0020	0.0023
AbipSat65-286	59.1	0.0029	5.91	0.0040	0.0036
AbipSat66-169	62.1	0.0028	1.26	ND	ND
AbipSat67-304	67.8	0.0027	2.01	0.0020	0.0019
AbipSat68-237	54.9	0.0025	6.66	0.0006	0.0006
AbipSat69-438	63.5	0.0025	2.03	0.0020	0.0019
AbipSat70-168	56.0	0.0024	3.23	0.0009	0.0008
AbipSat71-438	61.9	0.0023	1.93	0.0012	0.0011
AbipSat72-426	64.1	0.0022	2.45	0.0019	0.0018
AbipSat73-396	71.0	0.0020	7.27	0.0012	0.0011
AbipSat74-344	61.0	0.0019	7.28	0.0023	0.0022
AbipSat75-471	61.6	0.0019	7.51	0.0011	0.0011
AbipSat76-140	76.4	0.0018	4.76	0.0006	0.0005
AbipSat77-155	54.8	0.0018	4.58	0.0024	0.0022
AbipSat78-399	63.9	0.0017	4.18	0.0021	0.0020
AbipSat79-309	67.0	0.0017	1.39	0.0034	0.0032
AbipSat80-337	57.3	0.0016	3.51	0.0019	0.0018
AbipSat81-258	66.7	0.0016	3.95	0.0006	0.0005
AbipSat82-291	59.8	0.0014	2.87	0.0009	0.0008
AbipSat83-191	64.9	0.0012	0.93	0.0009	0.0008
AbipSat84-169	52.7	0.0011	0.57	0.0005	0.0004
AbipSat85-284	65.1	0.0011	5.45	0.0010	0.0009
AbipSat86-294	61.9	0.0009	3.25	0.0016	0.0015
Satellite proportion		14.6677		8.1351	12.1966

**Table 2:** Oligonucleotides used for FISH analyses in the chromosomes of *A. bipunctata*.

SatDNA Family	Oligonucleotide Name	Sequence
AbipSat01-187	Abip-CL1-F	ACTGCTGTGAATAATCGG
	Abip-CL1-R	GACTTTTTGATCTGAGGG
AbipSat02-497	Abip-CL4-F	AATATGGTGTTTTTGGGG
	Abip-CL4-R	TTTCACCTGTCAAATGGC
AbipSat03-8	AbipCL-13	GTCAAATGTCATCTGTCAAA
AbipSat04-193	Abip-CL46-F	CAAATTTTTTCTCTTCGC
	Abip-CL46-R	ACCATGCATGCTGTATTG
AbipSat05-258	Abip-CL42-F	TTTCGAAAAATTATATTCC
	Abip-CL42-R	CTTTGTAGCTCAATTGTTC
AbipSat06-176	Abip-CL46-RE2-F	AACGAATTCCACCAAGGG
	Abip-CL46-RE2-R	TCGTTCAAGTAGCCGAGC

## Data Availability

The data presented in the study are available in the article. The CHRISMAPP script is also available in the GitHub repository (https://github.com/LoriteLab/CHRISMAPP, accessed on 20 June 2024).

## Supplementary Materials

The following supporting information can be downloaded at: https://www.mdpi.com/article/10.3390/ijms25179214/s1.
