# Supplementary material for: Satellitome Analysis of Adalia bipunctata (Coleoptera): Revealing Centromeric Turnover and Potential Chromosome Rearrangements in a Comparative Interspecific Study
Source: Int J Mol Sci. 2024 Aug 25;25(17):9214. doi: 10.3390/ijms25179214 (PMC11394905; doi:10.3390/ijms25179214)
Supplement: Supplementary file 1 [file ijms-25-09214-s001.zip › Supplementary Figure S2 landscape.pdf]

Supplementary Figure S2. Satellite DNA landscapes for all satDNA families found in *Adalia bipunctata*.

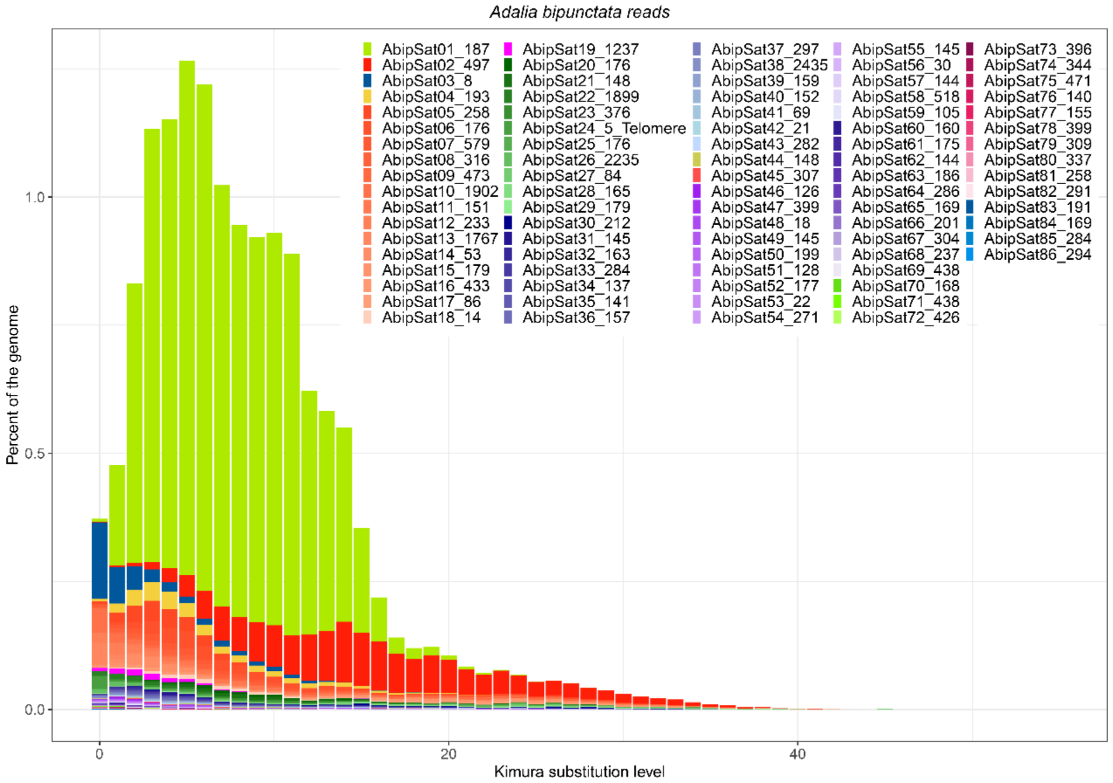

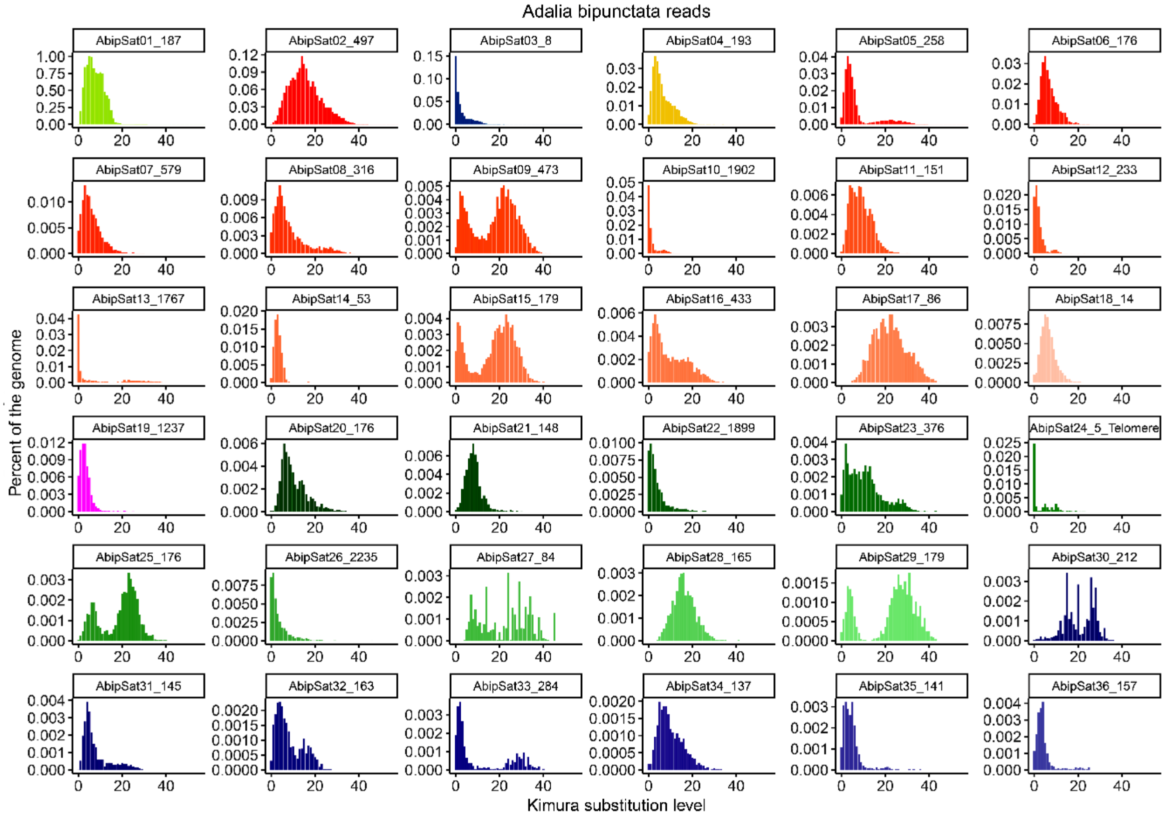

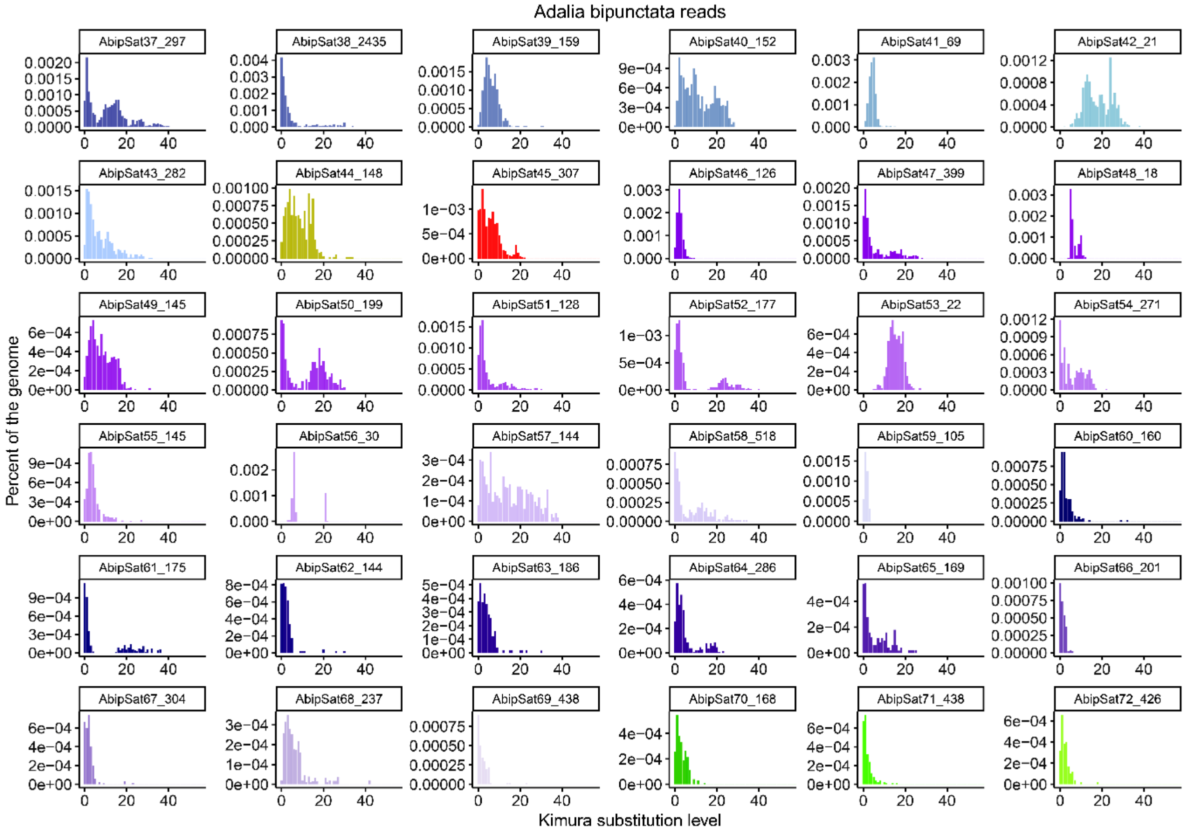
Adalia bipunctata reads
AbipSat37_297
AbipSat38_2435
AbipSat39_159
AbipSat40_152
AbipSat41_69
AbipSat42_21
AbipSat43_282
AbipSat44_148
AbipSat45_307
AbipSat46_126
AbipSat47_399
AbipSat48_18
AbipSat49_145
AbipSat50_199
AbipSat51_128
AbipSat52_177
AbipSat53_22
AbipSat54_271
AbipSat55_145
AbipSat56_30
AbipSat57_144
AbipSat58_518
AbipSat59_105
AbipSat60_160
AbipSat61_175
AbipSat62_144
AbipSat63_186
AbipSat64_286
AbipSat65_169
AbipSat66_201
AbipSat67_304
AbipSat68_237
AbipSat69_438
AbipSat70_168
AbipSat71_438
AbipSat72_426
Percent of the genome
Kimura substitution level

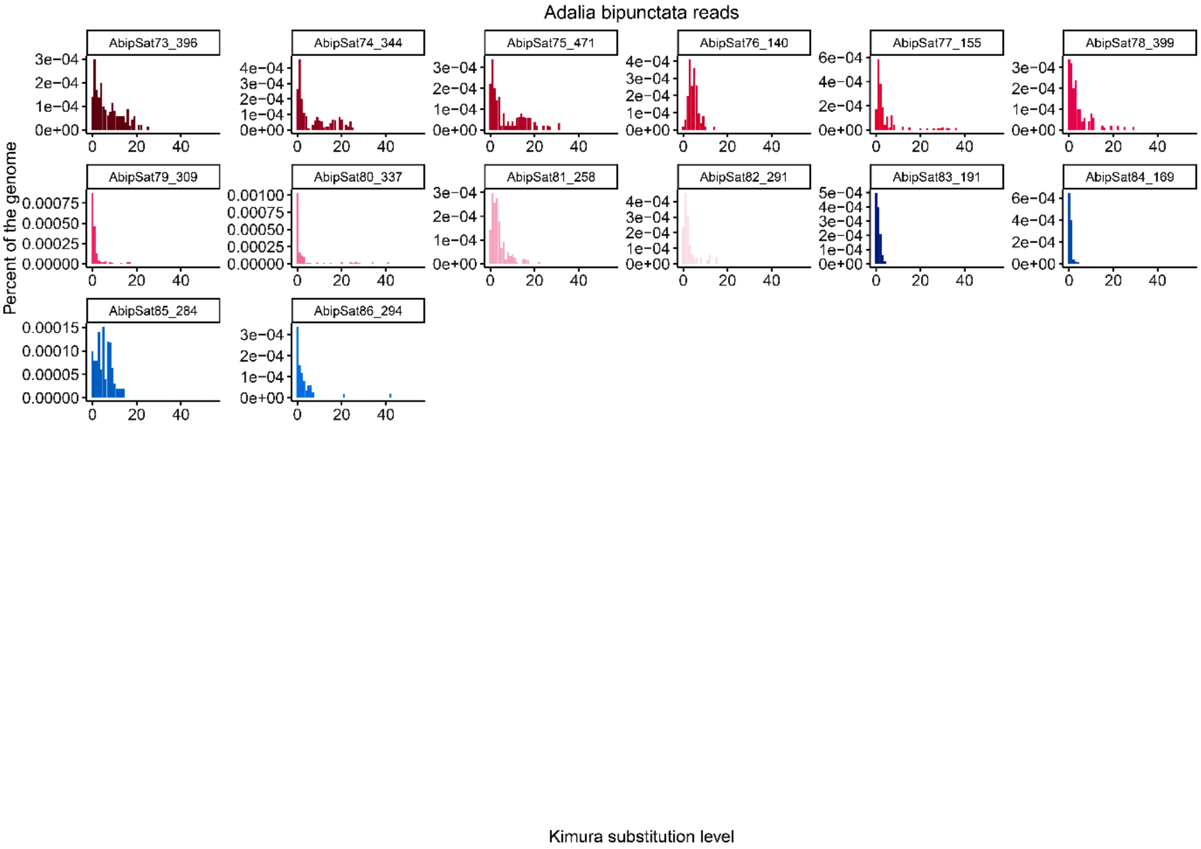
Adalia bipunctata reads
AbipSat73_396
AbipSat74_344
AbipSat75_471
AbipSat76_140
AbipSat77_155
AbipSat78_399
AbipSat79_309
AbipSat80_337
AbipSat81_258
AbipSat82_291
AbipSat83_191
AbipSat84_169
AbipSat85_284
AbipSat86_294
Percent of the genome
Kimura substitution level
