# Supplementary material for: Satellitome Analysis of Adalia bipunctata (Coleoptera): Revealing Centromeric Turnover and Potential Chromosome Rearrangements in a Comparative Interspecific Study
Source: Int J Mol Sci. 2024 Aug 25;25(17):9214. doi: 10.3390/ijms25179214 (PMC11394905; doi:10.3390/ijms25179214)
Supplement: Supplementary file 1 [file ijms-25-09214-s001.zip › Supplementary Figure S3 superfamilies.pdf]

Supplementary Figure S3. Alignments of superfamily members in the *Adalia bipunctata* satellitome. Asterisks indicate conserved positions.

```
AbipSat15-179      CAAGCTACAGTATTGAAACTATCTGGTGCAATATAGAGCGTTTATTCCTCTTTTGAAAAA      60
AbipSat25-176      CAAGCTACACTAGTGGAA-TTTCGGGCGTAATATAGAGCACTGCTTCCTCTTCACAAAAA      59
AbipSat29-174      CAAGCCACAGTAATGAAACTTT--GGCGGAACATAGAGCATCTATTCCTCTTGAGAAAAA      58
                   ***** *** ** ** ** * *  ** * ** *******     ********   *****

AbipSat15-179      AAATTACGAGGAGAGTTTGCGGCTGGTAAAATCAGGAGGAAAATGGAATAAATCTGAAAA      120
AbipSat25-176      AAATTACGAGAGGAGTCTGCGGTGGTAGGATAAGGA--GAAAAAAAATAAAATCTCCAAA      117
AbipSat29-174      ACATTACTTGGTGGATCTGCGAAGGTAAGATTAGGAGACACAAAA---TAAAATATAGAA      115
                   * *****  *  *  * ****   *    *         * **   *  ***      **

AbipSat15-179      AAAAAGTTTTGTTTTCATTCAATCCACAAACTTTGAAAATTCGTAGCTGTGTCTGTGGG       179
AbipSat25-176      AAAAAGTTTTTTATTTGTTCAATTCCTCAACTTTGAAAATTCGTAACTGTGTCTGTGGG       176
AbipSat29-174      AAAAGTTCTTTTTTTCACTAATTTCGCAAACTTTGAAACTCCGTAATTGCTTCTGTGTG       174
                   ****  * ** * **   * * * *   ********** * ****  **  ****** *
```

Superfamily 1

```
AbipSat52-177      ATATGGAGTTGGATTCGTCGGAGAAAATTCTTTTTTTGAAAAAAAAATTTTCGATTTTCT      60
AbipSat61-175      ATATTAATTTTTTATCGAGTTGTAAAAAAGTTTTTT-GGAAAAAAACGTTCACATTTT-T      58
                   ****  * **    ***      ****   ****** * *******  **   ***** *

AbipSat52-177      CGATTTTGTAAATTCCCTATGGCGTTTTGAATTAATTCAAAGCCATTTCTGAATAGAGTA      120
AbipSat61-175      CGTTCTCGTGGAATCCCTATGGCGTTTCGAATTAATTCATGAGCTTTTCTGAATAGAGTA      118
                   ** * * **  * ************** ***********    * ***************

AbipSat52-177      TTCTGTTGTCGATATTTCGGTGAGAAAATTATCCGTGAAGGTCCAACAGTTTCGGAG         177
AbipSat61-175      TTTCTTGGCTGACATTTCAGTGAAAAAATGATTCATTTACGACCAATAGTTCCGGAG         175
                   **   * *  ** ***** **** ***** ** * *  * * **** **** *****
```

Superfamily2
