# Supplementary material for: Satellitome Analysis of Adalia bipunctata (Coleoptera): Revealing Centromeric Turnover and Potential Chromosome Rearrangements in a Comparative Interspecific Study
Source: Int J Mol Sci. 2024 Aug 25;25(17):9214. doi: 10.3390/ijms25179214 (PMC11394905; doi:10.3390/ijms25179214)
Supplement: Supplementary file 1 [file ijms-25-09214-s001.zip › Supplementary Figure S4 CHRISMAPP A_bipunctata.pdf]

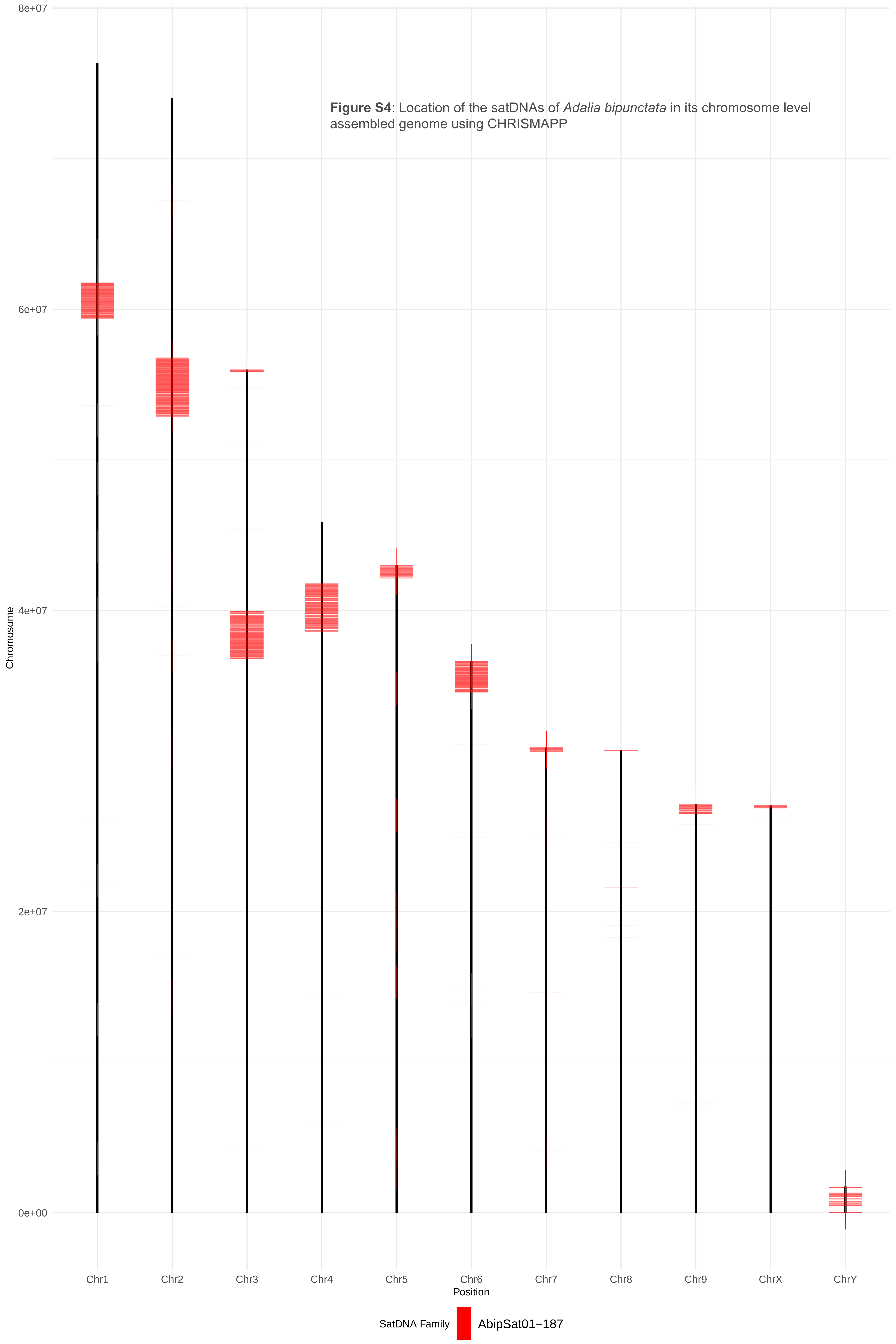
Figure S4: Location of the satDNAs of Adalia bipunctata in its chromosome level assembled genome using CHRISMAPP
8e+07
6e+07
4e+07
2e+07
0e+00
Chromosome
Chr1
Chr2
Chr3
Chr4
Chr5
Chr6
Chr7
Chr8
Chr9
ChrX
ChrY
Position
SatDNA Family
AbipSat01−187

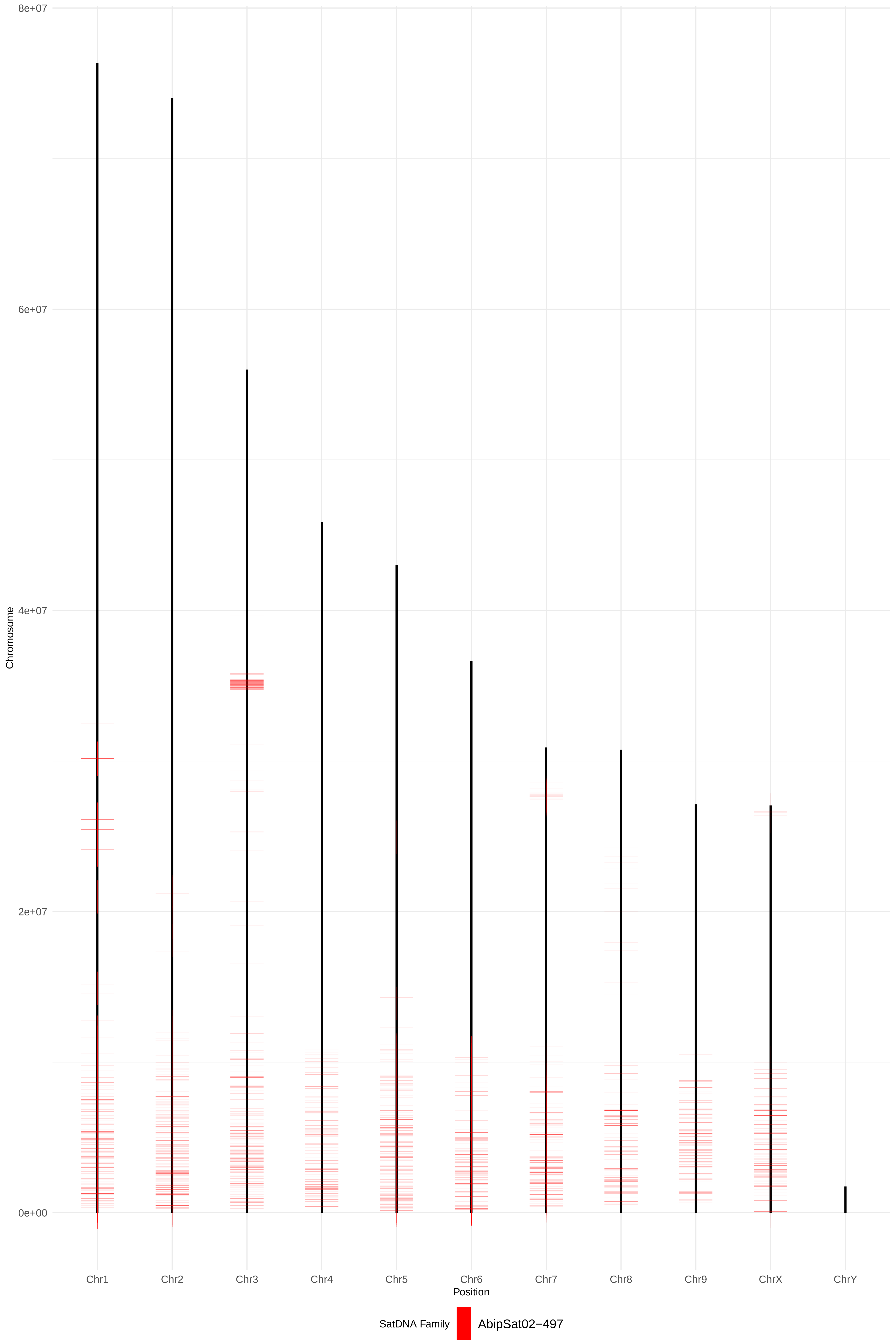

8e+07
6e+07
4e+07
2e+07
0e+00
Chromosome
Chr1
Chr2
Chr3
Chr4
Chr5
Chr6
Chr7
Chr8
Chr9
ChrX
ChrY
Position
SatDNA Family
AbipSat02−497

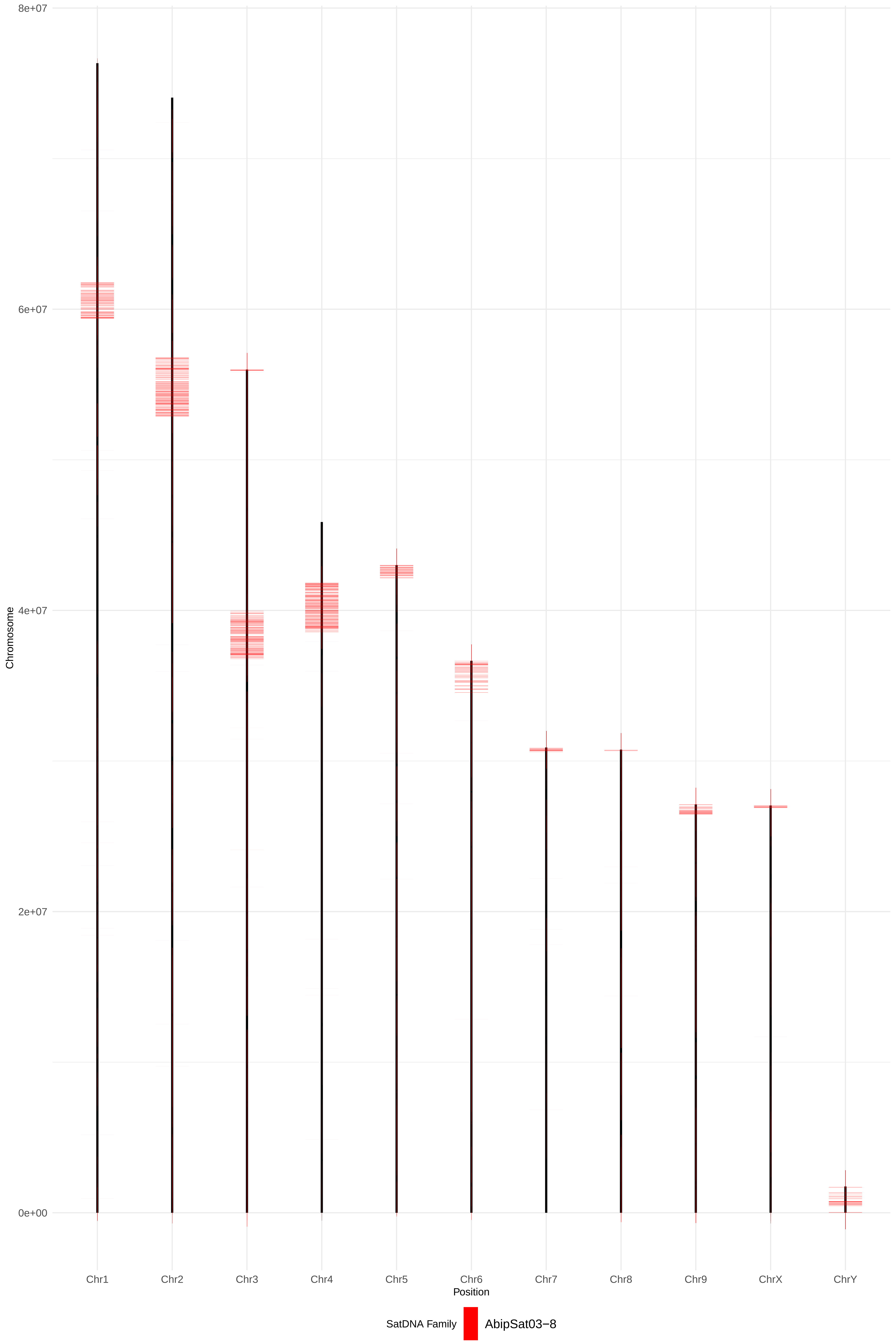
8e+07
6e+07
4e+07
2e+07
0e+00
Chromosome
Chr1
Chr2
Chr3
Chr4
Chr5
Chr6
Chr7
Chr8
Chr9
ChrX
ChrY
Position
SatDNA Family
AbipSat03−8

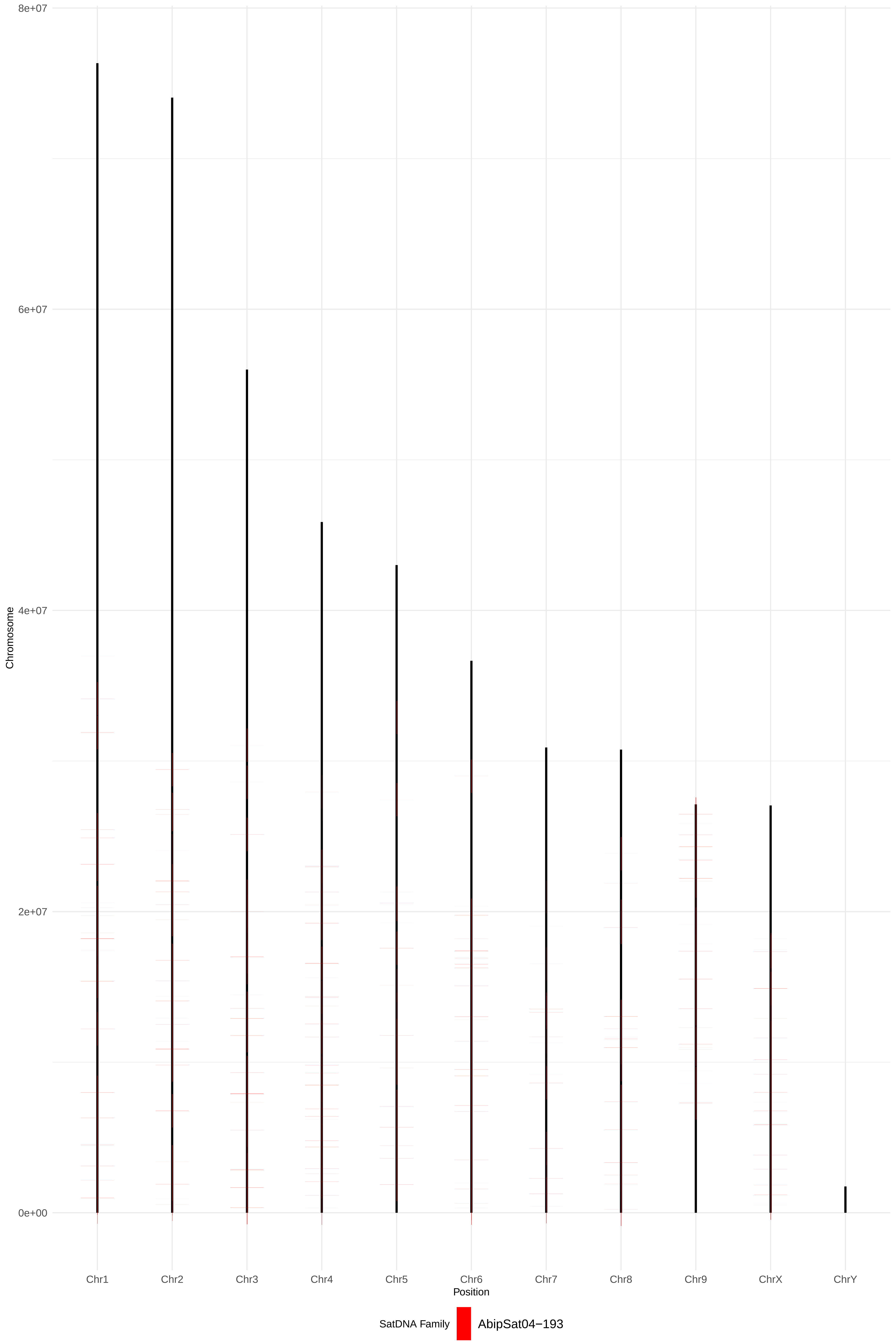

8e+07
6e+07
4e+07
2e+07
0e+00
Chromosome
Chr1
Chr2
Chr3
Chr4
Chr5
Chr6
Chr7
Chr8
Chr9
ChrX
ChrY
Position
SatDNA Family
AbipSat04−193

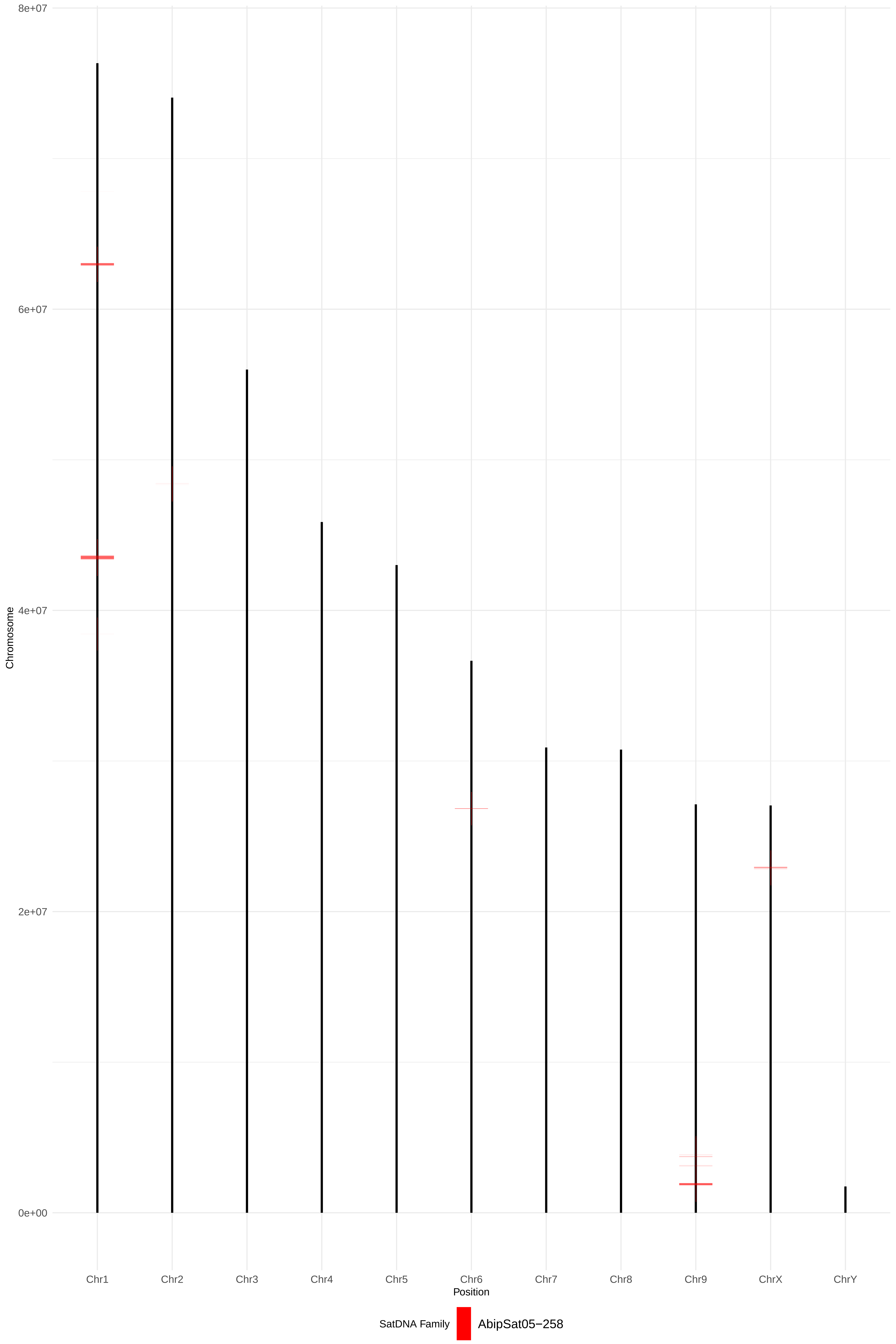

8e+07
6e+07
4e+07
2e+07
0e+00
Chromosome
Chr1
Chr2
Chr3
Chr4
Chr5
Chr6
Chr7
Chr8
Chr9
ChrX
ChrY
Position
SatDNA Family
AbipSat05−258

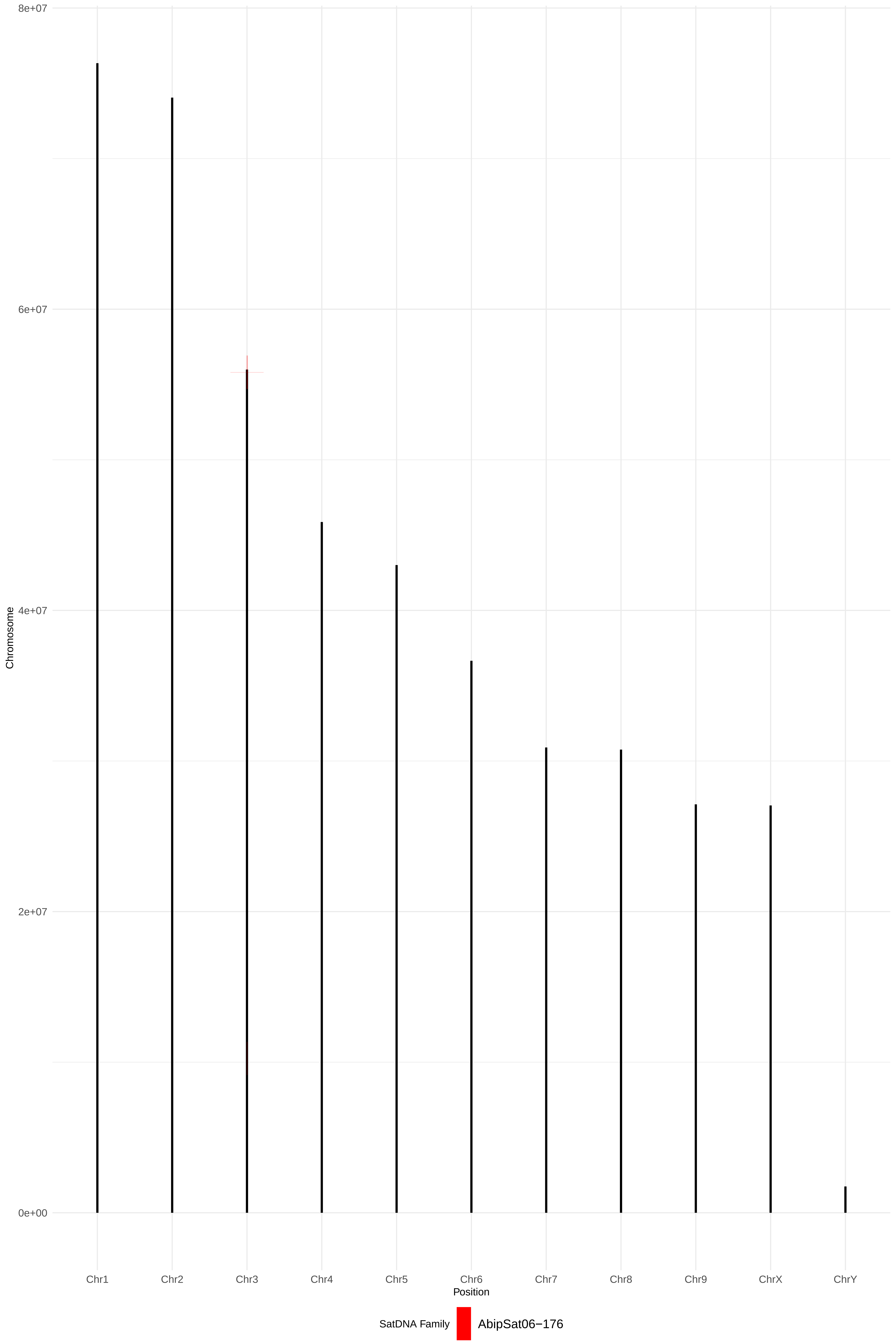

8e+07
6e+07
4e+07
2e+07
0e+00
Chromosome
Chr1
Chr2
Chr3
Chr4
Chr5
Chr6
Chr7
Chr8
Chr9
ChrX
ChrY
Position
SatDNA Family
AbipSat06−176

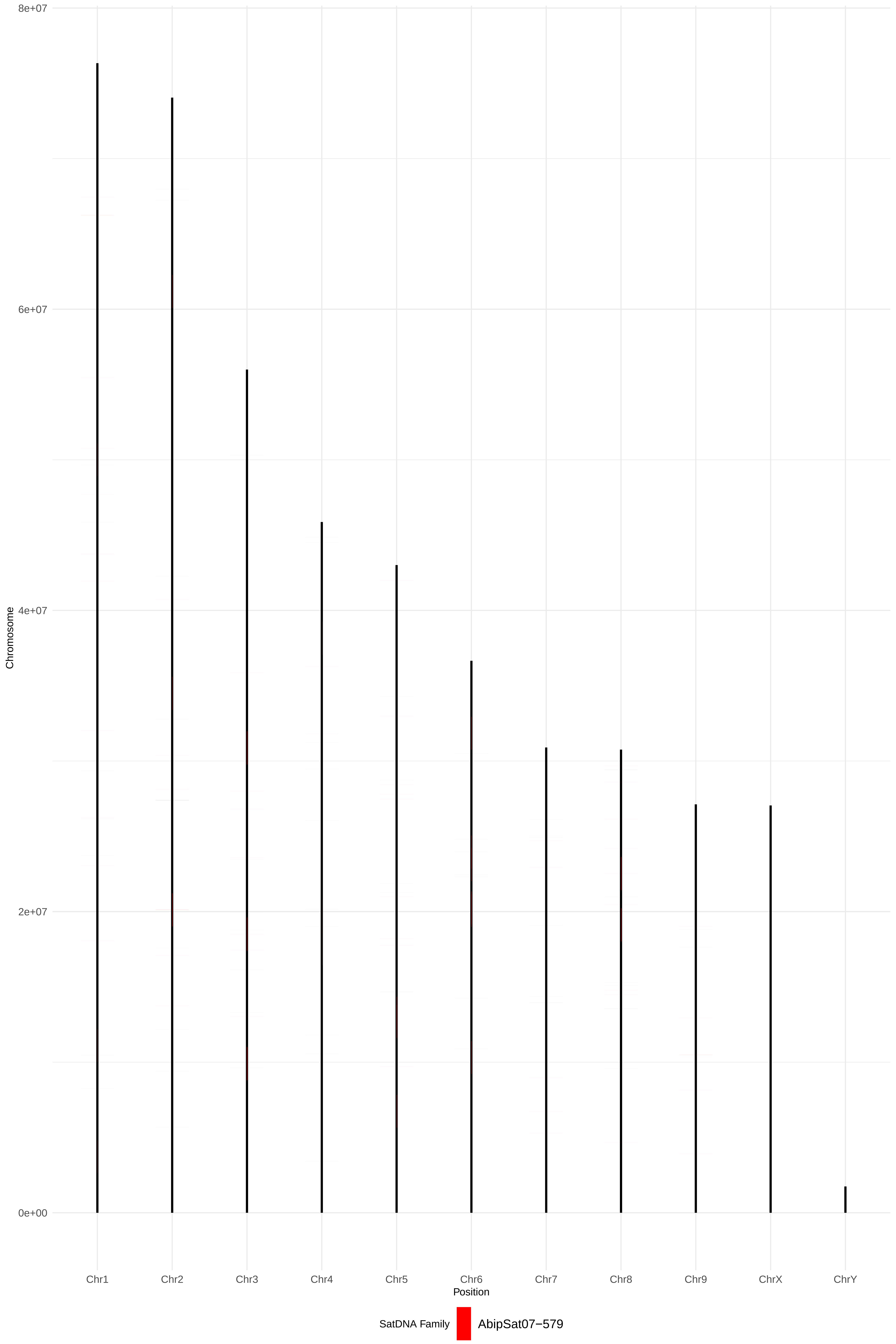

8e+07
6e+07
4e+07
2e+07
0e+00
Chromosome
Chr1
Chr2
Chr3
Chr4
Chr5
Chr6
Chr7
Chr8
Chr9
ChrX
ChrY
Position
SatDNA Family
AbipSat07−579

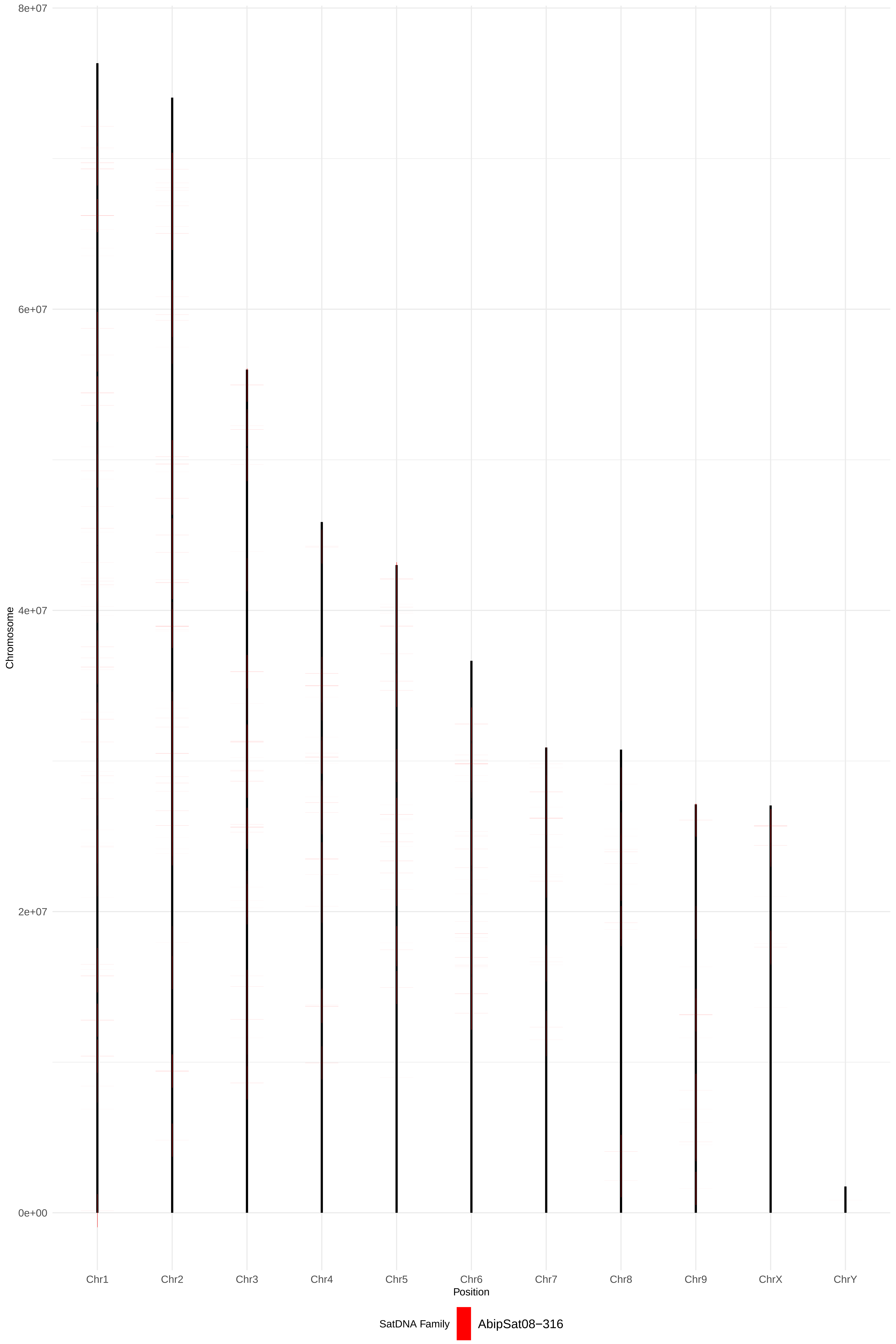

8e+07
6e+07
4e+07
2e+07
0e+00
Chromosome
Chr1
Chr2
Chr3
Chr4
Chr5
Chr6
Chr7
Chr8
Chr9
ChrX
ChrY
Position
SatDNA Family
AbipSat08−316

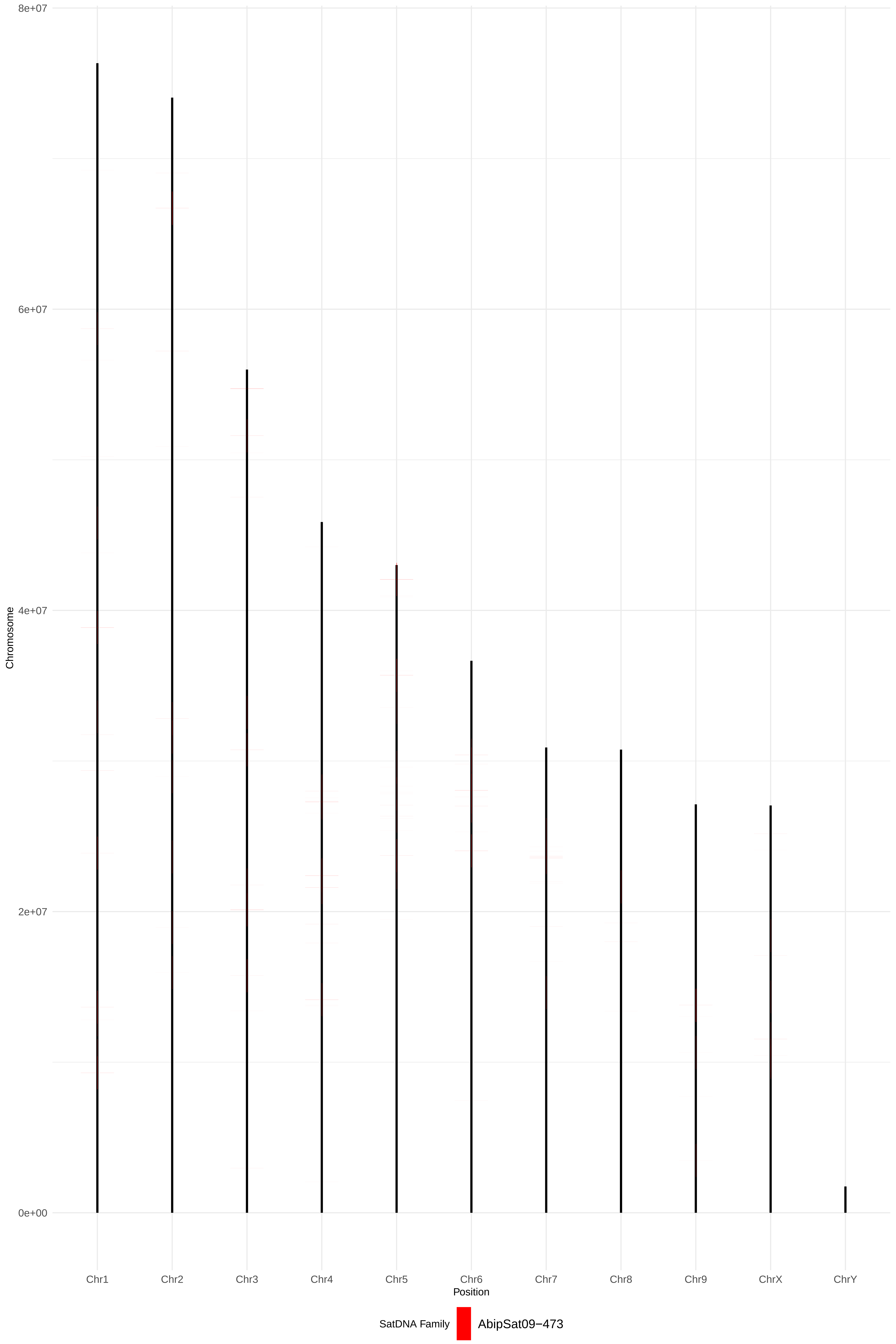

8e+07
6e+07
4e+07
2e+07
0e+00
Chromosome
Chr1
Chr2
Chr3
Chr4
Chr5
Chr6
Chr7
Chr8
Chr9
ChrX
ChrY
Position
SatDNA Family
AbipSat09−473

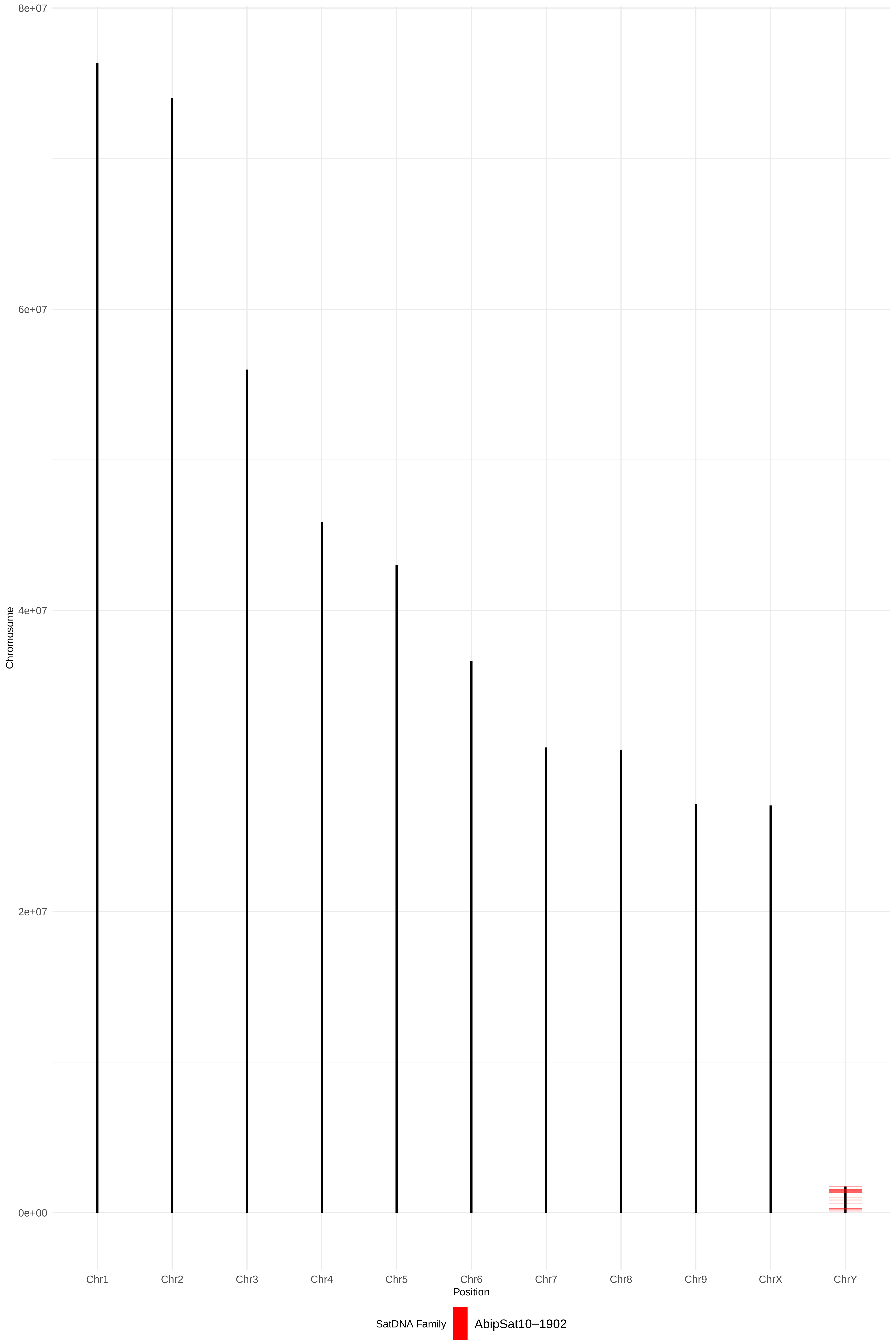

8e+07
6e+07
4e+07
2e+07
0e+00
Chromosome
Chr1
Chr2
Chr3
Chr4
Chr5
Chr6
Chr7
Chr8
Chr9
ChrX
ChrY
Position
SatDNA Family
AbipSat10−1902

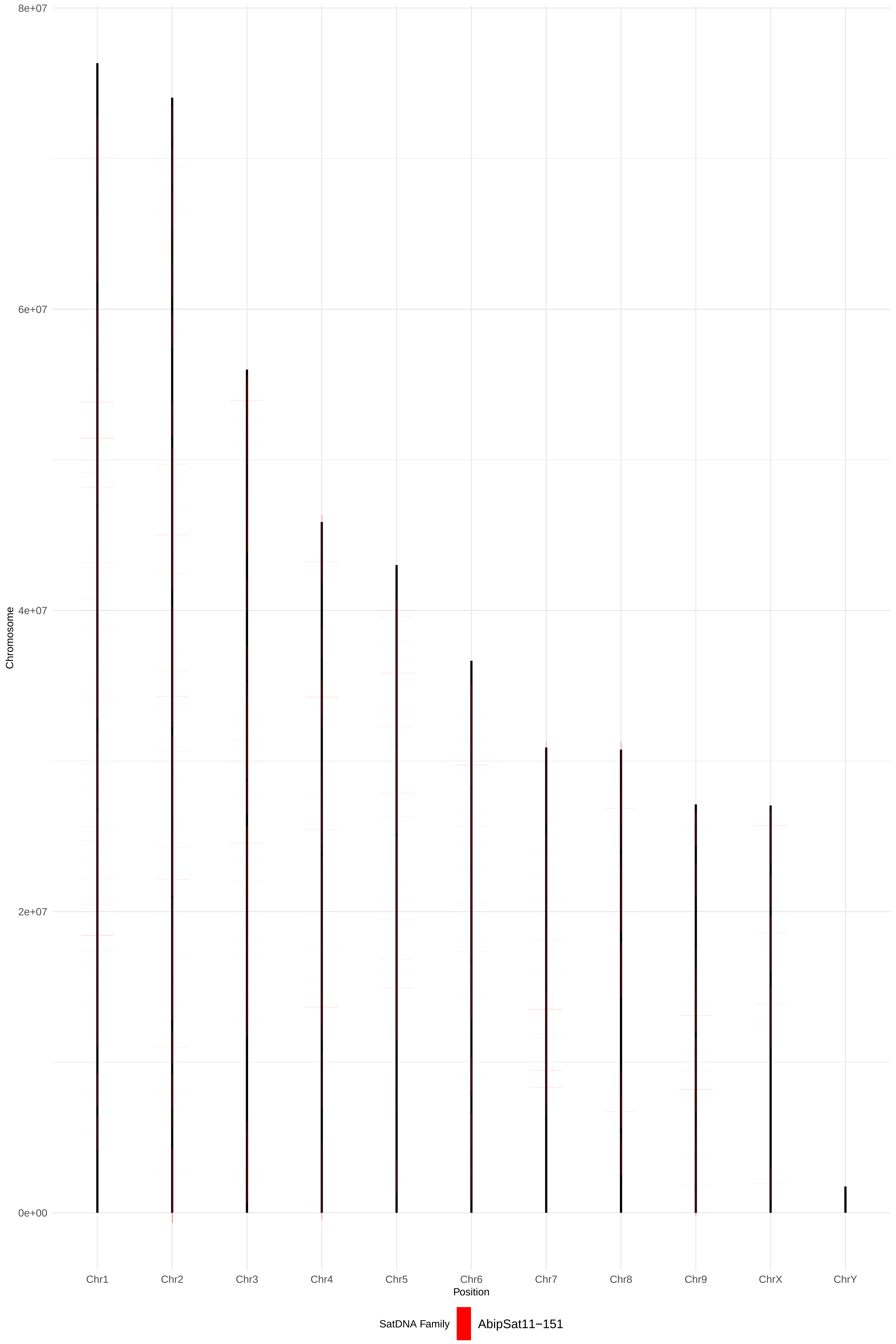
8e+07
6e+07
4e+07
2e+07
0e+00
Chromosome
Chr1
Chr2
Chr3
Chr4
Chr5
Chr6
Chr7
Chr8
Chr9
ChrX
ChrY
Position
SatDNA Family
AbipSat11−151

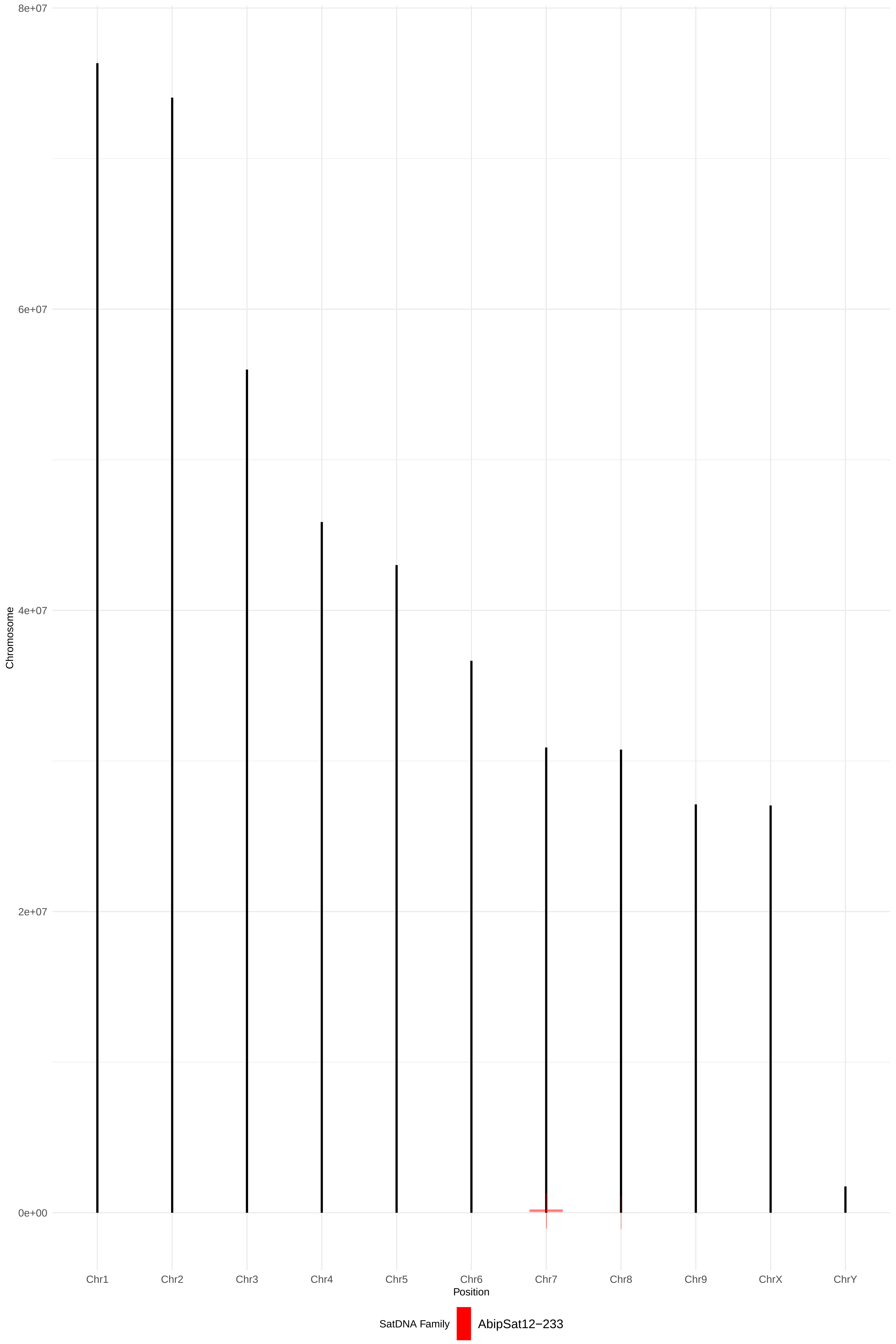
8e+07
6e+07
4e+07
2e+07
0e+00
Chromosome
Chr1
Chr2
Chr3
Chr4
Chr5
Chr6
Chr7
Chr8
Chr9
ChrX
ChrY
Position
SatDNA Family
AbipSat12−233

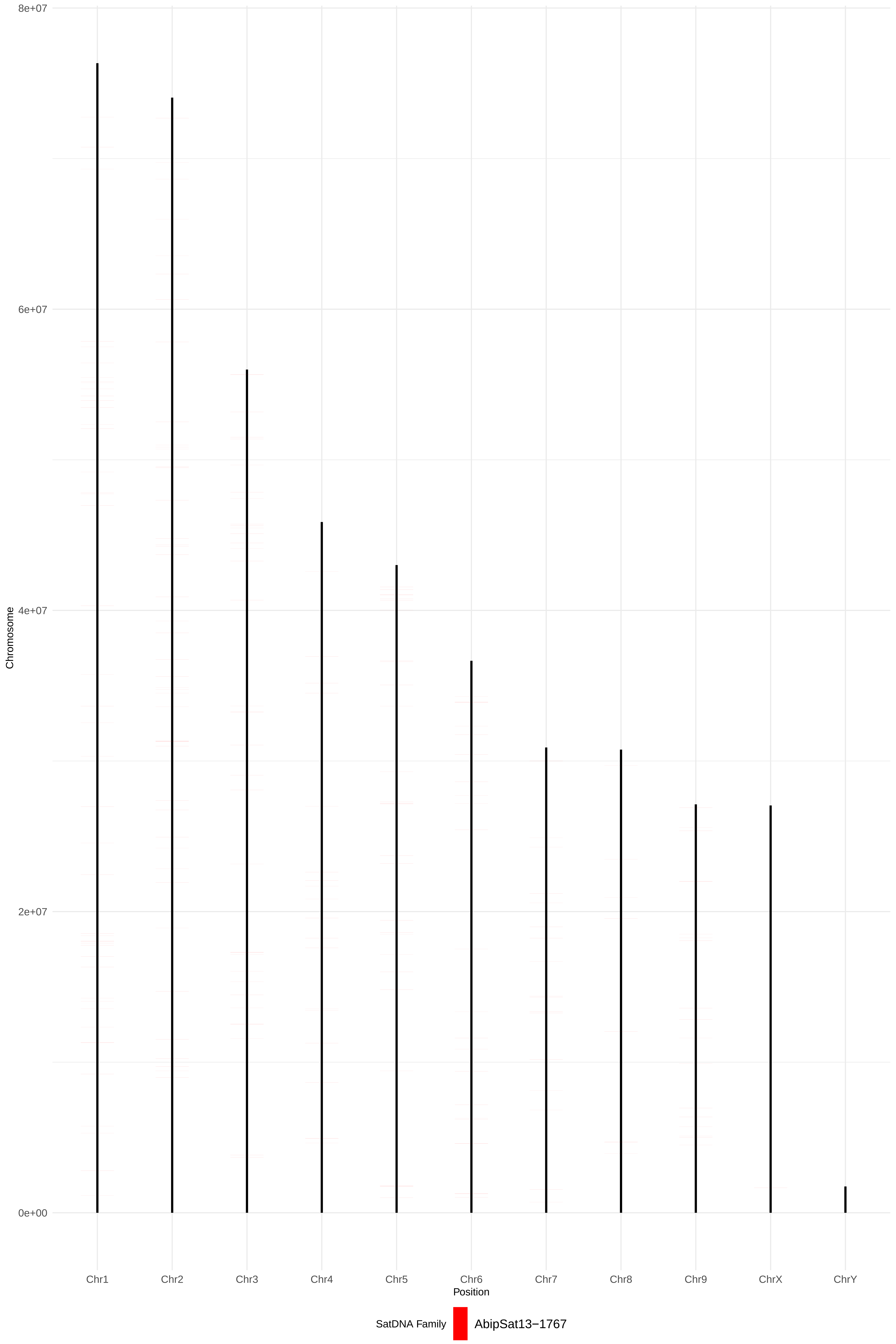

8e+07
6e+07
4e+07
2e+07
0e+00
Chromosome
Chr1
Chr2
Chr3
Chr4
Chr5
Chr6
Chr7
Chr8
Chr9
ChrX
ChrY
Position
SatDNA Family
AbipSat13−1767

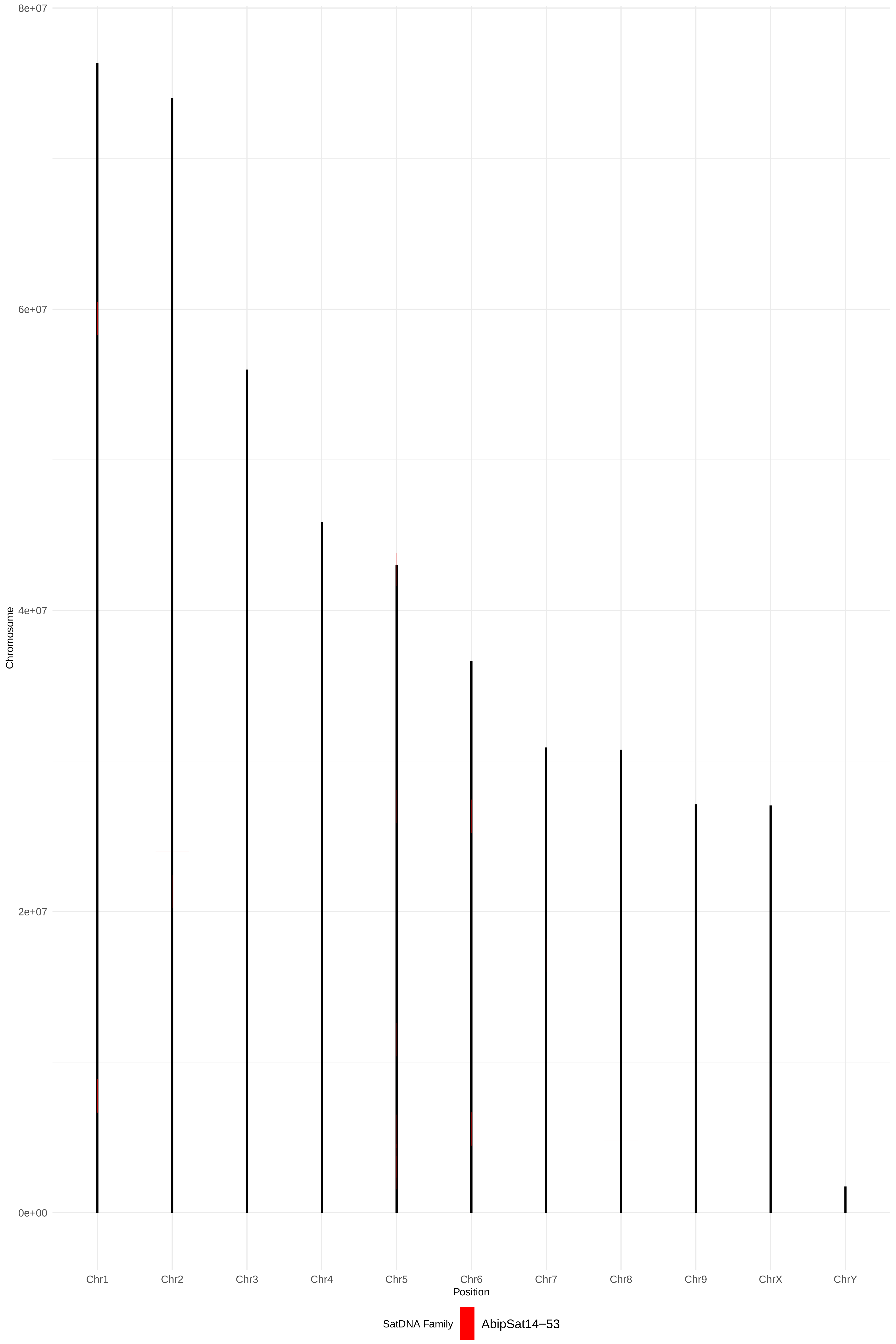

8e+07
6e+07
4e+07
2e+07
0e+00
Chromosome
Chr1
Chr2
Chr3
Chr4
Chr5
Chr6
Chr7
Chr8
Chr9
ChrX
ChrY
Position
SatDNA Family
AbipSat14−53

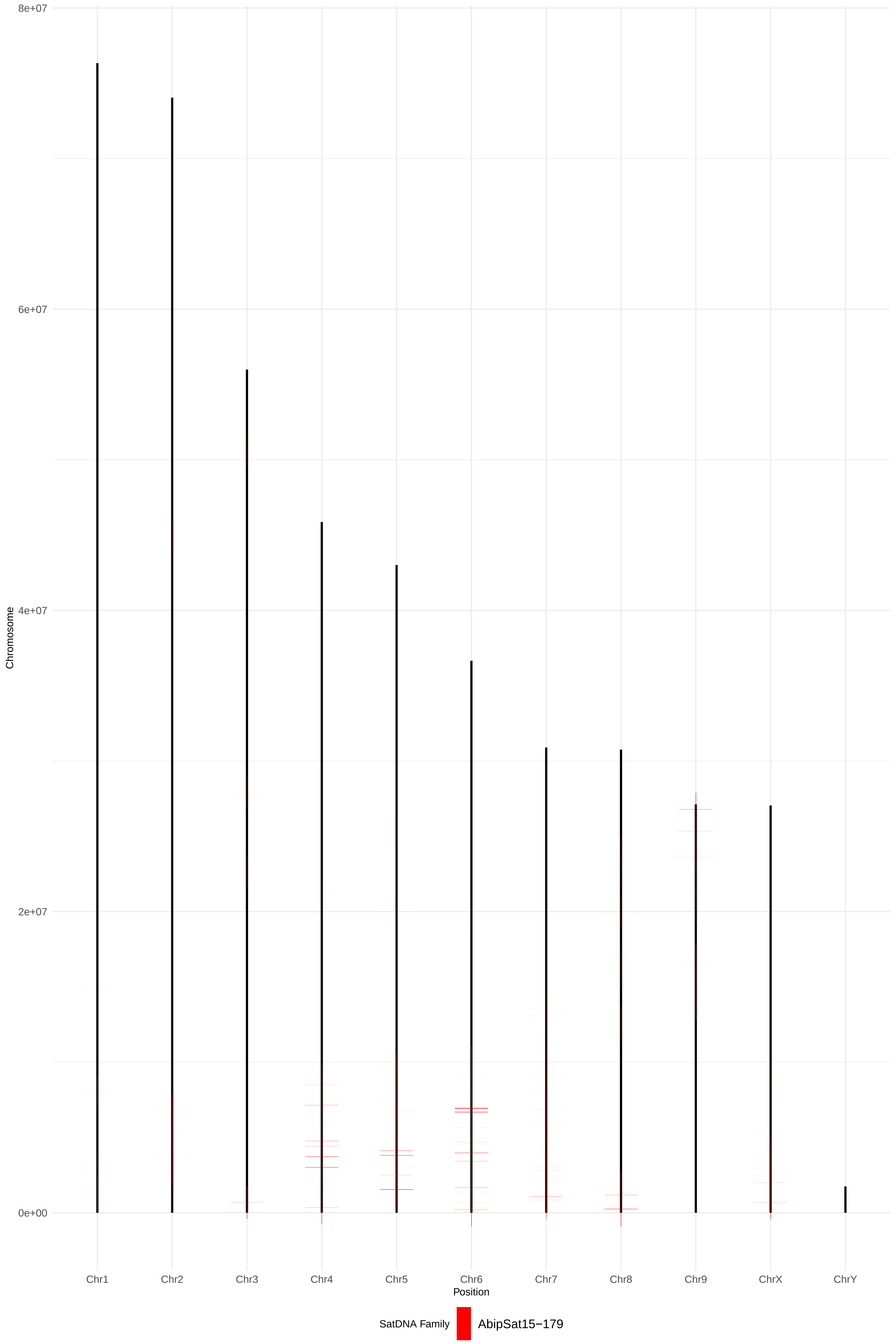

8e+07
6e+07
4e+07
2e+07
0e+00
Chromosome
Chr1
Chr2
Chr3
Chr4
Chr5
Chr6
Chr7
Chr8
Chr9
ChrX
ChrY
Position
SatDNA Family
AbipSat15−179

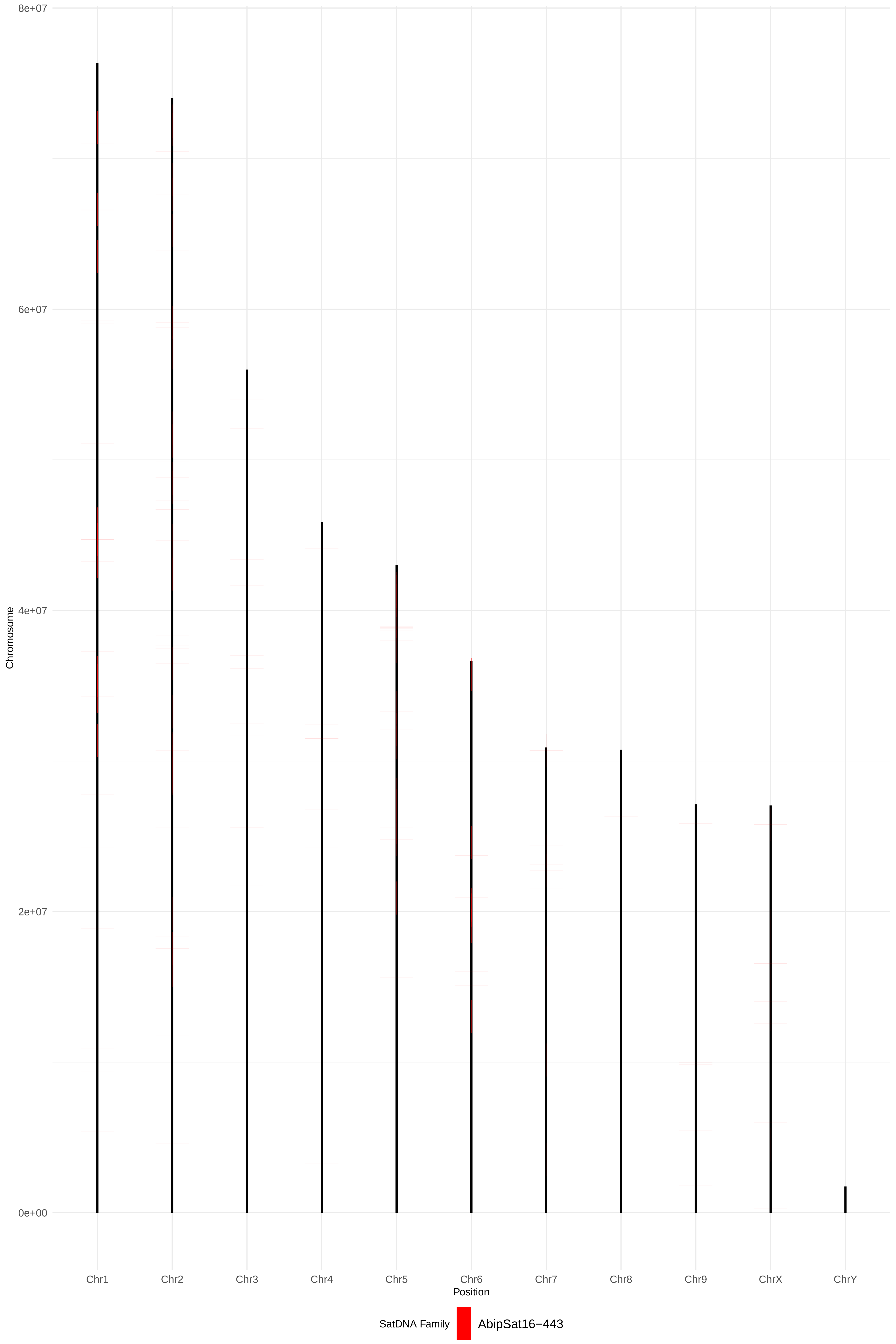

8e+07
6e+07
4e+07
2e+07
0e+00
Chromosome
Chr1
Chr2
Chr3
Chr4
Chr5
Chr6
Chr7
Chr8
Chr9
ChrX
ChrY
Position
SatDNA Family
AbipSat16−443

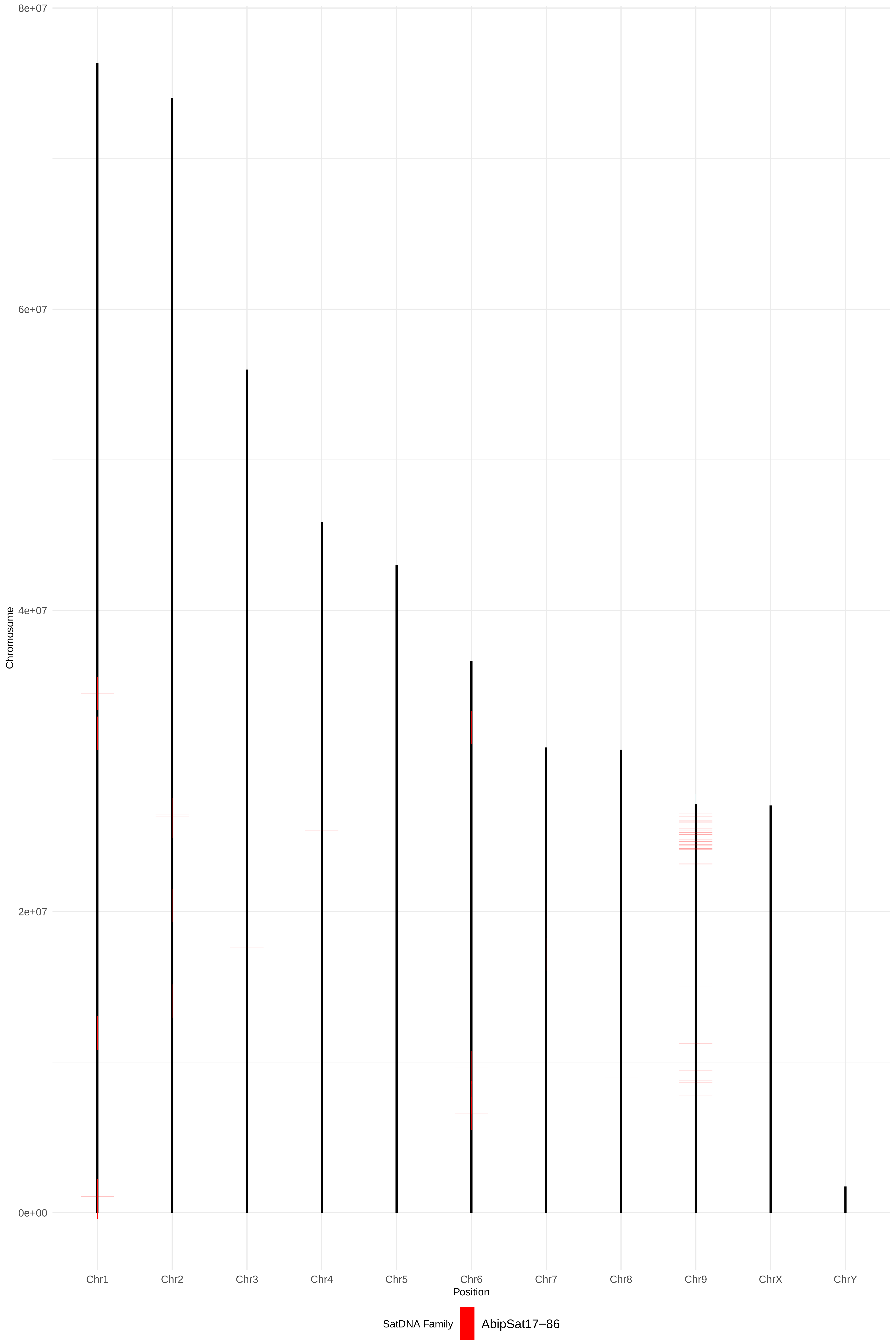

8e+07
6e+07
4e+07
2e+07
0e+00
Chromosome
Chr1
Chr2
Chr3
Chr4
Chr5
Chr6
Chr7
Chr8
Chr9
ChrX
ChrY
Position
SatDNA Family
AbipSat17−86

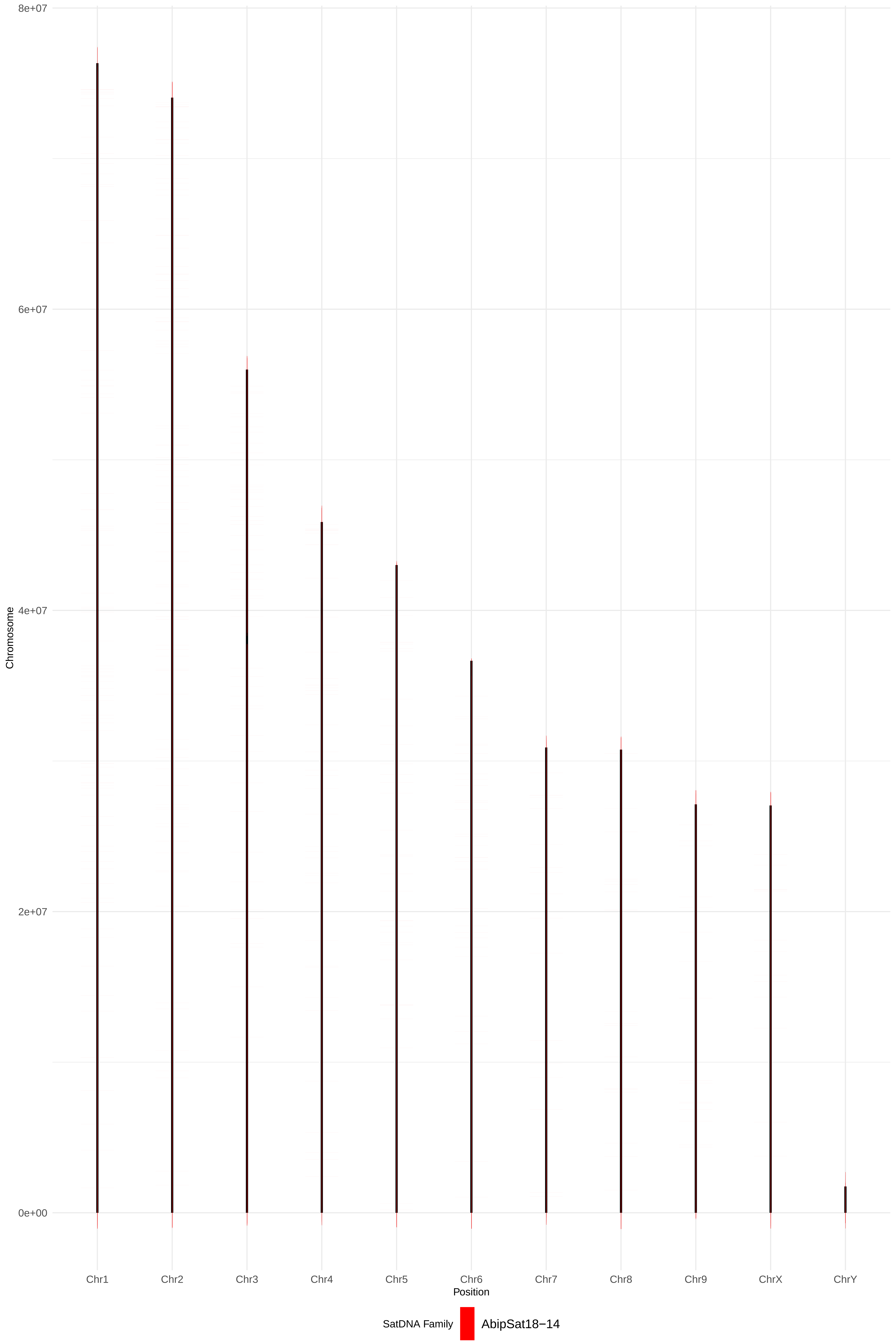

8e+07
6e+07
4e+07
2e+07
0e+00
Chromosome
Chr1
Chr2
Chr3
Chr4
Chr5
Chr6
Chr7
Chr8
Chr9
ChrX
ChrY
Position
SatDNA Family
AbipSat18−14

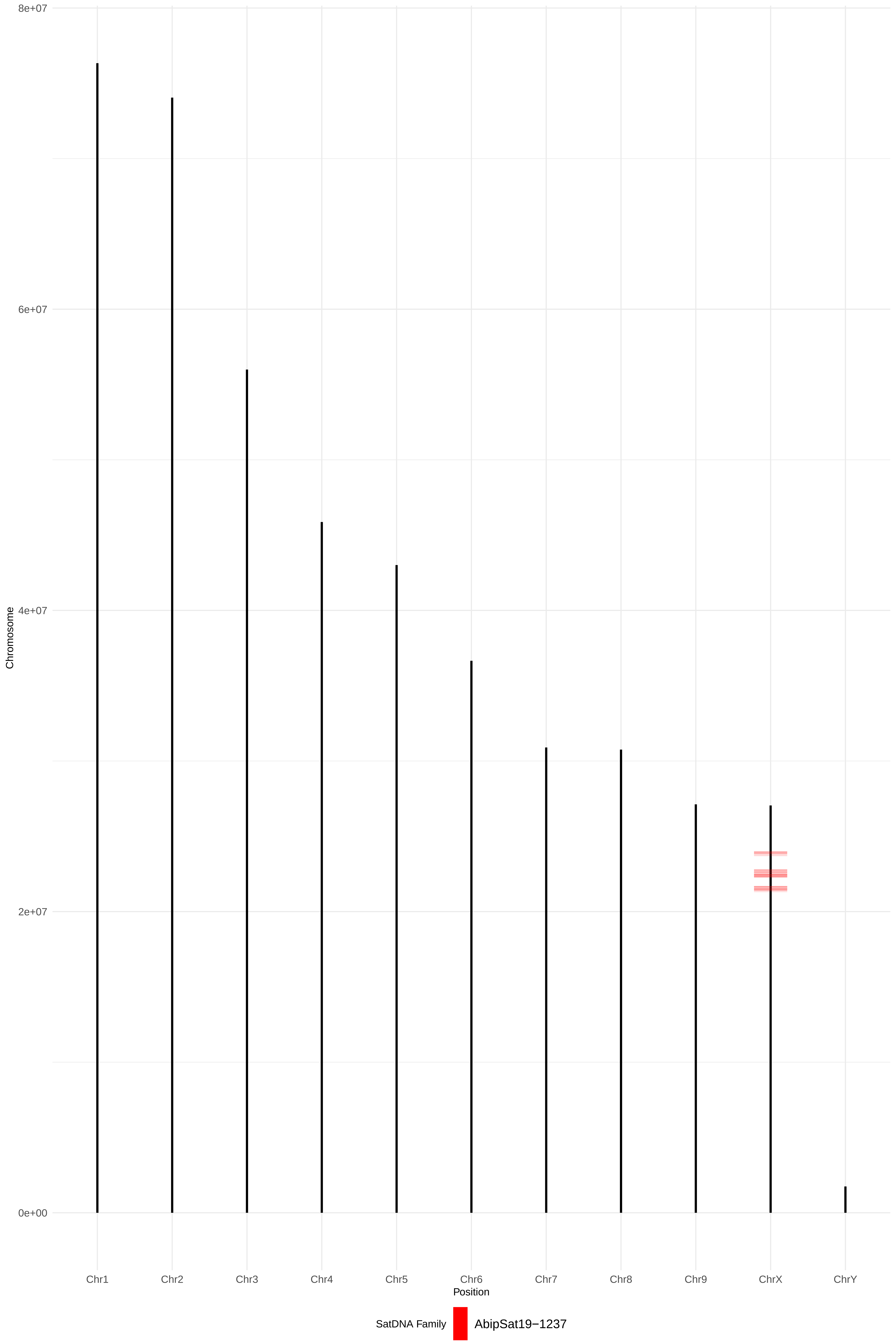

8e+07
6e+07
4e+07
2e+07
0e+00
Chromosome
Chr1
Chr2
Chr3
Chr4
Chr5
Chr6
Chr7
Chr8
Chr9
ChrX
ChrY
Position
SatDNA Family
AbipSat19−1237

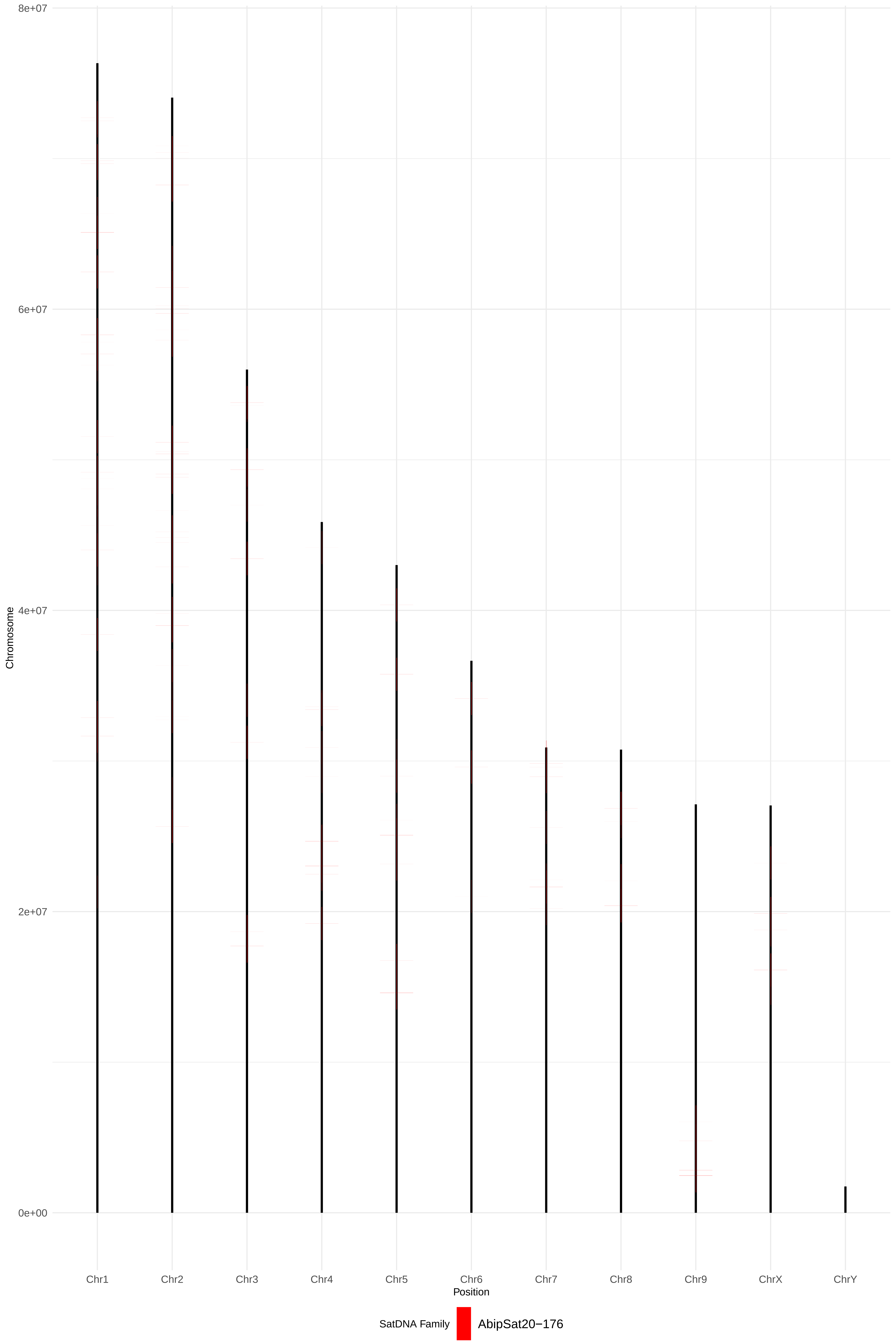

8e+07
6e+07
4e+07
2e+07
0e+00
Chromosome
Chr1
Chr2
Chr3
Chr4
Chr5
Chr6
Chr7
Chr8
Chr9
ChrX
ChrY
Position
SatDNA Family
AbipSat20−176

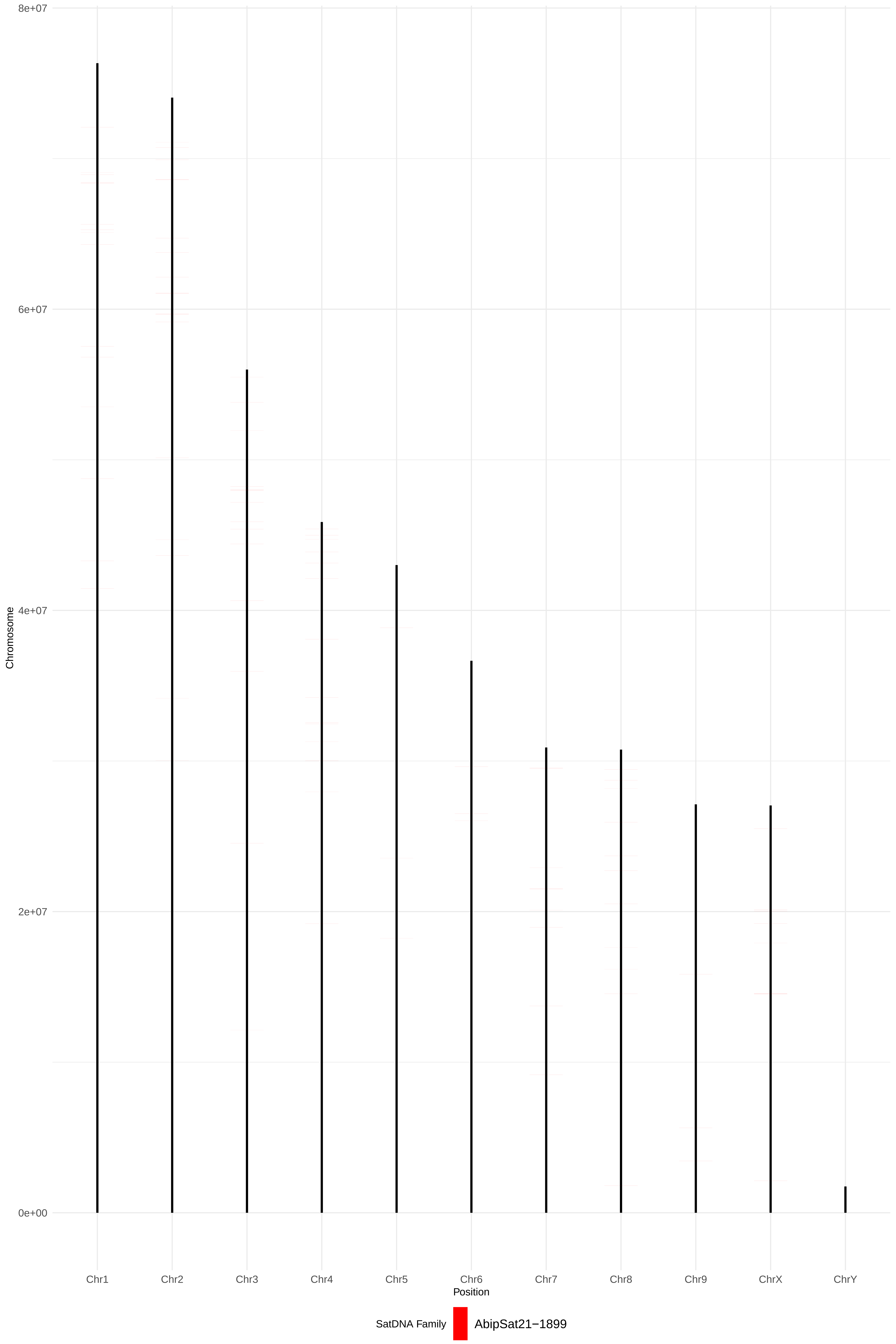

8e+07
6e+07
4e+07
2e+07
0e+00
Chromosome
Chr1
Chr2
Chr3
Chr4
Chr5
Chr6
Chr7
Chr8
Chr9
ChrX
ChrY
Position
SatDNA Family
AbipSat21−1899

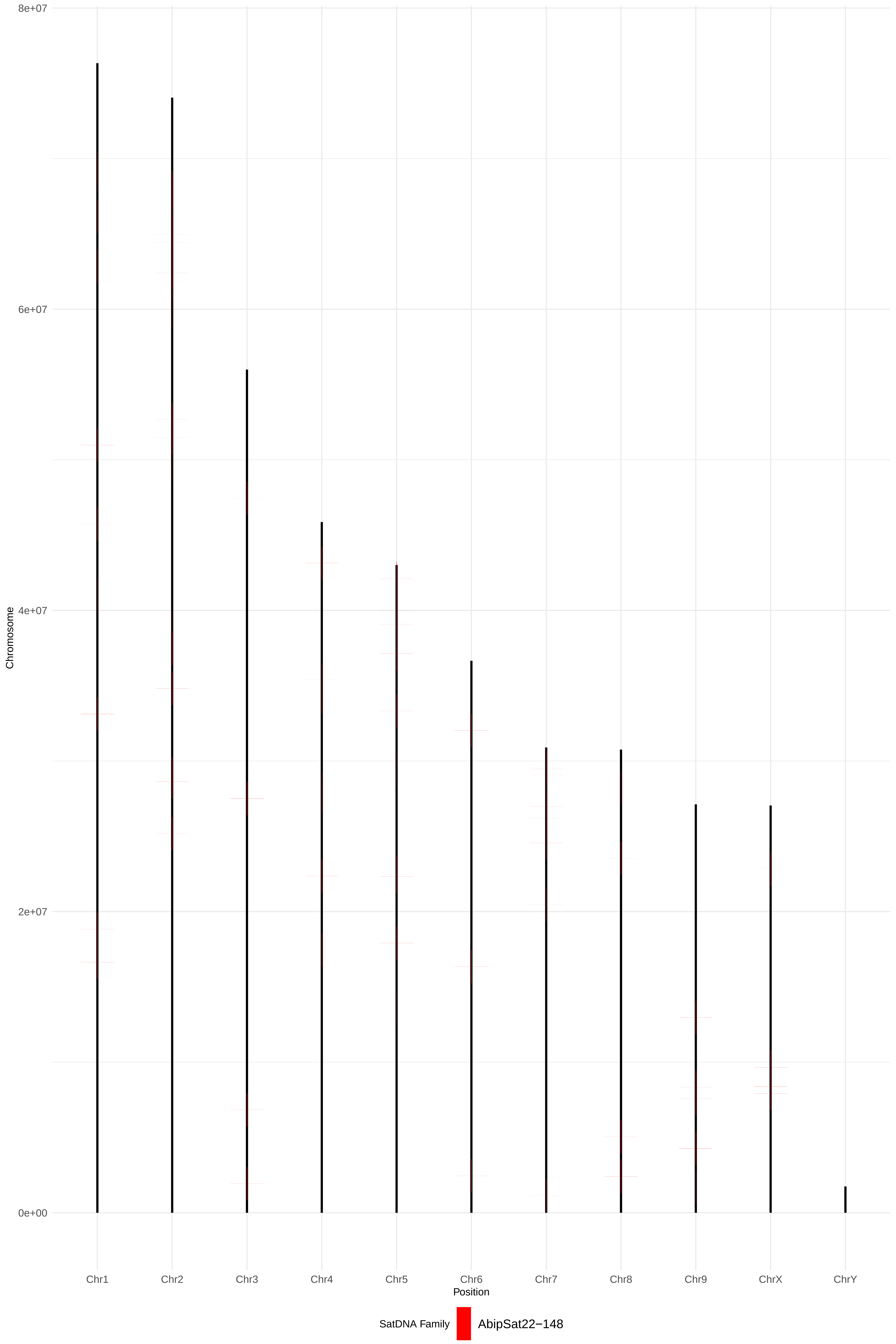
8e+07
6e+07
4e+07
2e+07
0e+00
Chromosome
Chr1
Chr2
Chr3
Chr4
Chr5
Chr6
Chr7
Chr8
Chr9
ChrX
ChrY
Position
SatDNA Family
AbipSat22−148

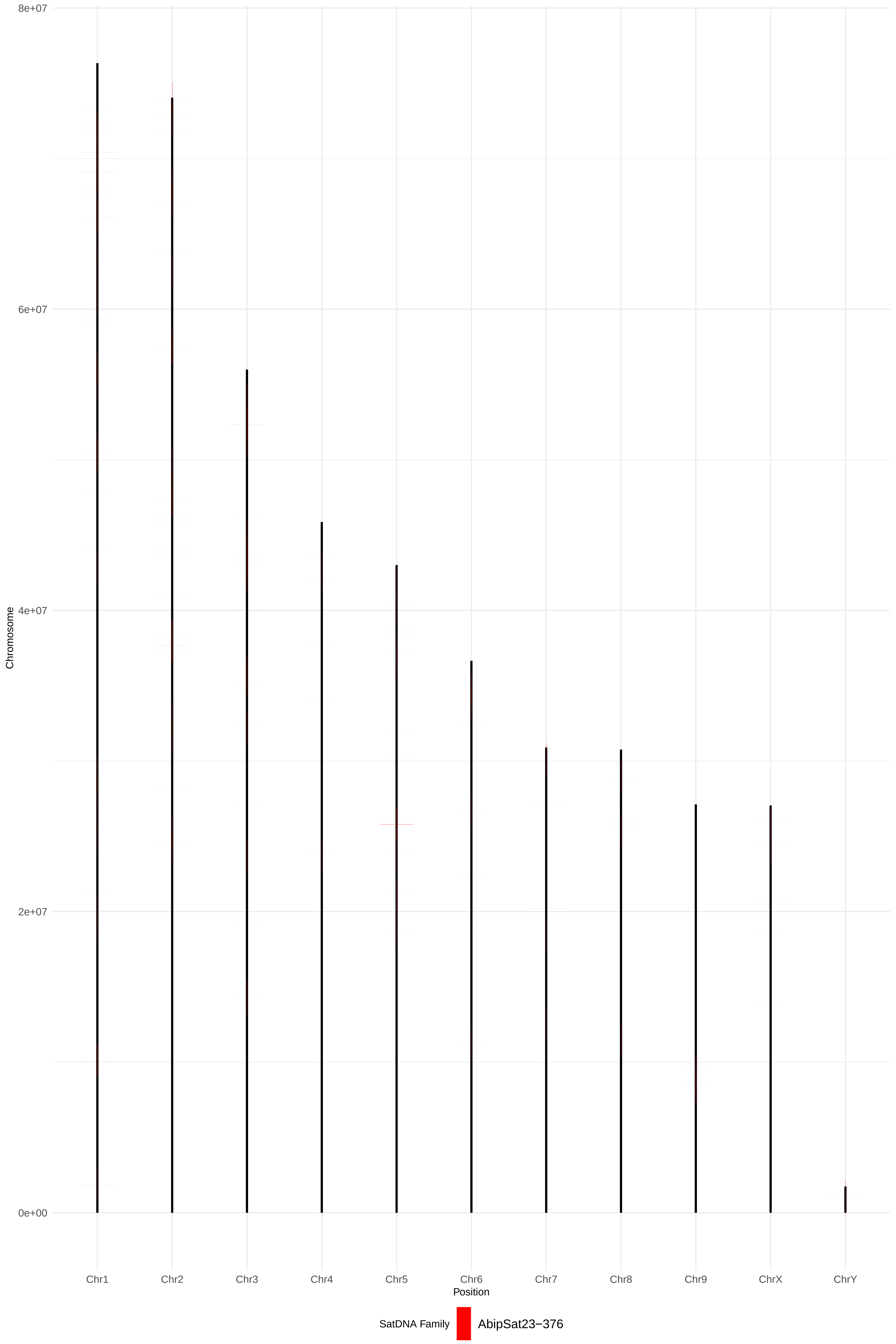
8e+07
6e+07
4e+07
2e+07
0e+00
Chromosome
Chr1
Chr2
Chr3
Chr4
Chr5
Chr6
Chr7
Chr8
Chr9
ChrX
ChrY
Position
SatDNA Family
AbipSat23−376

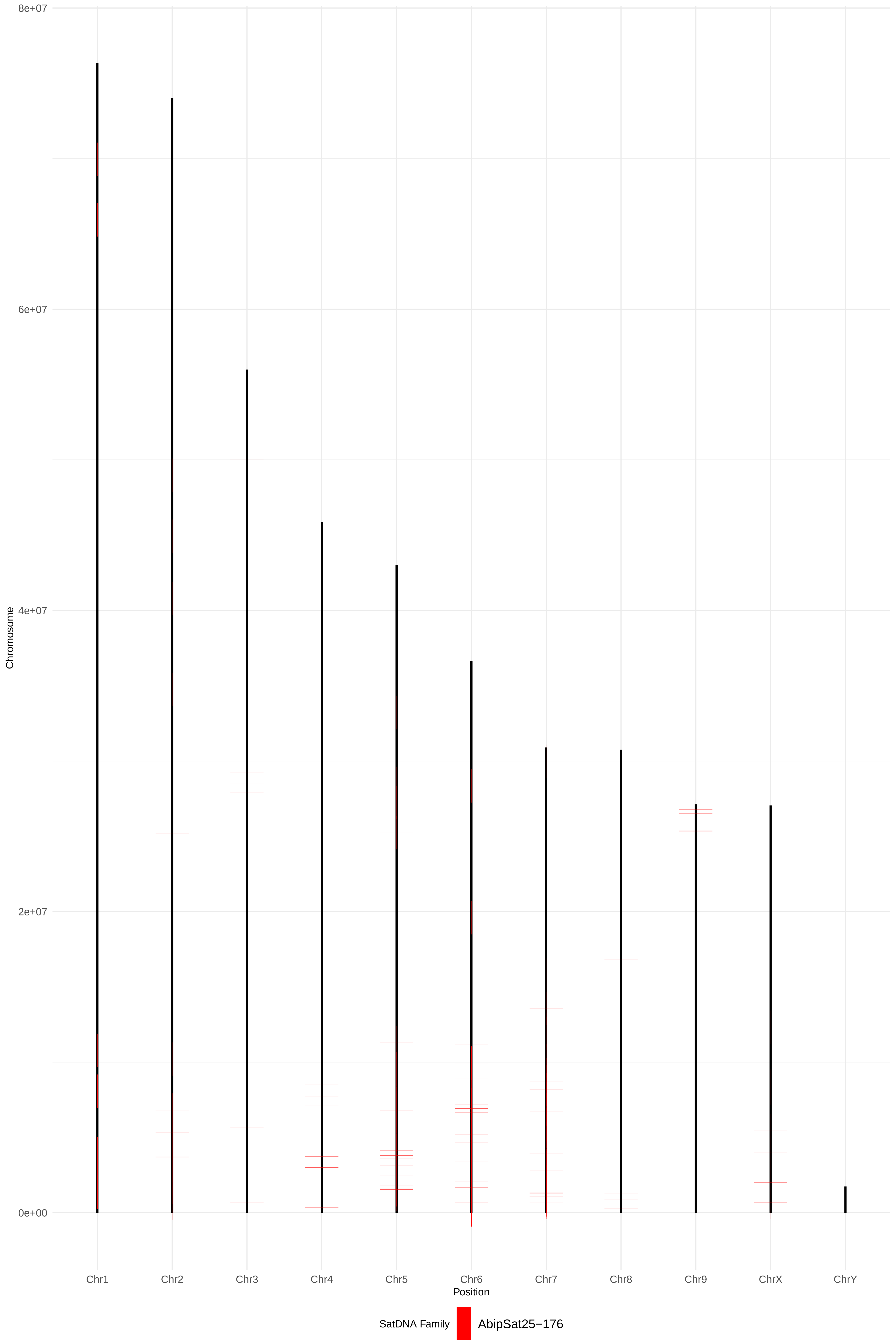

8e+07
6e+07
4e+07
2e+07
0e+00
Chromosome
Chr1
Chr2
Chr3
Chr4
Chr5
Chr6
Chr7
Chr8
Chr9
ChrX
ChrY
Position
SatDNA Family
AbipSat25−176

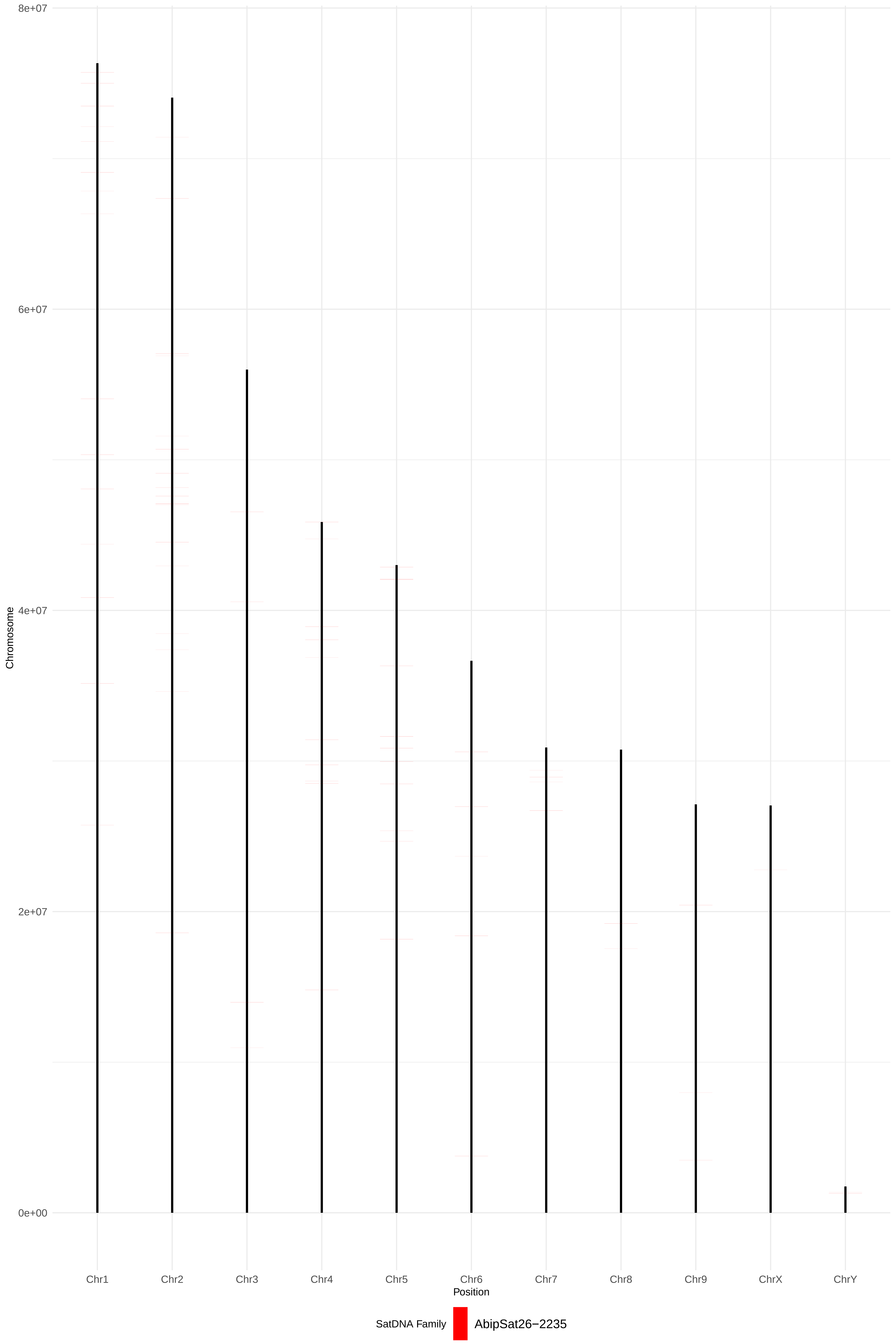

8e+07
6e+07
4e+07
2e+07
0e+00
Chromosome
Chr1
Chr2
Chr3
Chr4
Chr5
Chr6
Chr7
Chr8
Chr9
ChrX
ChrY
Position
SatDNA Family
AbipSat26−2235

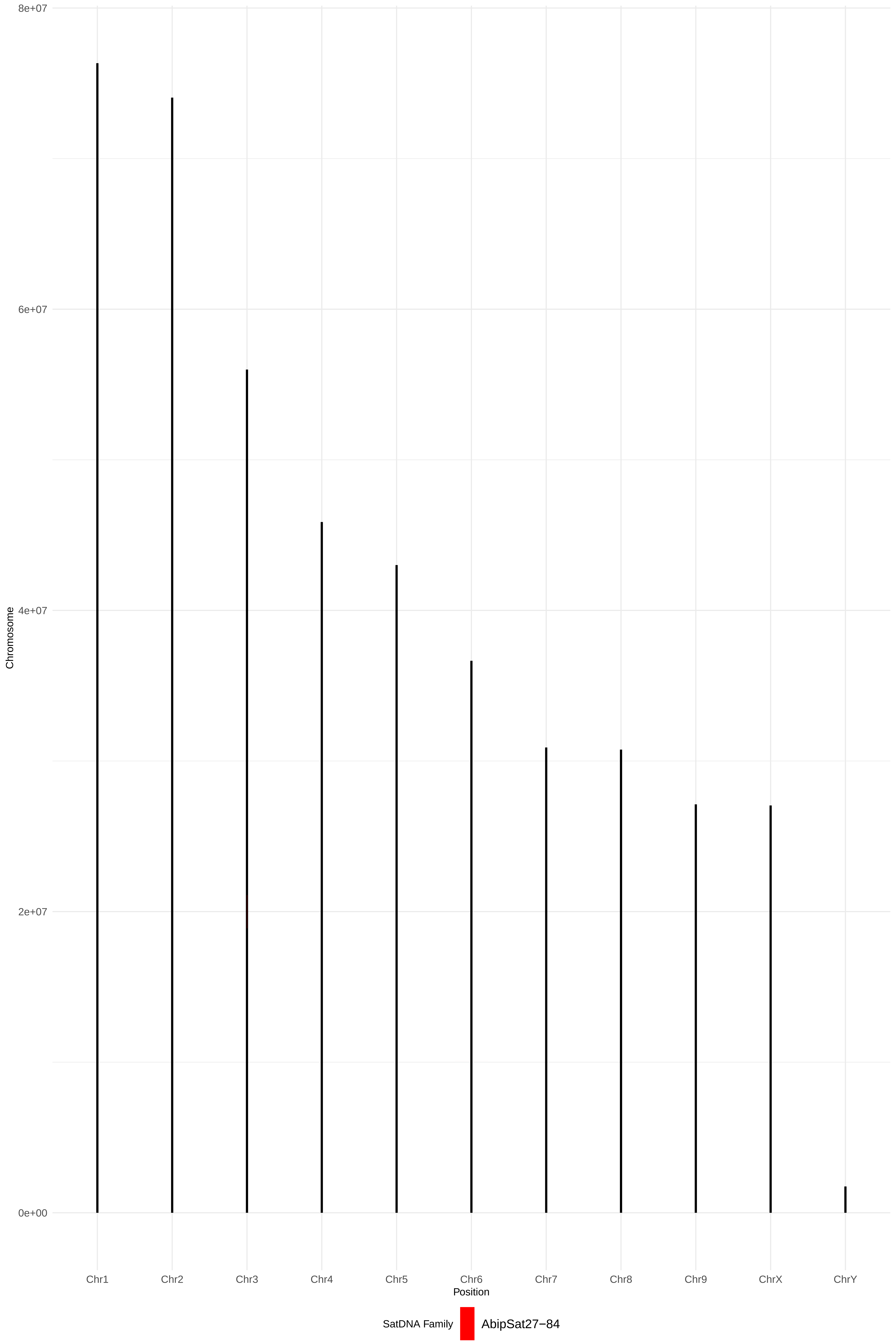
8e+07
6e+07
4e+07
2e+07
0e+00
Chromosome
Chr1
Chr2
Chr3
Chr4
Chr5
Chr6
Chr7
Chr8
Chr9
ChrX
ChrY
Position
SatDNA Family
AbipSat27−84

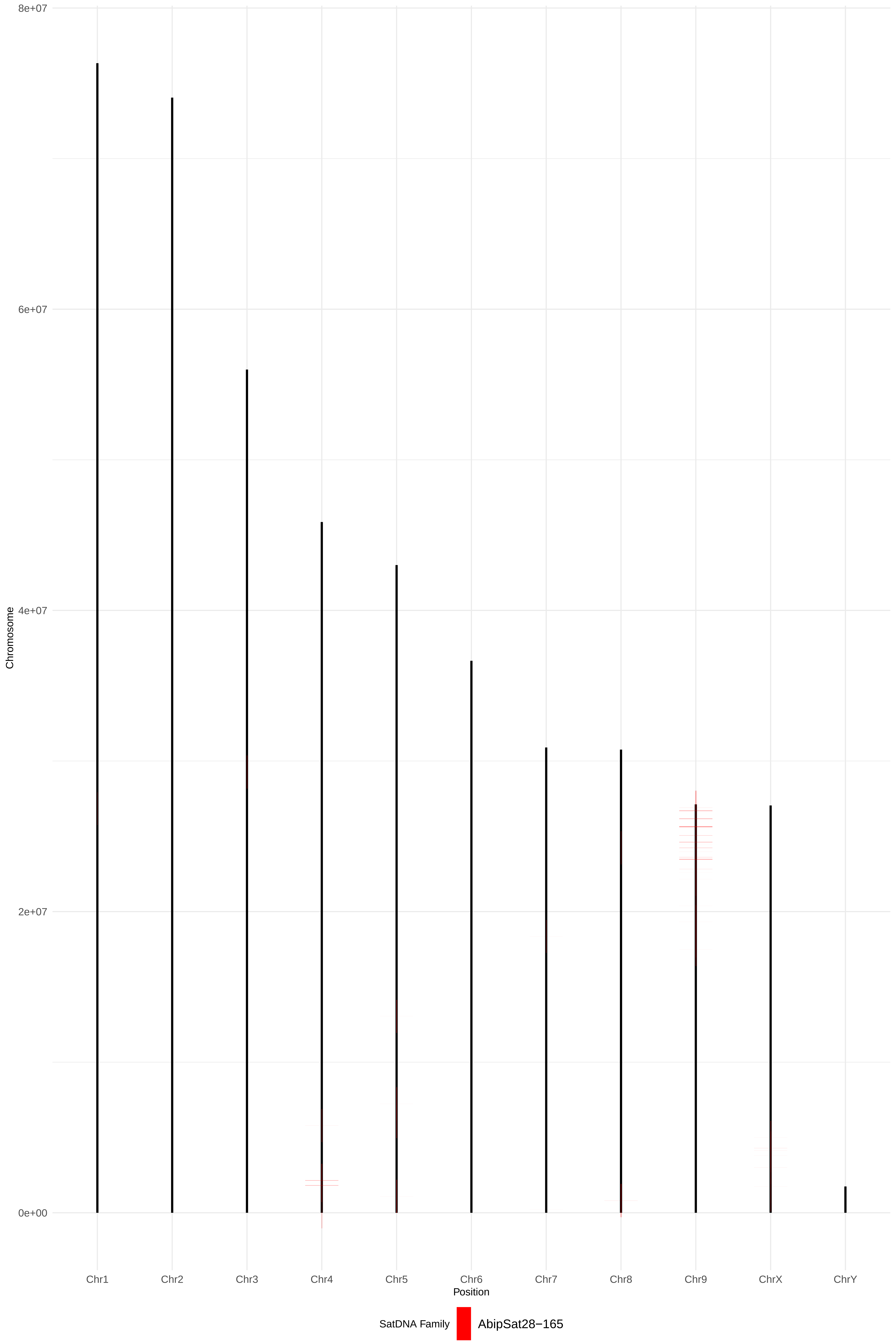
8e+07
6e+07
4e+07
2e+07
0e+00
Chromosome
Chr1
Chr2
Chr3
Chr4
Chr5
Chr6
Chr7
Chr8
Chr9
ChrX
ChrY
Position
SatDNA Family
AbipSat28−165

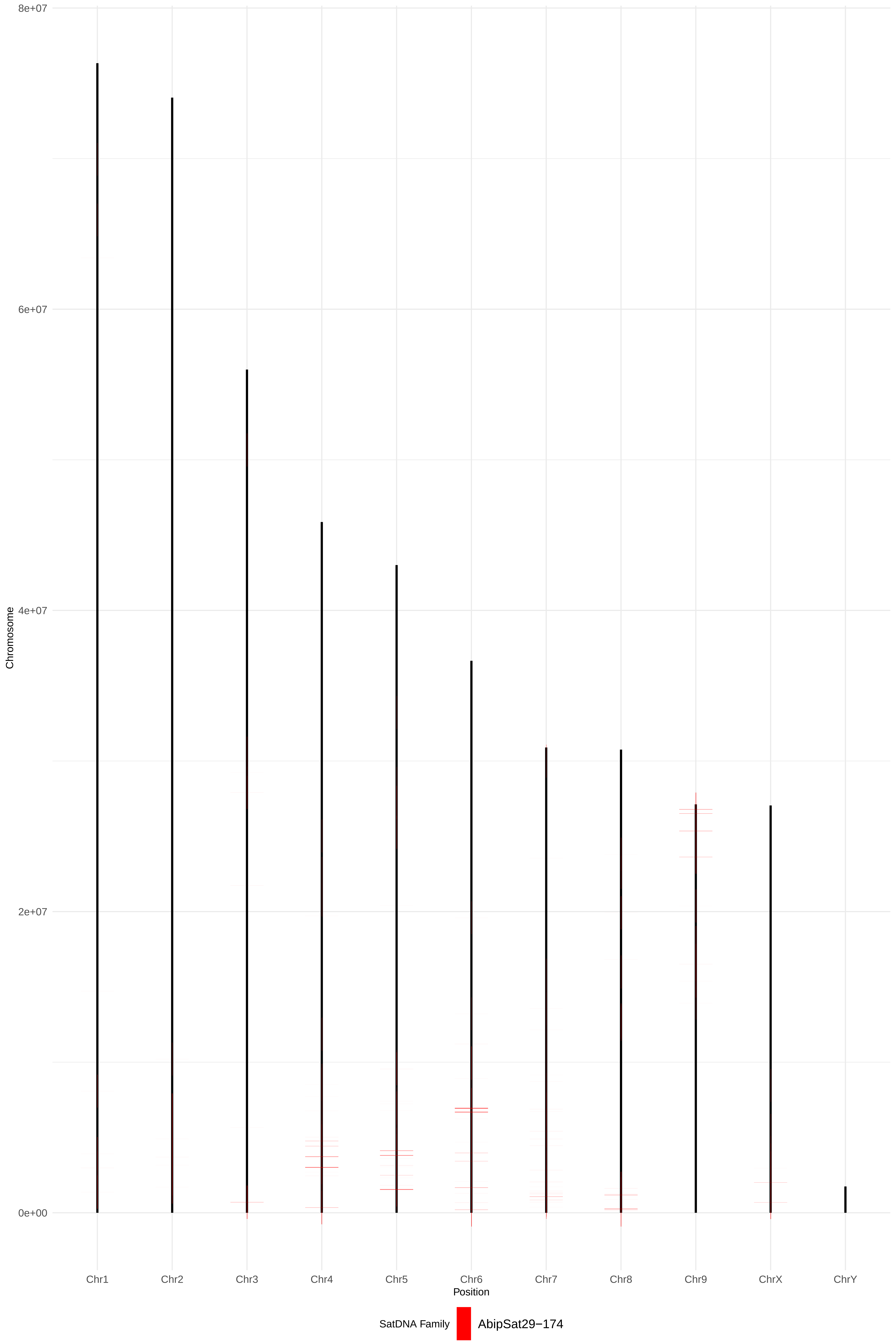

8e+07
6e+07
4e+07
2e+07
0e+00
Chromosome
Chr1
Chr2
Chr3
Chr4
Chr5
Chr6
Chr7
Chr8
Chr9
ChrX
ChrY
Position
SatDNA Family
AbipSat29−174

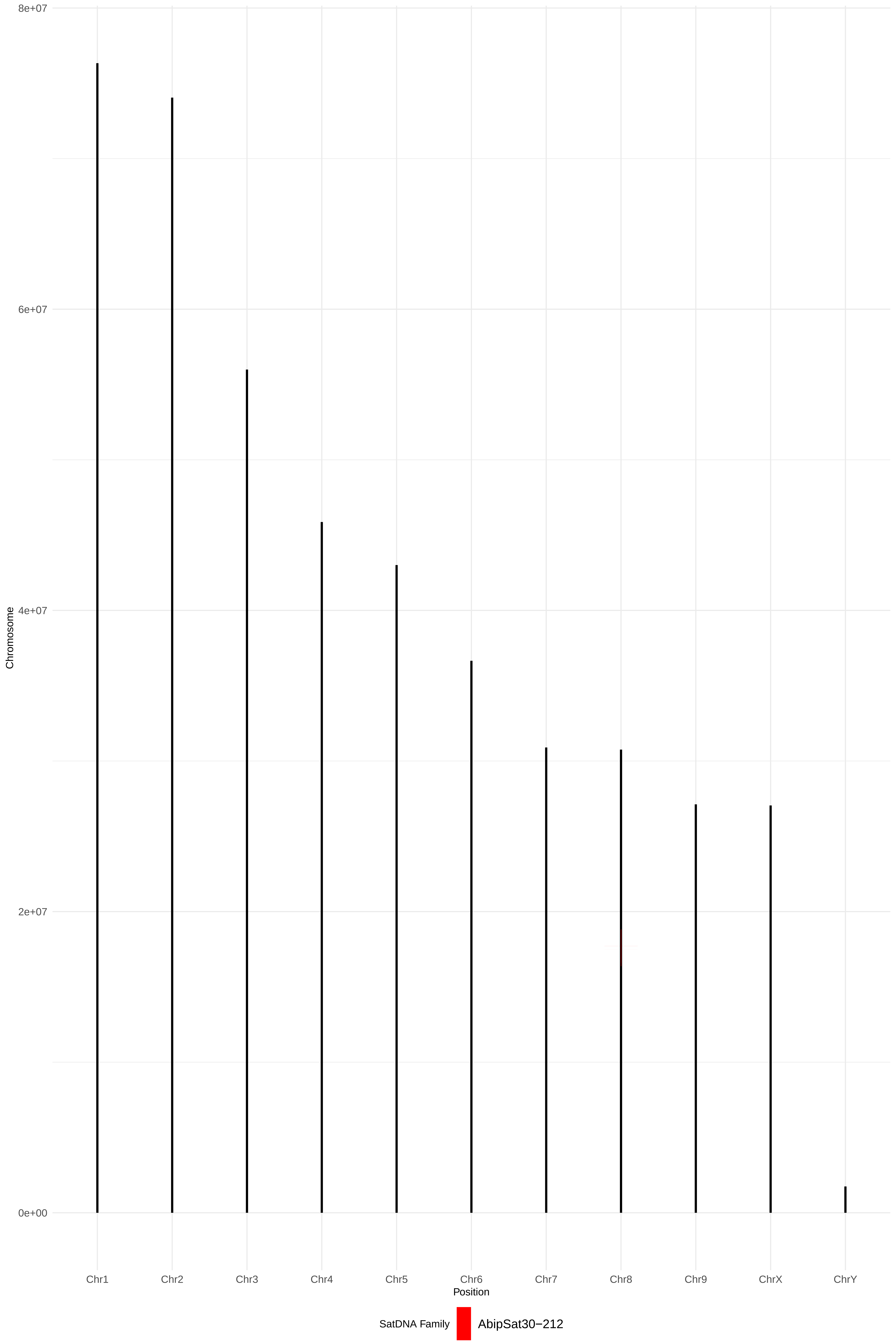

8e+07
6e+07
4e+07
2e+07
0e+00
Chromosome
Chr1
Chr2
Chr3
Chr4
Chr5
Chr6
Chr7
Chr8
Chr9
ChrX
ChrY
Position
SatDNA Family
AbipSat30−212

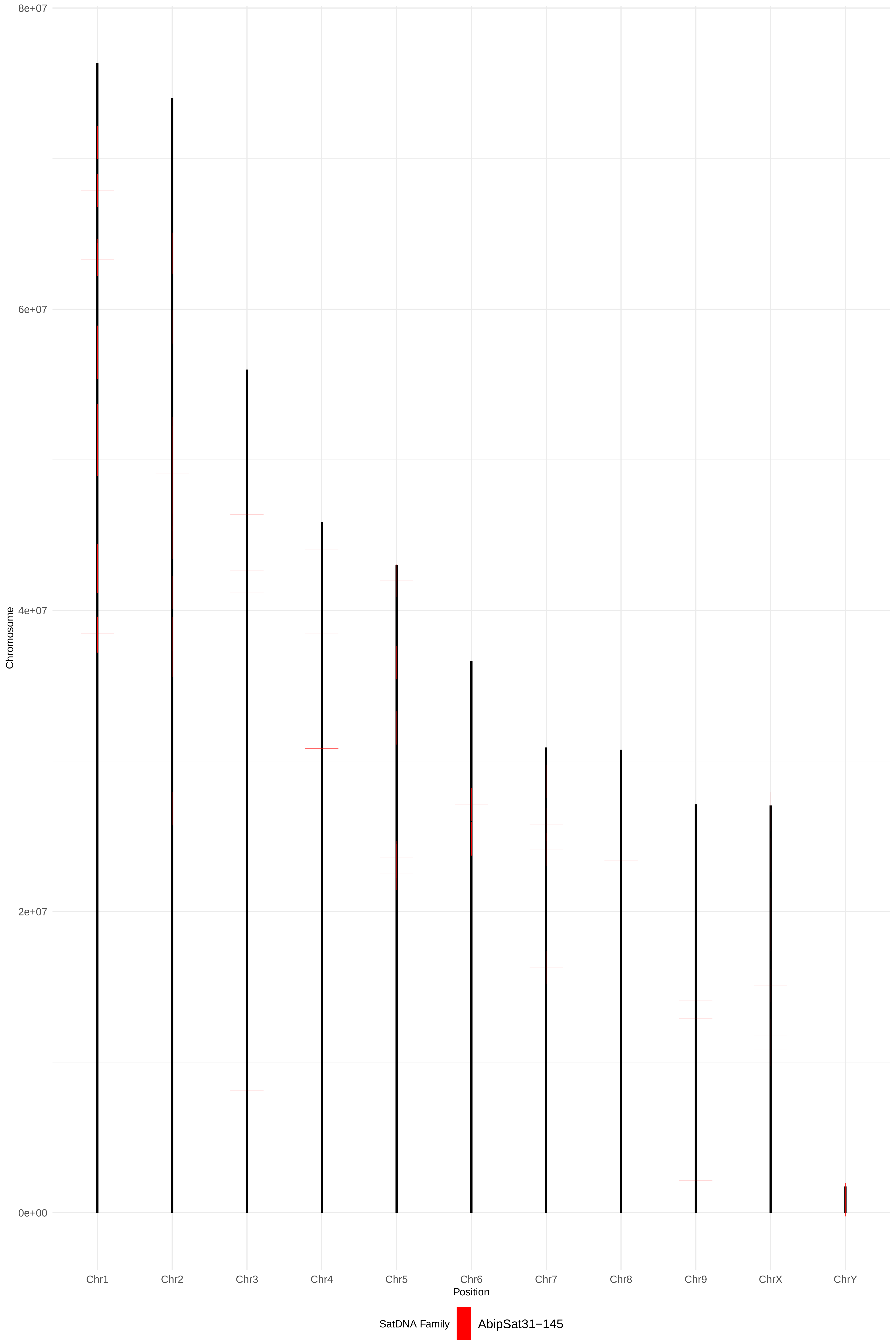

8e+07
6e+07
4e+07
2e+07
0e+00
Chromosome
Chr1
Chr2
Chr3
Chr4
Chr5
Chr6
Chr7
Chr8
Chr9
ChrX
ChrY
Position
SatDNA Family
AbipSat31−145

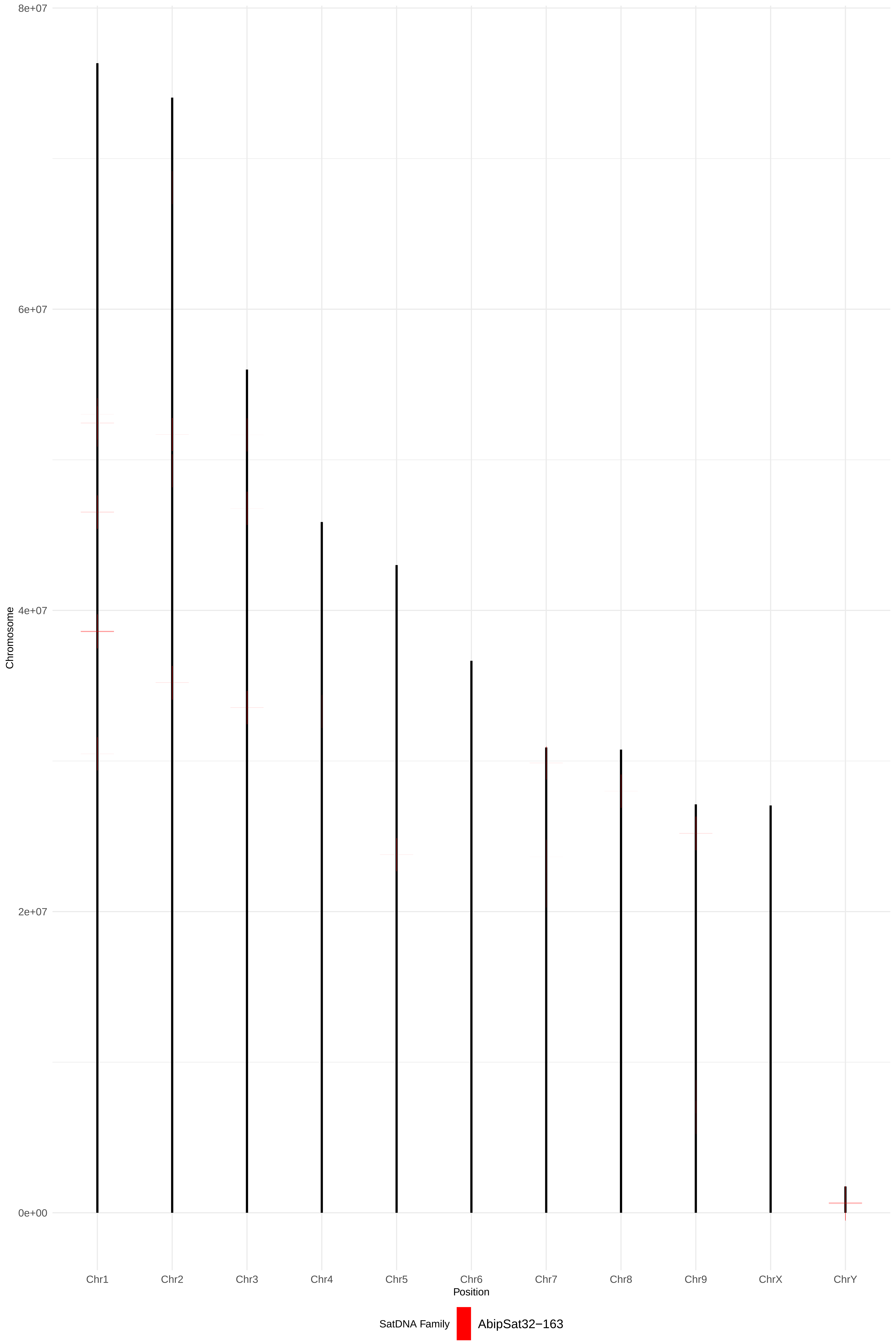

8e+07
6e+07
4e+07
2e+07
0e+00
Chromosome
Chr1
Chr2
Chr3
Chr4
Chr5
Chr6
Chr7
Chr8
Chr9
ChrX
ChrY
Position
SatDNA Family
AbipSat32−163

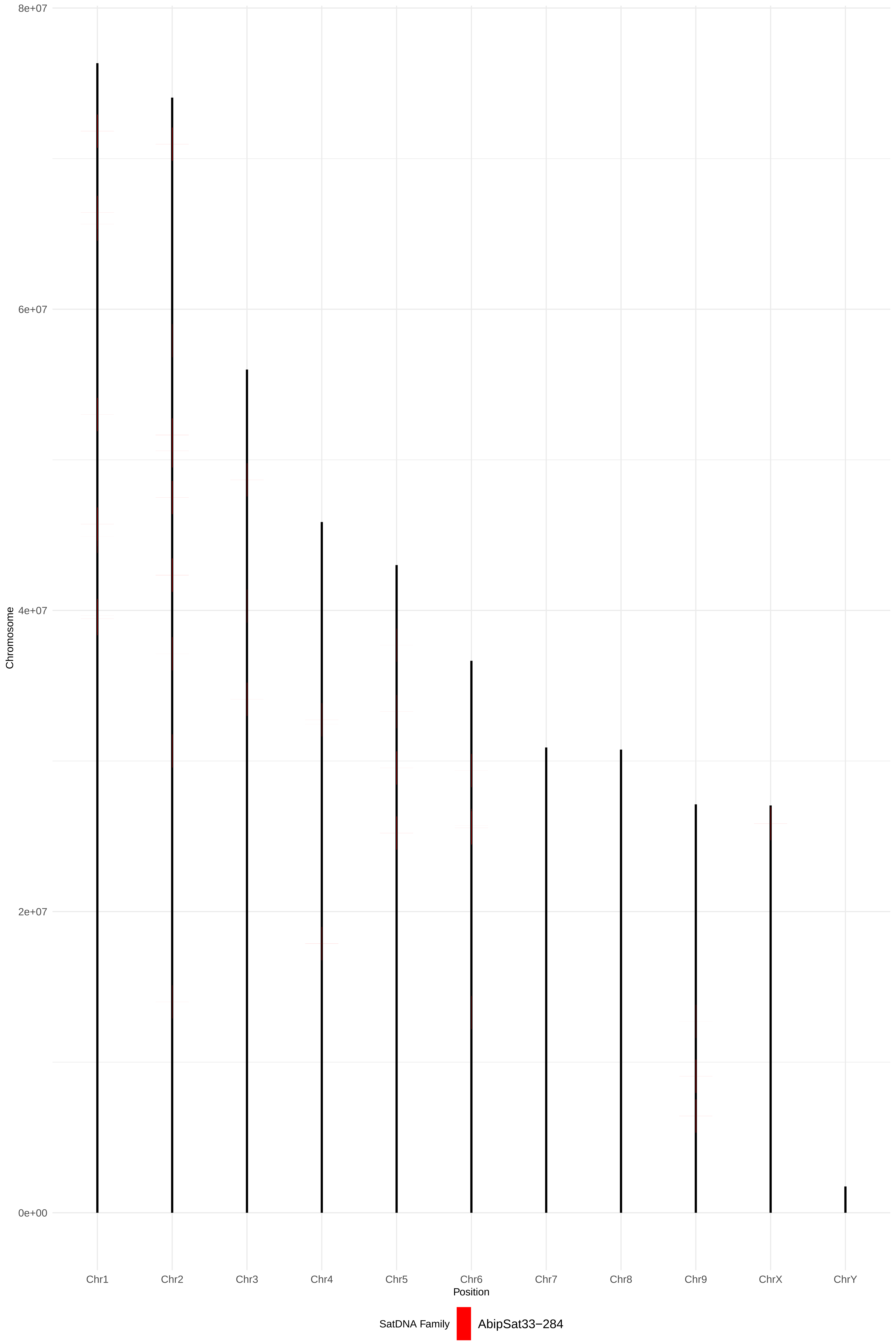

8e+07
6e+07
4e+07
2e+07
0e+00
Chromosome
Chr1
Chr2
Chr3
Chr4
Chr5
Chr6
Chr7
Chr8
Chr9
ChrX
ChrY
Position
SatDNA Family
AbipSat33−284

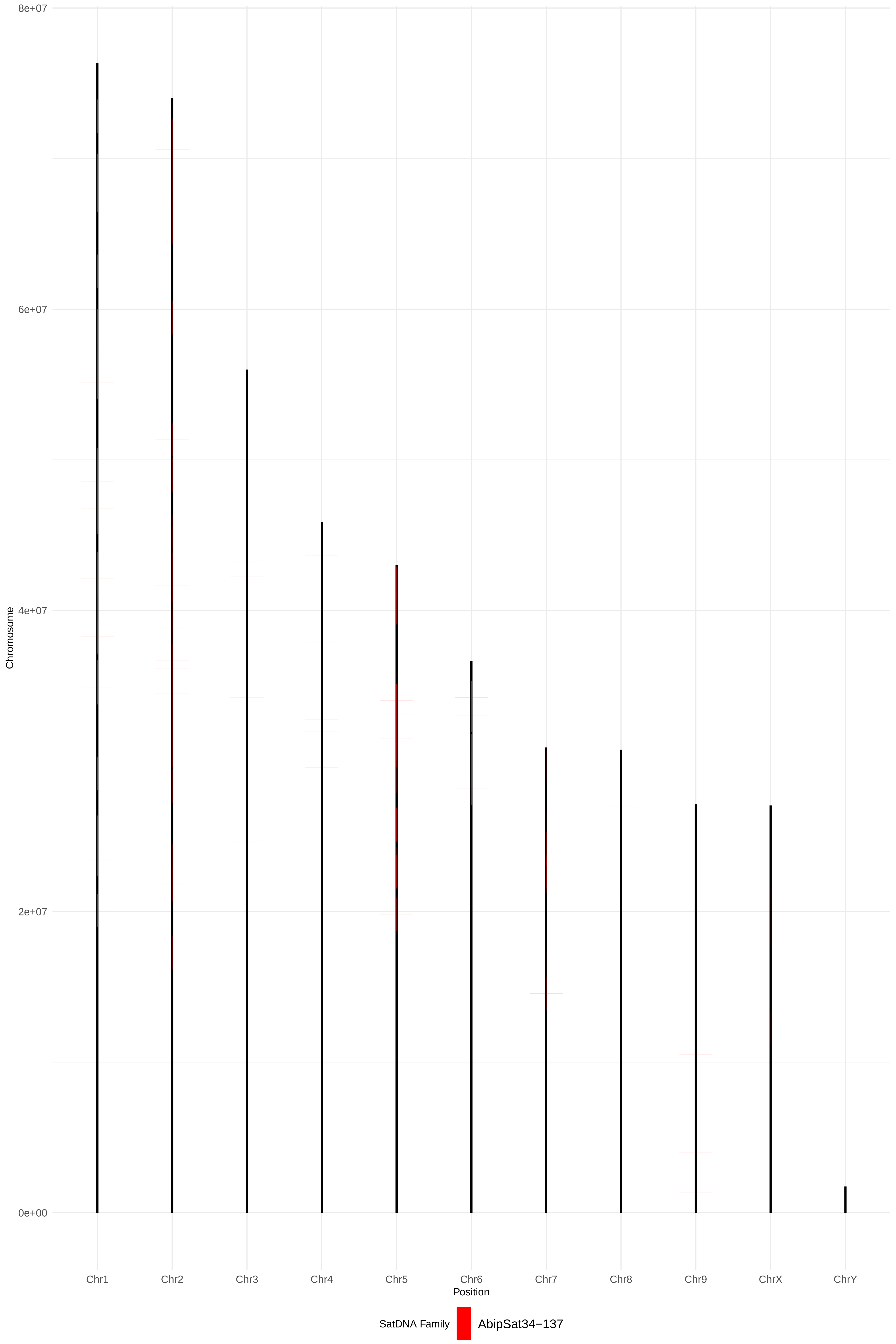

8e+07
6e+07
4e+07
2e+07
0e+00
Chromosome
Chr1
Chr2
Chr3
Chr4
Chr5
Chr6
Chr7
Chr8
Chr9
ChrX
ChrY
Position
SatDNA Family
AbipSat34−137

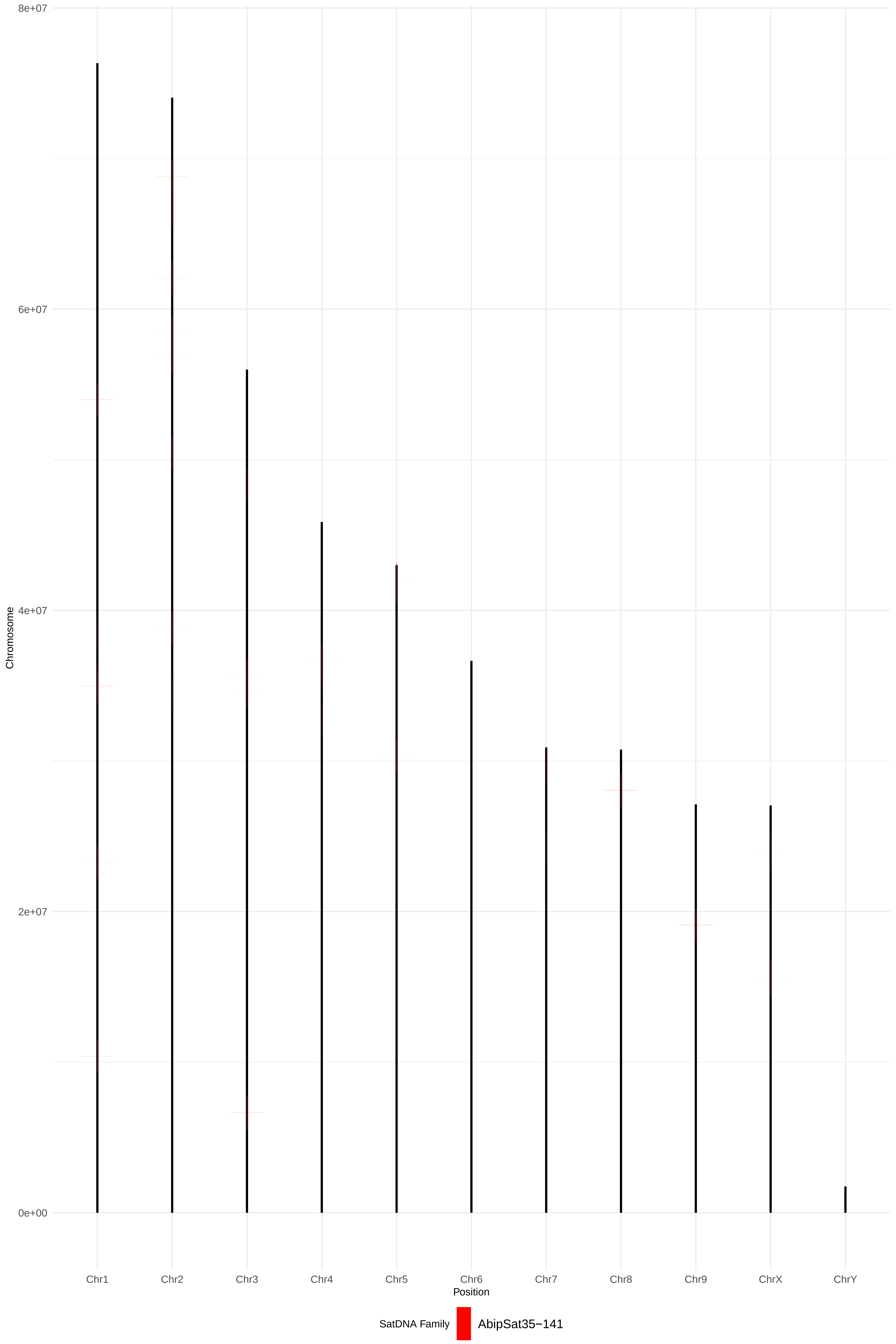

8e+07
6e+07
4e+07
2e+07
0e+00
Chromosome
Chr1
Chr2
Chr3
Chr4
Chr5
Chr6
Chr7
Chr8
Chr9
ChrX
ChrY
Position
SatDNA Family
AbipSat35−141

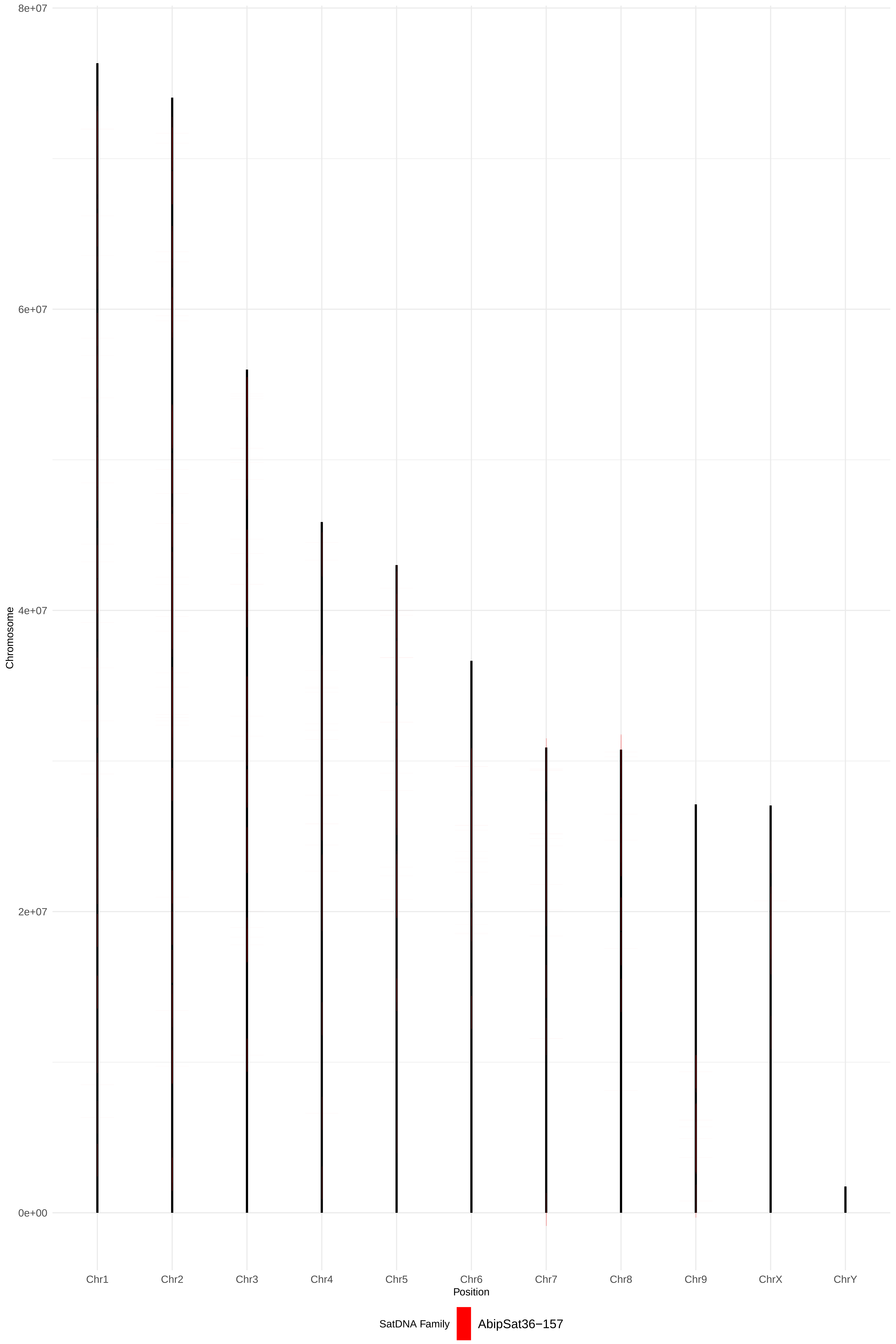

8e+07
6e+07
4e+07
2e+07
0e+00
Chromosome
Chr1
Chr2
Chr3
Chr4
Chr5
Chr6
Chr7
Chr8
Chr9
ChrX
ChrY
Position
SatDNA Family
AbipSat36−157

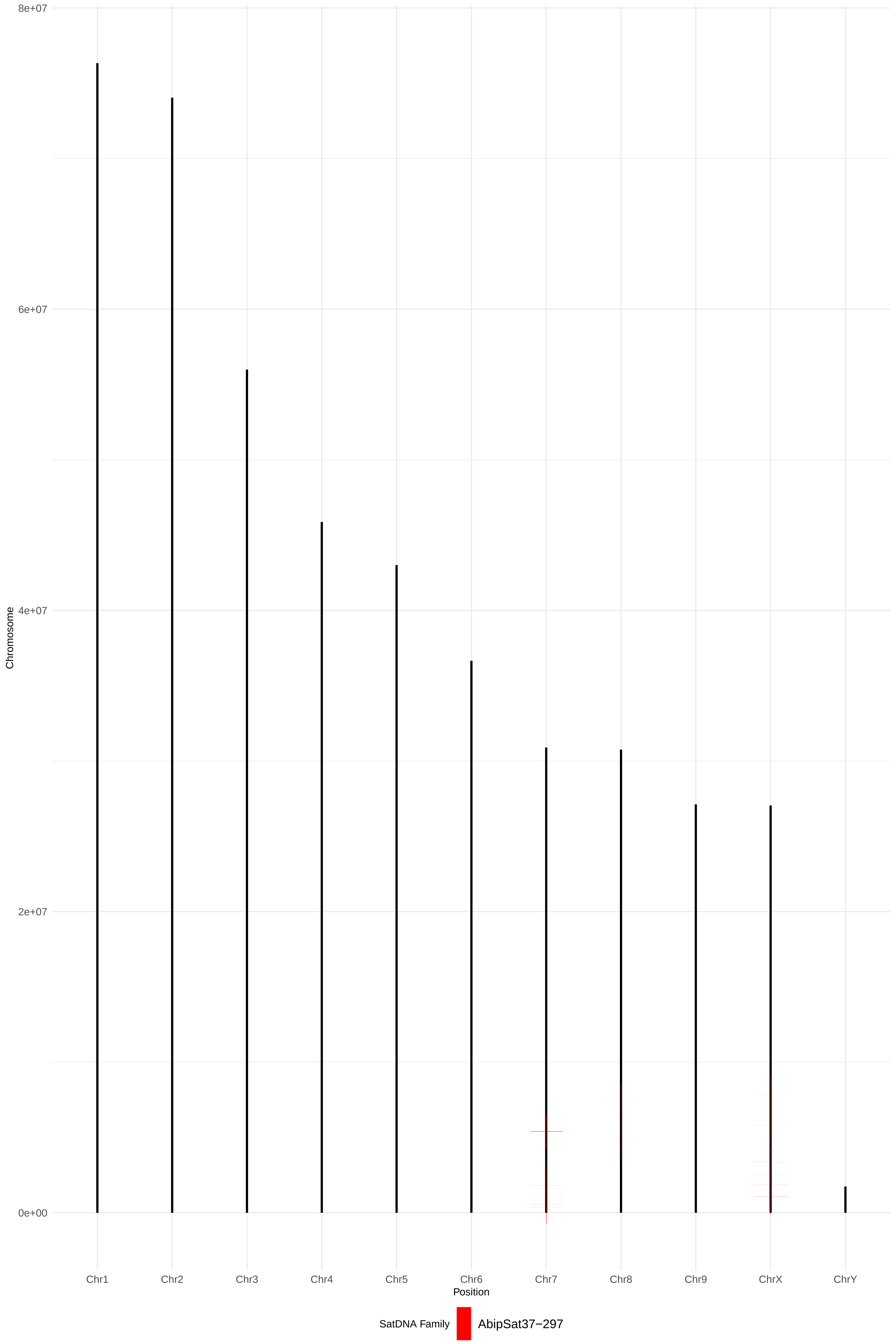

8e+07
6e+07
4e+07
2e+07
0e+00
Chromosome
Chr1
Chr2
Chr3
Chr4
Chr5
Chr6
Chr7
Chr8
Chr9
ChrX
ChrY
Position
SatDNA Family
AbipSat37−297

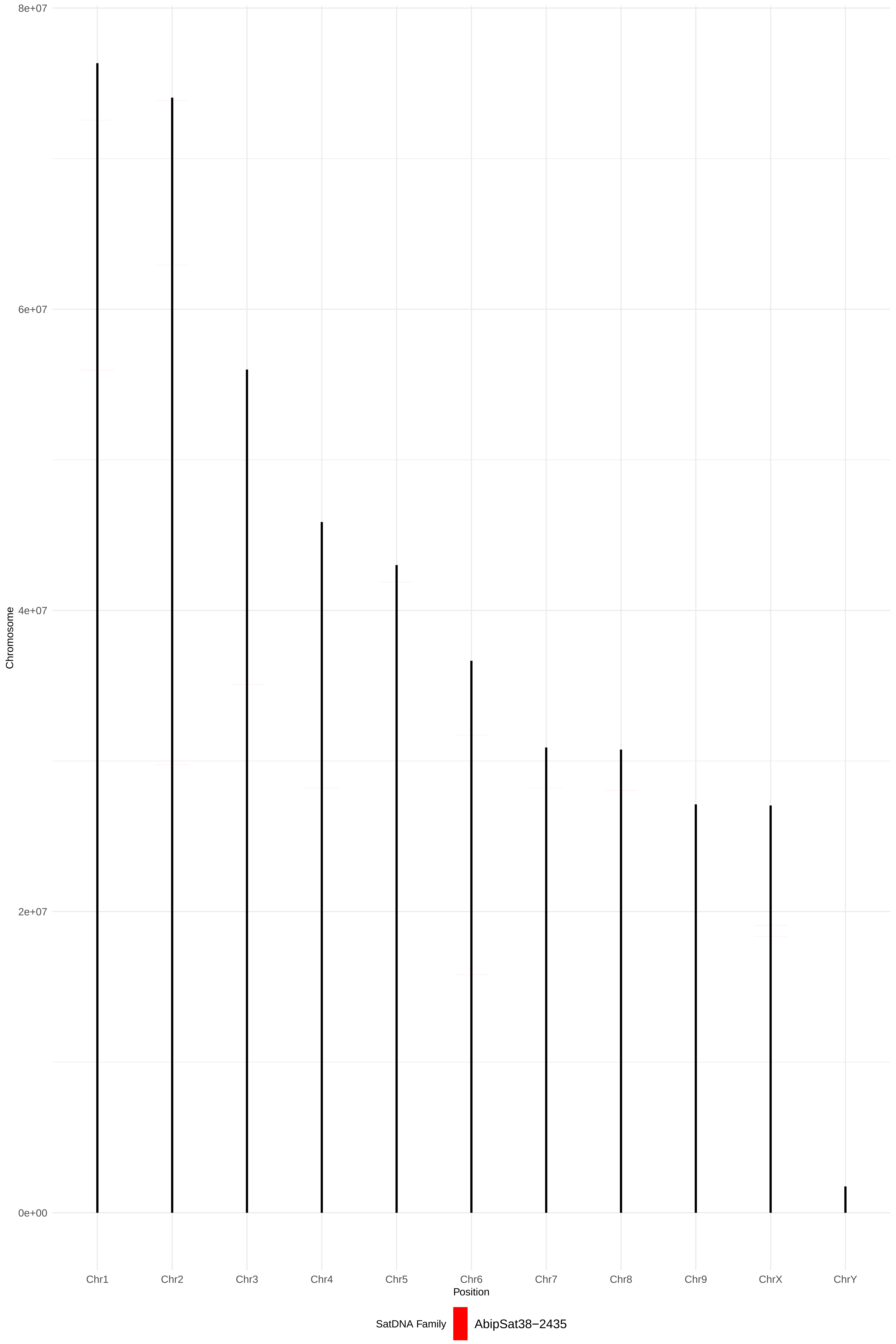

8e+07
6e+07
4e+07
2e+07
0e+00
Chromosome
Chr1
Chr2
Chr3
Chr4
Chr5
Chr6
Chr7
Chr8
Chr9
ChrX
ChrY
Position
SatDNA Family
AbipSat38−2435

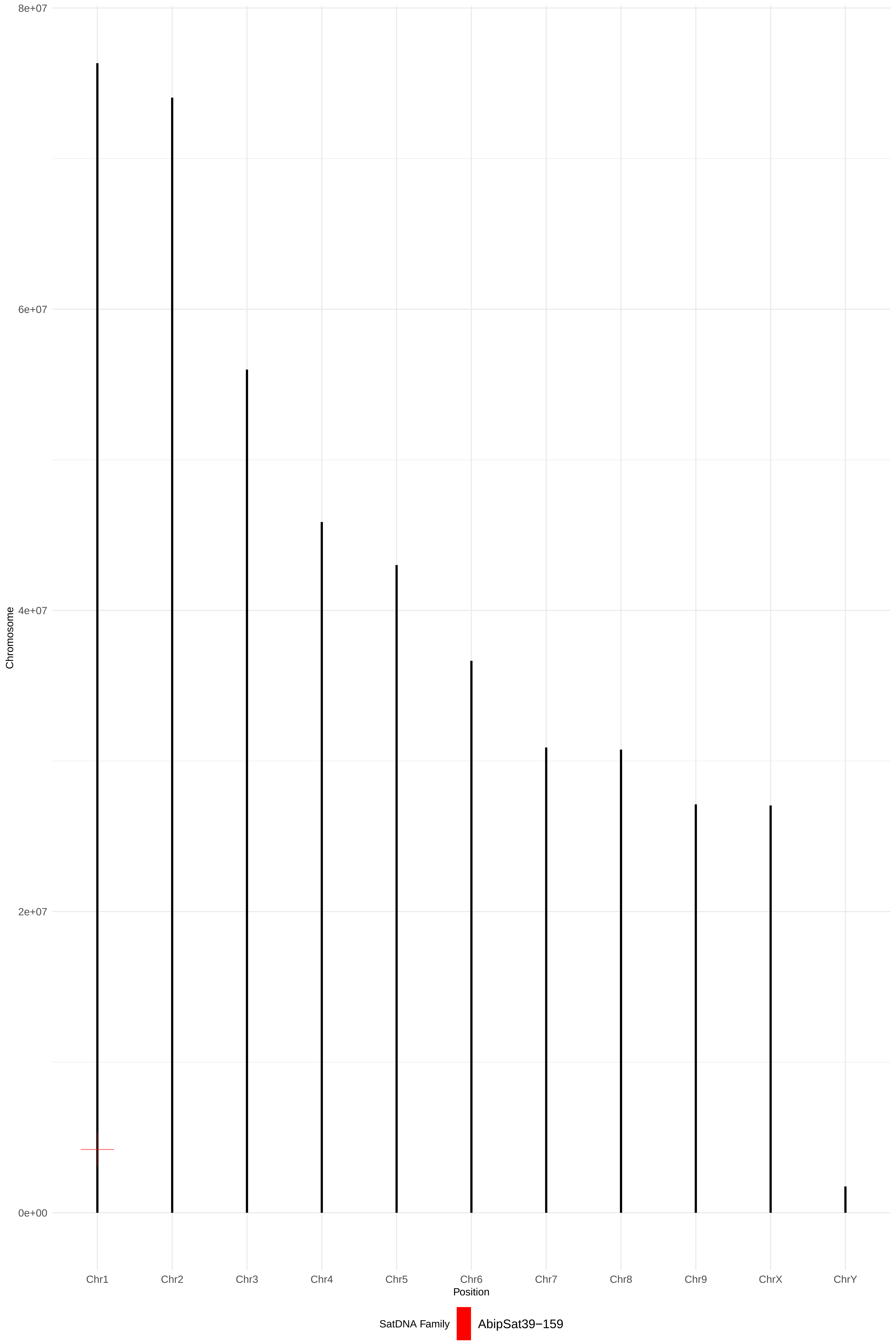

8e+07
6e+07
4e+07
2e+07
0e+00
Chromosome
Chr1
Chr2
Chr3
Chr4
Chr5
Chr6
Chr7
Chr8
Chr9
ChrX
ChrY
Position
SatDNA Family
AbipSat39−159

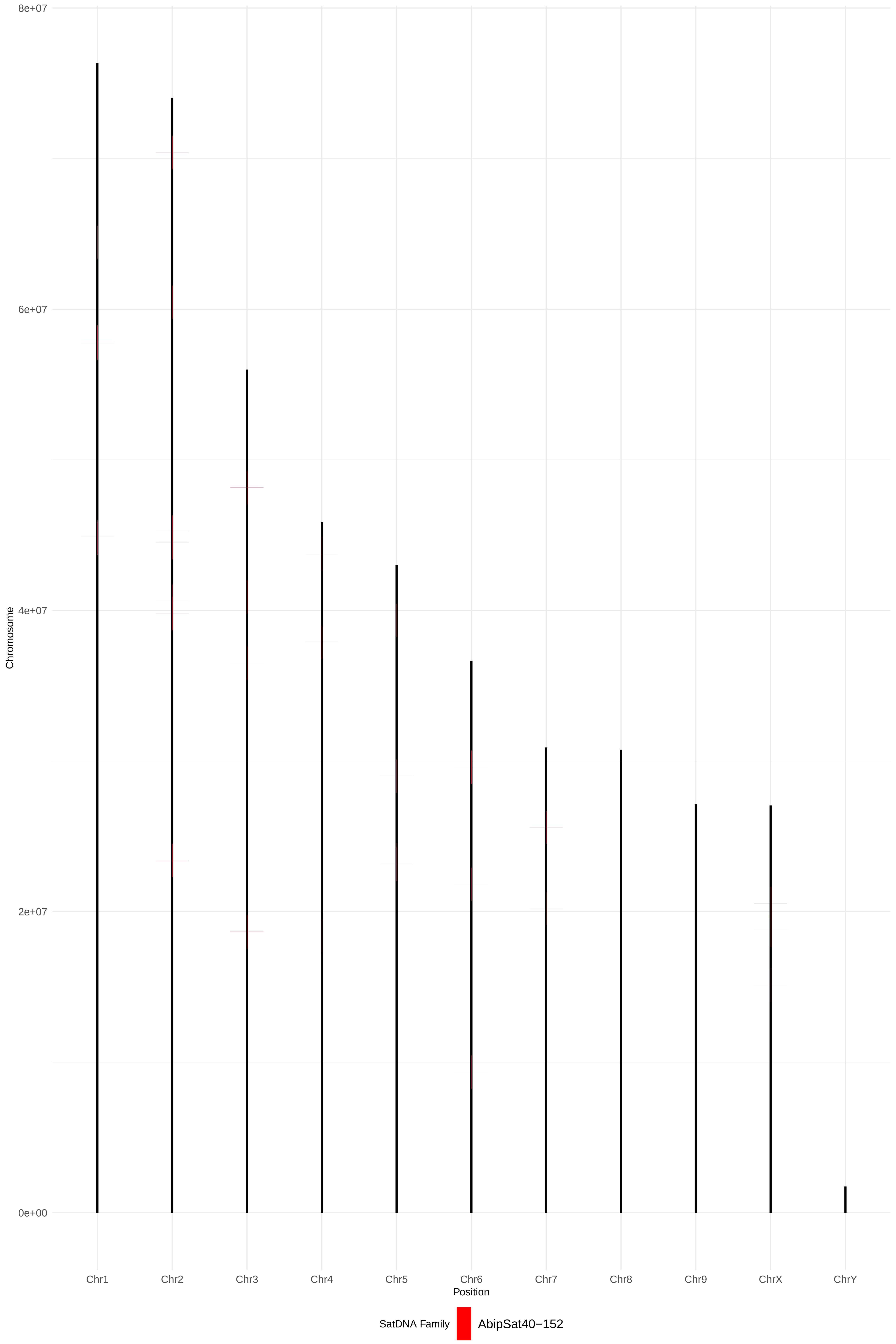
8e+07
6e+07
4e+07
2e+07
0e+00
Chromosome
Chr1
Chr2
Chr3
Chr4
Chr5
Chr6
Chr7
Chr8
Chr9
ChrX
ChrY
Position
SatDNA Family
AbipSat40−152

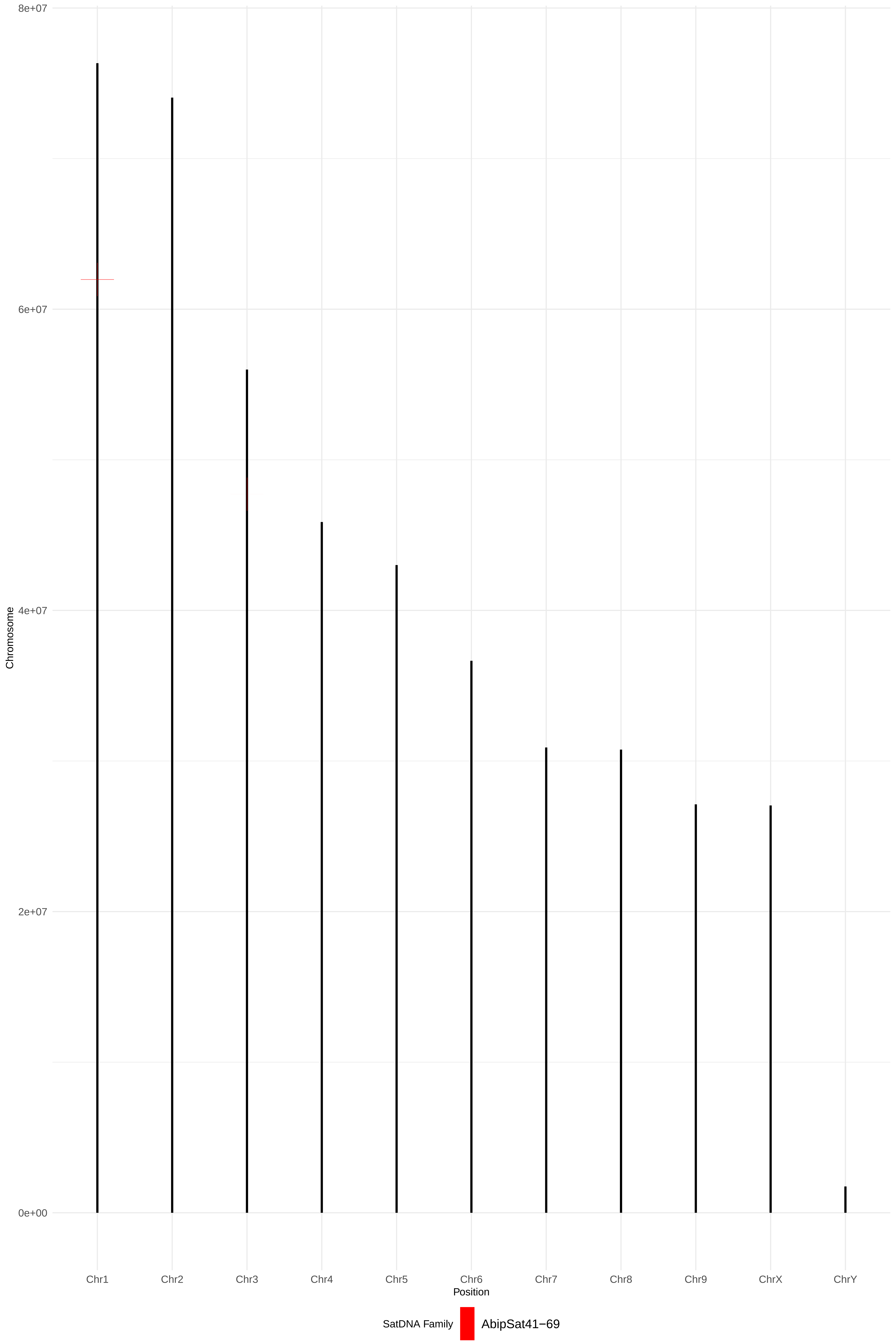

8e+07
6e+07
4e+07
2e+07
0e+00
Chromosome
Chr1
Chr2
Chr3
Chr4
Chr5
Chr6
Chr7
Chr8
Chr9
ChrX
ChrY
Position
SatDNA Family
AbipSat41−69

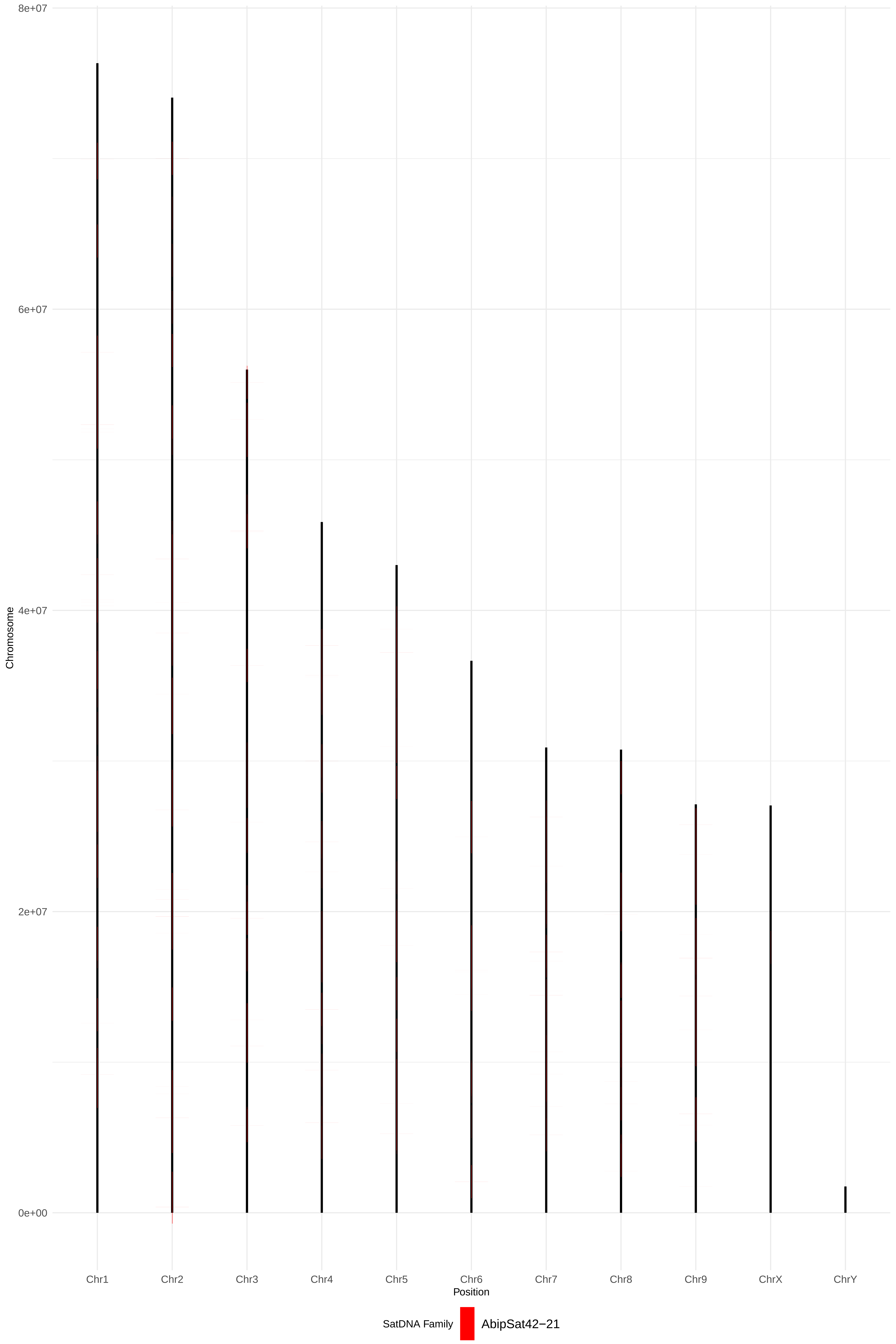

8e+07
6e+07
4e+07
2e+07
0e+00
Chromosome
Chr1
Chr2
Chr3
Chr4
Chr5
Chr6
Chr7
Chr8
Chr9
ChrX
ChrY
Position
SatDNA Family
AbipSat42−21

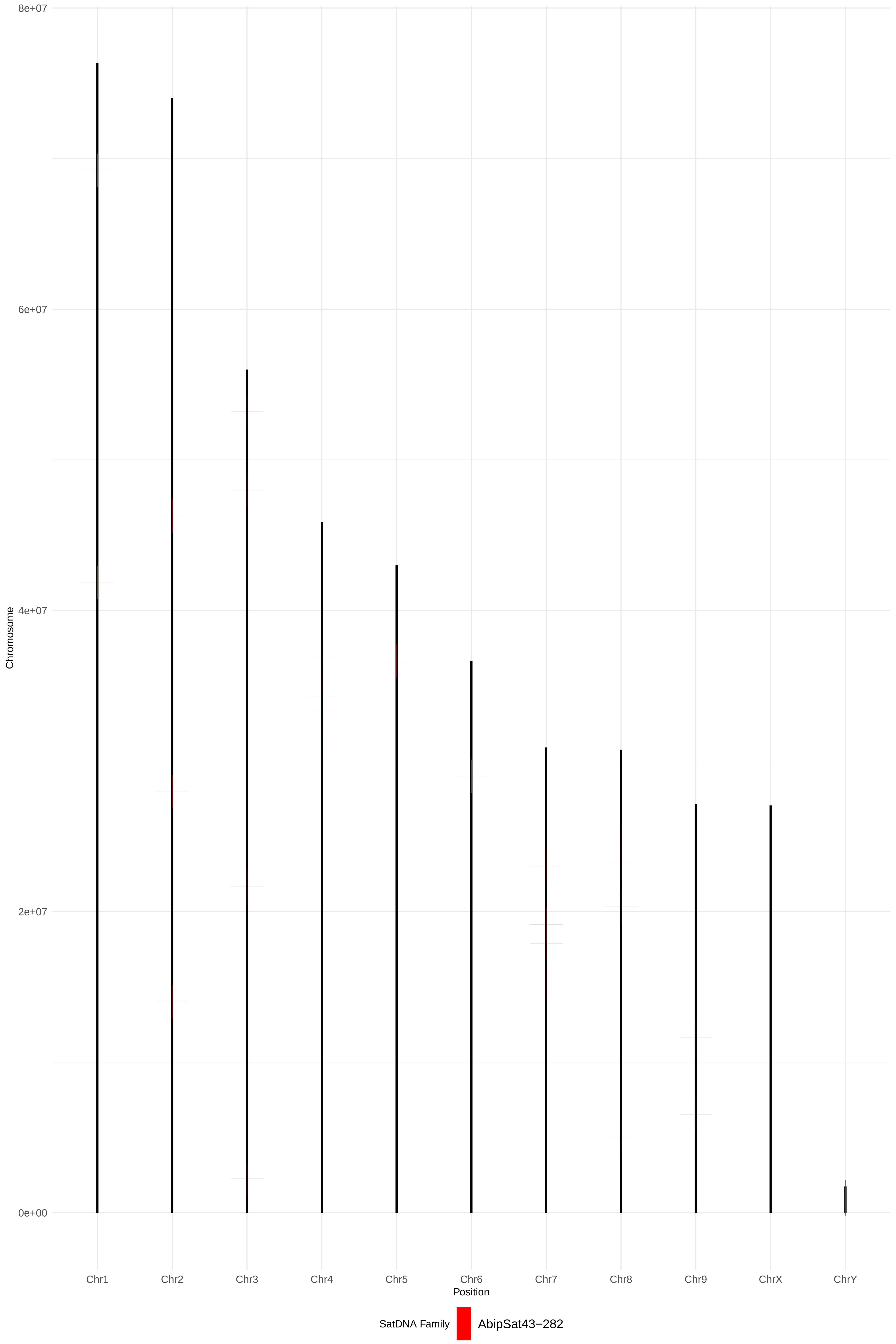

8e+07
6e+07
4e+07
2e+07
0e+00
Chromosome
Chr1
Chr2
Chr3
Chr4
Chr5
Chr6
Chr7
Chr8
Chr9
ChrX
ChrY
Position
SatDNA Family
AbipSat43−282

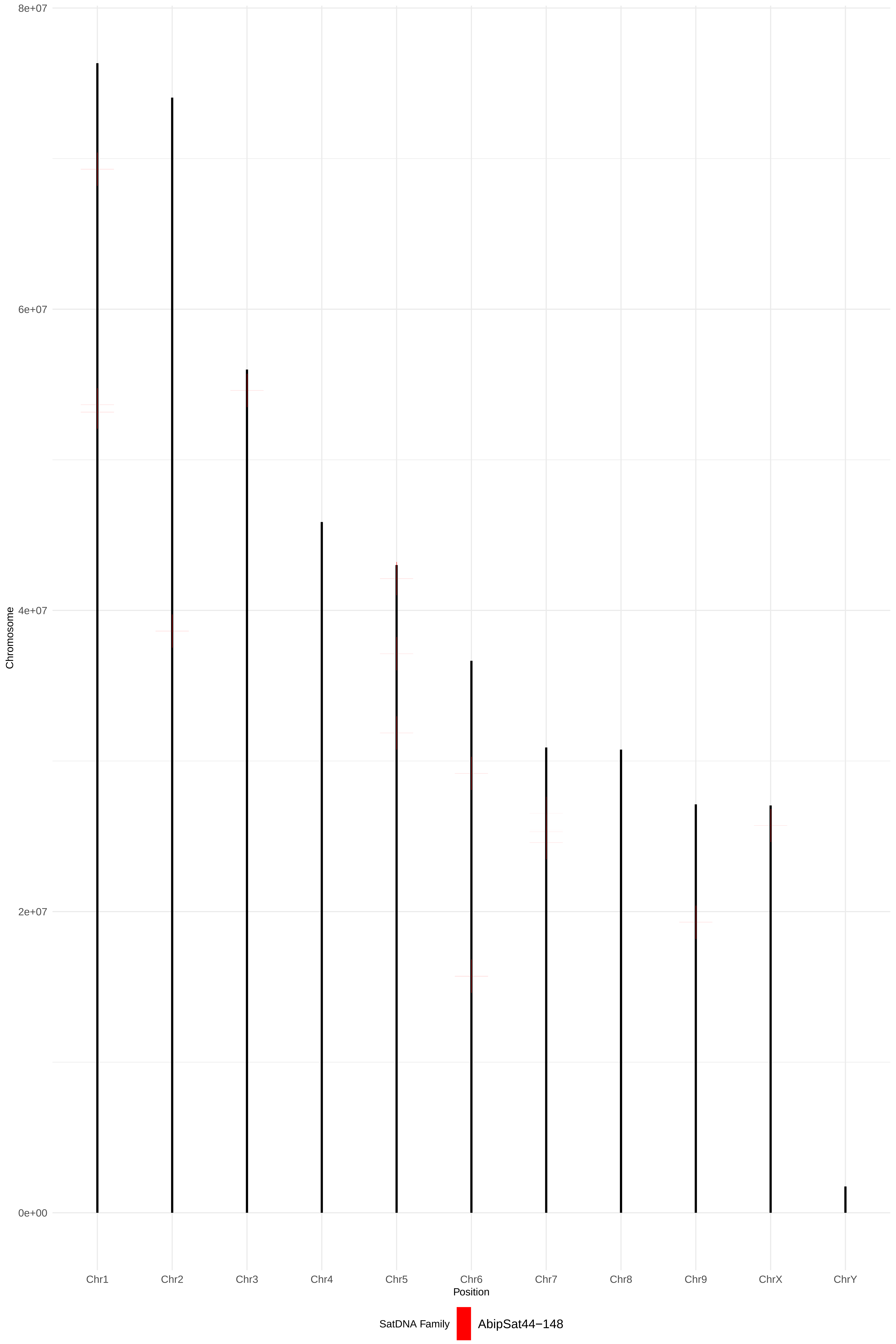

8e+07
6e+07
4e+07
2e+07
0e+00
Chromosome
Chr1
Chr2
Chr3
Chr4
Chr5
Chr6
Chr7
Chr8
Chr9
ChrX
ChrY
Position
SatDNA Family
AbipSat44−148

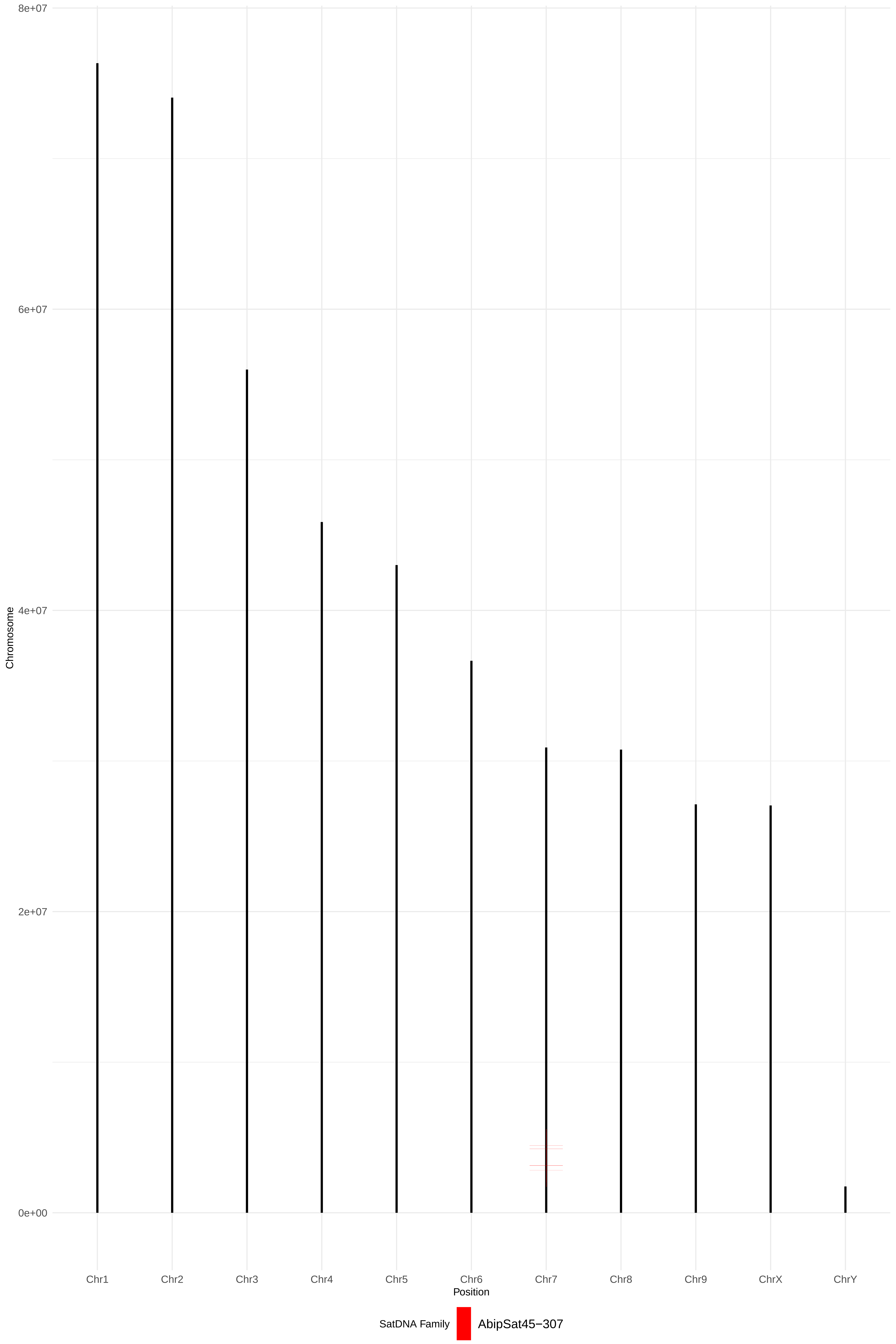

8e+07
6e+07
4e+07
2e+07
0e+00
Chromosome
Chr1
Chr2
Chr3
Chr4
Chr5
Chr6
Chr7
Chr8
Chr9
ChrX
ChrY
Position
SatDNA Family
AbipSat45−307

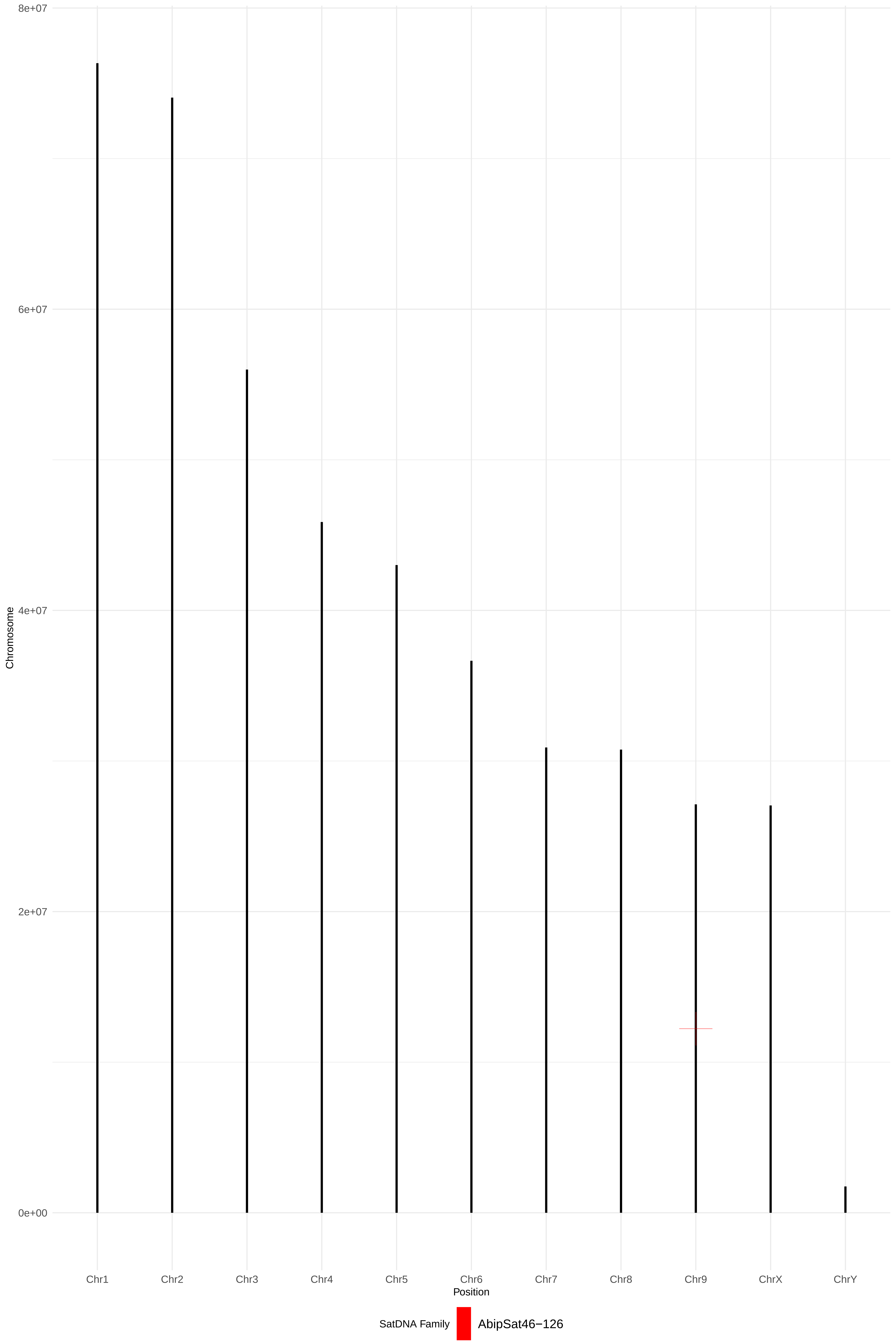
8e+07
6e+07
4e+07
2e+07
0e+00
Chromosome
Chr1
Chr2
Chr3
Chr4
Chr5
Chr6
Chr7
Chr8
Chr9
ChrX
ChrY
Position
SatDNA Family
AbipSat46−126

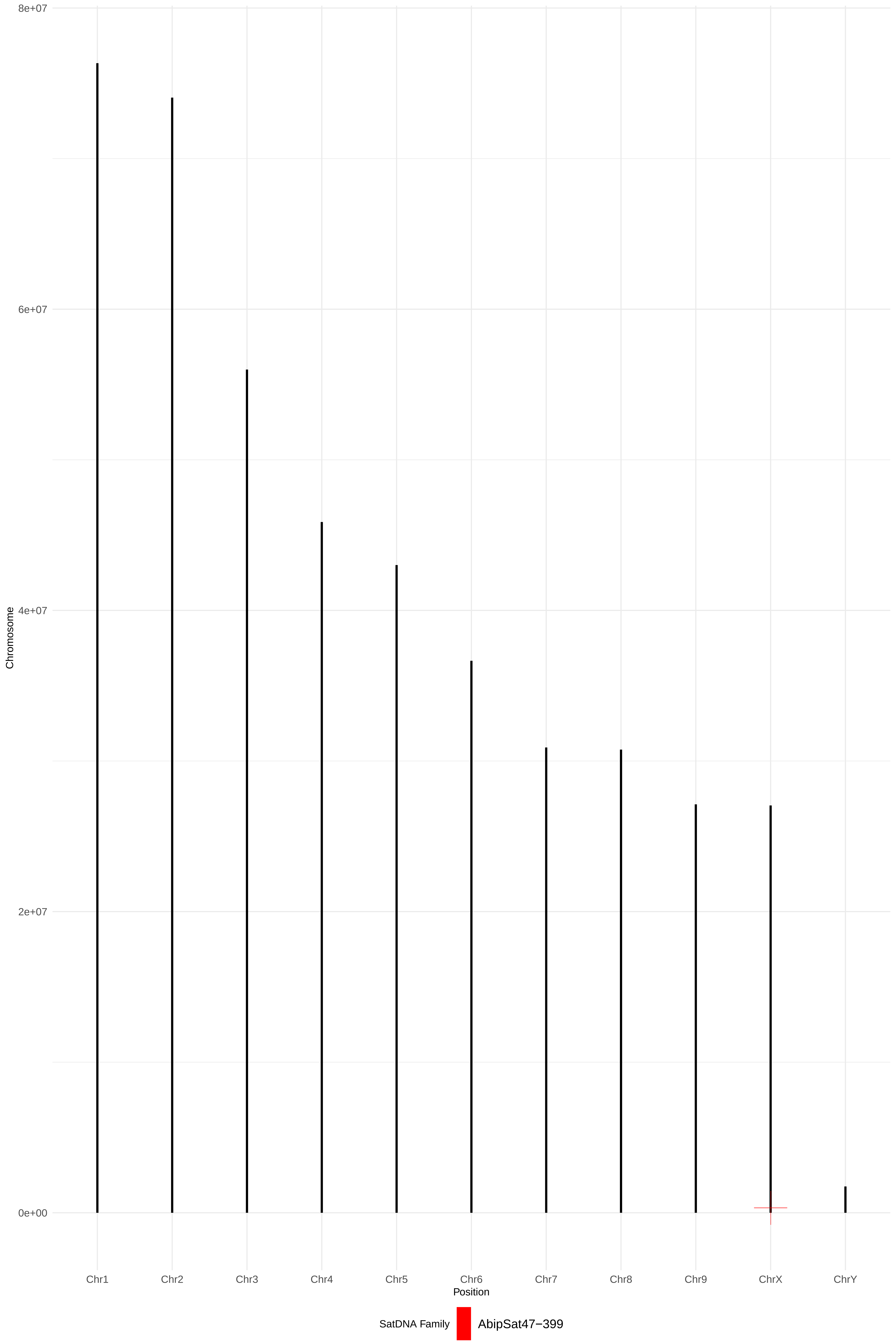

8e+07
6e+07
4e+07
2e+07
0e+00
Chromosome
Chr1
Chr2
Chr3
Chr4
Chr5
Chr6
Chr7
Chr8
Chr9
ChrX
ChrY
Position
SatDNA Family
AbipSat47−399

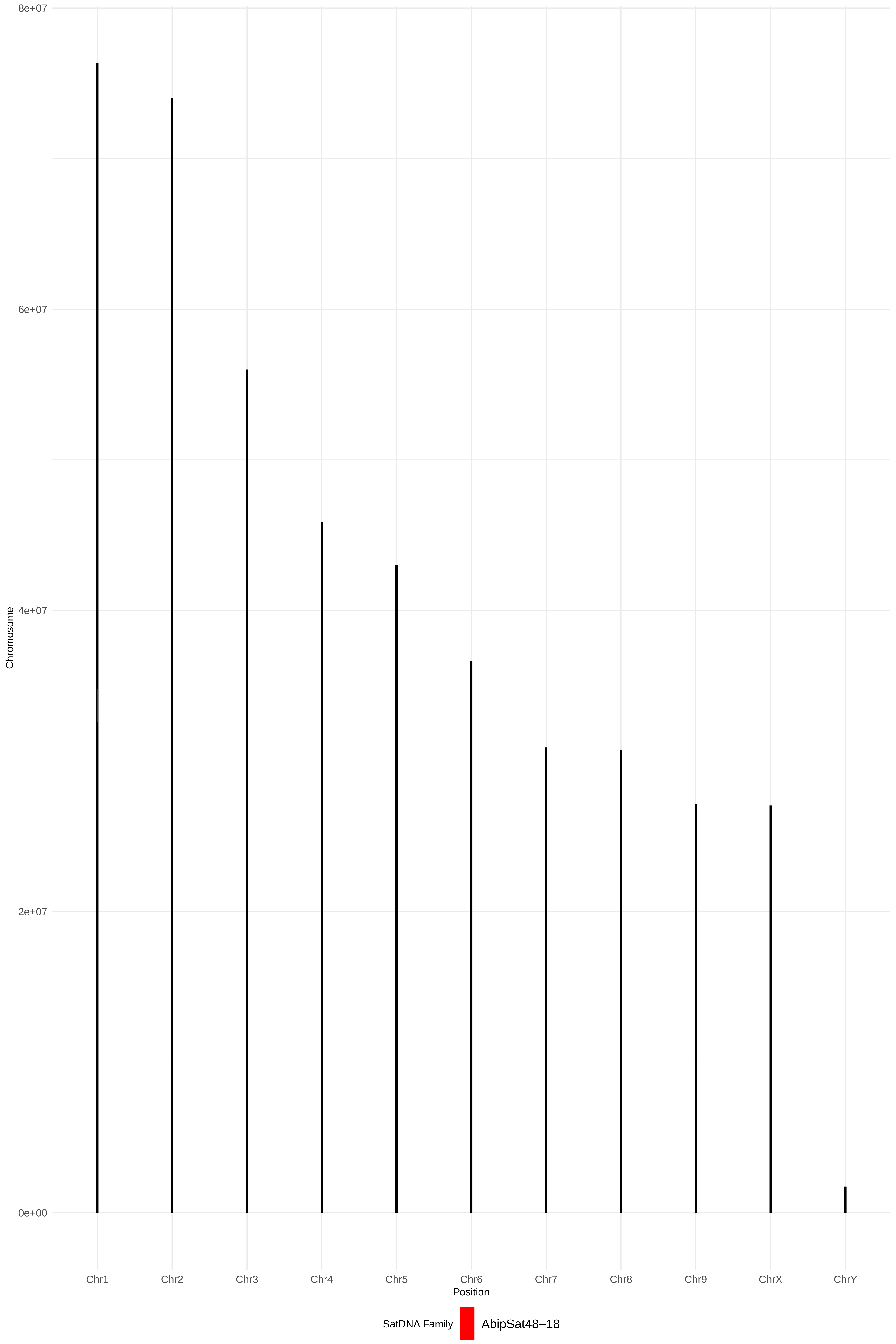
8e+07
6e+07
4e+07
2e+07
0e+00
Chromosome
Chr1
Chr2
Chr3
Chr4
Chr5
Chr6
Chr7
Chr8
Chr9
ChrX
ChrY
Position
SatDNA Family
AbipSat48−18

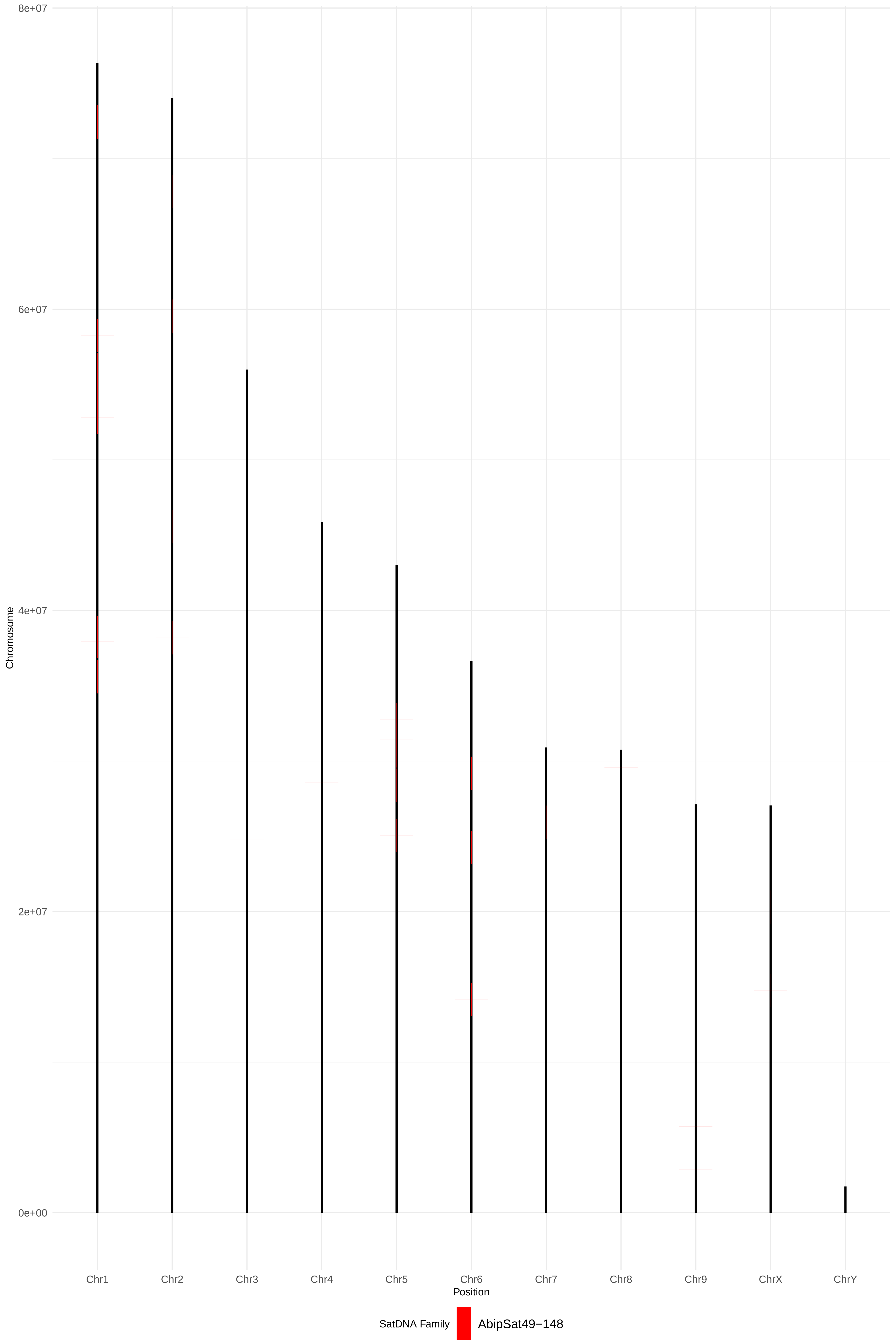

8e+07
6e+07
4e+07
2e+07
0e+00
Chromosome
Chr1
Chr2
Chr3
Chr4
Chr5
Chr6
Chr7
Chr8
Chr9
ChrX
ChrY
Position
SatDNA Family
AbipSat49−148

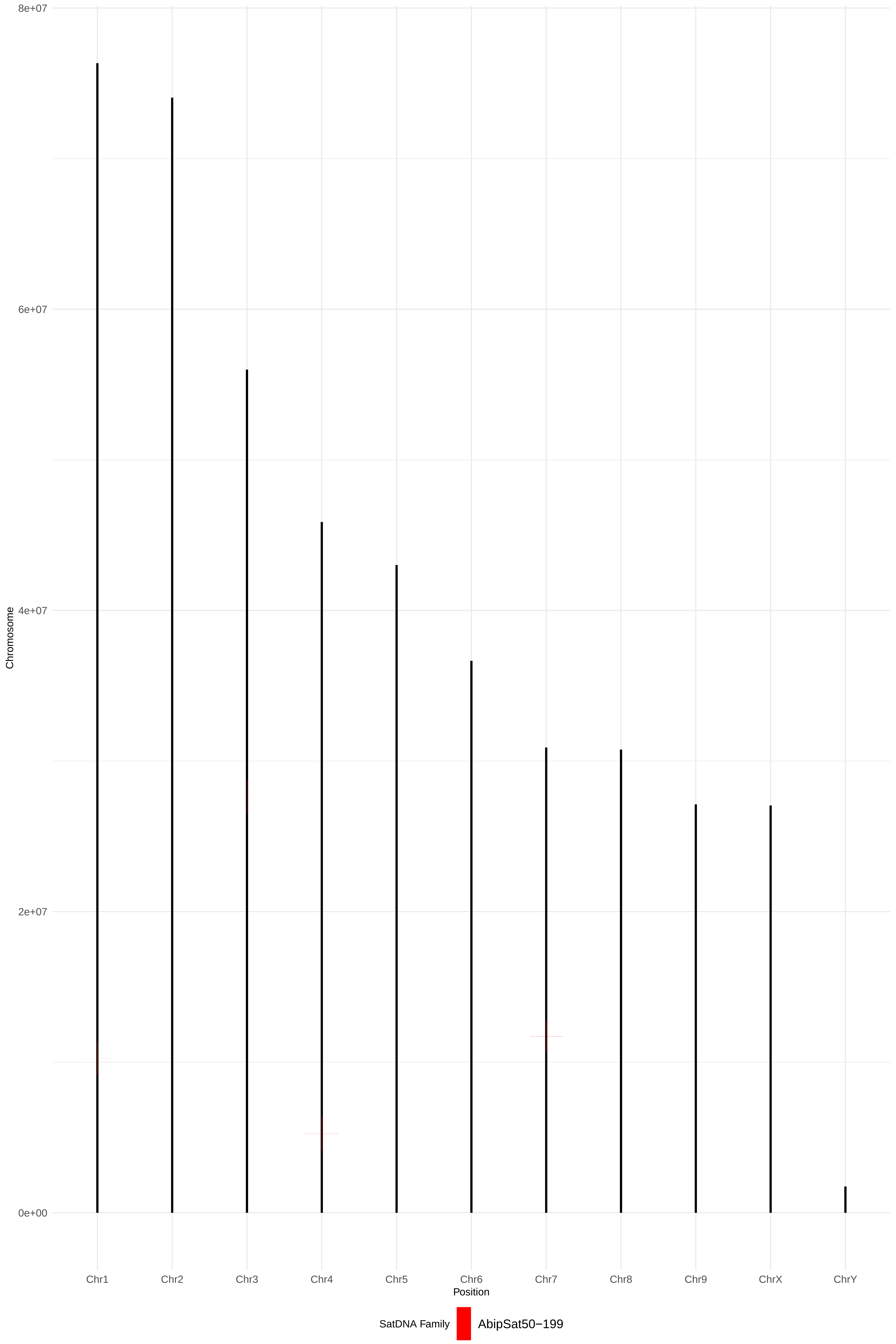
8e+07
6e+07
4e+07
2e+07
0e+00
Chromosome
Chr1
Chr2
Chr3
Chr4
Chr5
Chr6
Chr7
Chr8
Chr9
ChrX
ChrY
Position
SatDNA Family
AbipSat50−199

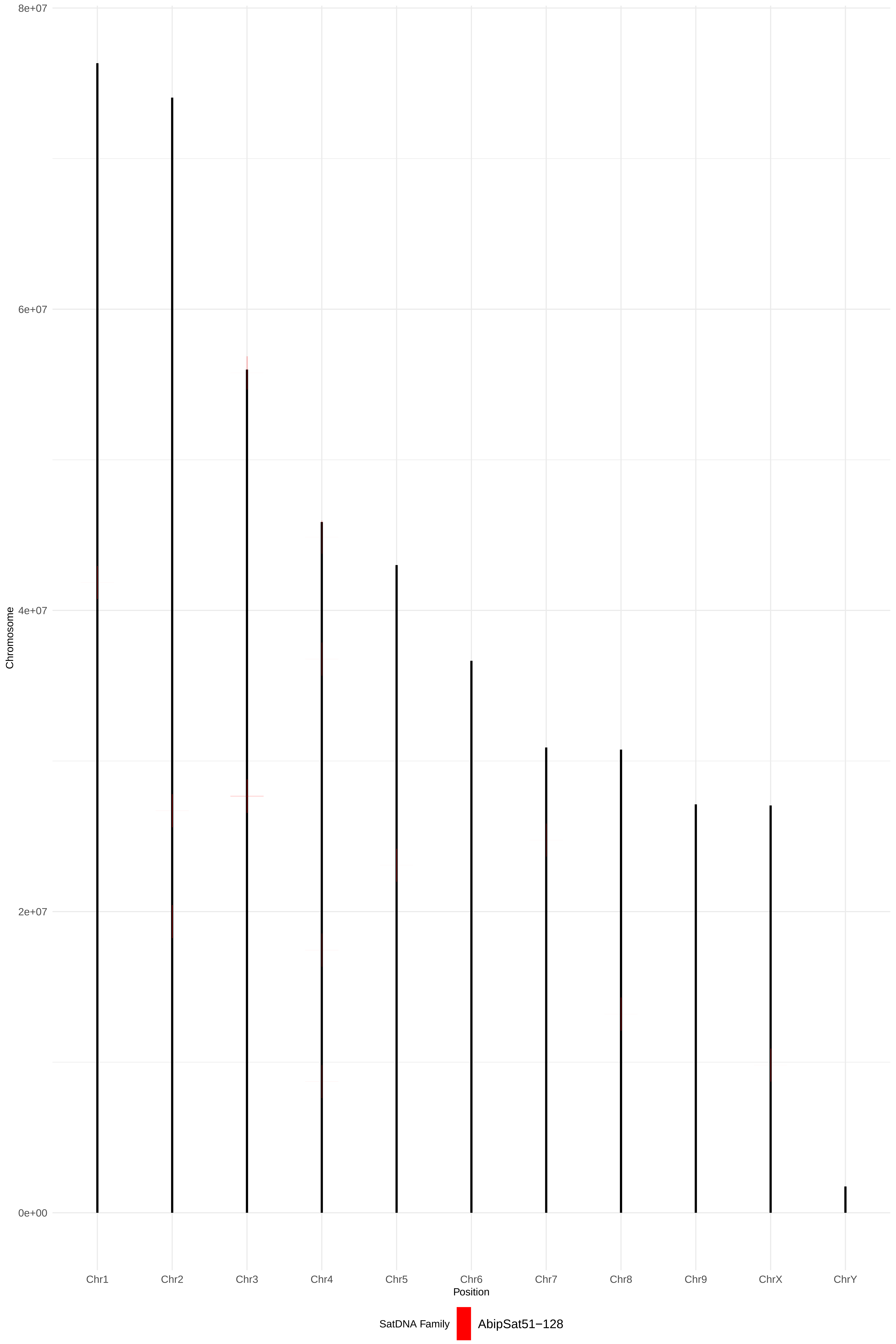

8e+07
6e+07
4e+07
2e+07
0e+00
Chromosome
Chr1
Chr2
Chr3
Chr4
Chr5
Chr6
Chr7
Chr8
Chr9
ChrX
ChrY
Position
SatDNA Family
AbipSat51−128

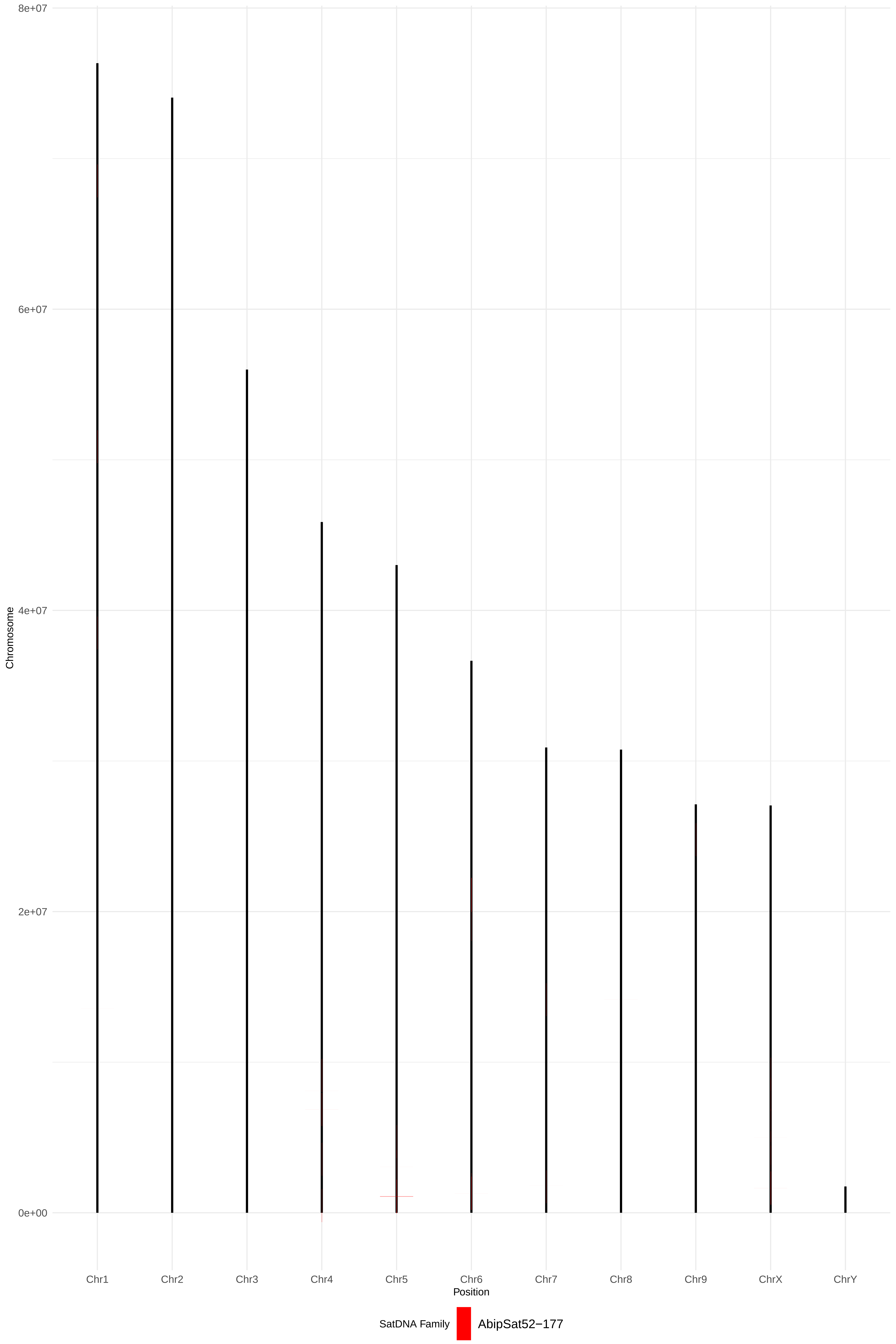

8e+07
6e+07
4e+07
2e+07
0e+00
Chromosome
Chr1
Chr2
Chr3
Chr4
Chr5
Chr6
Chr7
Chr8
Chr9
ChrX
ChrY
Position
SatDNA Family
AbipSat52−177

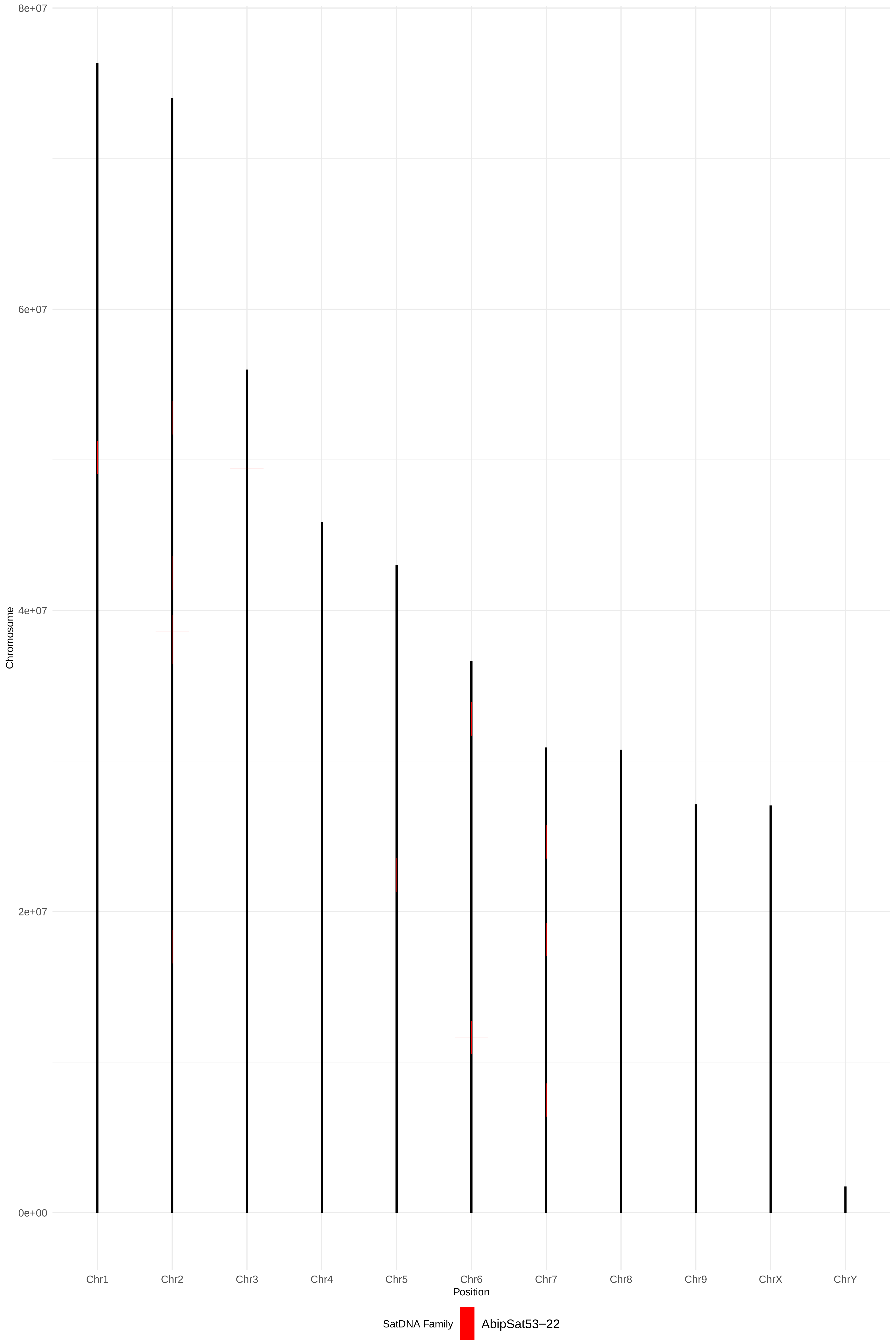

8e+07
6e+07
4e+07
2e+07
0e+00
Chromosome
Chr1
Chr2
Chr3
Chr4
Chr5
Chr6
Chr7
Chr8
Chr9
ChrX
ChrY
Position
SatDNA Family
AbipSat53−22

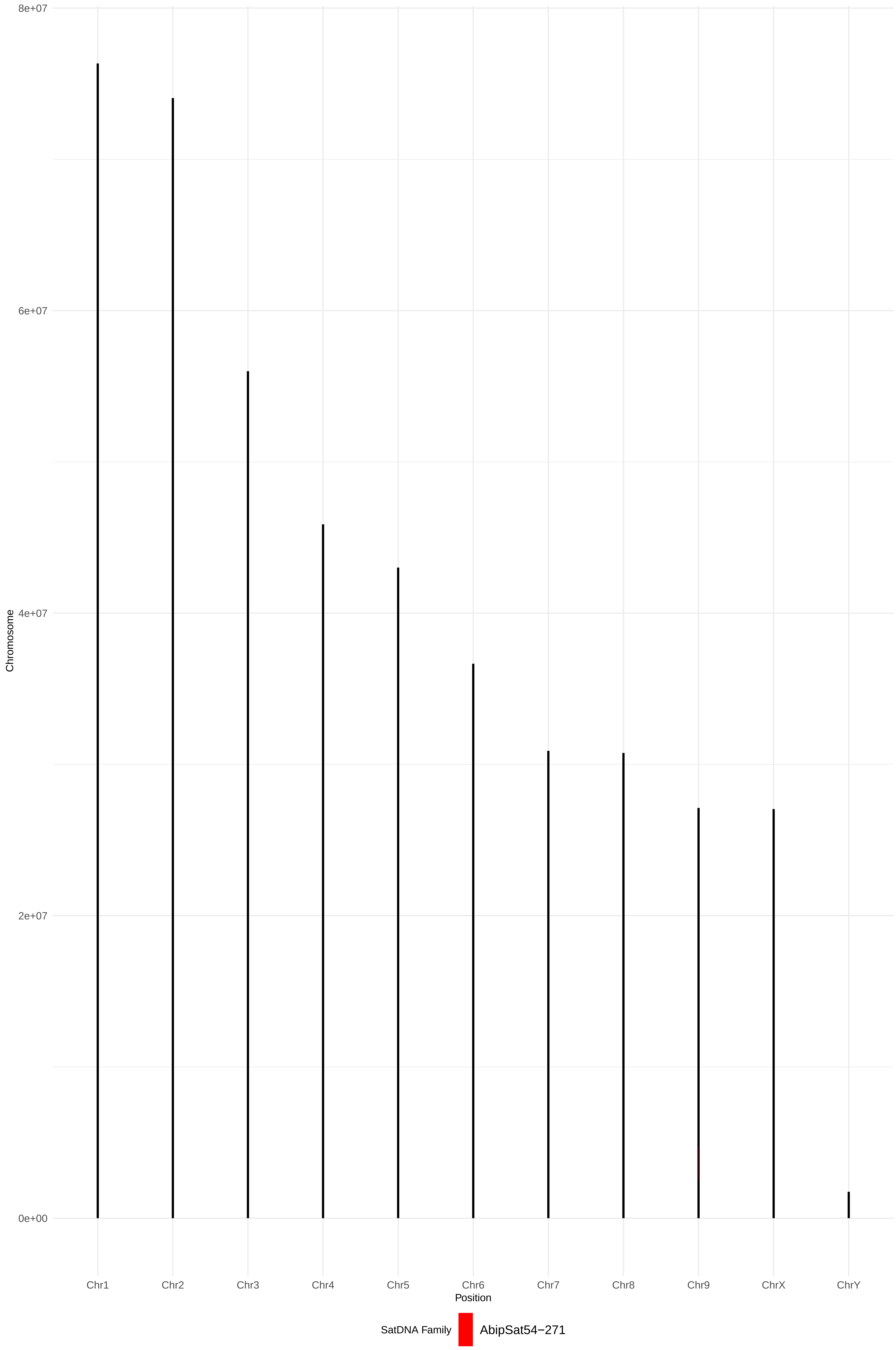
8e+07
6e+07
4e+07
2e+07
0e+00
Chromosome
Chr1
Chr2
Chr3
Chr4
Chr5
Chr6
Chr7
Chr8
Chr9
ChrX
ChrY
Position
SatDNA Family
AbipSat54−271

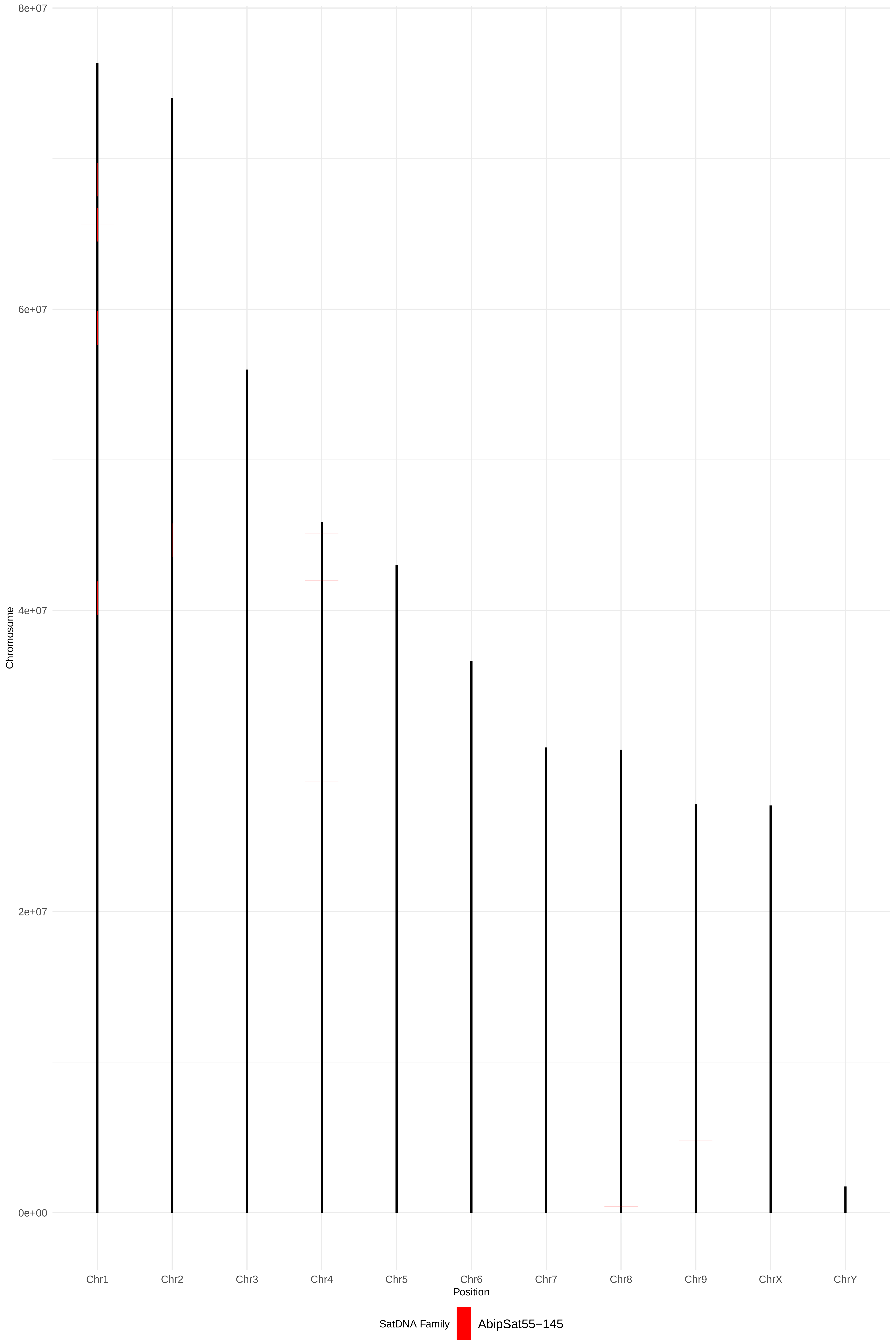

8e+07
6e+07
4e+07
2e+07
0e+00
Chromosome
Chr1
Chr2
Chr3
Chr4
Chr5
Chr6
Chr7
Chr8
Chr9
ChrX
ChrY
Position
SatDNA Family
AbipSat55−145

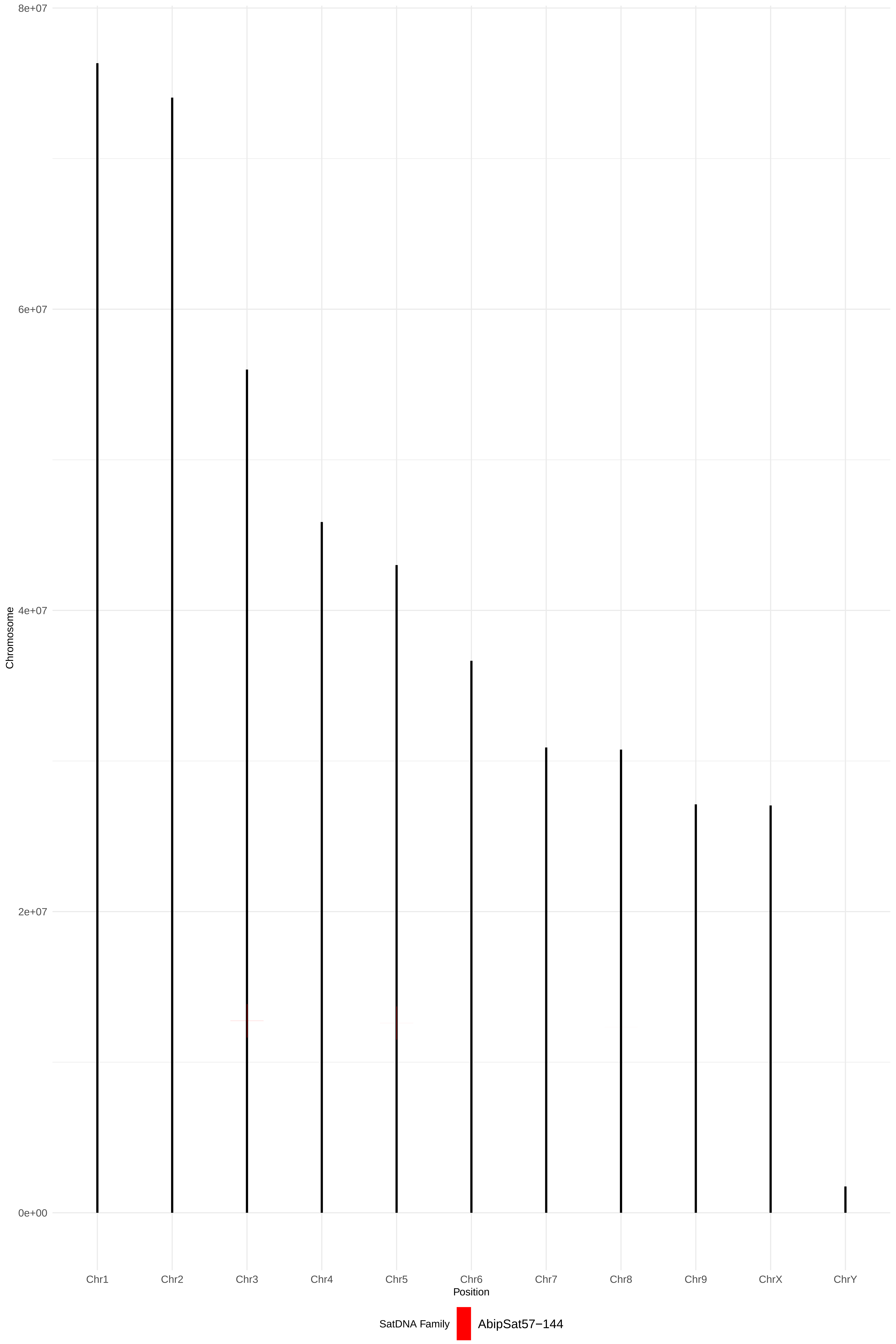

8e+07
6e+07
4e+07
2e+07
0e+00
Chromosome
Chr1
Chr2
Chr3
Chr4
Chr5
Chr6
Chr7
Chr8
Chr9
ChrX
ChrY
Position
SatDNA Family
AbipSat57−144

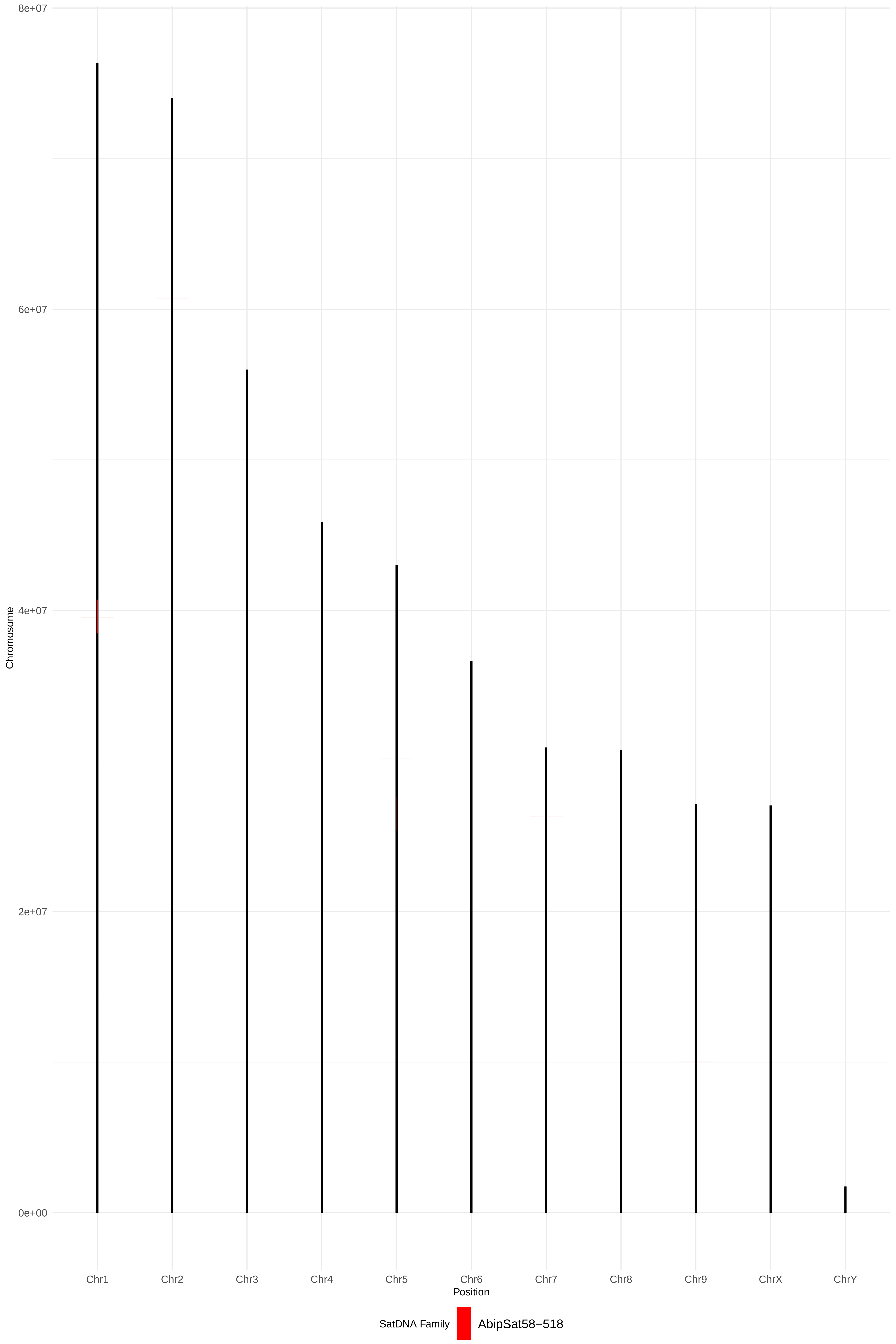

8e+07
6e+07
4e+07
2e+07
0e+00
Chromosome
Chr1
Chr2
Chr3
Chr4
Chr5
Chr6
Chr7
Chr8
Chr9
ChrX
ChrY
Position
SatDNA Family
AbipSat58−518

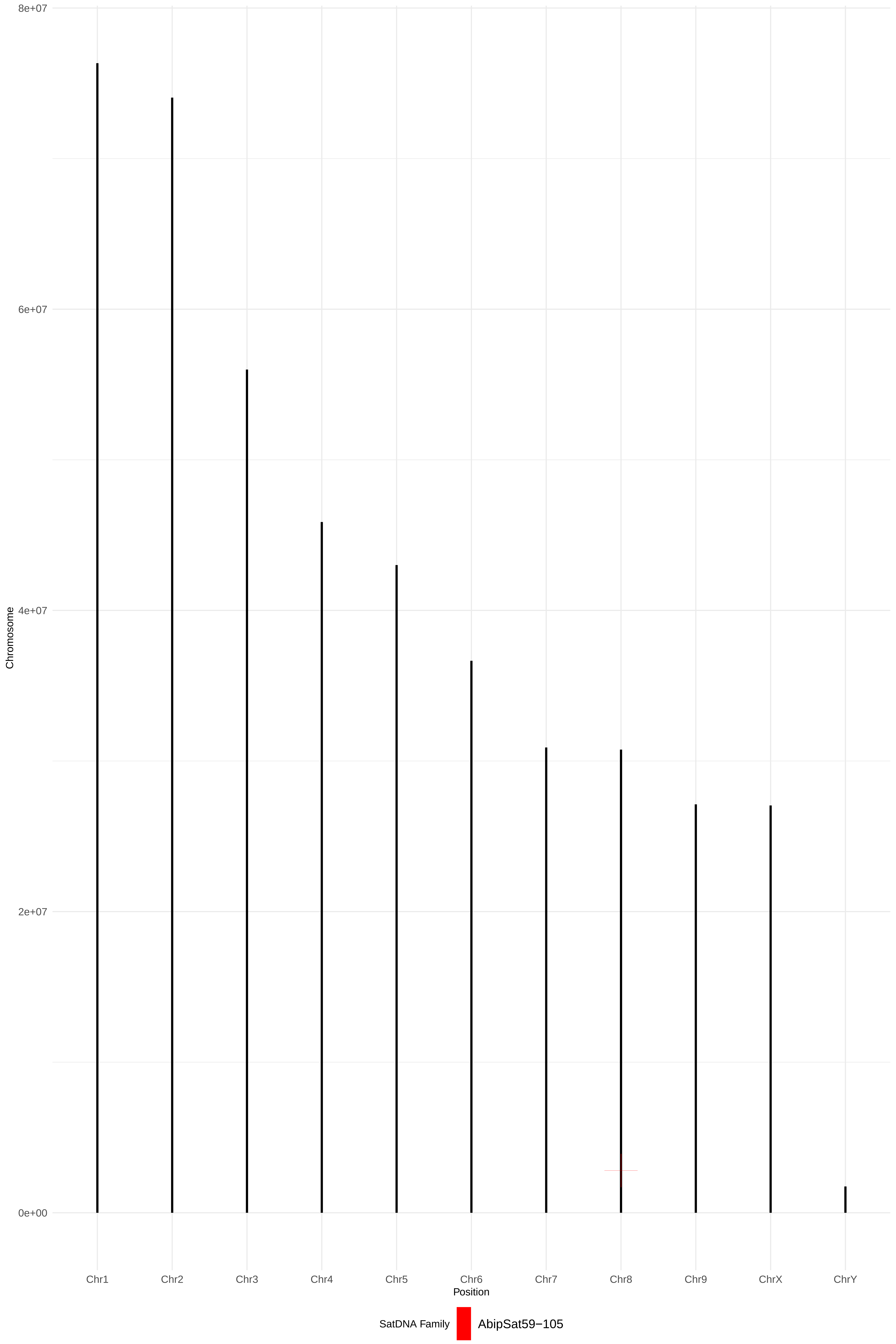

8e+07
6e+07
4e+07
2e+07
0e+00
Chromosome
Chr1
Chr2
Chr3
Chr4
Chr5
Chr6
Chr7
Chr8
Chr9
ChrX
ChrY
Position
SatDNA Family
AbipSat59−105

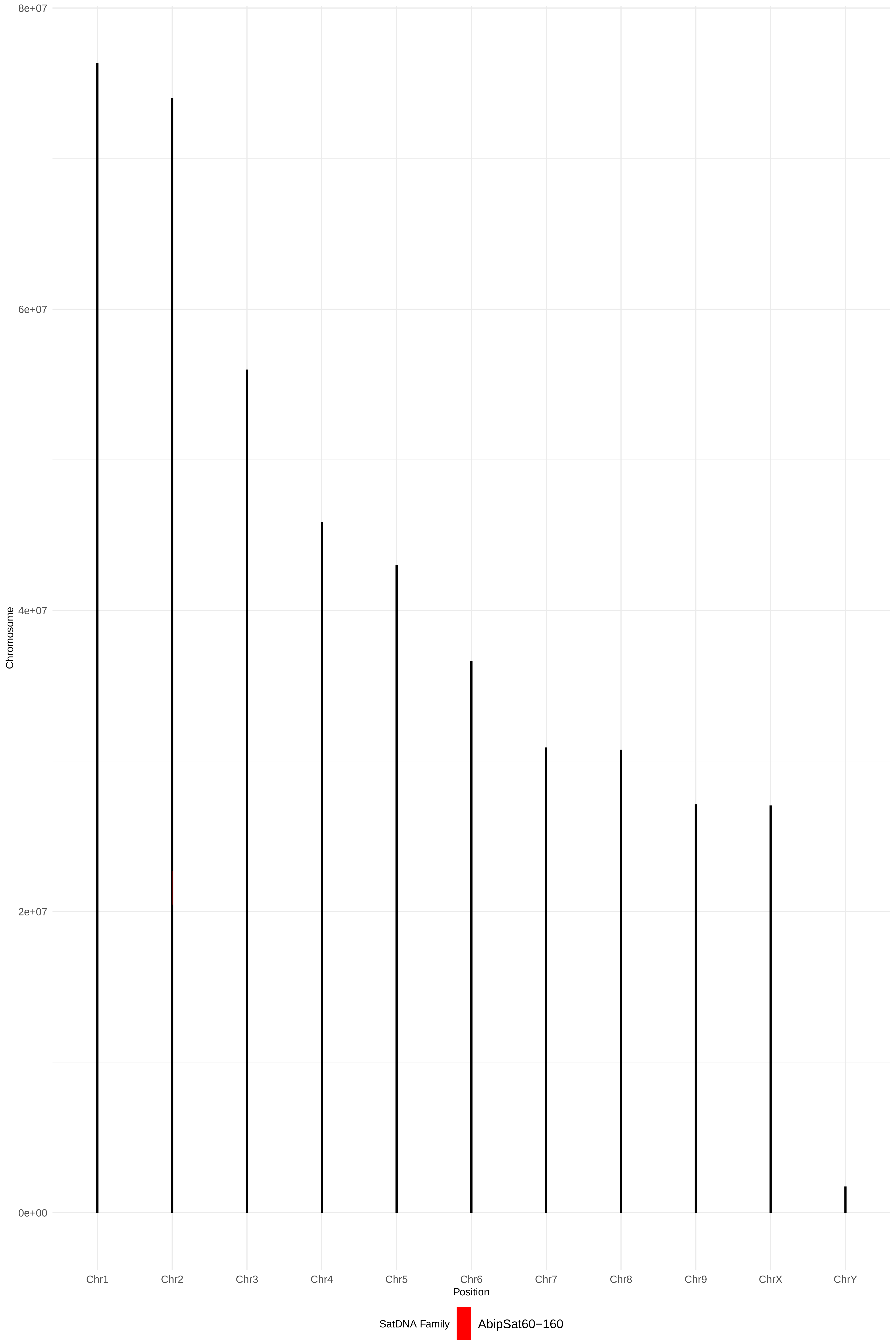

8e+07
6e+07
4e+07
2e+07
0e+00
Chromosome
Chr1
Chr2
Chr3
Chr4
Chr5
Chr6
Chr7
Chr8
Chr9
ChrX
ChrY
Position
SatDNA Family
AbipSat60−160

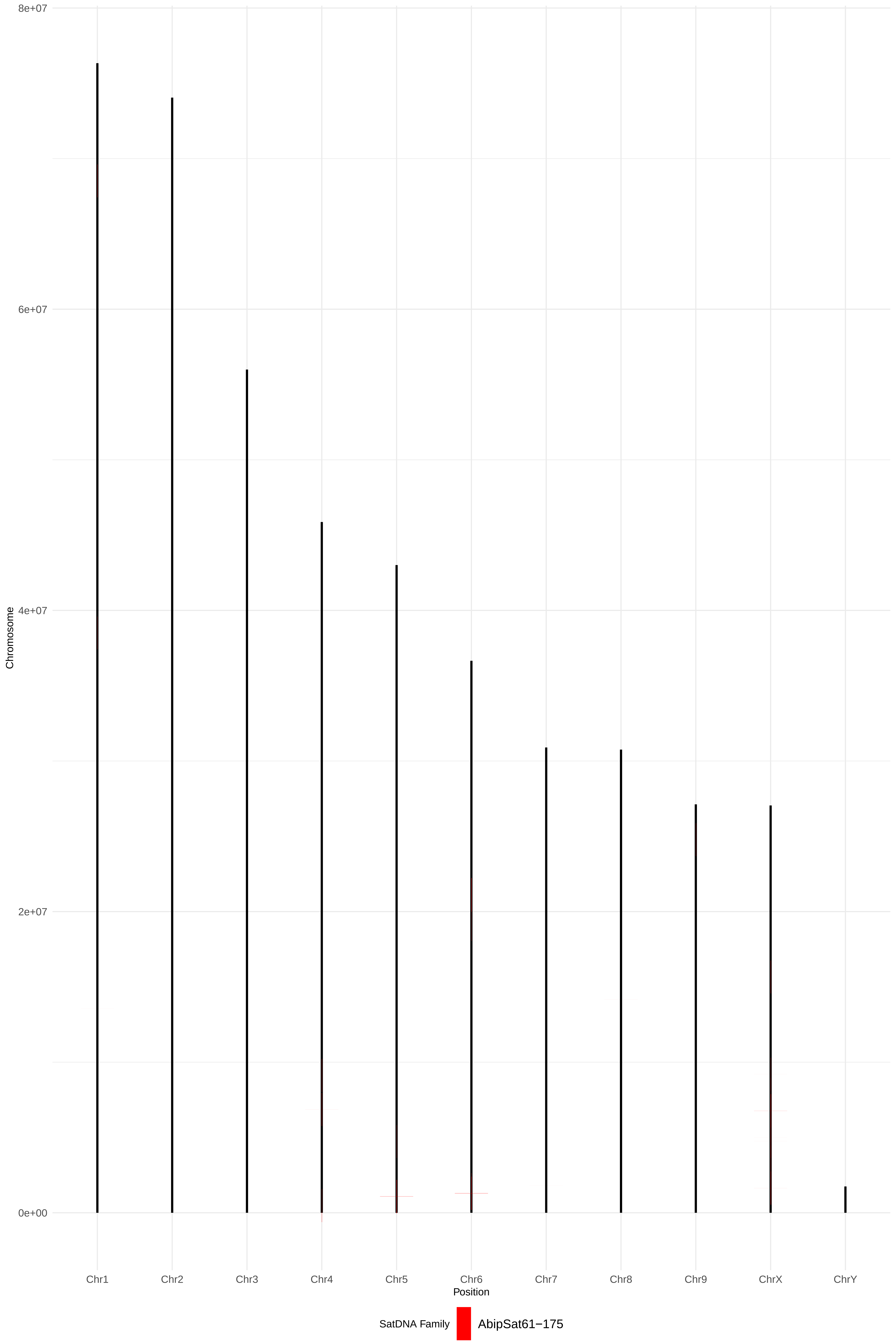

8e+07
6e+07
4e+07
2e+07
0e+00
Chromosome
Chr1
Chr2
Chr3
Chr4
Chr5
Chr6
Chr7
Chr8
Chr9
ChrX
ChrY
Position
SatDNA Family
AbipSat61−175

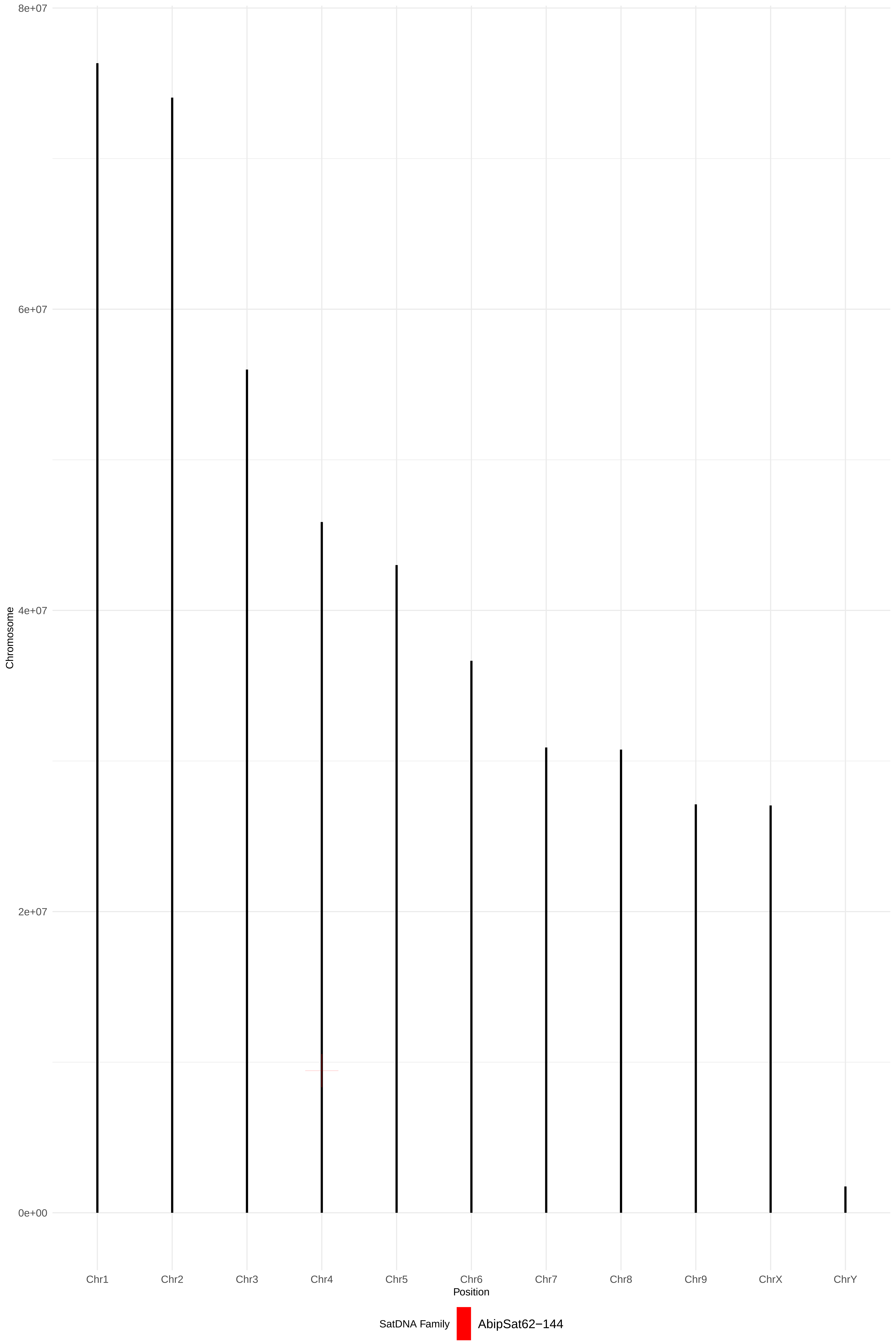

8e+07
6e+07
4e+07
2e+07
0e+00
Chromosome
Chr1
Chr2
Chr3
Chr4
Chr5
Chr6
Chr7
Chr8
Chr9
ChrX
ChrY
Position
SatDNA Family
AbipSat62−144

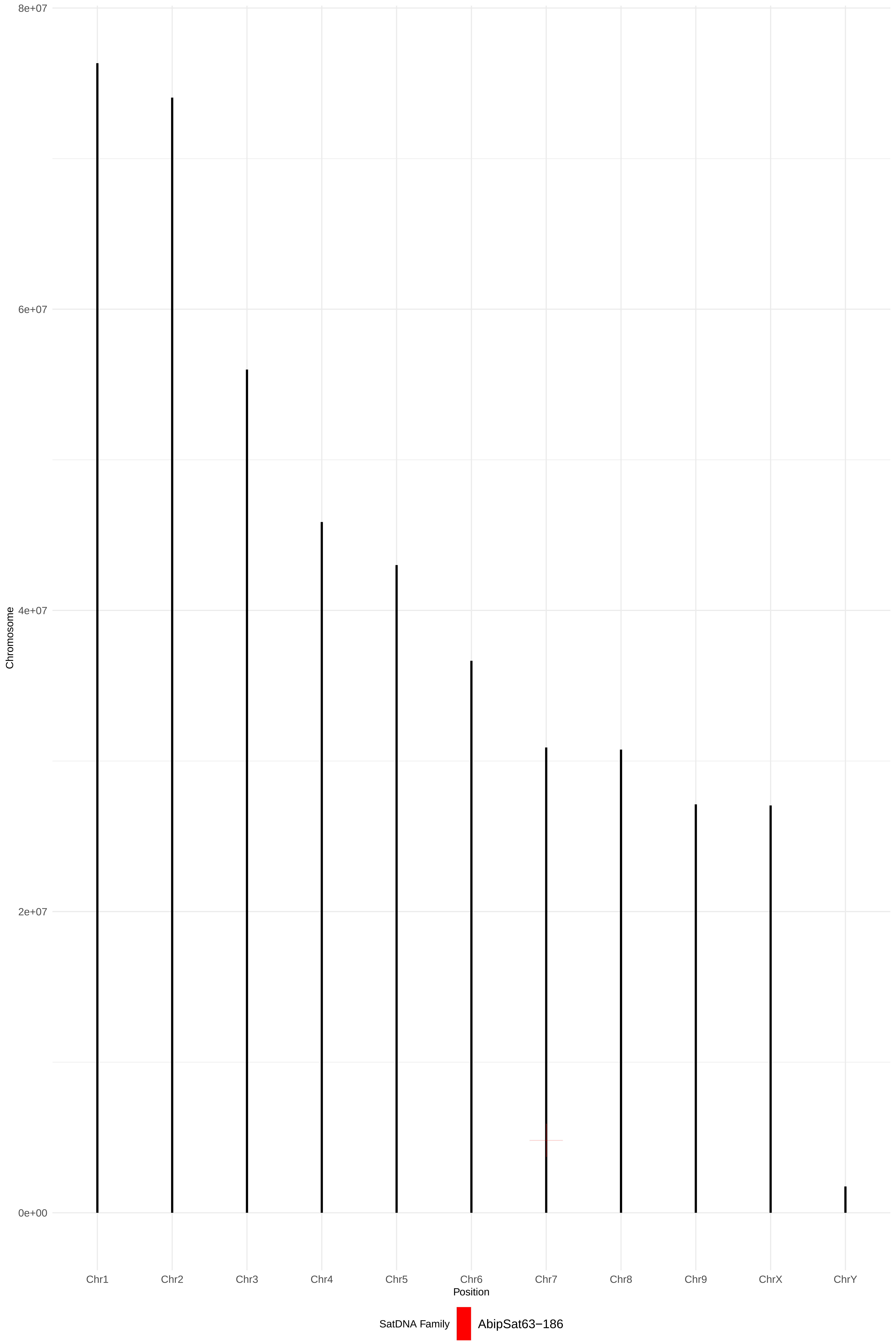

8e+07
6e+07
4e+07
2e+07
0e+00
Chromosome
Chr1
Chr2
Chr3
Chr4
Chr5
Chr6
Chr7
Chr8
Chr9
ChrX
ChrY
Position
SatDNA Family
AbipSat63−186

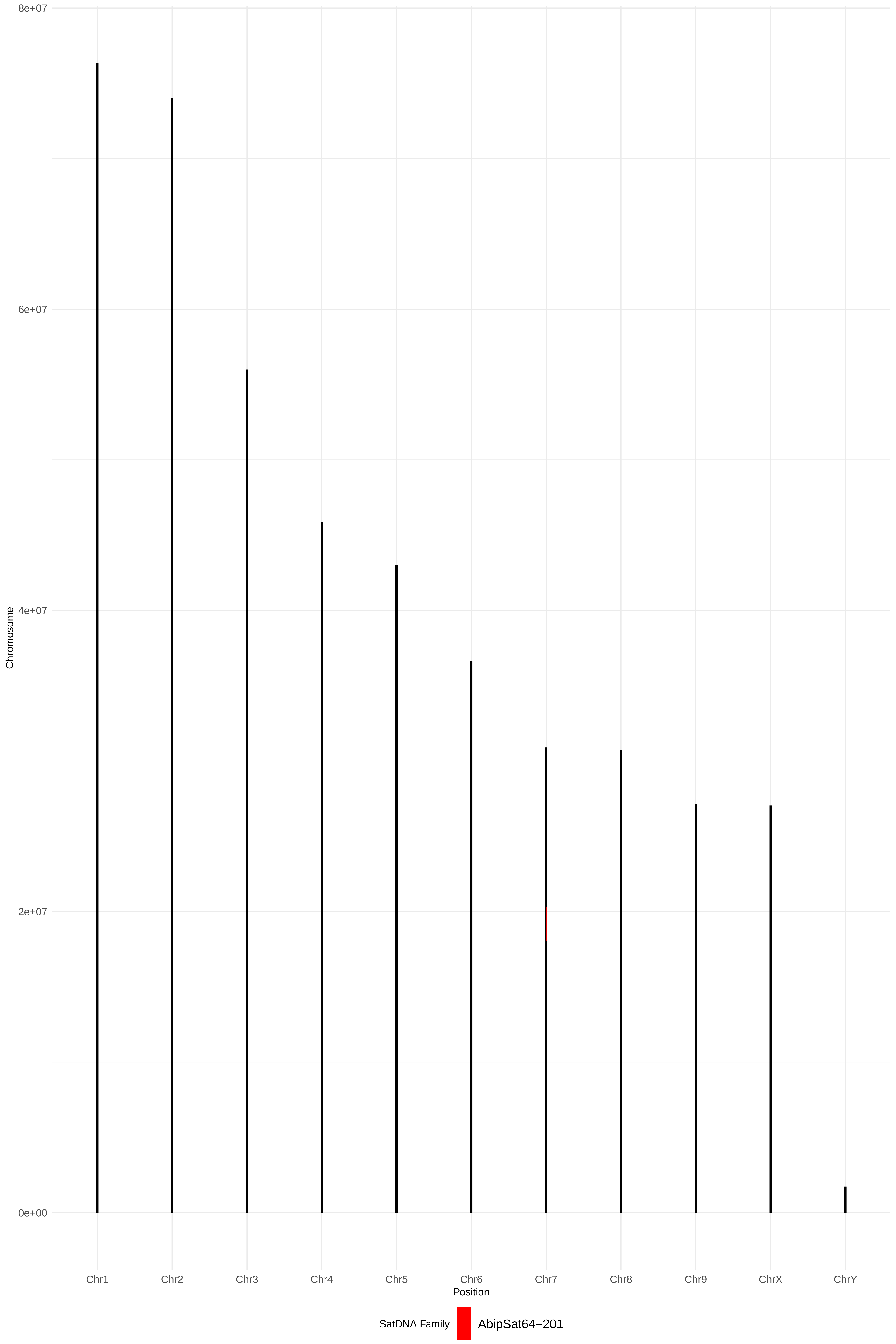

8e+07
6e+07
4e+07
2e+07
0e+00
Chromosome
Chr1
Chr2
Chr3
Chr4
Chr5
Chr6
Chr7
Chr8
Chr9
ChrX
ChrY
Position
SatDNA Family
AbipSat64−201

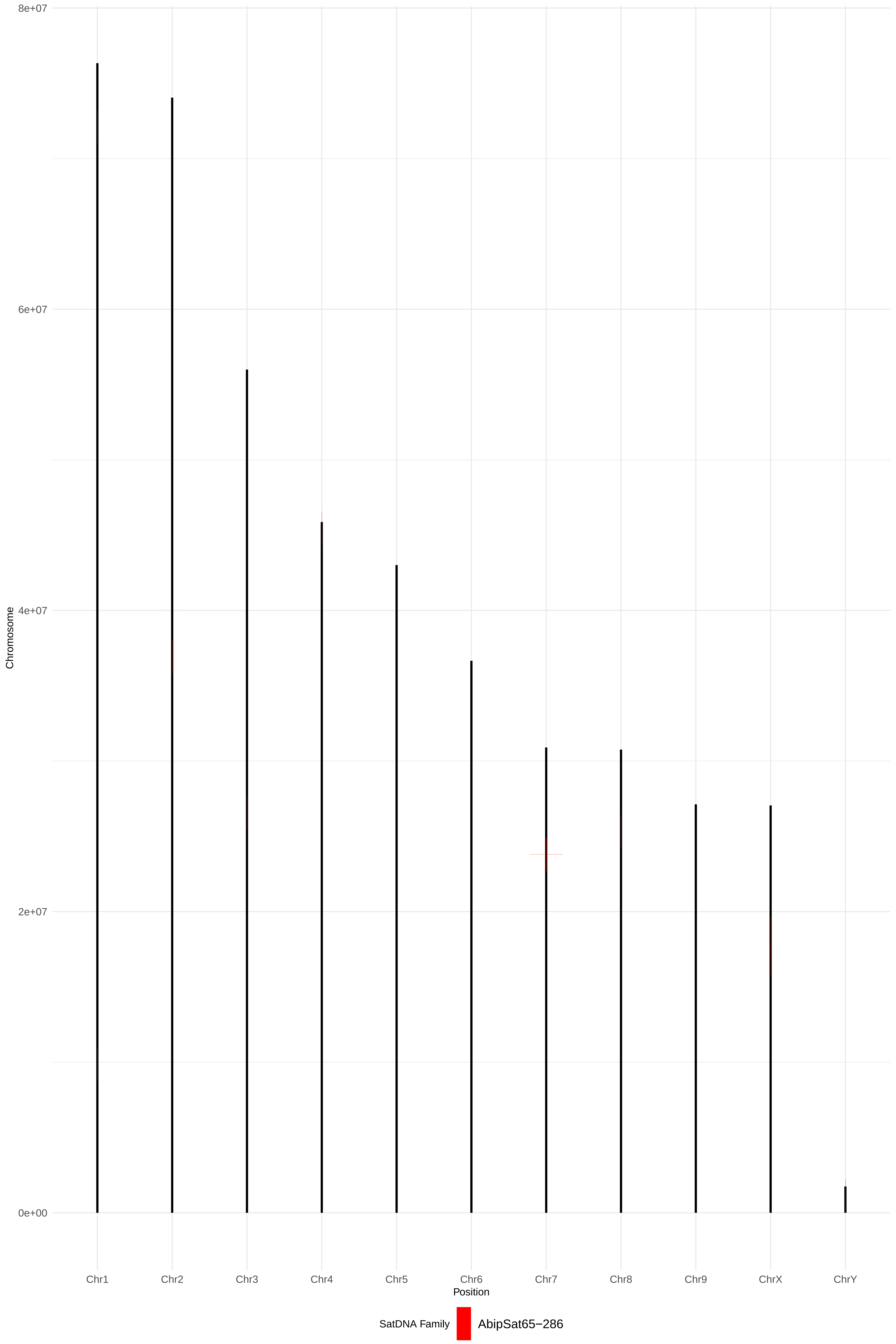

8e+07
6e+07
4e+07
2e+07
0e+00
Chromosome
Chr1
Chr2
Chr3
Chr4
Chr5
Chr6
Chr7
Chr8
Chr9
ChrX
ChrY
Position
SatDNA Family
AbipSat65−286

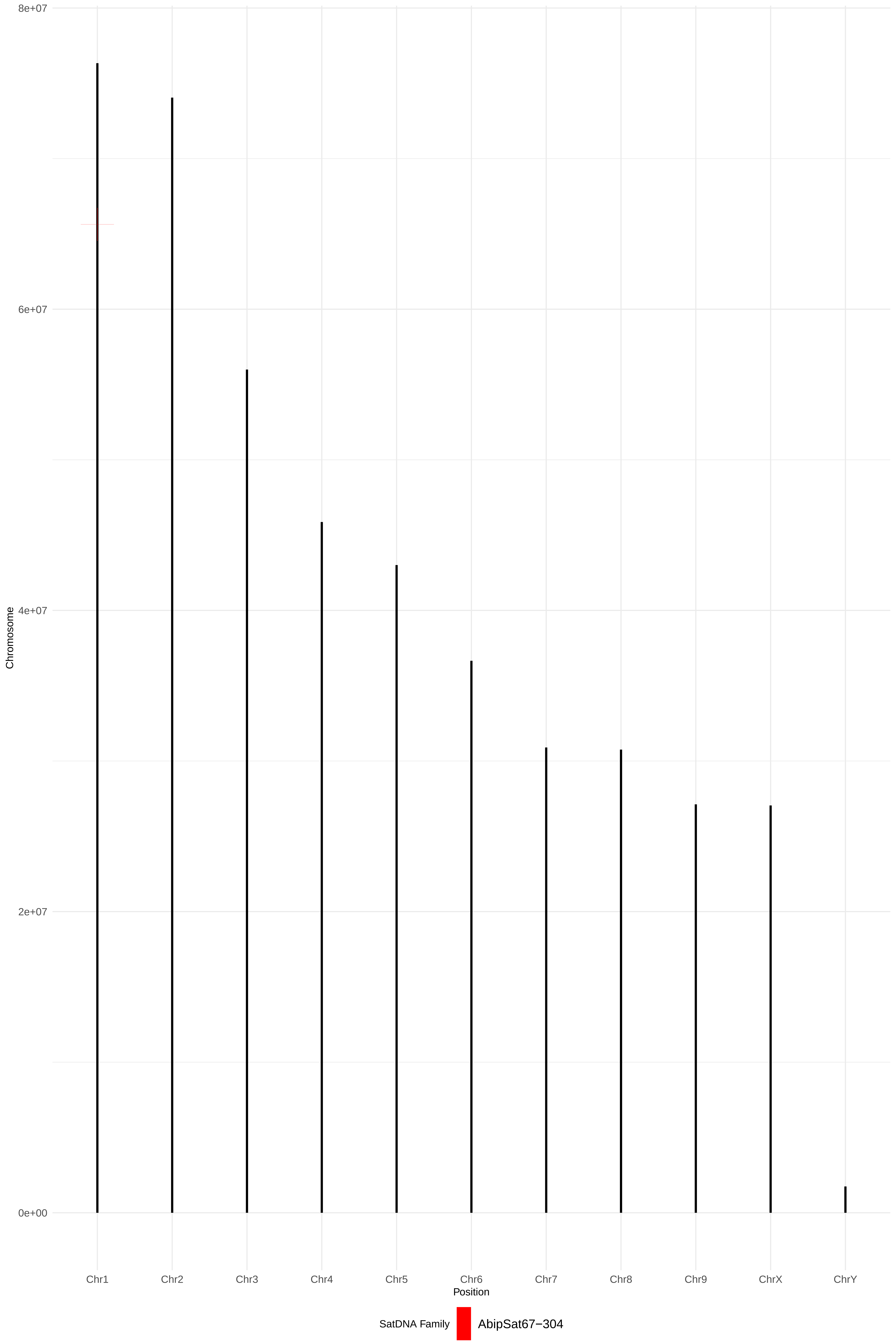
8e+07
6e+07
4e+07
2e+07
0e+00
Chromosome
Chr1
Chr2
Chr3
Chr4
Chr5
Chr6
Chr7
Chr8
Chr9
ChrX
ChrY
Position
SatDNA Family
AbipSat67−304

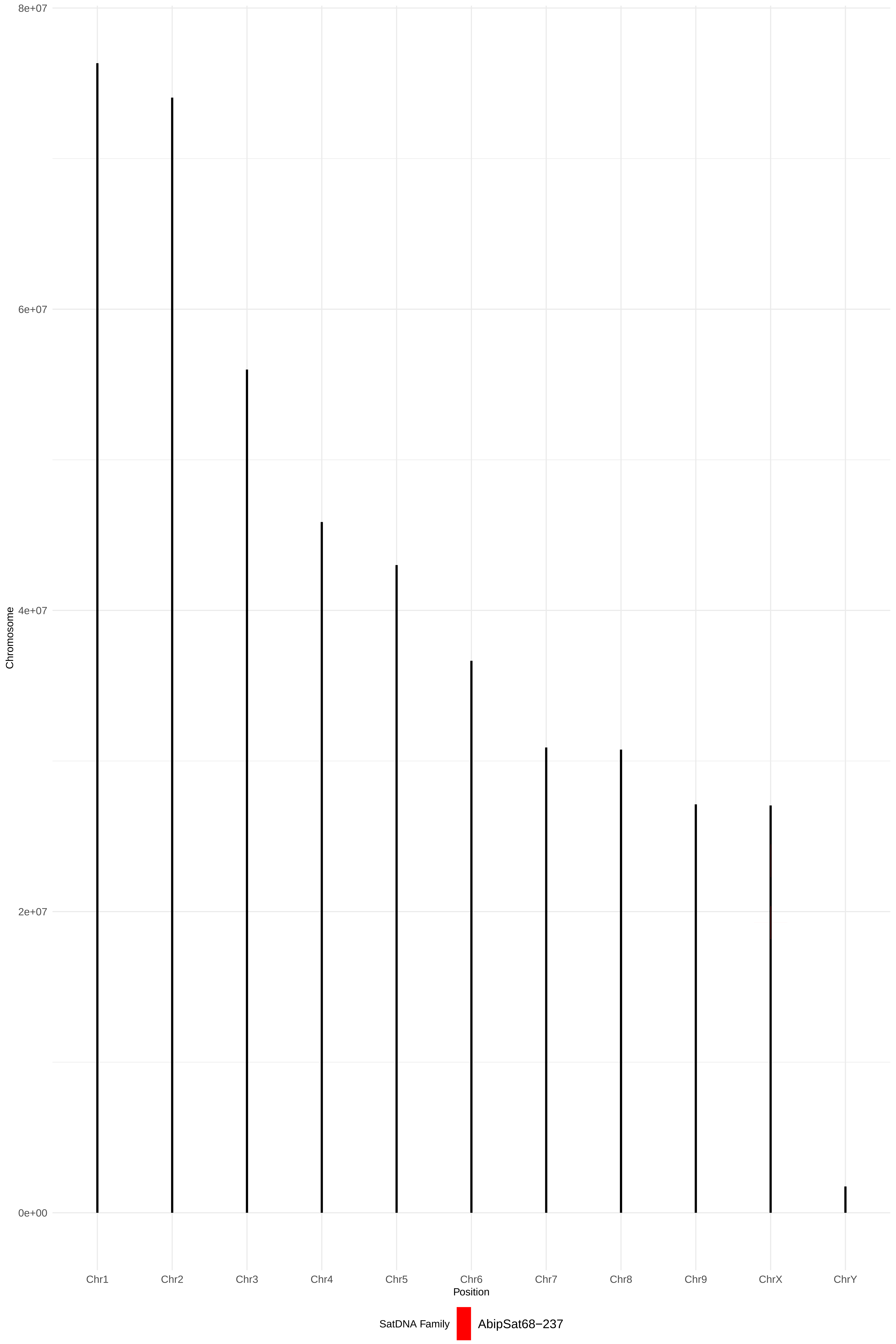

8e+07
6e+07
4e+07
2e+07
0e+00
Chromosome
Chr1
Chr2
Chr3
Chr4
Chr5
Chr6
Chr7
Chr8
Chr9
ChrX
ChrY
Position
SatDNA Family
AbipSat68−237

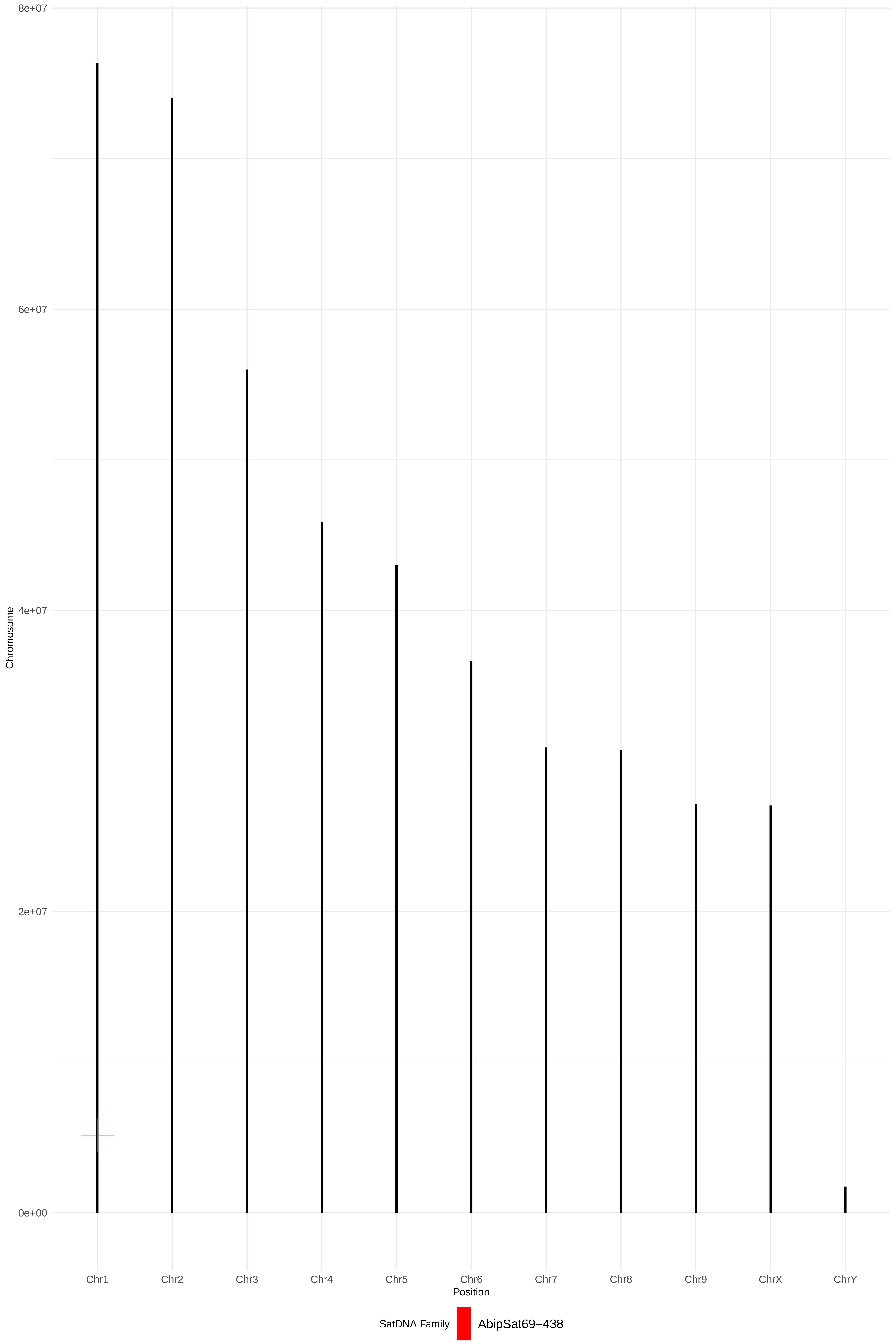

8e+07
6e+07
4e+07
2e+07
0e+00
Chromosome
Chr1
Chr2
Chr3
Chr4
Chr5
Chr6
Chr7
Chr8
Chr9
ChrX
ChrY
Position
SatDNA Family
AbipSat69−438

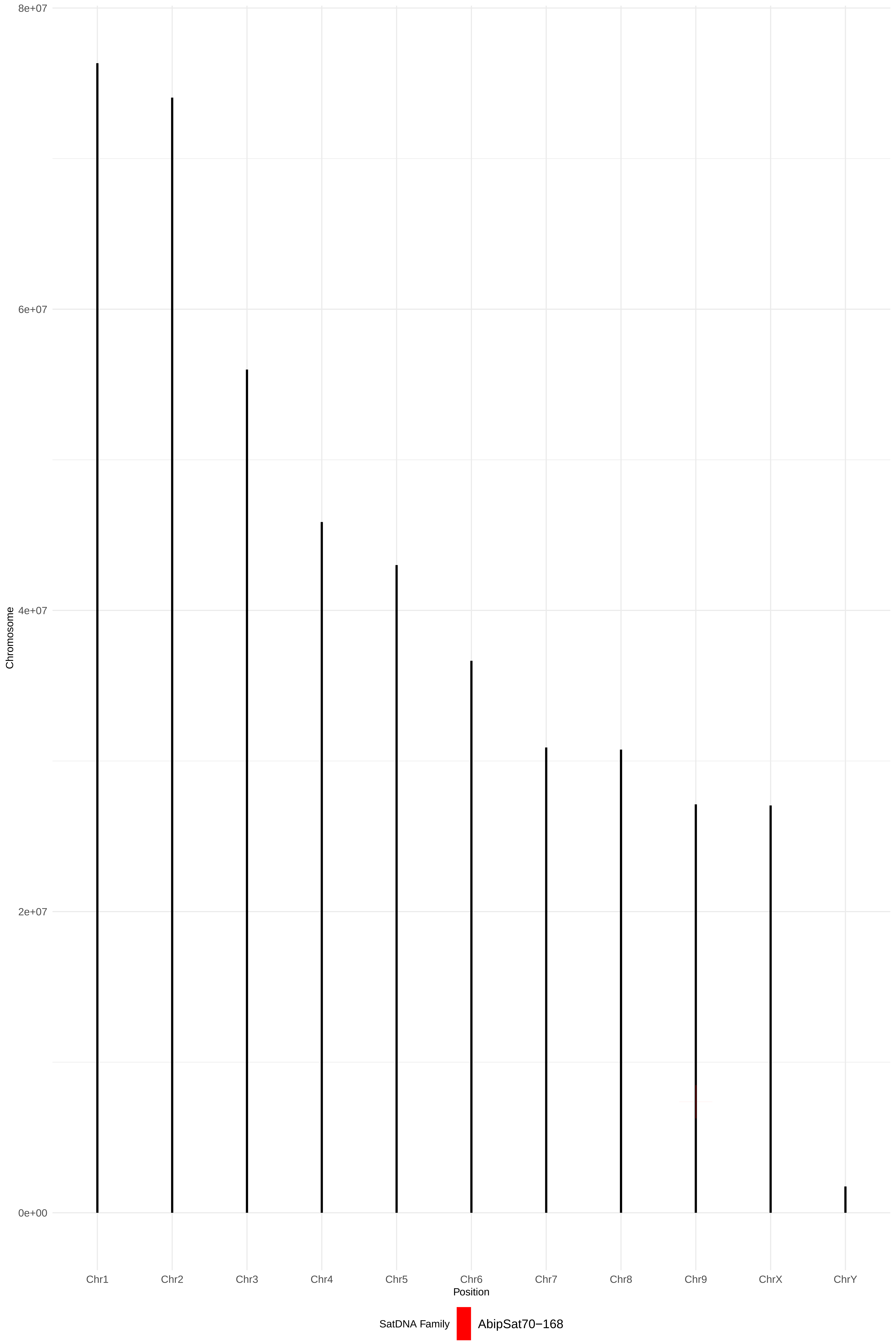

8e+07
6e+07
4e+07
2e+07
0e+00
Chromosome
Chr1
Chr2
Chr3
Chr4
Chr5
Chr6
Chr7
Chr8
Chr9
ChrX
ChrY
Position
SatDNA Family
AbipSat70−168

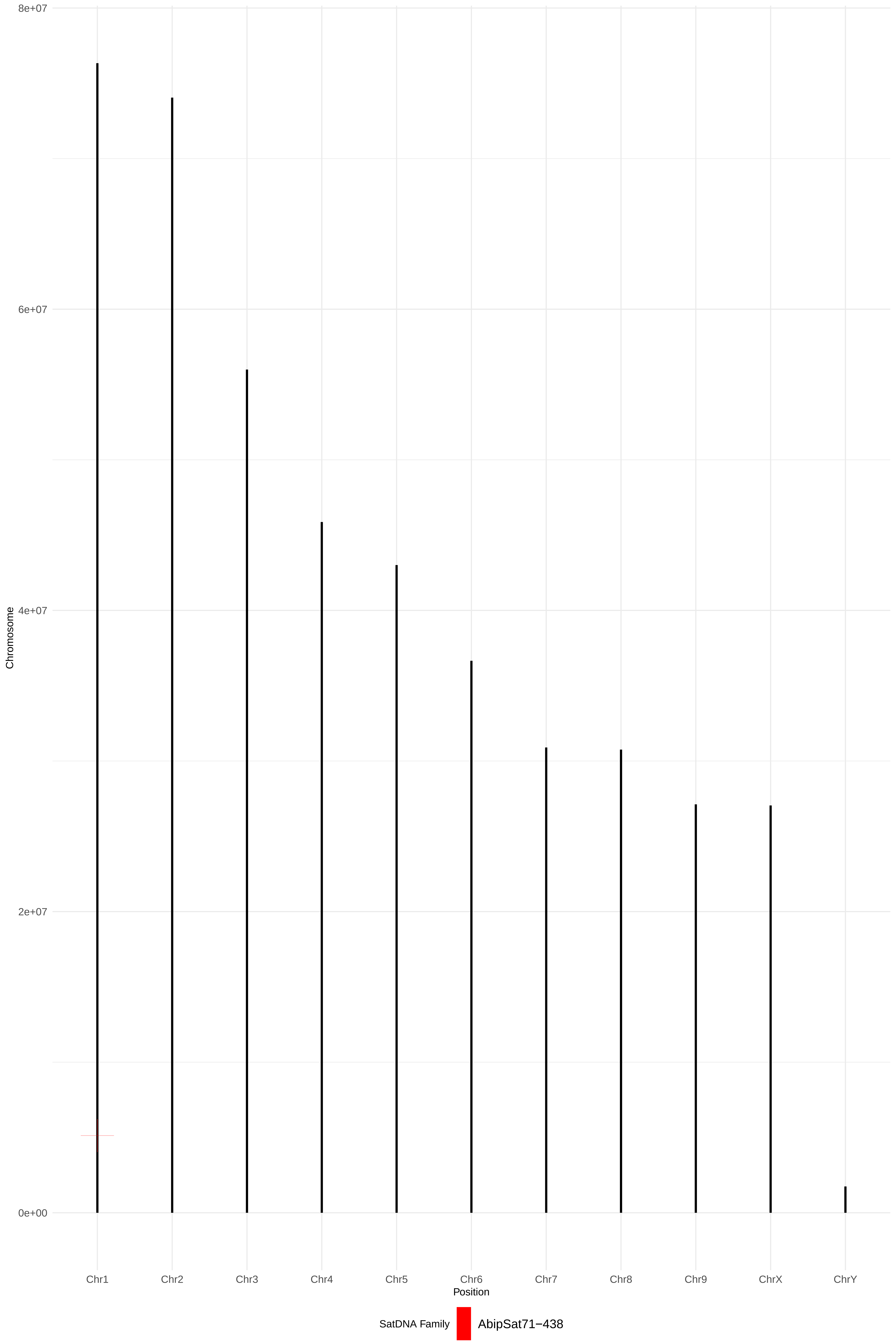
8e+07
6e+07
4e+07
2e+07
0e+00
Chromosome
Chr1
Chr2
Chr3
Chr4
Chr5
Chr6
Chr7
Chr8
Chr9
ChrX
ChrY
Position
SatDNA Family
AbipSat71−438

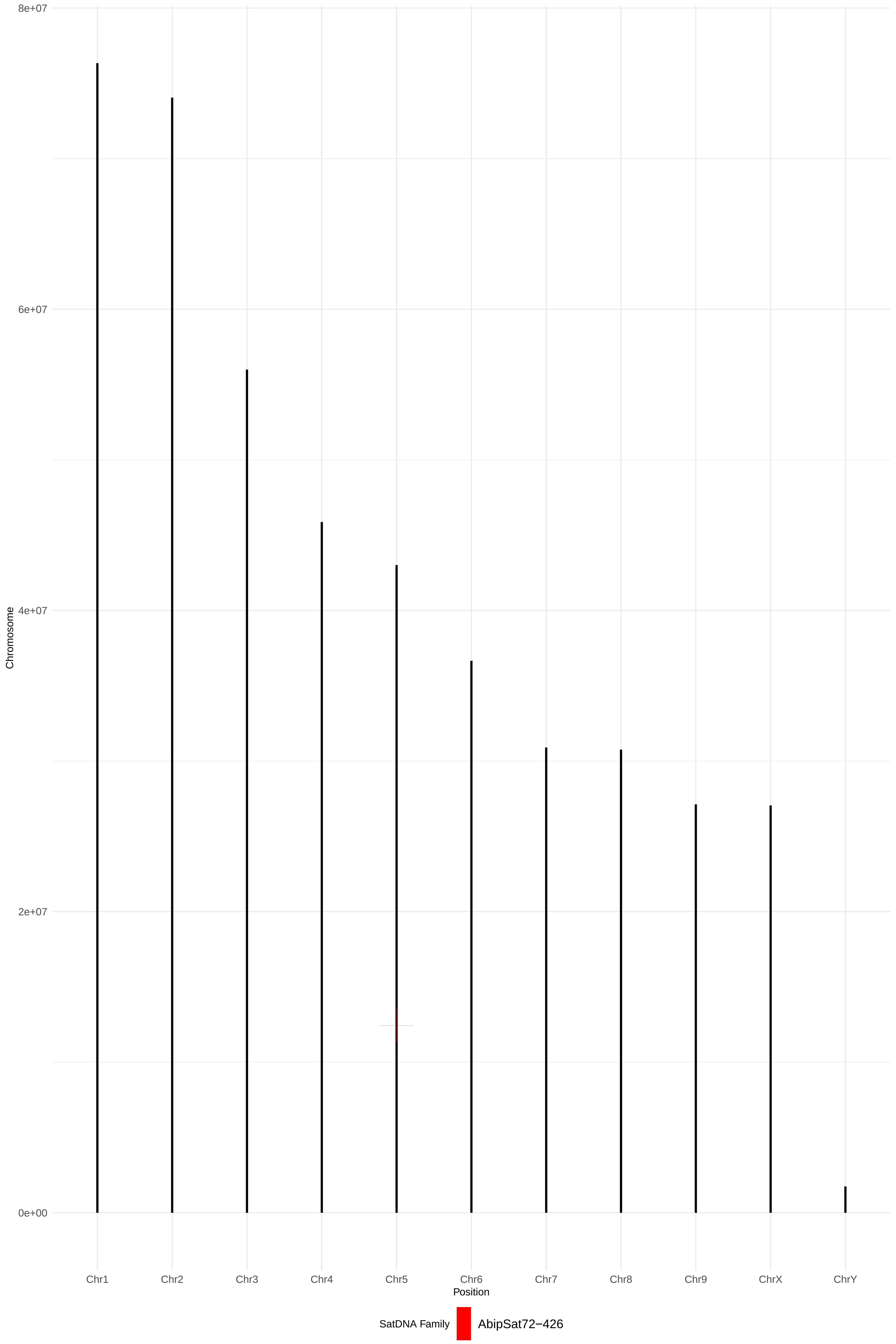

8e+07
6e+07
4e+07
2e+07
0e+00
Chromosome
Chr1
Chr2
Chr3
Chr4
Chr5
Chr6
Chr7
Chr8
Chr9
ChrX
ChrY
Position
SatDNA Family
AbipSat72−426

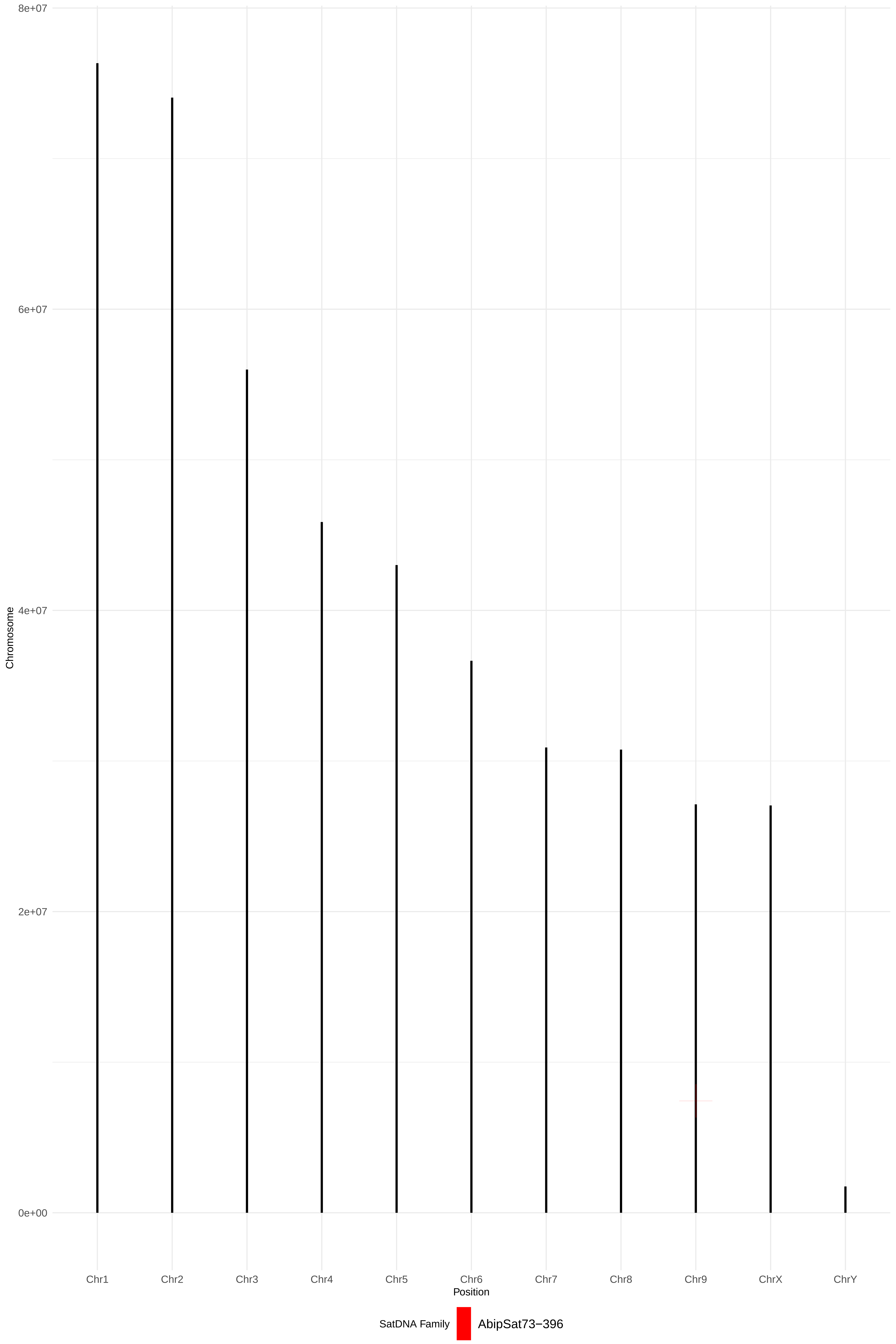
8e+07
6e+07
4e+07
2e+07
0e+00
Chromosome
Chr1
Chr2
Chr3
Chr4
Chr5
Chr6
Chr7
Chr8
Chr9
ChrX
ChrY
Position
SatDNA Family
AbipSat73−396

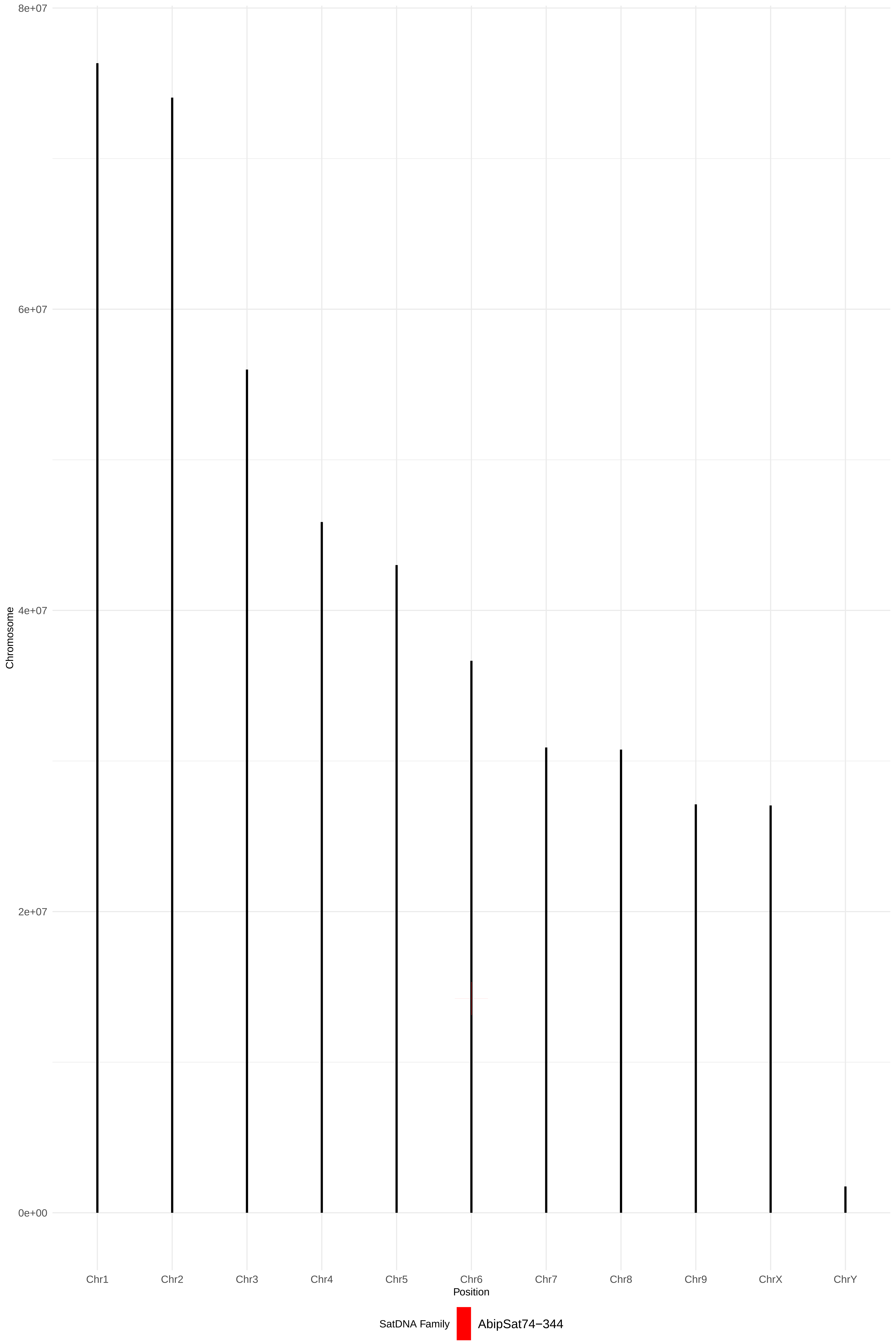
8e+07
6e+07
4e+07
2e+07
0e+00
Chromosome
Chr1
Chr2
Chr3
Chr4
Chr5
Chr6
Chr7
Chr8
Chr9
ChrX
ChrY
Position
SatDNA Family
AbipSat74−344

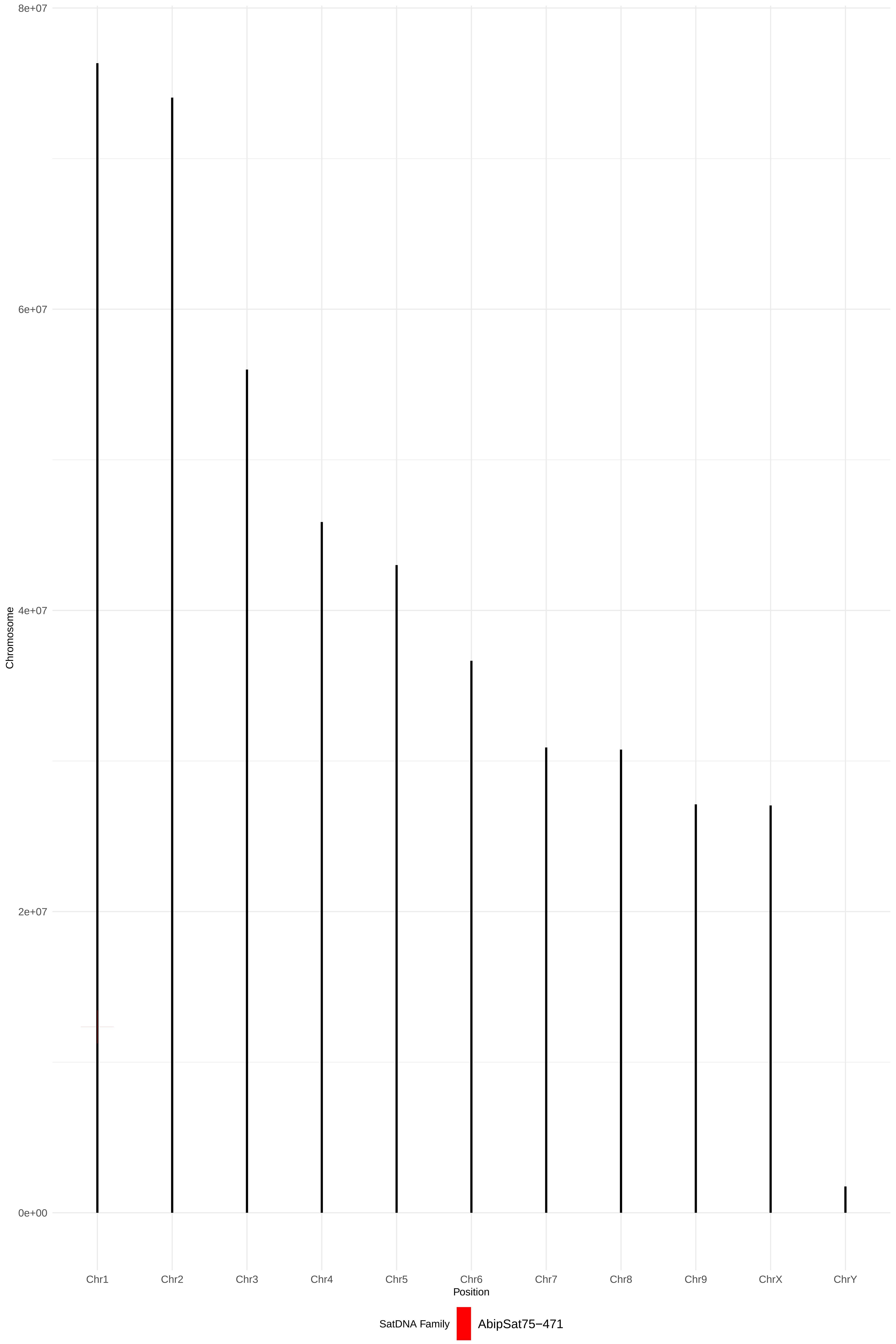

8e+07
6e+07
4e+07
2e+07
0e+00
Chromosome
Chr1
Chr2
Chr3
Chr4
Chr5
Chr6
Chr7
Chr8
Chr9
ChrX
ChrY
Position
SatDNA Family
AbipSat75−471

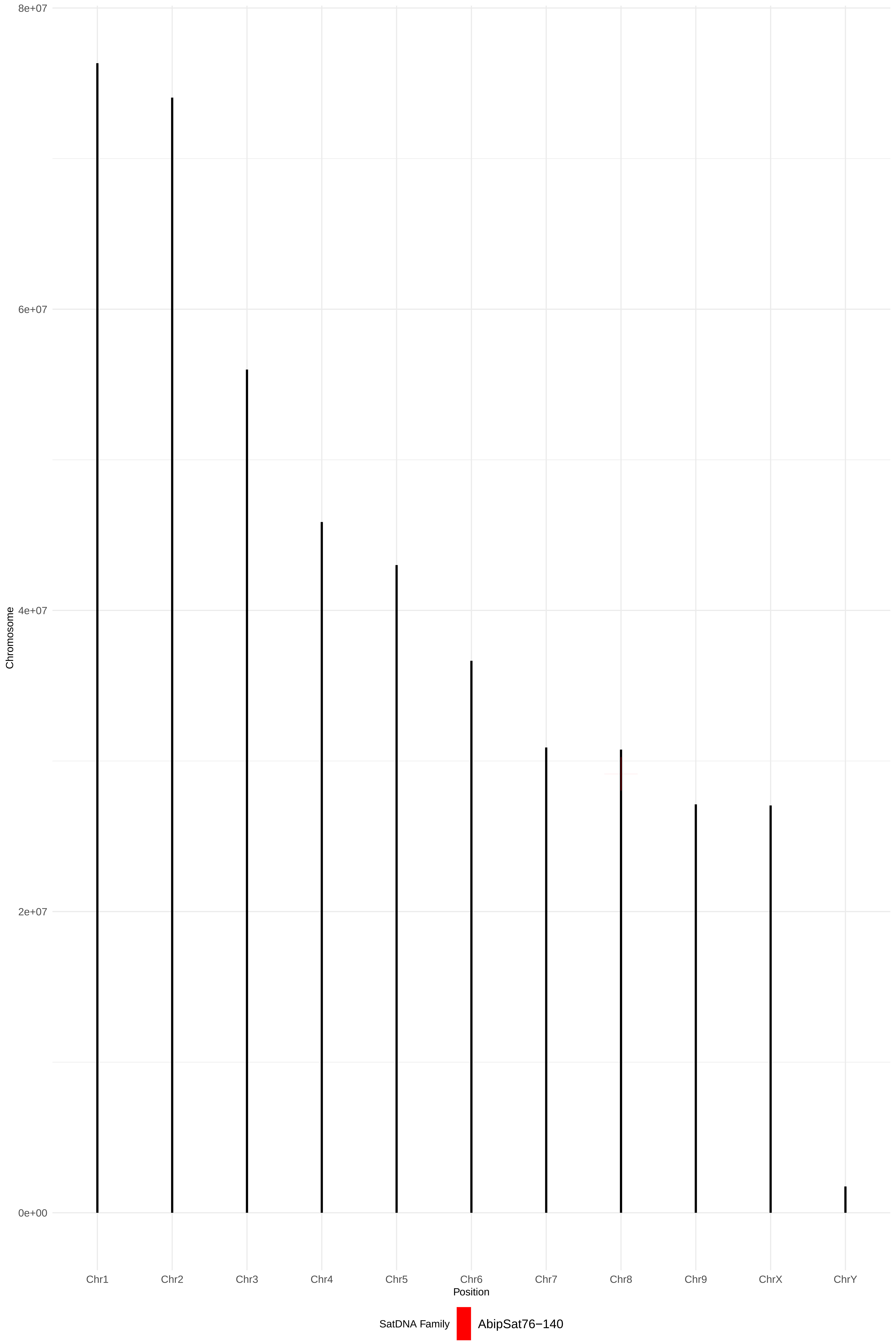

8e+07
6e+07
4e+07
2e+07
0e+00
Chromosome
Chr1
Chr2
Chr3
Chr4
Chr5
Chr6
Chr7
Chr8
Chr9
ChrX
ChrY
Position
SatDNA Family
AbipSat76−140

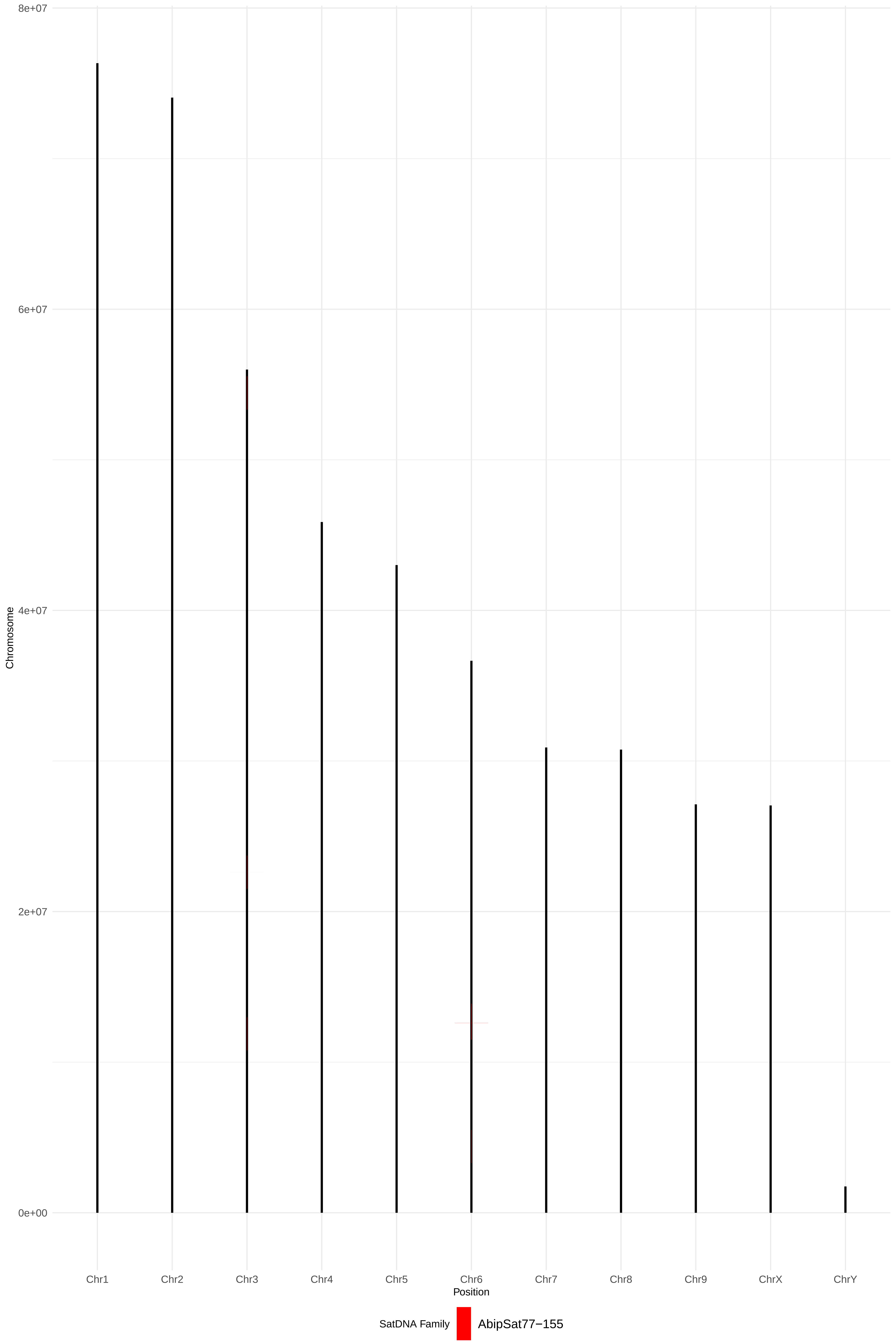

8e+07
6e+07
4e+07
2e+07
0e+00
Chromosome
Chr1
Chr2
Chr3
Chr4
Chr5
Chr6
Chr7
Chr8
Chr9
ChrX
ChrY
Position
SatDNA Family
AbipSat77−155

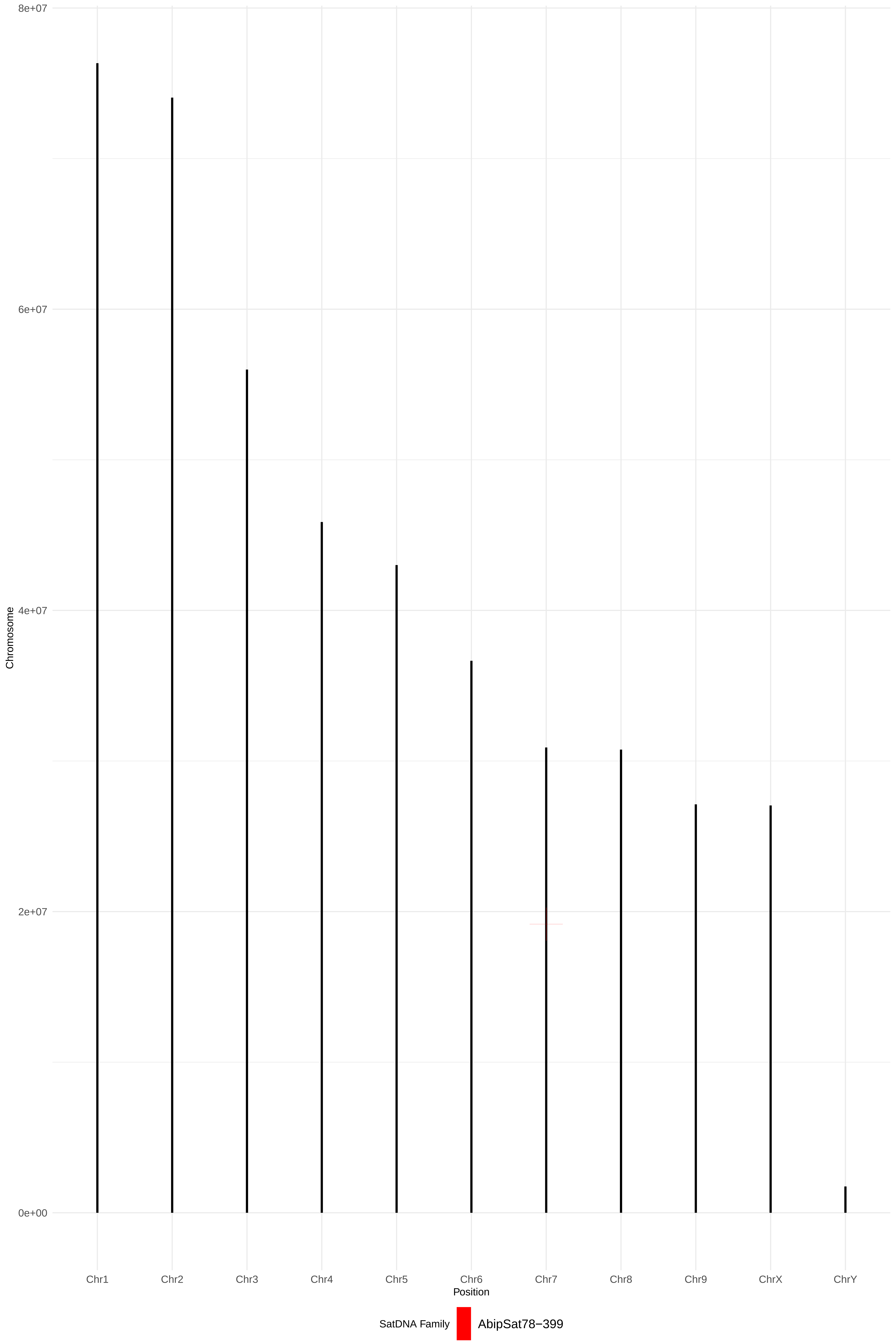

8e+07
6e+07
4e+07
2e+07
0e+00
Chromosome
Chr1
Chr2
Chr3
Chr4
Chr5
Chr6
Chr7
Chr8
Chr9
ChrX
ChrY
Position
SatDNA Family
AbipSat78−399

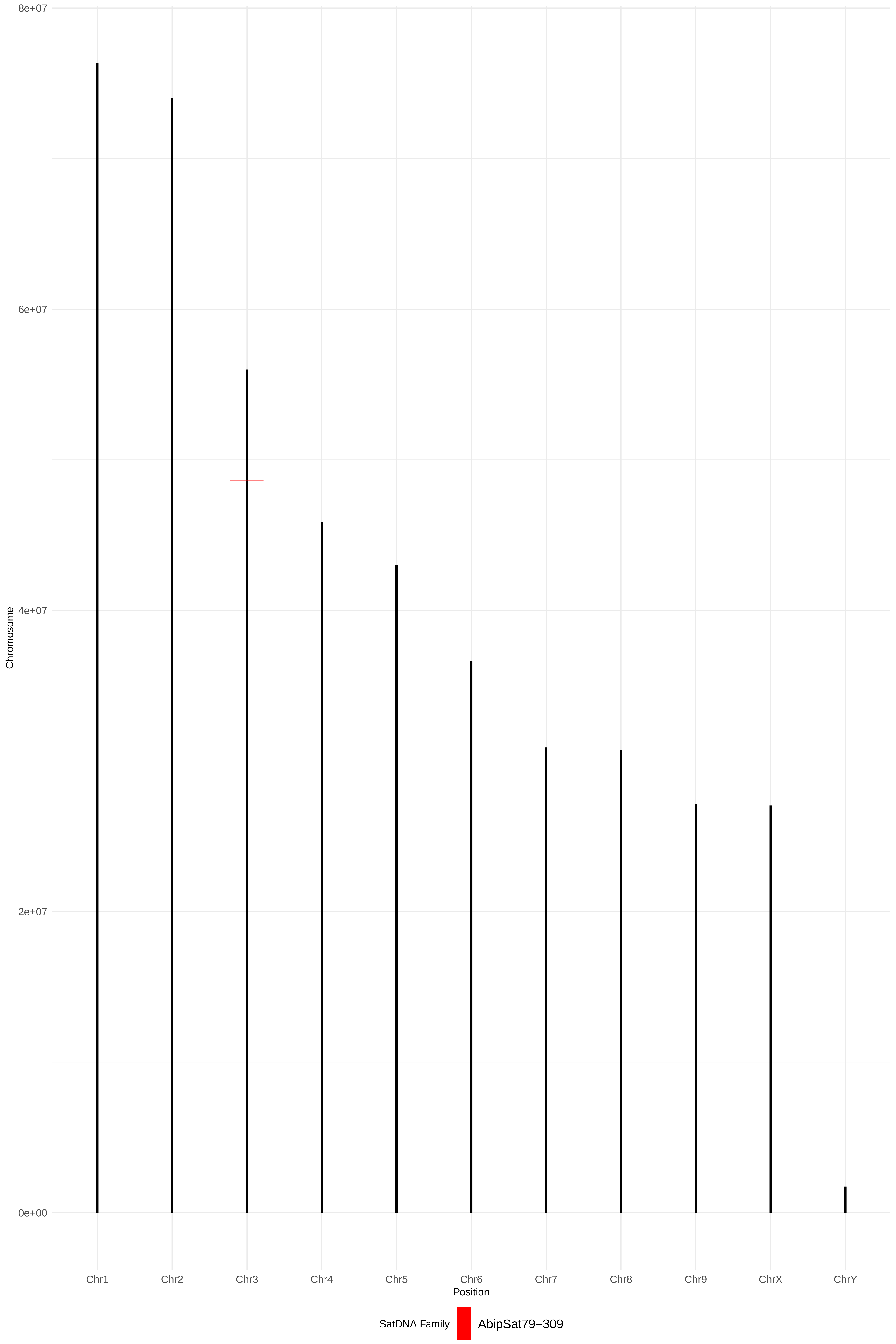

8e+07
6e+07
4e+07
2e+07
0e+00
Chromosome
Chr1
Chr2
Chr3
Chr4
Chr5
Chr6
Chr7
Chr8
Chr9
ChrX
ChrY
Position
SatDNA Family
AbipSat79−309

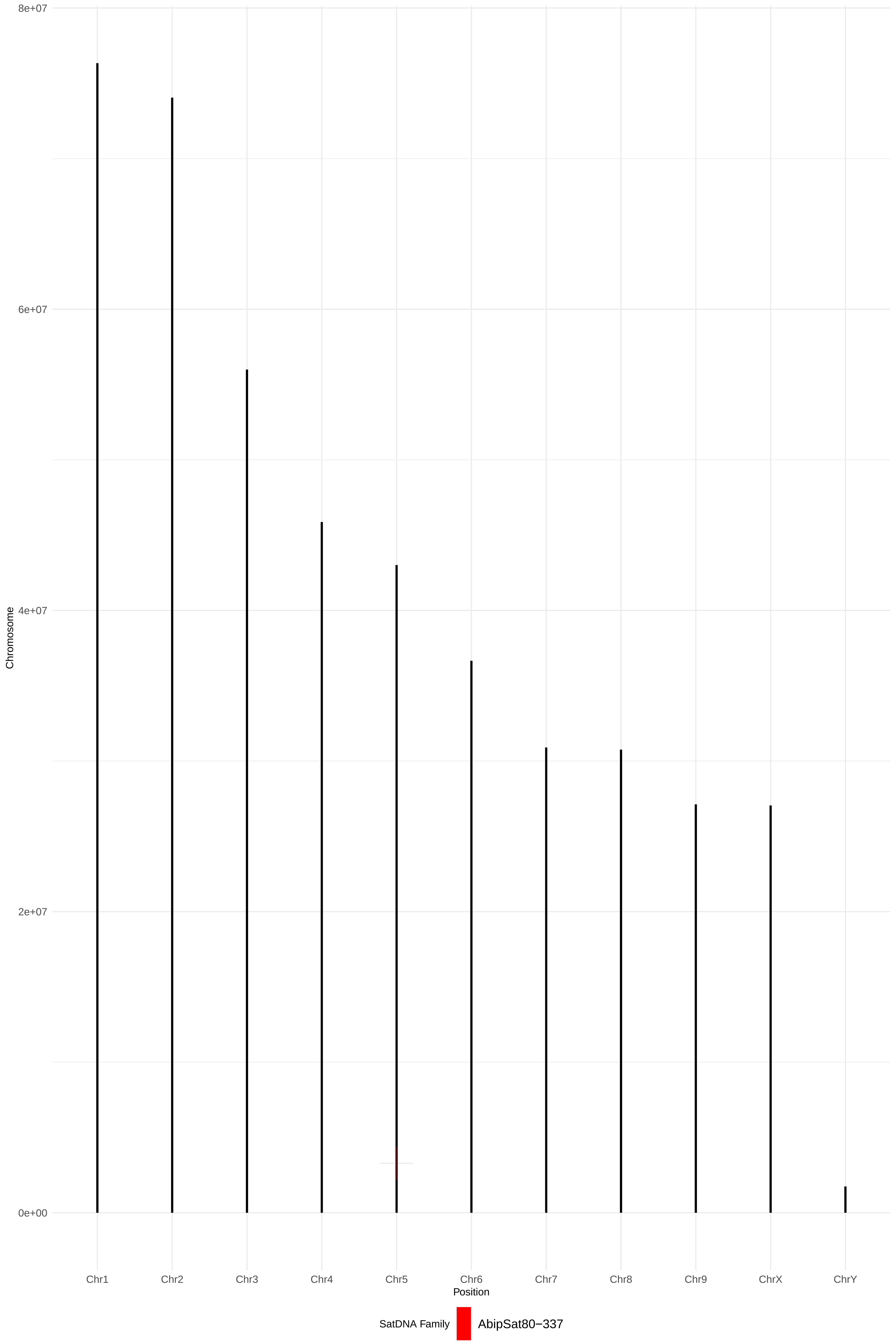

8e+07
6e+07
4e+07
2e+07
0e+00
Chromosome
Chr1
Chr2
Chr3
Chr4
Chr5
Chr6
Chr7
Chr8
Chr9
ChrX
ChrY
Position
SatDNA Family
AbipSat80−337

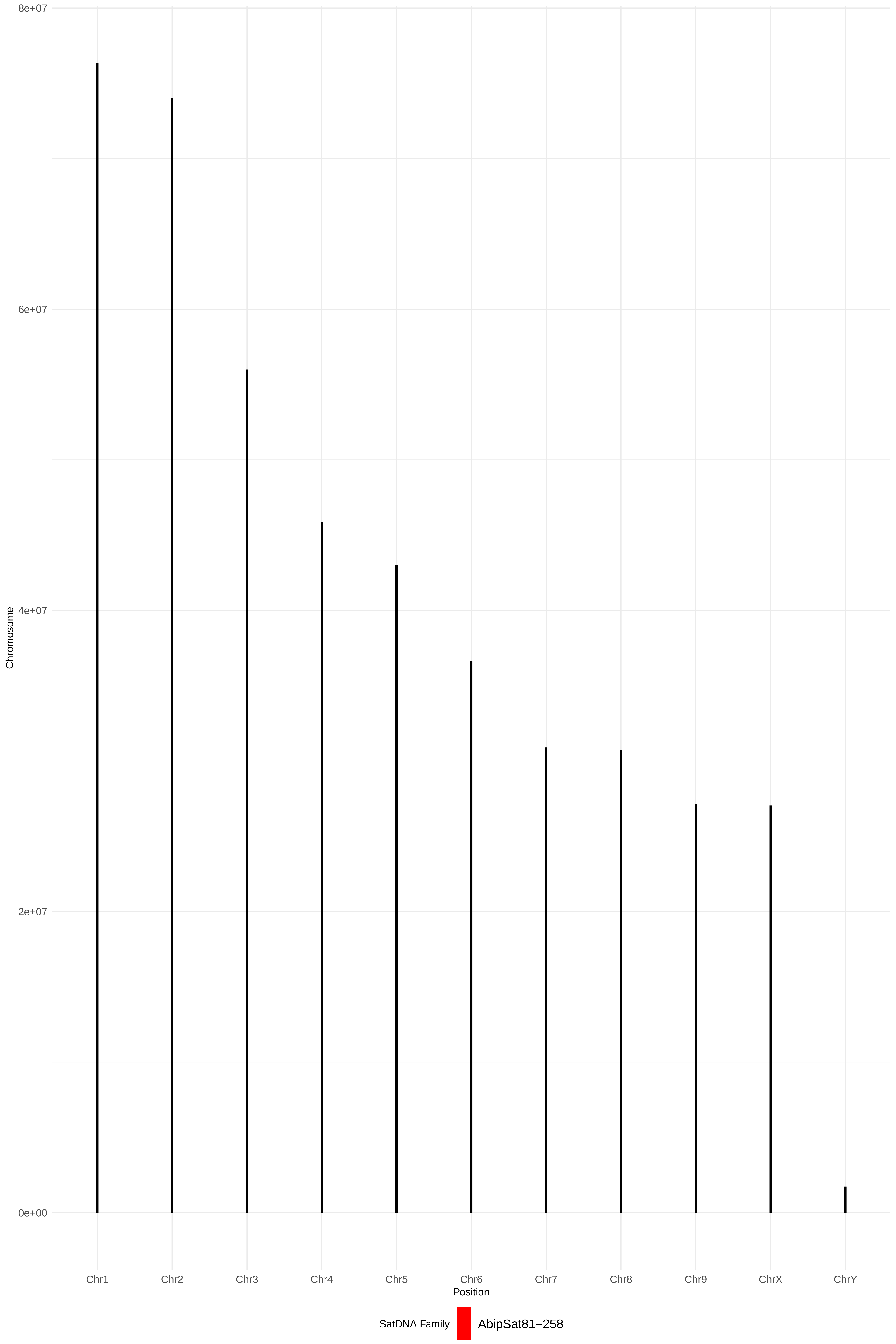

8e+07
6e+07
4e+07
2e+07
0e+00
Chromosome
Chr1
Chr2
Chr3
Chr4
Chr5
Chr6
Chr7
Chr8
Chr9
ChrX
ChrY
Position
SatDNA Family
AbipSat81−258

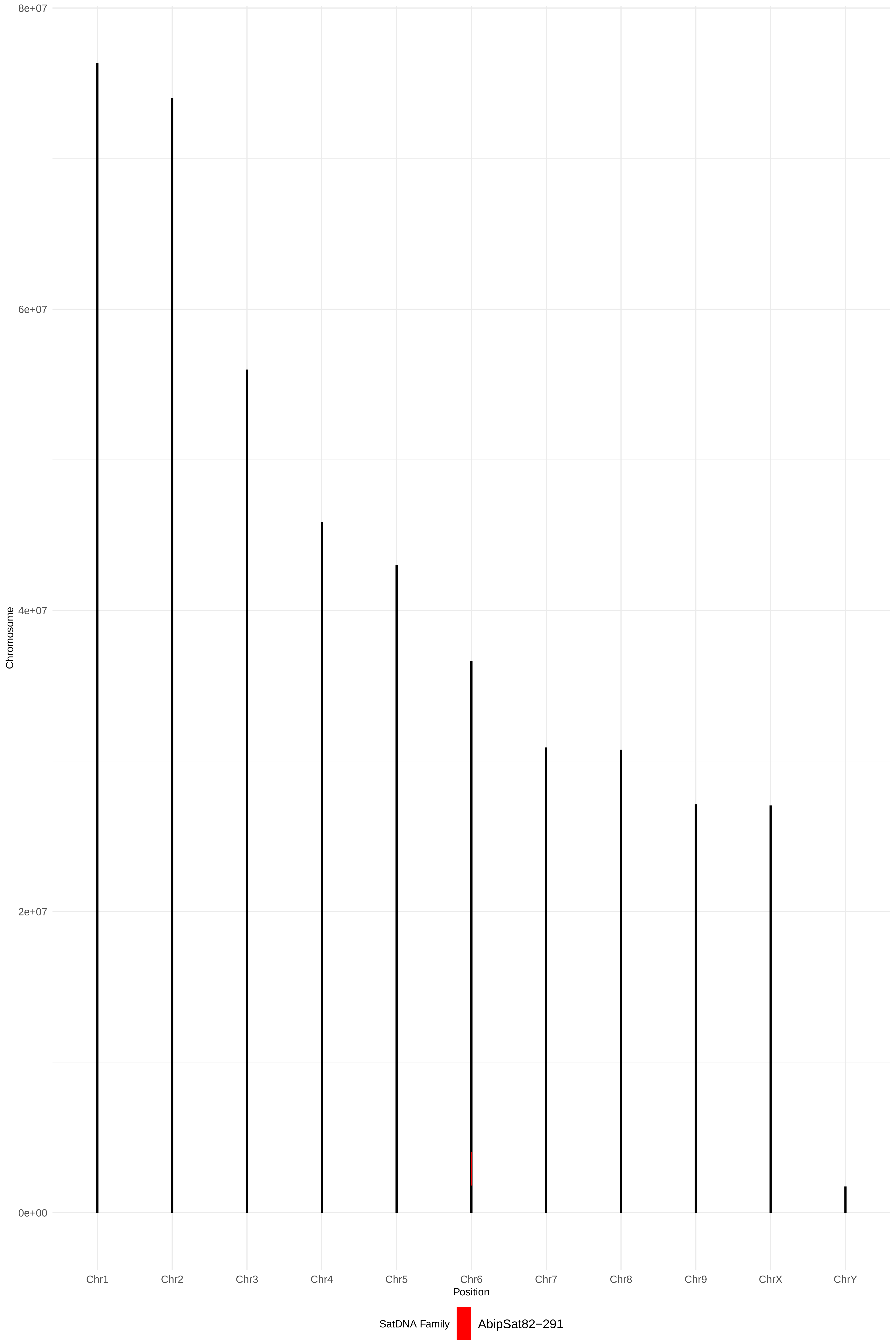

8e+07
6e+07
4e+07
2e+07
0e+00
Chromosome
Chr1
Chr2
Chr3
Chr4
Chr5
Chr6
Chr7
Chr8
Chr9
ChrX
ChrY
Position
SatDNA Family
AbipSat82−291

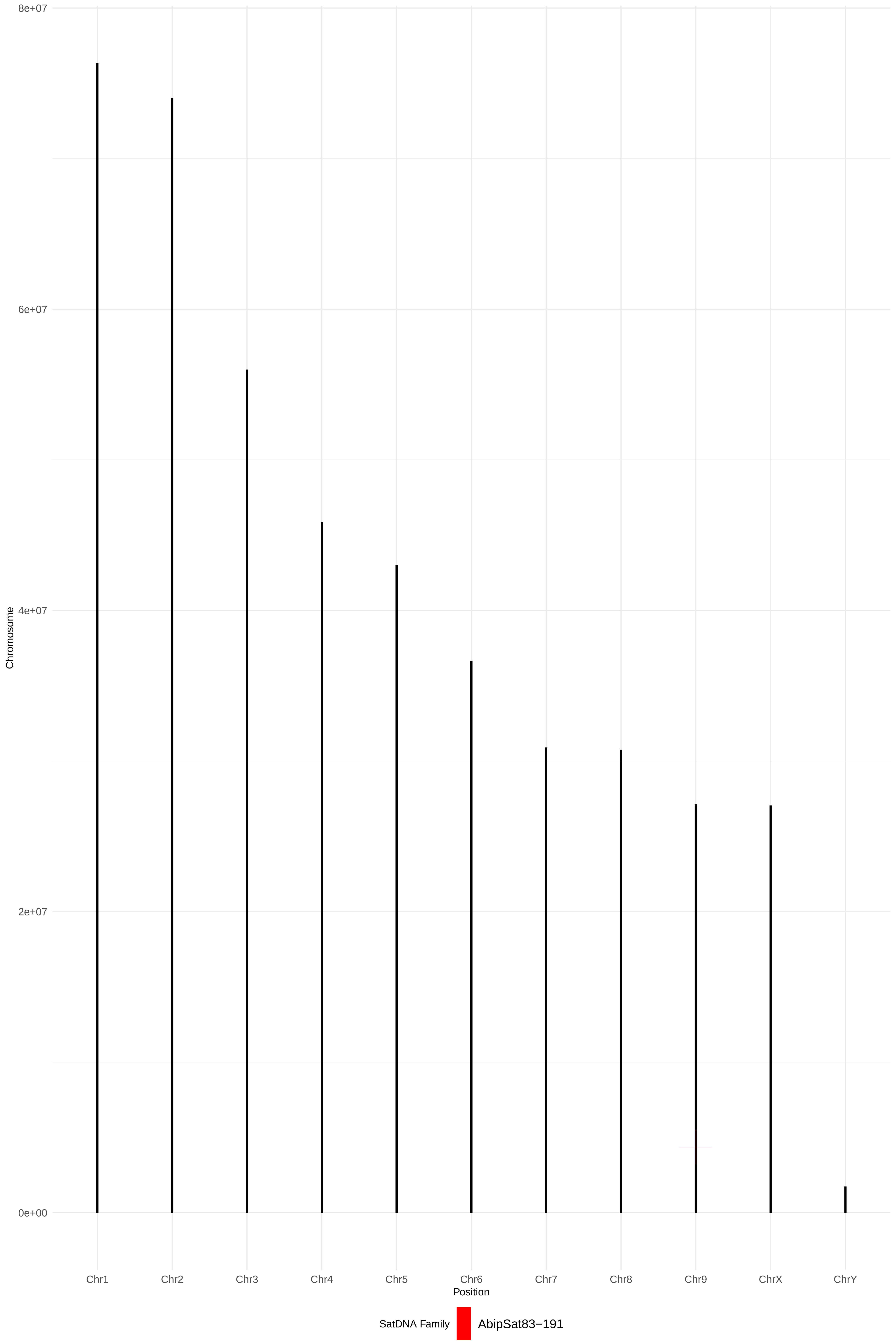
8e+07
6e+07
4e+07
2e+07
0e+00
Chromosome
Chr1
Chr2
Chr3
Chr4
Chr5
Chr6
Chr7
Chr8
Chr9
ChrX
ChrY
Position
SatDNA Family
AbipSat83−191

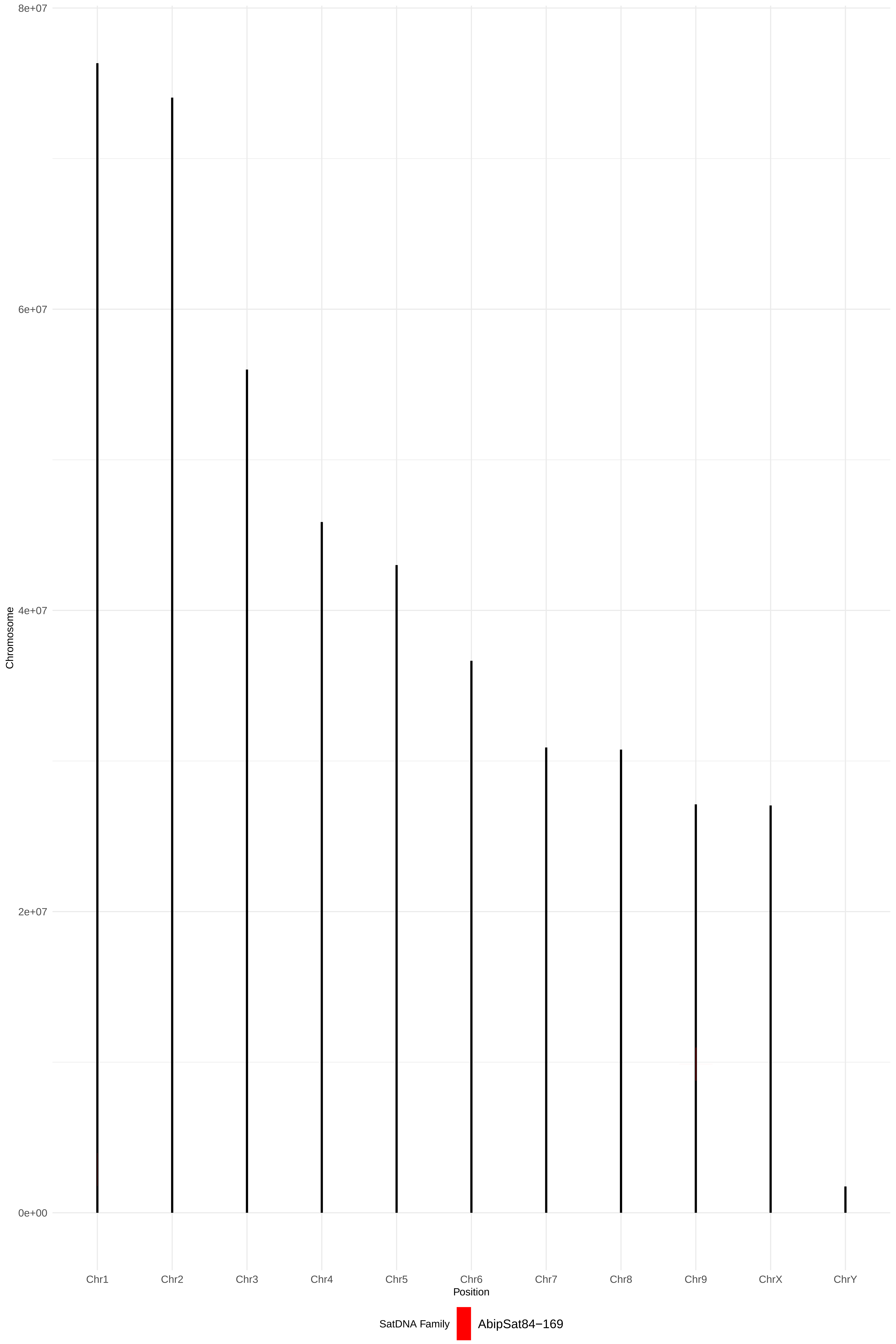
8e+07
6e+07
4e+07
2e+07
0e+00
Chromosome
Chr1
Chr2
Chr3
Chr4
Chr5
Chr6
Chr7
Chr8
Chr9
ChrX
ChrY
Position
SatDNA Family
AbipSat84−169

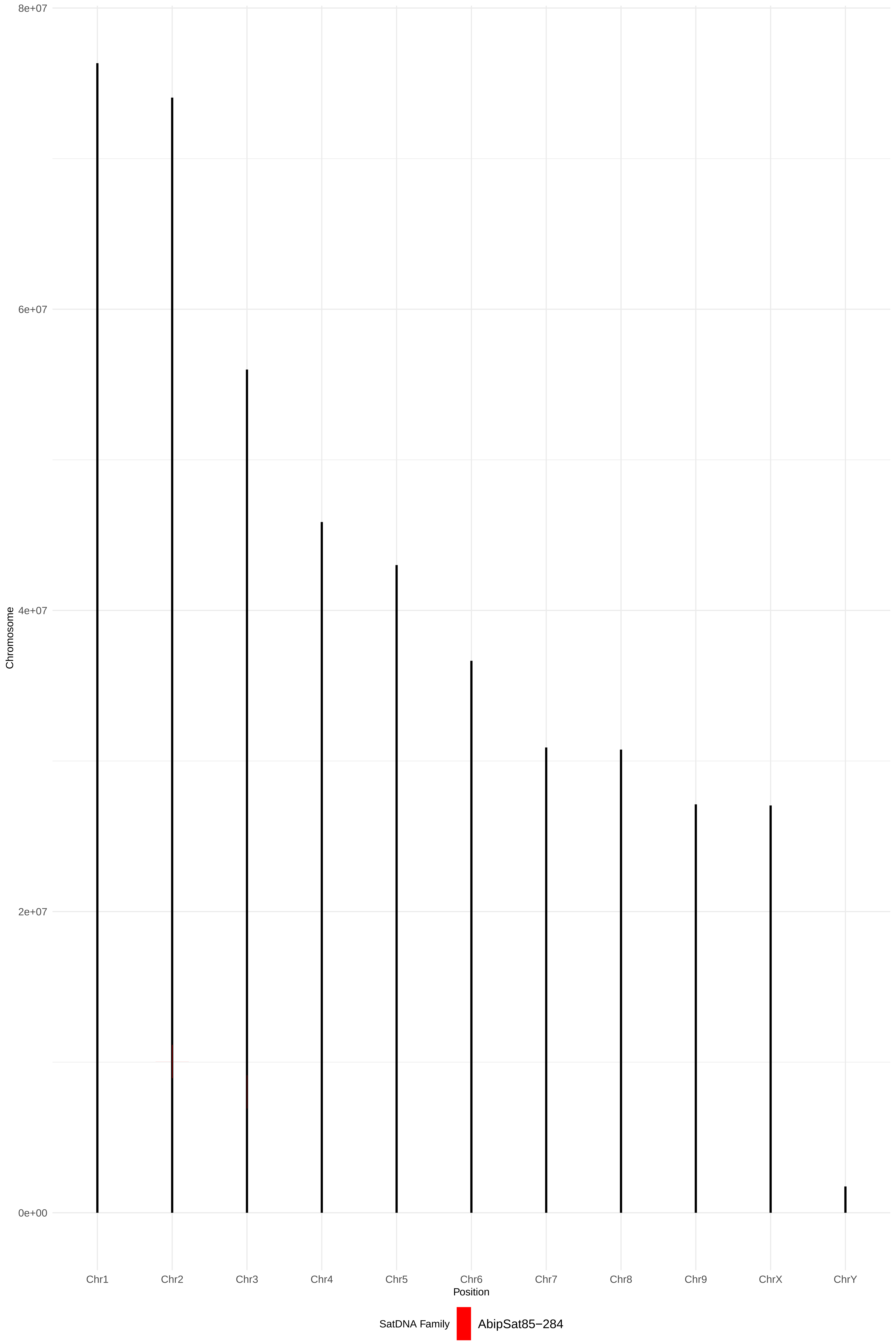
8e+07
6e+07
4e+07
2e+07
0e+00
Chromosome
Chr1
Chr2
Chr3
Chr4
Chr5
Chr6
Chr7
Chr8
Chr9
ChrX
ChrY
Position
SatDNA Family
AbipSat85−284

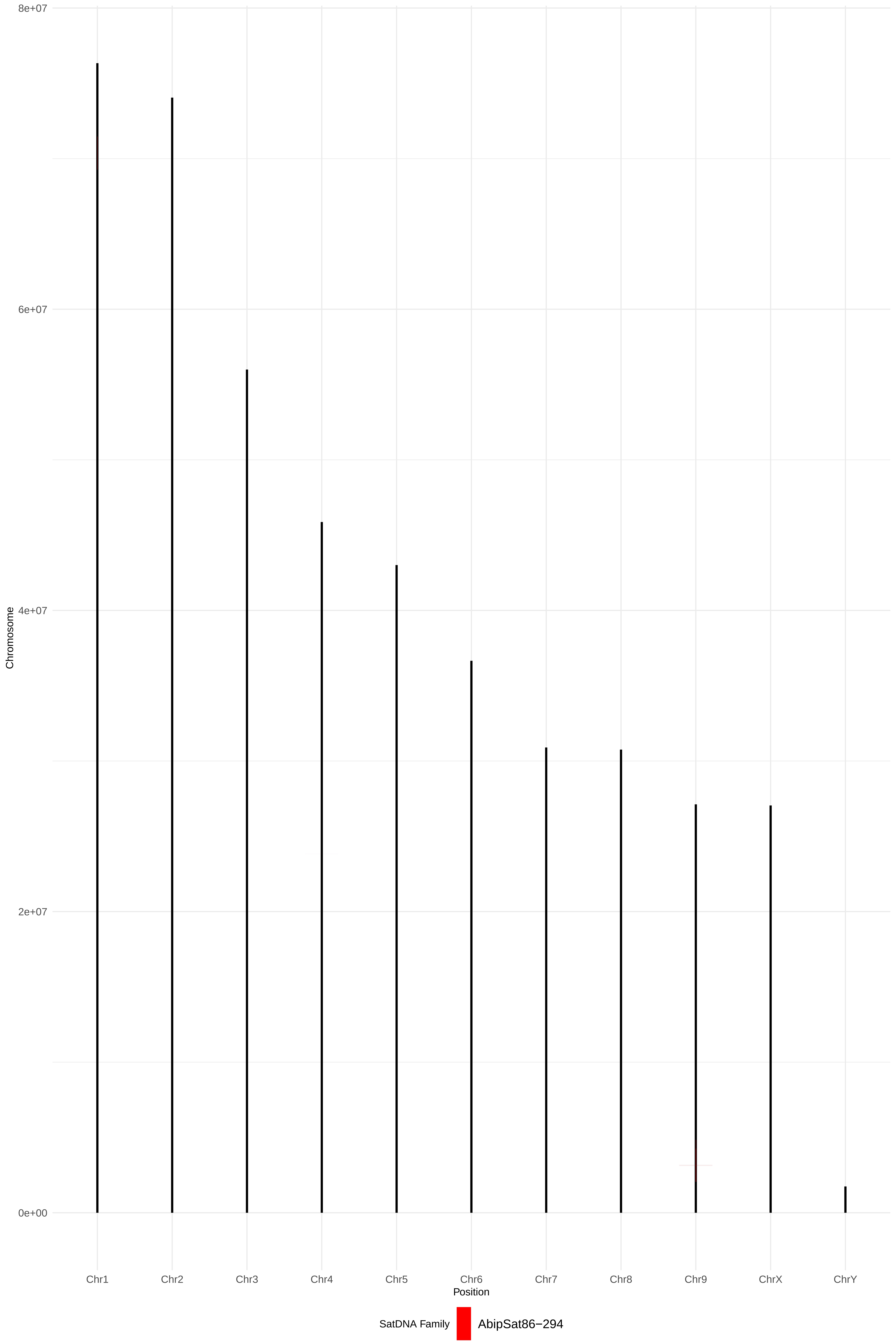

8e+07
6e+07
4e+07
2e+07
0e+00
Chromosome
Chr1
Chr2
Chr3
Chr4
Chr5
Chr6
Chr7
Chr8
Chr9
ChrX
ChrY
Position
SatDNA Family
AbipSat86−294
