# Supplementary material for: Satellitome Analysis of Adalia bipunctata (Coleoptera): Revealing Centromeric Turnover and Potential Chromosome Rearrangements in a Comparative Interspecific Study
Source: Int J Mol Sci. 2024 Aug 25;25(17):9214. doi: 10.3390/ijms25179214 (PMC11394905; doi:10.3390/ijms25179214)
Supplement: Supplementary file 1 [file ijms-25-09214-s001.zip › Supplementary Figure S5 CHRISMAPP scaffolds.pdf]

Figure S5: Location of the satDNAs of *Adalia bipunctata* in the 103 unplaced scaffolds of its genome assembly using CHRISMAPP

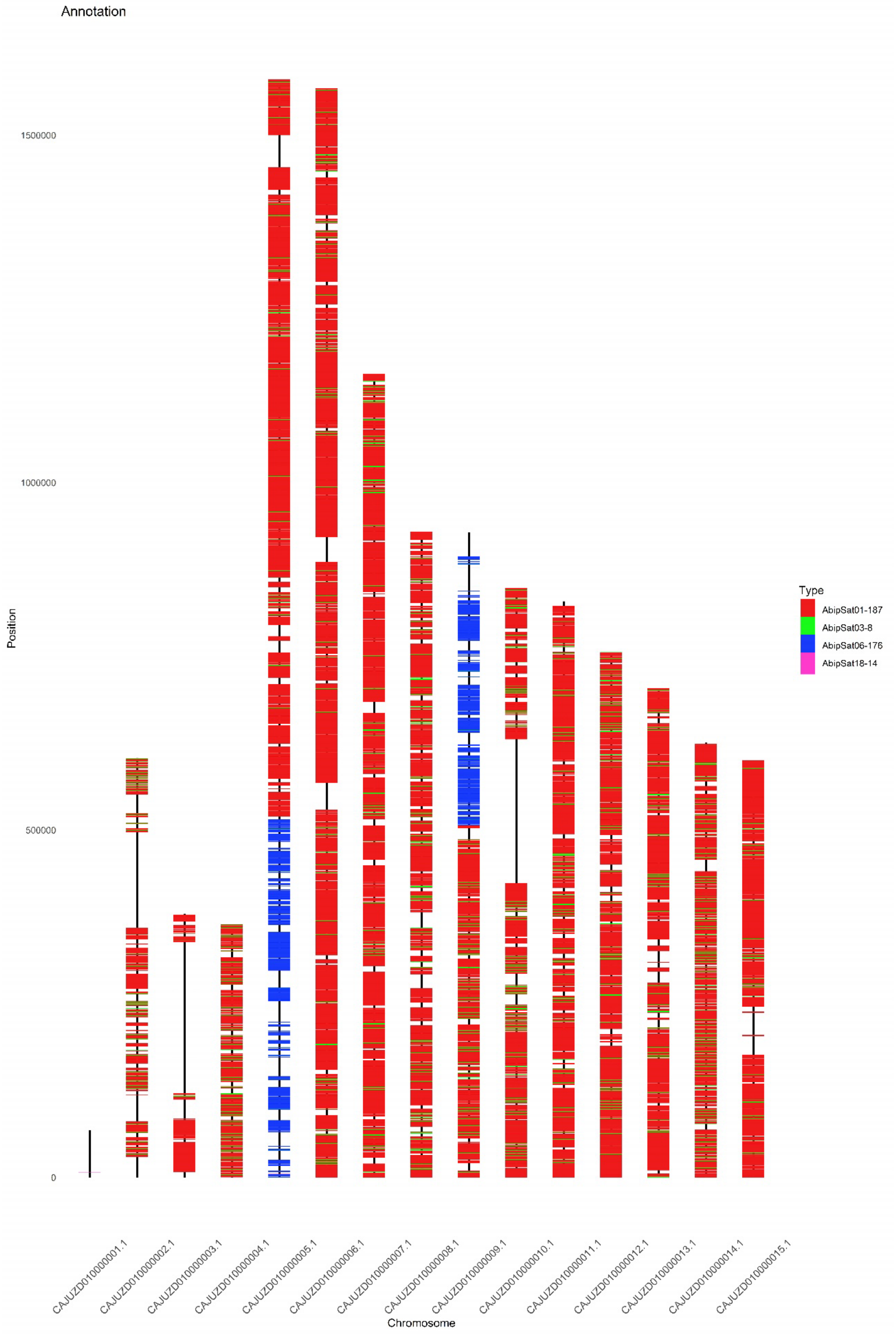

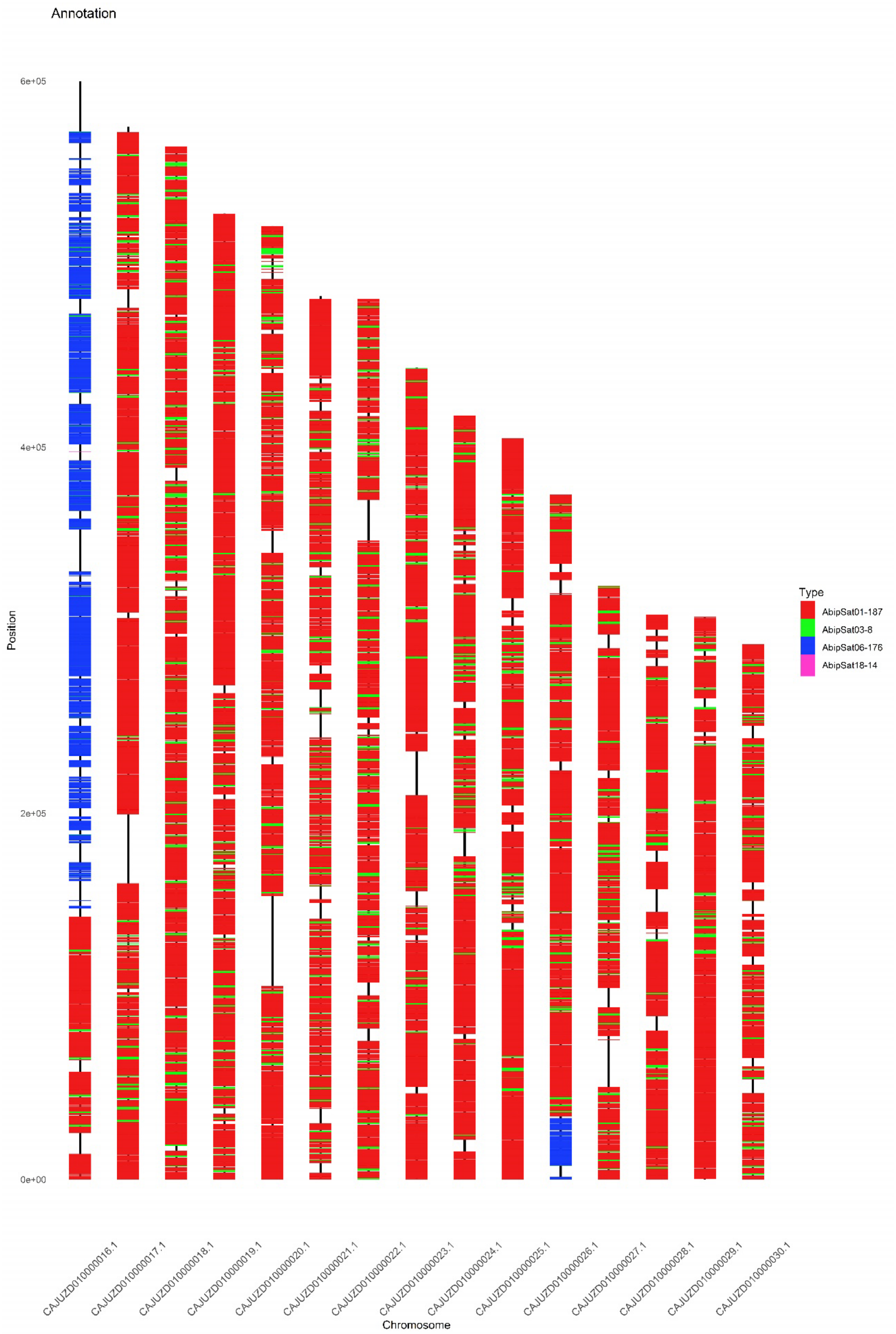
Annotation
6e+05
4e+05
2e+05
0e+00
Position
Type
AbipSat01-187
AbipSat03-8
AbipSat06-176
AbipSat18-14
CAJUZD010000016.1
CAJUZD010000017.1
CAJUZD010000018.1
CAJUZD010000019.1
CAJUZD010000020.1
CAJUZD010000021.1
CAJUZD010000022.1
CAJUZD010000023.1
CAJUZD010000024.1
CAJUZD010000025.1
CAJUZD010000026.1
CAJUZD010000027.1
CAJUZD010000028.1
CAJUZD010000029.1
CAJUZD010000030.1
Chromosome

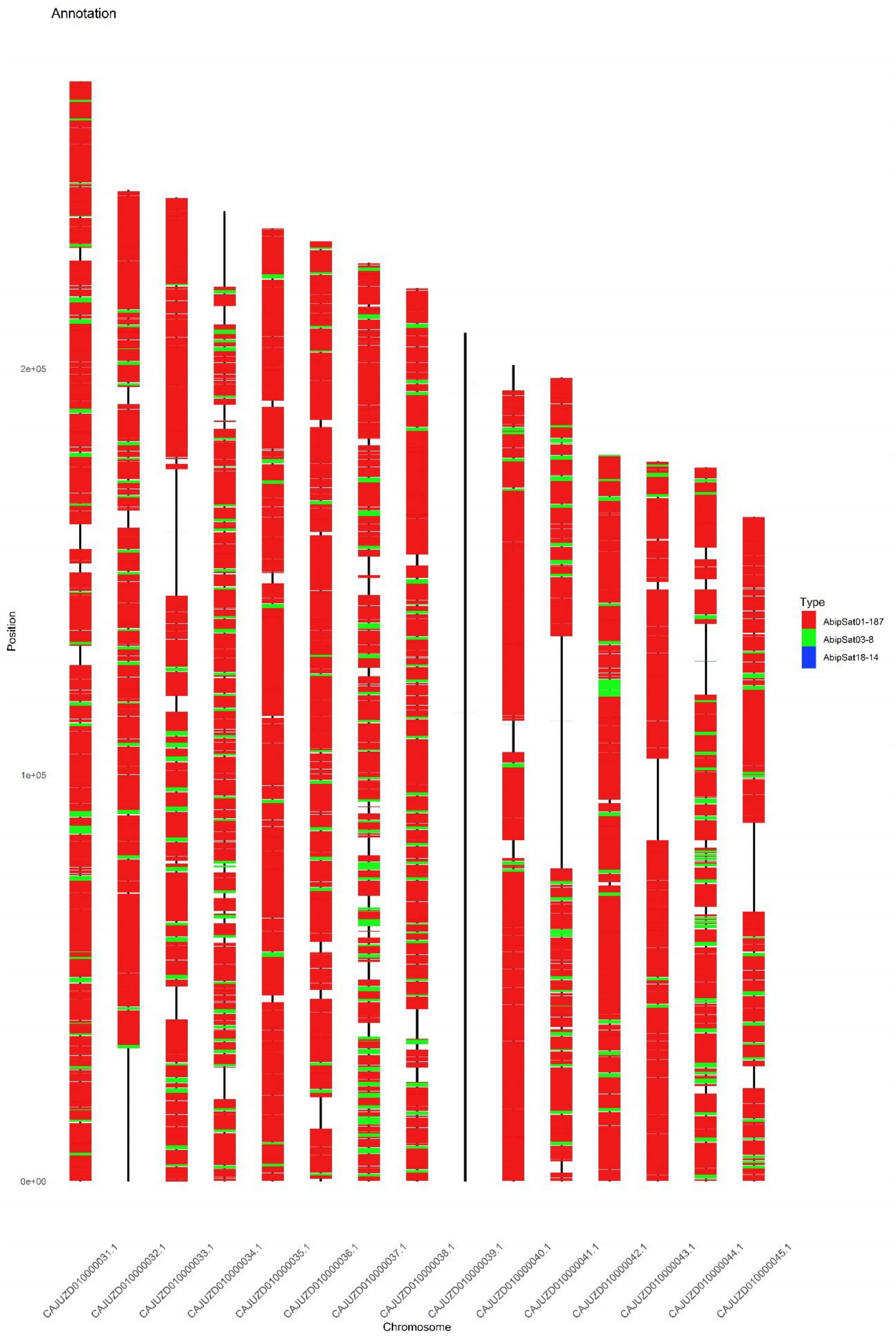
Annotation
Position
2e+05
1e+05
0e+00
CAJUZD010000031.1
CAJUZD010000032.1
CAJUZD010000033.1
CAJUZD010000034.1
CAJUZD010000035.1
CAJUZD010000036.1
CAJUZD010000037.1
CAJUZD010000038.1
CAJUZD010000039.1
CAJUZD010000040.1
CAJUZD010000041.1
CAJUZD010000042.1
CAJUZD010000043.1
CAJUZD010000044.1
CAJUZD010000045.1
Chromosome
Type
AbipSat01-187
AbipSat03-8
AbipSat18-14

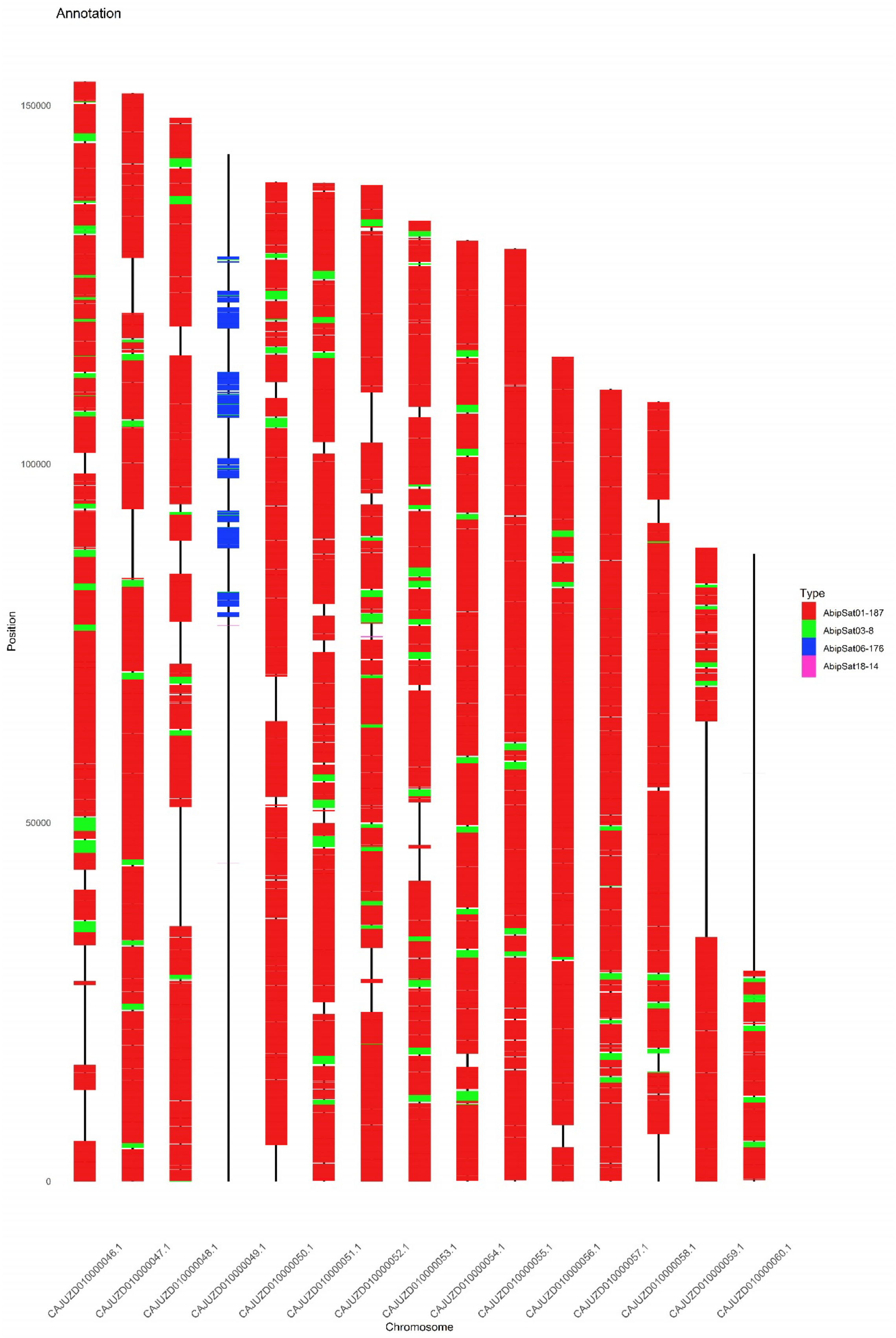

Annotation
150000
100000
50000
0
Position
CAJUZD010000046.1
CAJUZD010000047.1
CAJUZD010000048.1
CAJUZD010000049.1
CAJUZD010000050.1
CAJUZD010000051.1
CAJUZD010000052.1
CAJUZD010000053.1
CAJUZD010000054.1
CAJUZD010000055.1
CAJUZD010000056.1
CAJUZD010000057.1
CAJUZD010000058.1
CAJUZD010000059.1
CAJUZD010000060.1
Chromosome
Type
AbipSat01-187
AbipSat03-8
AbipSat06-176
AbipSat18-14

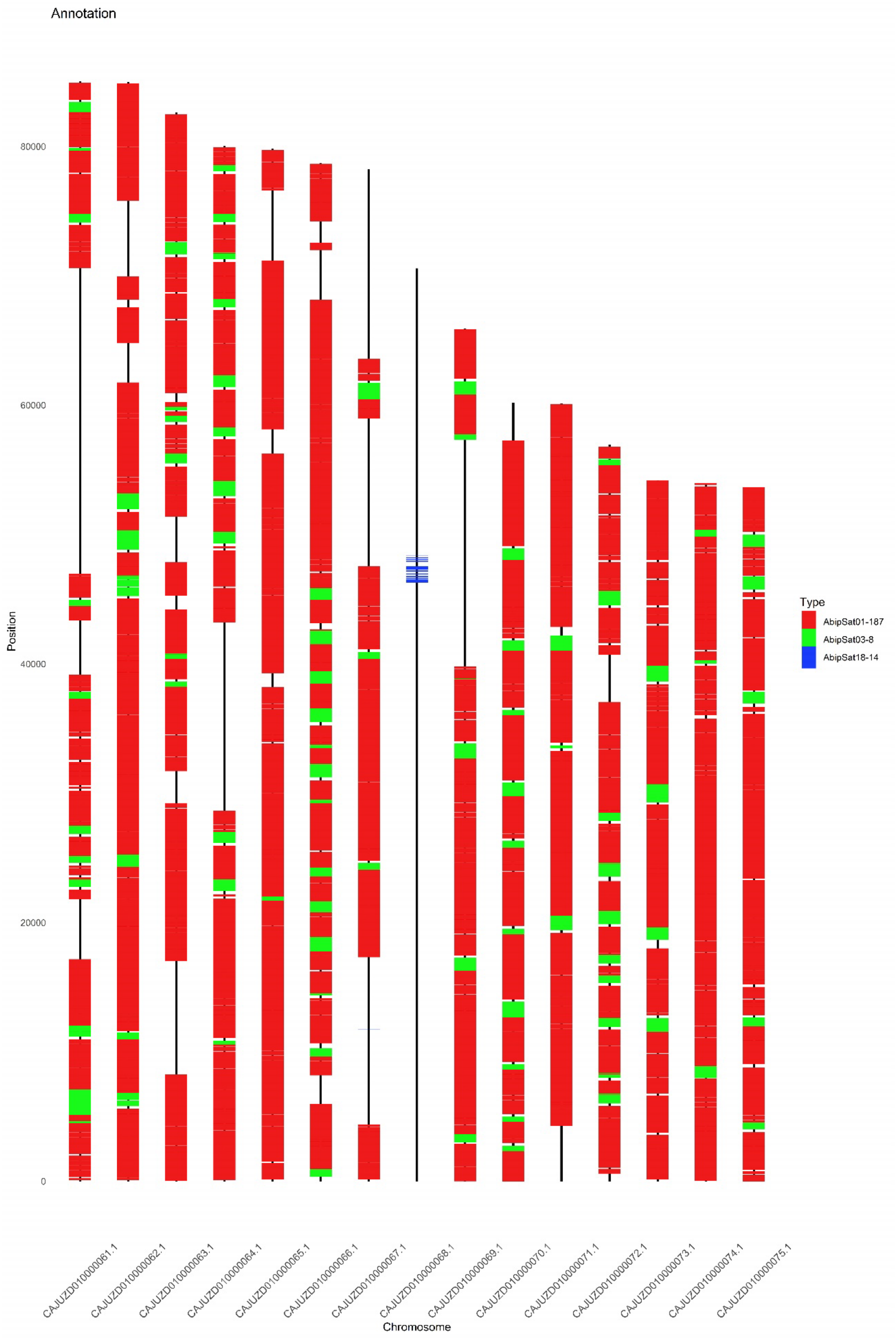

Annotation
Position
80000
60000
40000
20000
0
CAJUZD010000061.1
CAJUZD010000062.1
CAJUZD010000063.1
CAJUZD010000064.1
CAJUZD010000065.1
CAJUZD010000066.1
CAJUZD010000067.1
CAJUZD010000068.1
CAJUZD010000069.1
CAJUZD010000070.1
CAJUZD010000071.1
CAJUZD010000072.1
CAJUZD010000073.1
CAJUZD010000074.1
CAJUZD010000075.1
Chromosome
Type
AbipSat01-187
AbipSat03-8
AbipSat18-14

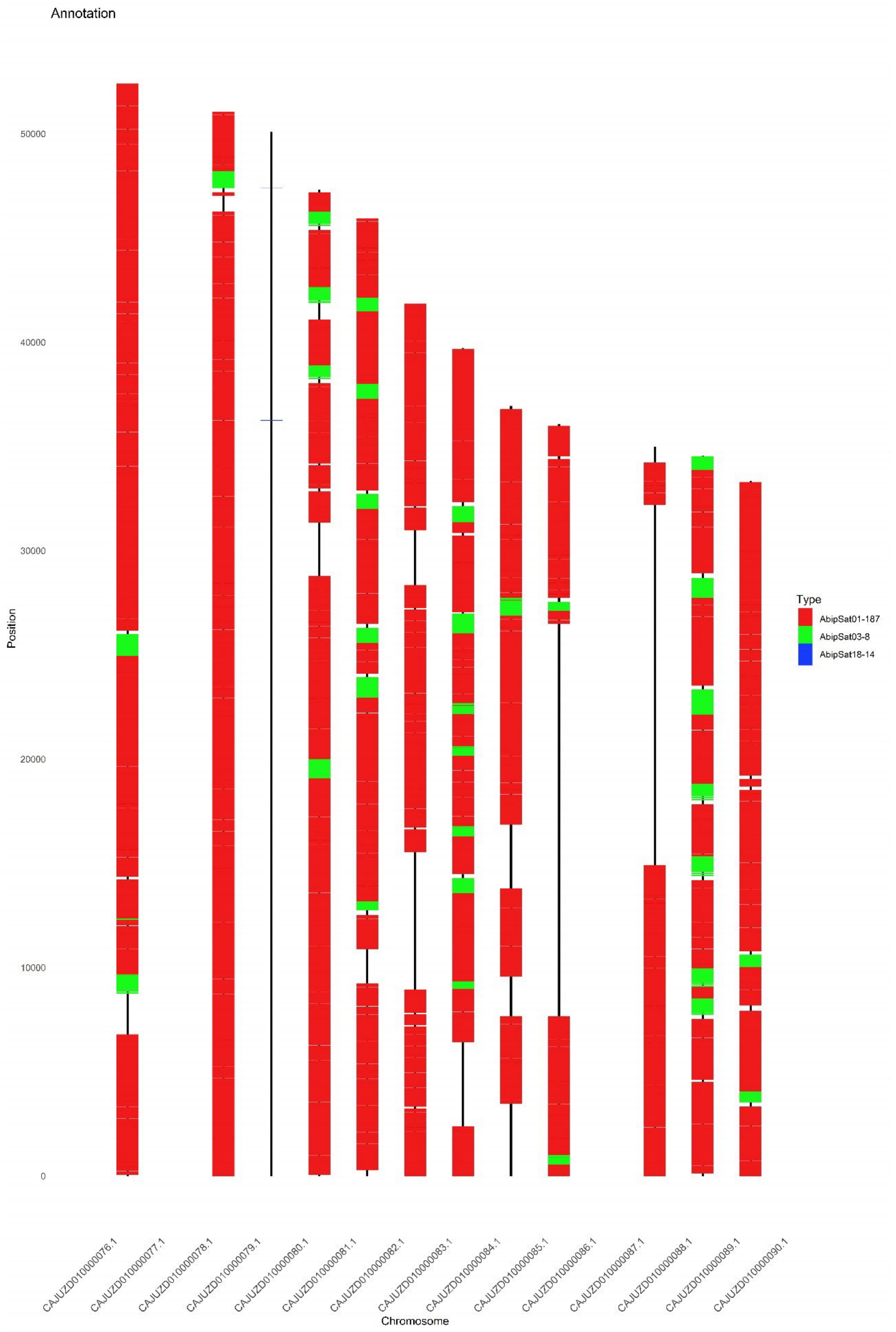
Annotation
Position
50000
40000
30000
20000
10000
0
Chromosome
CAJUZD010000076.1
CAJUZD010000077.1
CAJUZD010000078.1
CAJUZD010000079.1
CAJUZD010000080.1
CAJUZD010000081.1
CAJUZD010000082.1
CAJUZD010000083.1
CAJUZD010000084.1
CAJUZD010000085.1
CAJUZD010000086.1
CAJUZD010000087.1
CAJUZD010000088.1
CAJUZD010000089.1
CAJUZD010000090.1
Type
AbipSat01-187
AbipSat03-8
AbipSat18-14

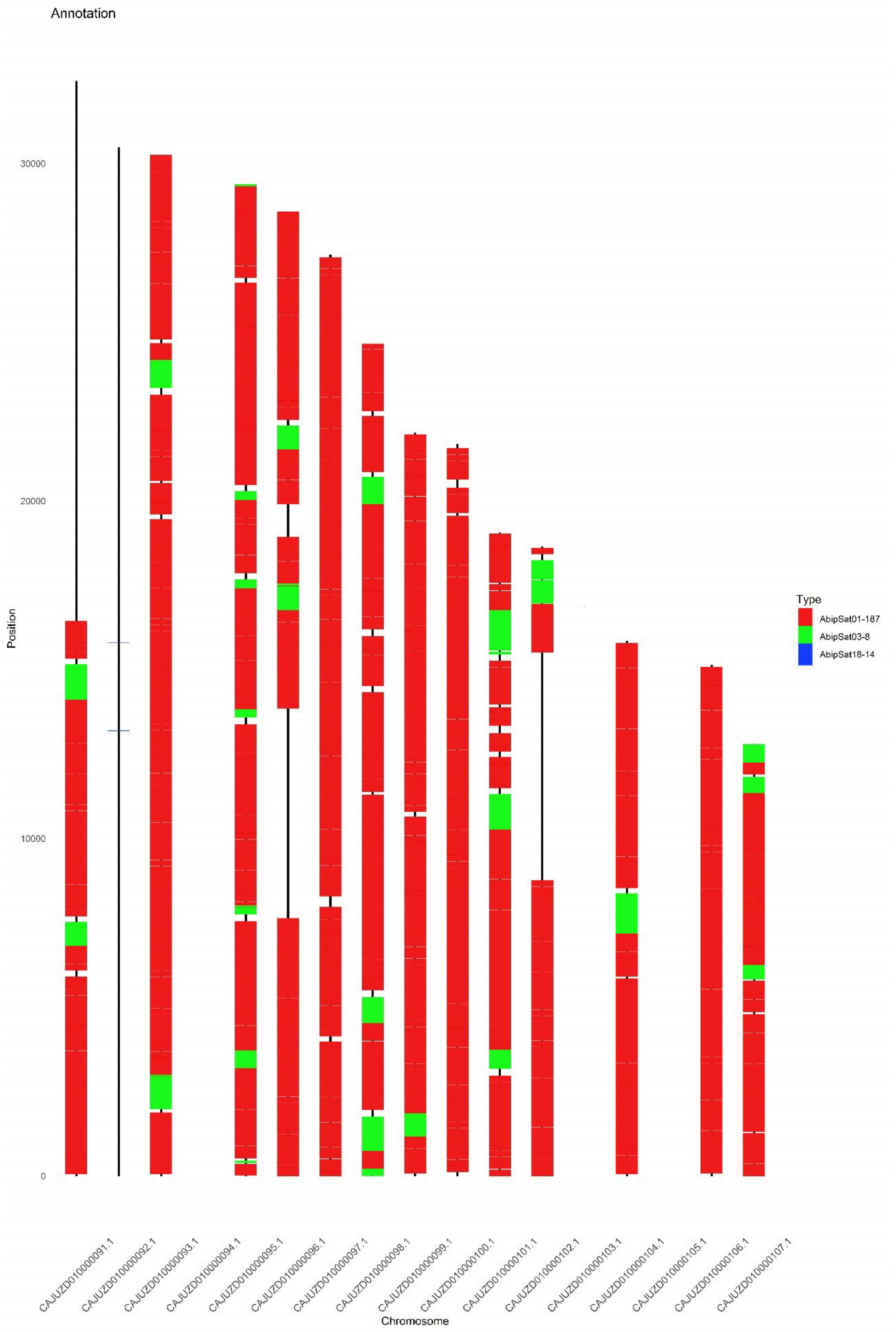

Annotation
Position
30000
20000
10000
0
Chromosome
CAJUZD010000091.1
CAJUZD010000092.1
CAJUZD010000093.1
CAJUZD010000094.1
CAJUZD010000095.1
CAJUZD010000096.1
CAJUZD010000097.1
CAJUZD010000098.1
CAJUZD010000099.1
CAJUZD010000100.1
CAJUZD010000101.1
CAJUZD010000102.1
CAJUZD010000103.1
CAJUZD010000104.1
CAJUZD010000105.1
CAJUZD010000106.1
CAJUZD010000107.1
Type
AbipSat01-187
AbipSat03-8
AbipSat18-14
