# Supplementary material for: Satellitome Analysis of Adalia bipunctata (Coleoptera): Revealing Centromeric Turnover and Potential Chromosome Rearrangements in a Comparative Interspecific Study
Source: Int J Mol Sci. 2024 Aug 25;25(17):9214. doi: 10.3390/ijms25179214 (PMC11394905; doi:10.3390/ijms25179214)
Supplement: Supplementary file 1 [file ijms-25-09214-s001.zip › Supplementary Table S1.pdf]

Table S1: Abundance of the satDNAs of *Adalia bipunctata* satellitome in the *Adalia bipunctata* and *Adalia decempunctata* genomes. ND = non-detected.

	Adalia bipunctata (reads)	*Adalia decempunctata* (reads)
AbipSat01-187	9.9808	ND
AbipSat02-497	1.6789	0.8261
AbipSat03-8	0.3864	< 0.0001
AbipSat04-193	0.2678	0.0263
AbipSat05-258	0.2383	0.0152
AbipSat06-176	0.2328	0.0025
AbipSat07-579	0.1054	0.0838
AbipSat08-316	0.1017	0.2652
AbipSat09-473	0.0916	0.1946
AbipSat10-1902	0.0874	0.6683
AbipSat11-151	0.0816	0.0608
AbipSat12-233	0.0810	ND
AbipSat13-1767	0.0799	0.0548
AbipSat14-53	0.0751	0.1259
AbipSat15-179	0.0712	0.0187
AbipSat16-443	0.0660	0.0980
AbipSat17-86	0.0642	0.0564
AbipSat18-14	0.0636	0.0028
AbipSat19-1237	0.0618	0.0406
AbipSat20-176	0.0550	0.0342
AbipSat21-1899	0.0537	0.0387
AbipSat22-148	0.0527	0.0615
AbipSat23-376	0.0474	0.0857
AbipSat24-5-TEL	0.0434	0.0252
AbipSat25-176	0.0416	0.0130
AbipSat26-2235	0.0398	0.0036
AbipSat27-84	0.0352	ND
AbipSat28-165	0.0330	0.0237
AbipSat29-174	0.0282	0.0140
AbipSat30-212	0.0278	ND
AbipSat31-145	0.0241	0.0378
AbipSat32-163	0.0237	0.1306
AbipSat33-284	0.0228	0.0356
AbipSat34-137	0.0223	0.0342
AbipSat35-141	0.0218	0.0252
AbipSat36-157	0.0194	0.0064
AbipSat37-297	0.0153	0.0062
AbipSat38-2435	0.0141	0.0189
AbipSat39-159	0.0132	0.0100
AbipSat40-152	0.0131	0.0055
AbipSat41-69	0.0117	0.0004
AbipSat42-21	0.0116	0.0307
AbipSat43-282	0.0111	0.0251

AbipSat44-148	0.0110	0.0402
AbipSat45-307	0.0109	0.0094
AbipSat46-126	0.0089	0.0043
AbipSat47-399	0.0080	0.0064
AbipSat48-18	0.0076	ND
AbipSat49-148	0.0075	0.0103
AbipSat50-199	0.0072	0.0161
AbipSat51-128	0.0071	0.0022
AbipSat52-177	0.0064	0.0040
AbipSat53-22	0.0059	0.0035
AbipSat54-271	0.0058	0.0008
AbipSat55-145	0.0054	0.0058
AbipSat56-30	0.0051	ND
AbipSat57-144	0.0049	0.0004
AbipSat58-518	0.0047	0.0067
AbipSat59-105	0.0039	0.0037
AbipSat60-160	0.0038	0.0019
AbipSat61-175	0.0038	0.0019
AbipSat62-144	0.0037	0.0017
AbipSat63-186	0.0031	0.0015
AbipSat64-201	0.0031	ND
AbipSat65-286	0.0029	< 0.0001
AbipSat66-169	0.0028	ND
AbipSat67-304	0.0027	0.0012
AbipSat68-237	0.0025	0.0008
AbipSat69-438	0.0025	0.0016
AbipSat70-168	0.0024	ND
AbipSat71-438	0.0023	0.0012
AbipSat72-426	0.0022	0.0022
AbipSat73-396	0.0020	0.0004
AbipSat74-344	0.0019	ND
AbipSat75-471	0.0019	0.0008
AbipSat76-140	0.0018	ND
AbipSat77-155	0.0018	0.0016
AbipSat78-399	0.0017	0.0003
AbipSat79-309	0.0017	ND
AbipSat80-337	0.0016	ND
AbipSat81-258	0.0016	ND
AbipSat82-291	0.0014	0.0002
AbipSat83-191	0.0012	< 0.0001
AbipSat84-169	0.0011	ND
AbipSat85-284	0.0011	0.0002
AbipSat86-294	0.0009	ND
