# Supplementary figures and images for: Satellitome Analysis of Adalia bipunctata (Coleoptera): Revealing Centromeric Turnover and Potential Chromosome Rearrangements in a Comparative Interspecific Study
Source: Int J Mol Sci. 2024 Aug 25;25(17):9214. doi: 10.3390/ijms25179214 (PMC11394905; doi:10.3390/ijms25179214)

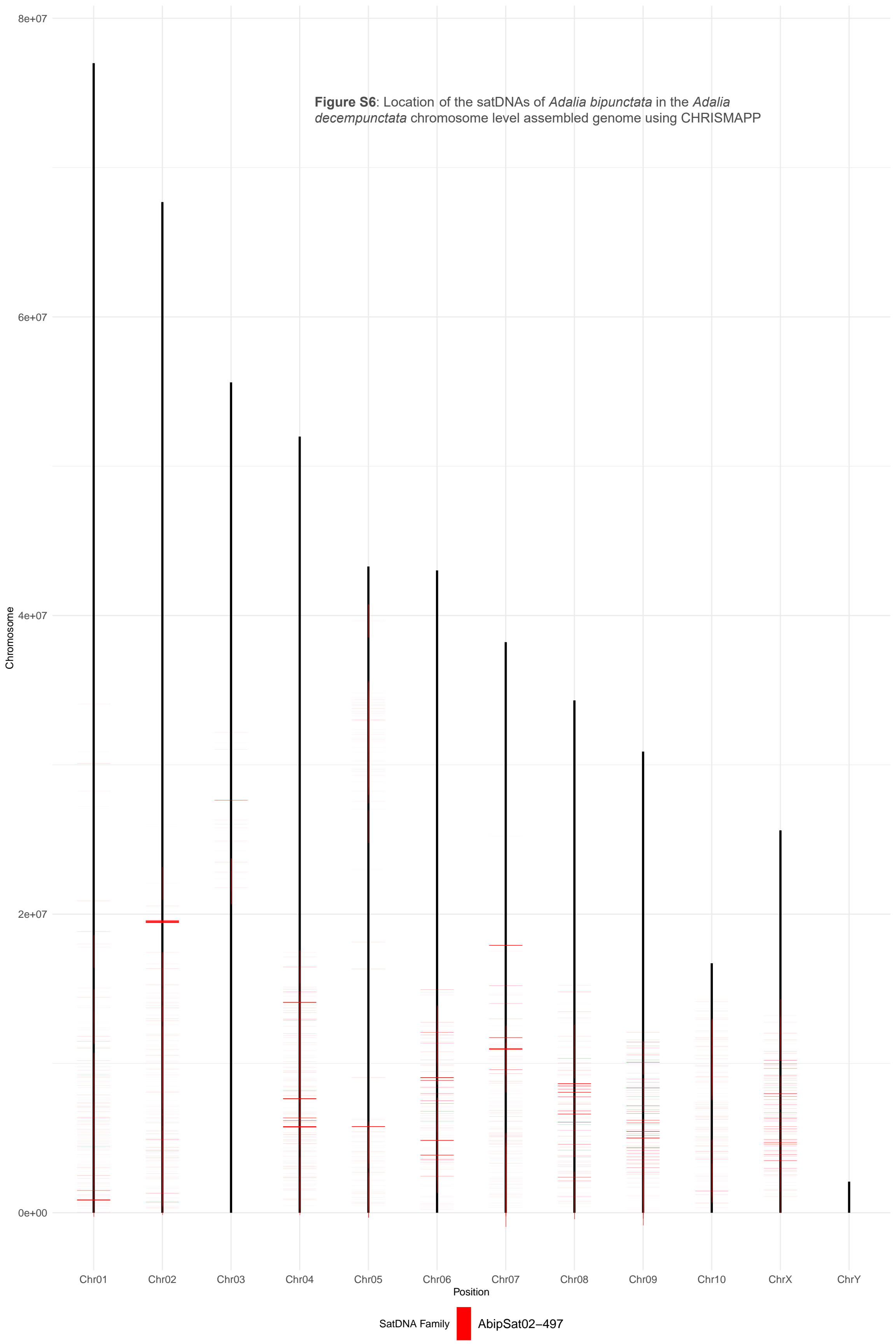

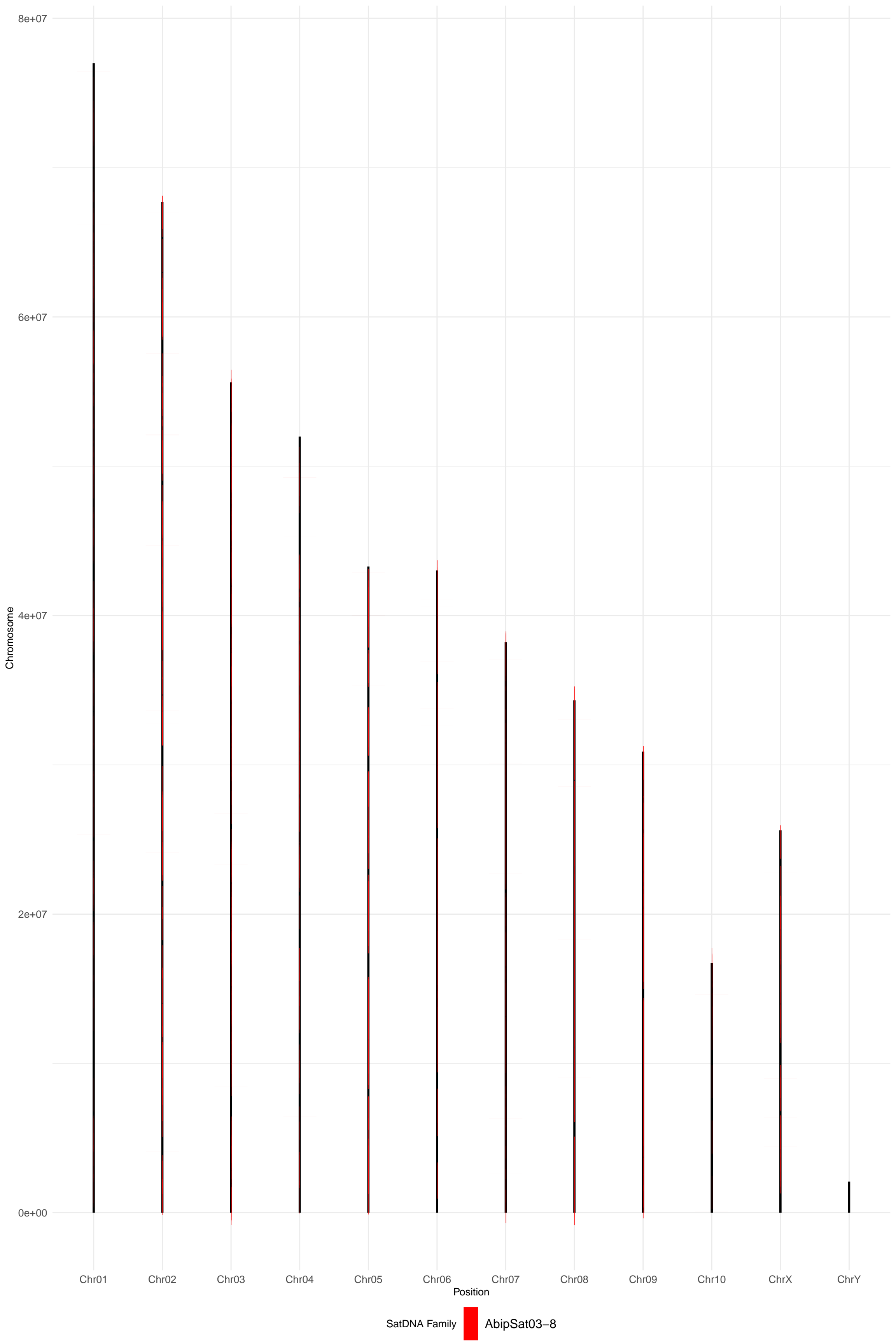

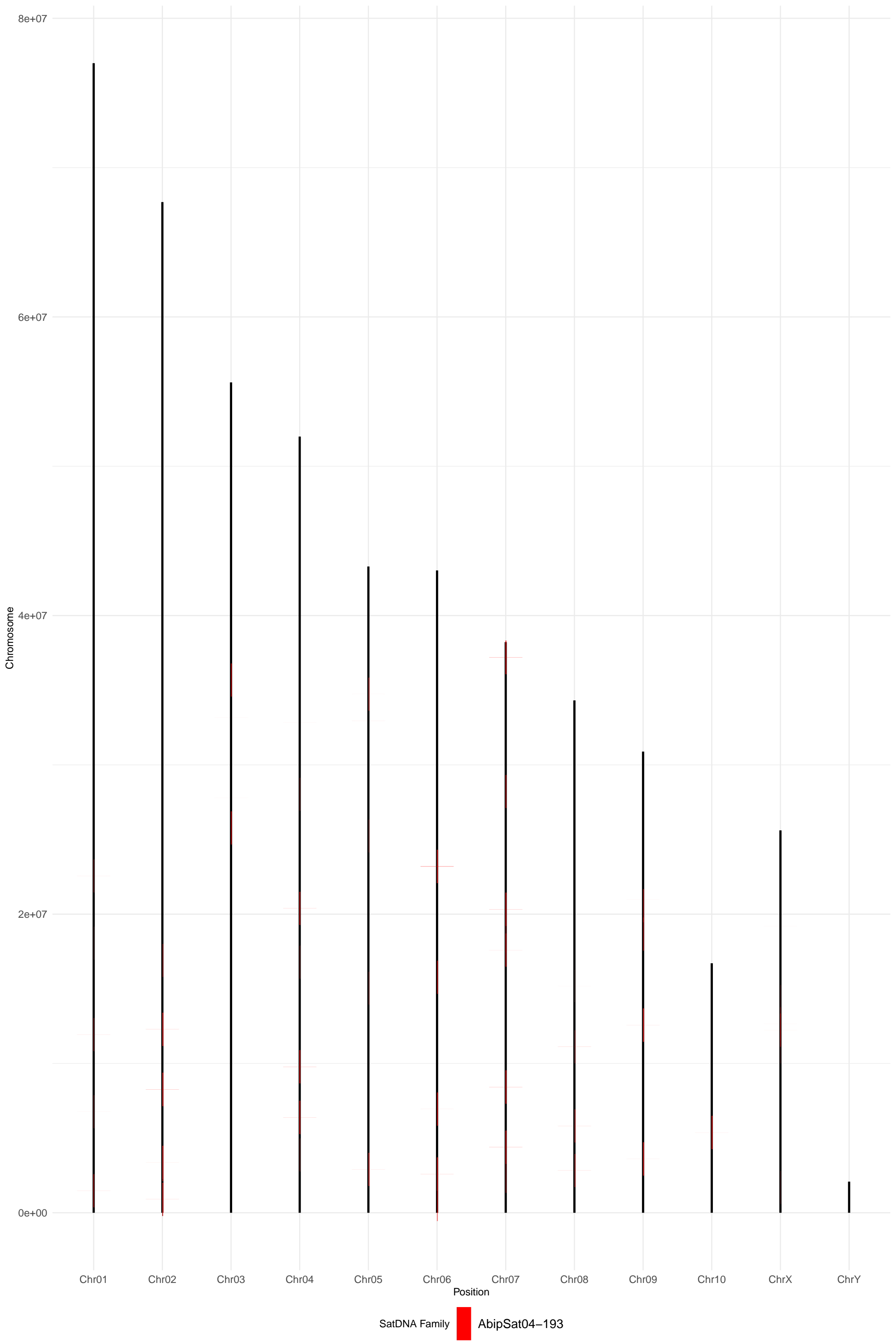

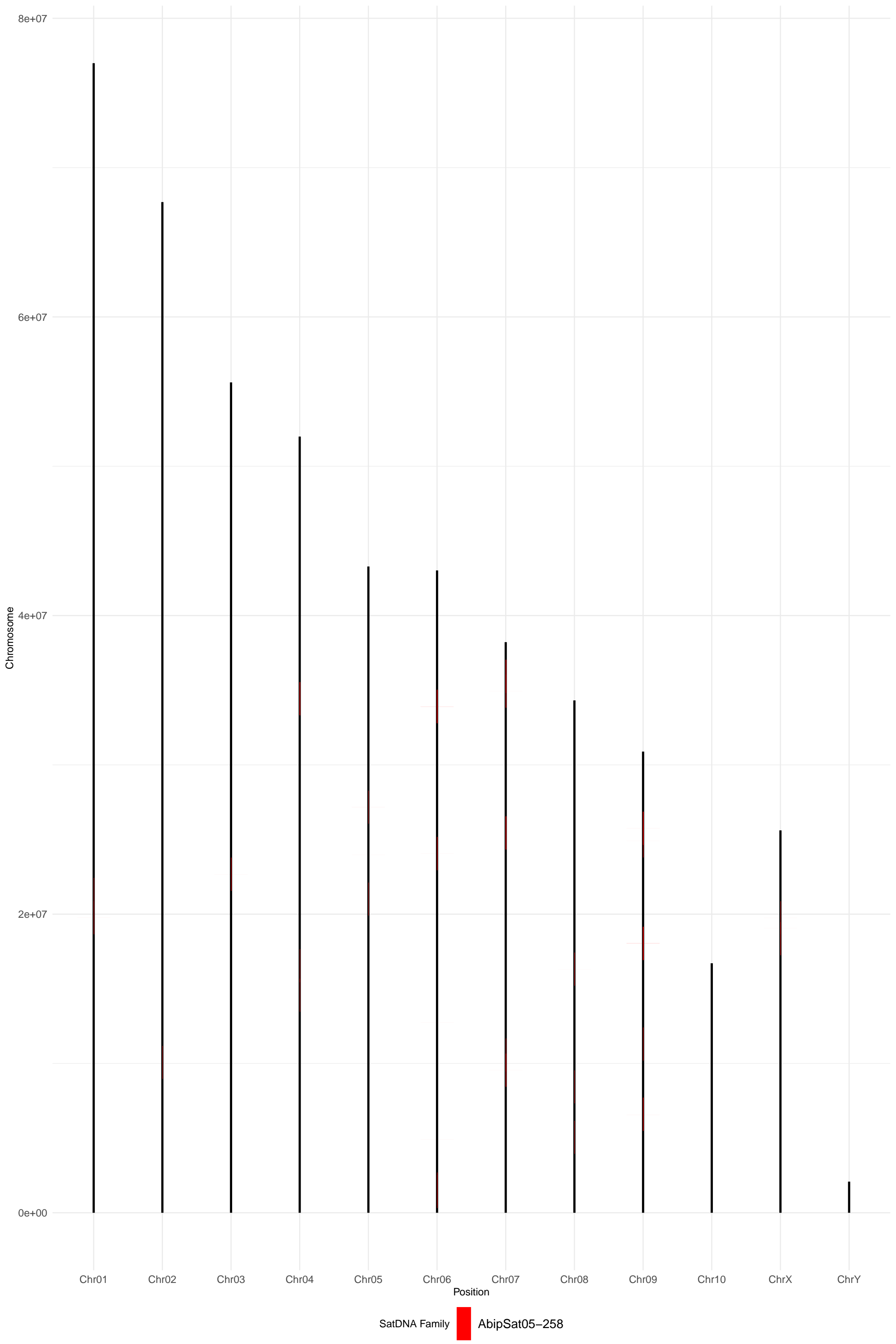

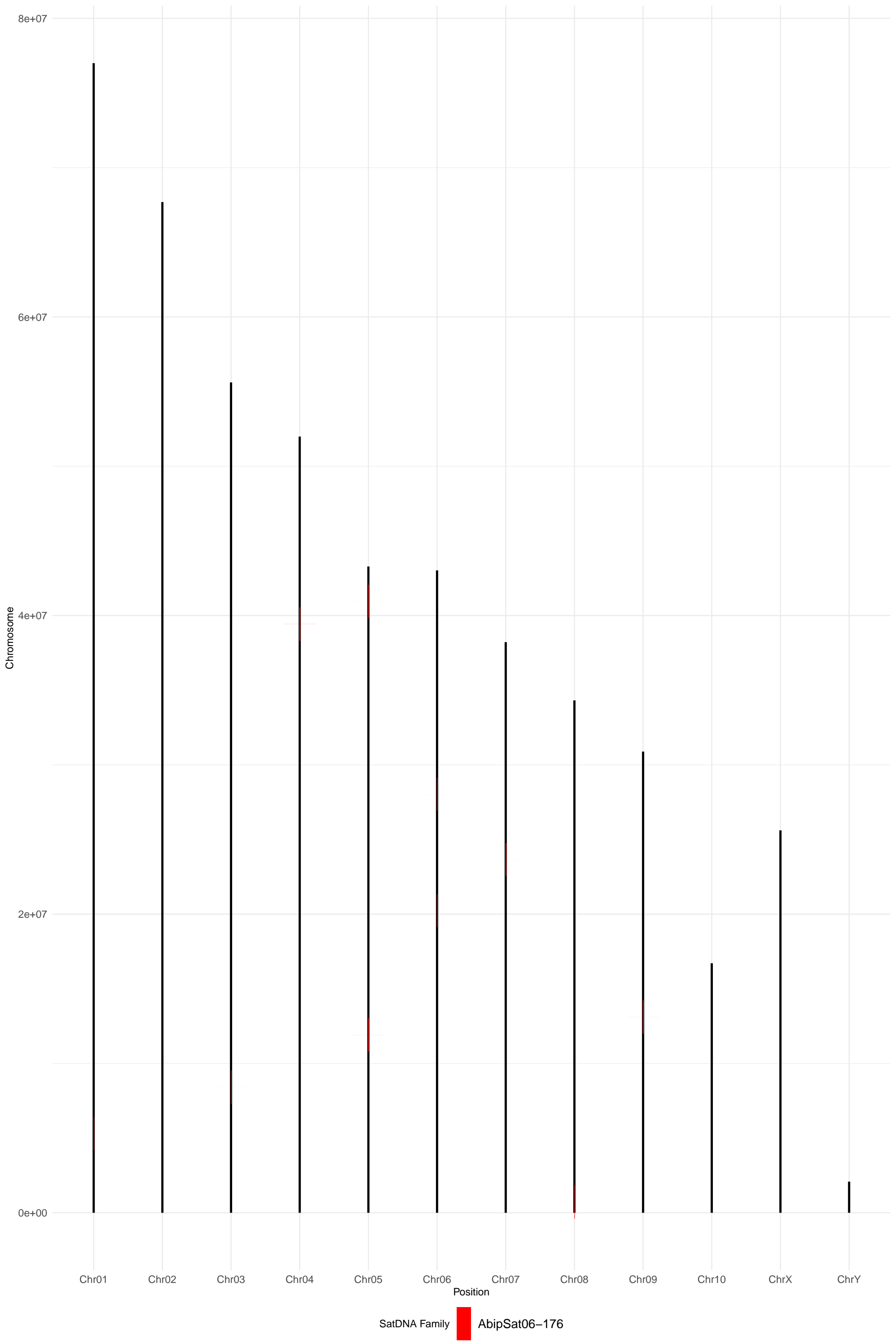

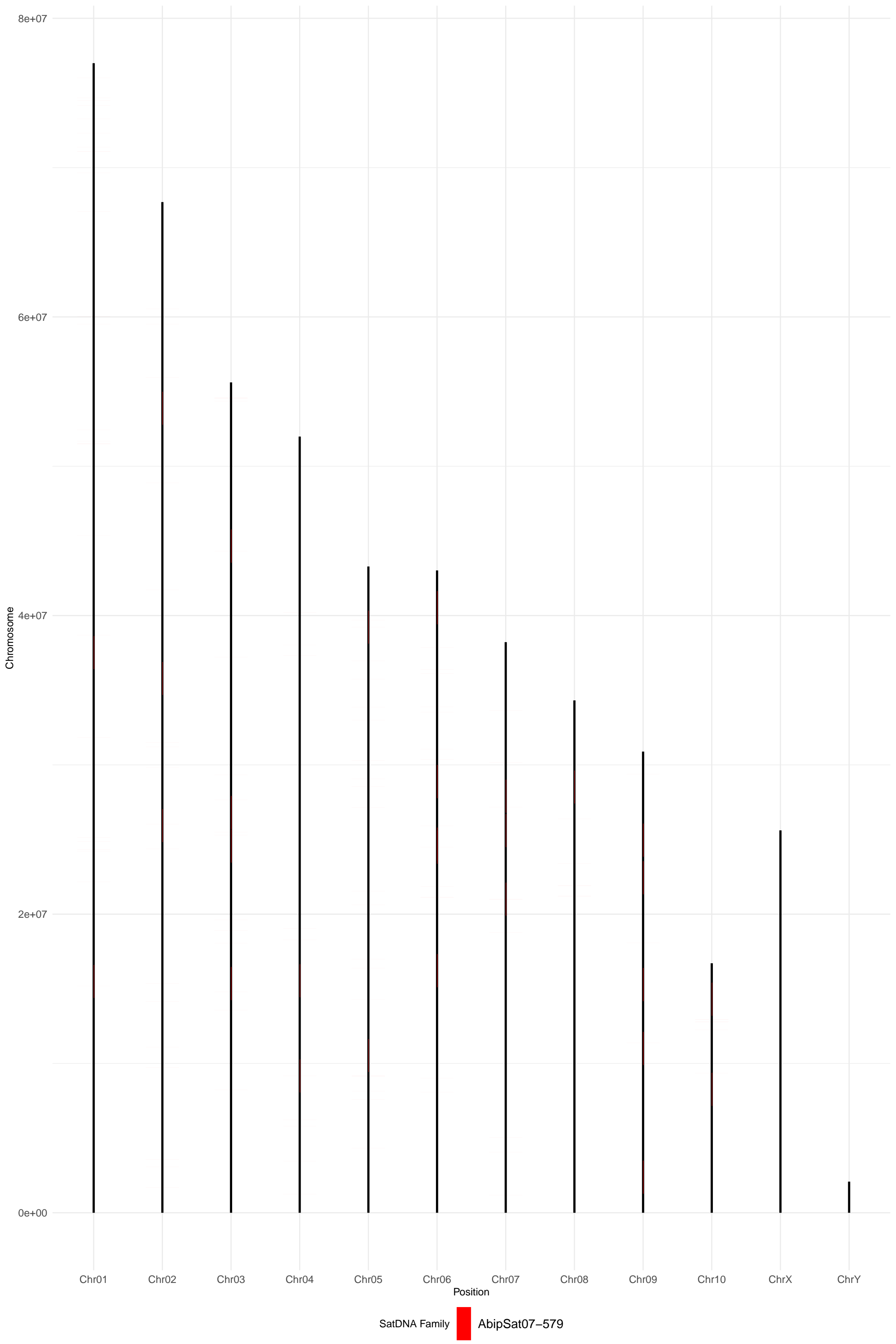

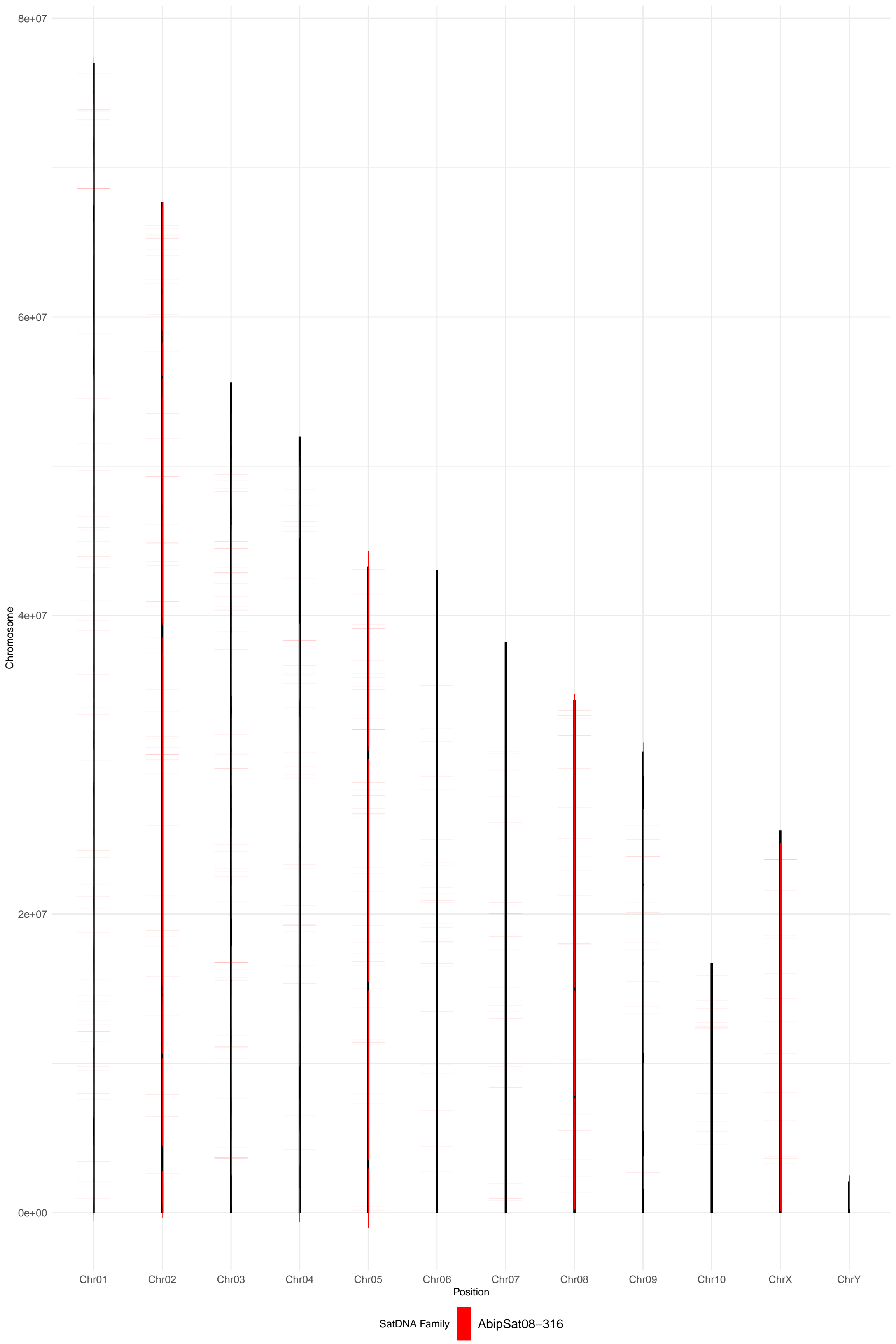

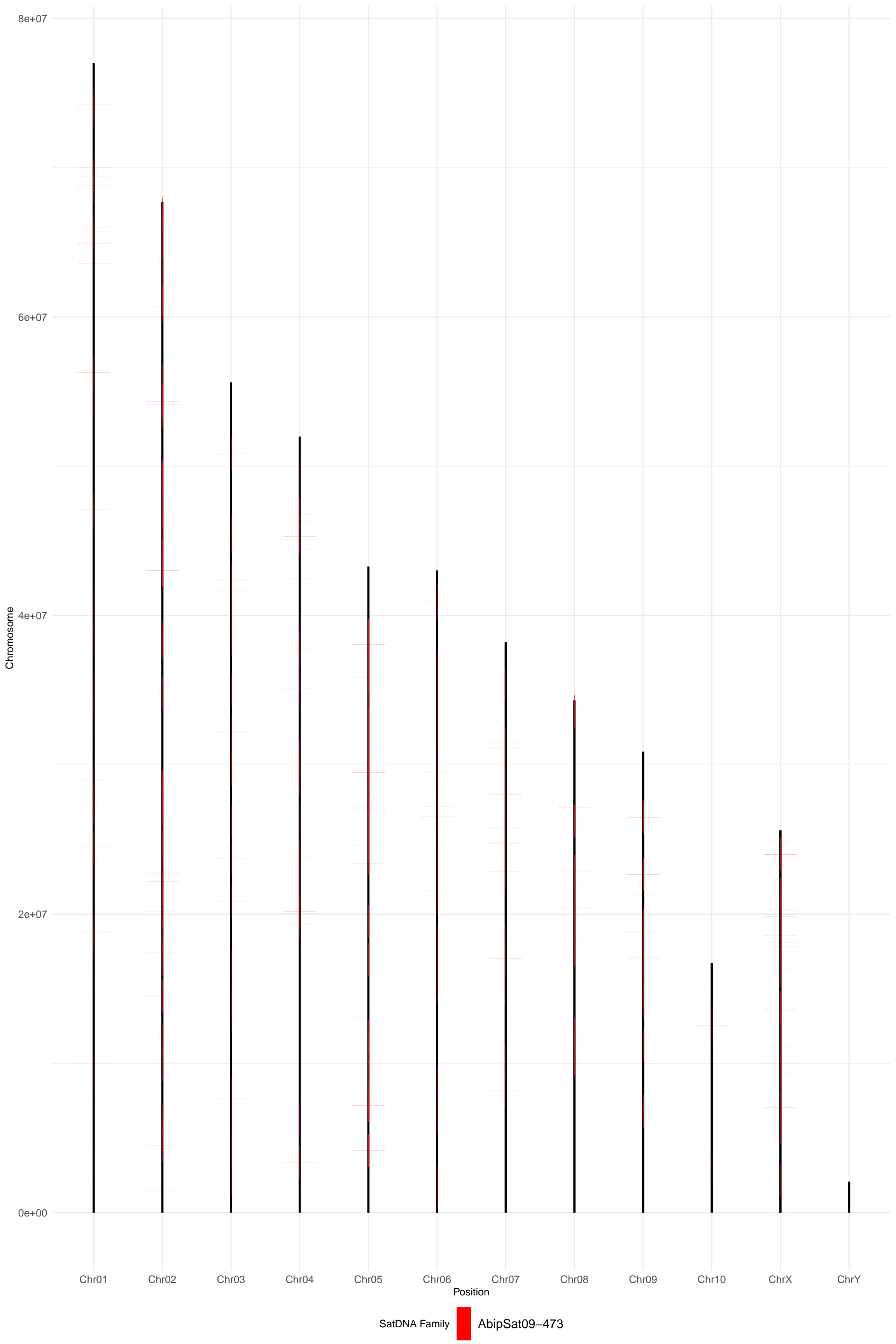

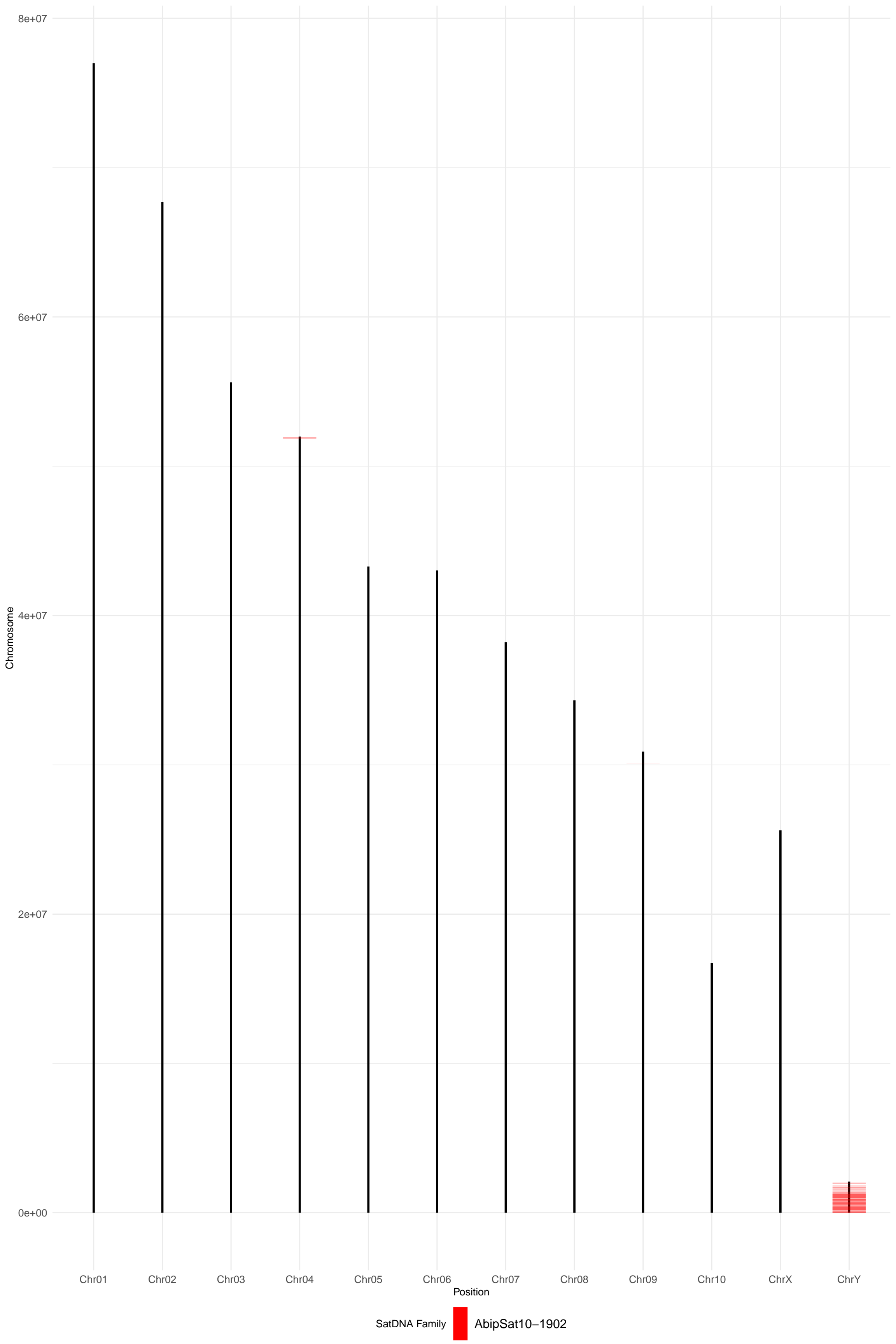

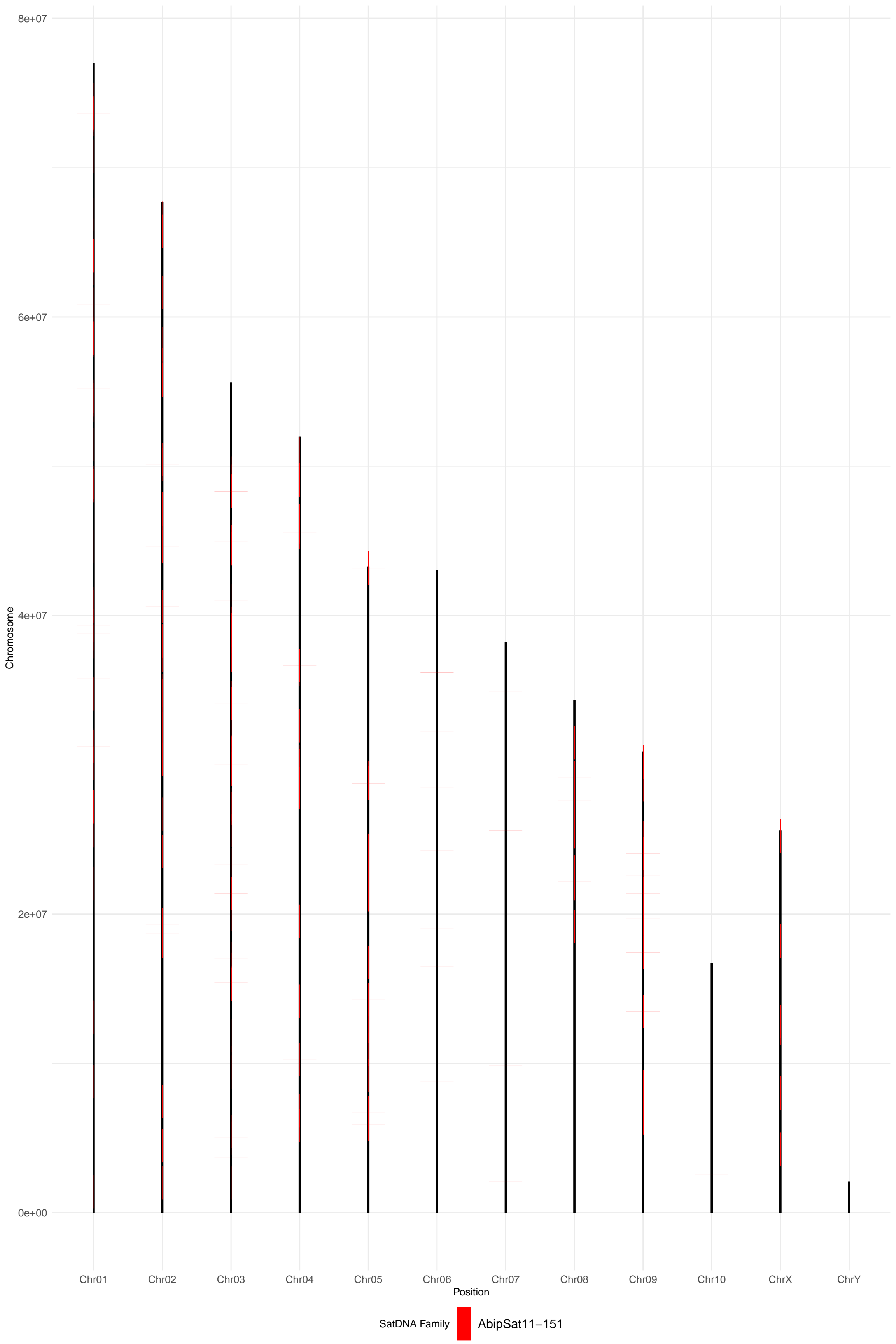

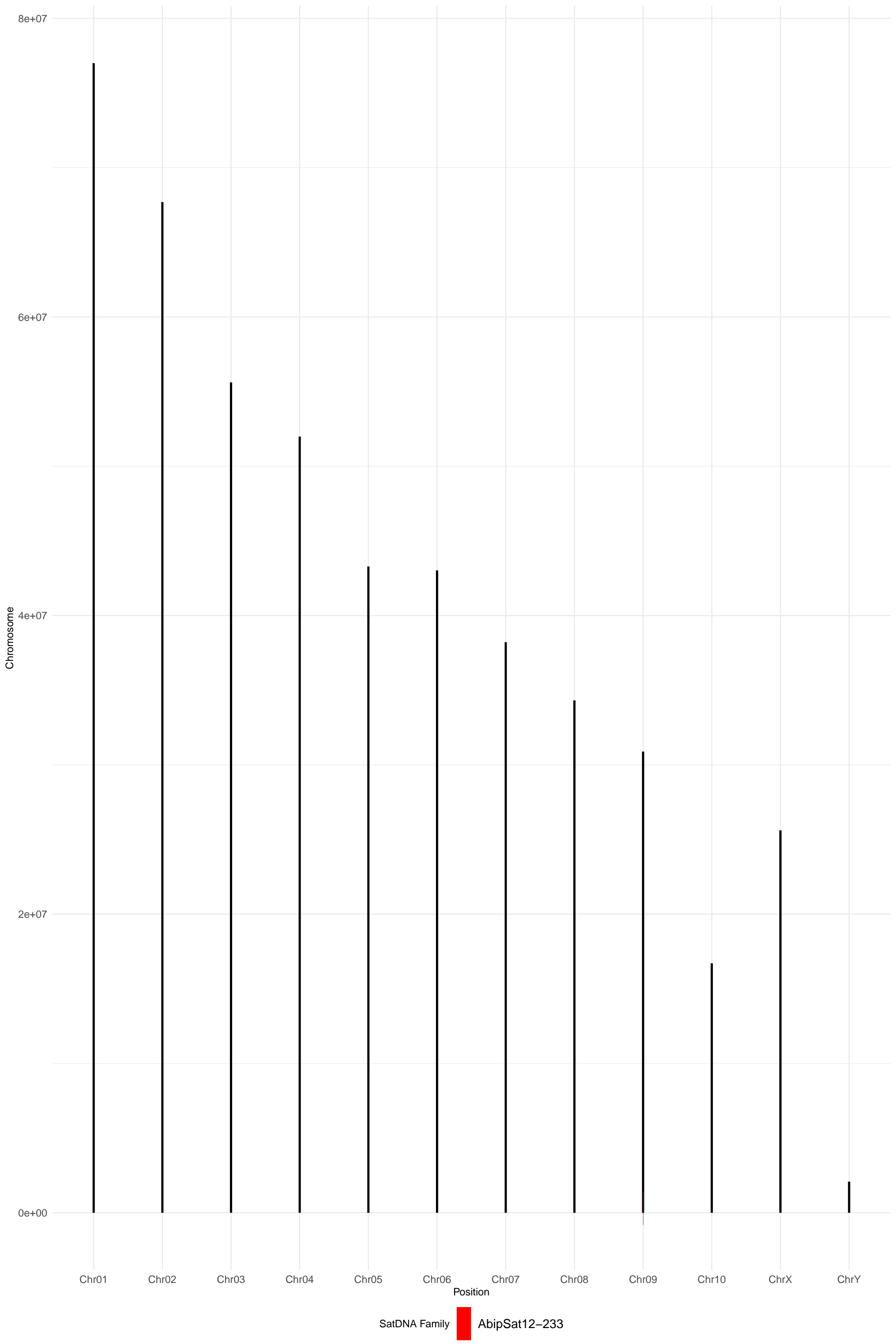

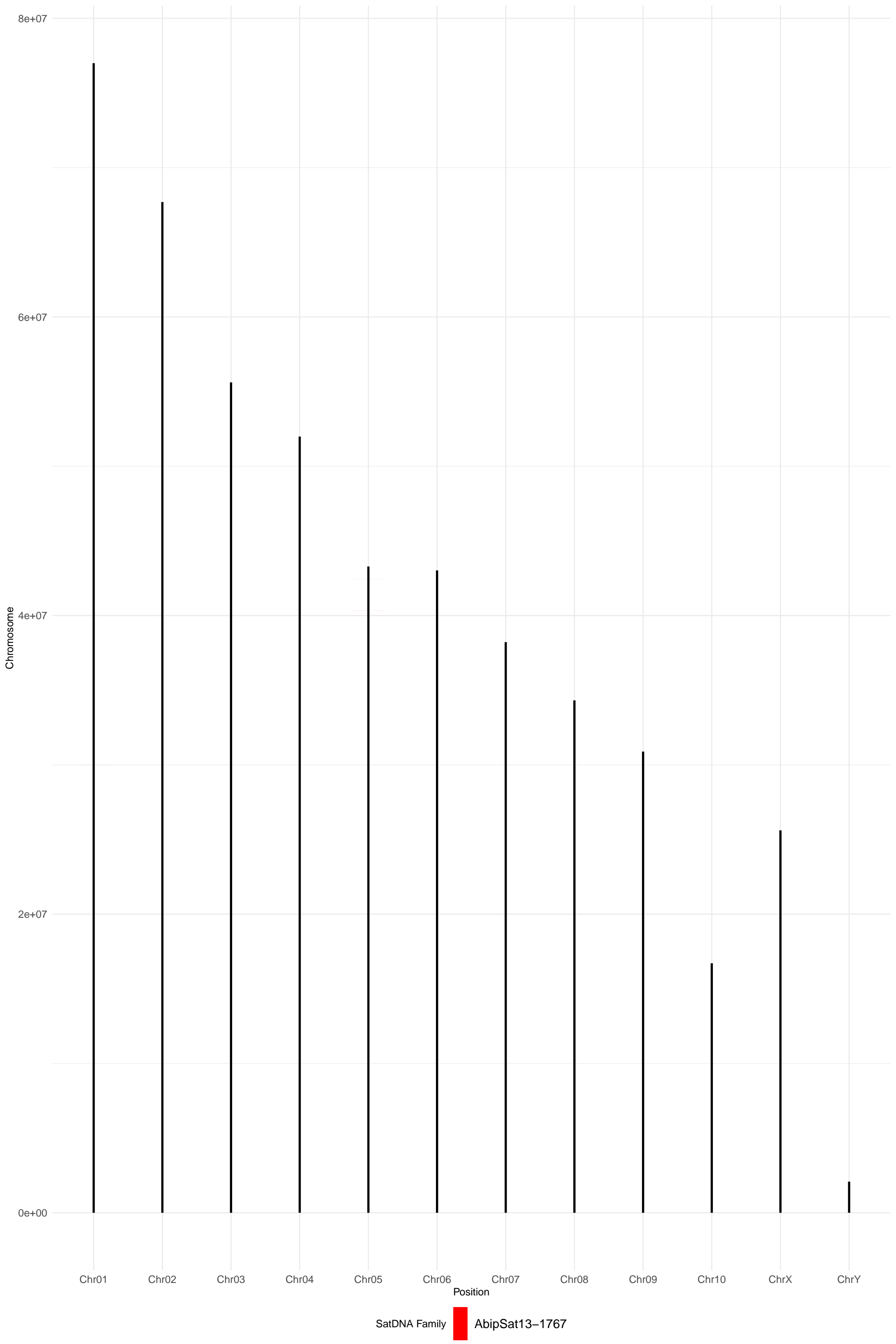

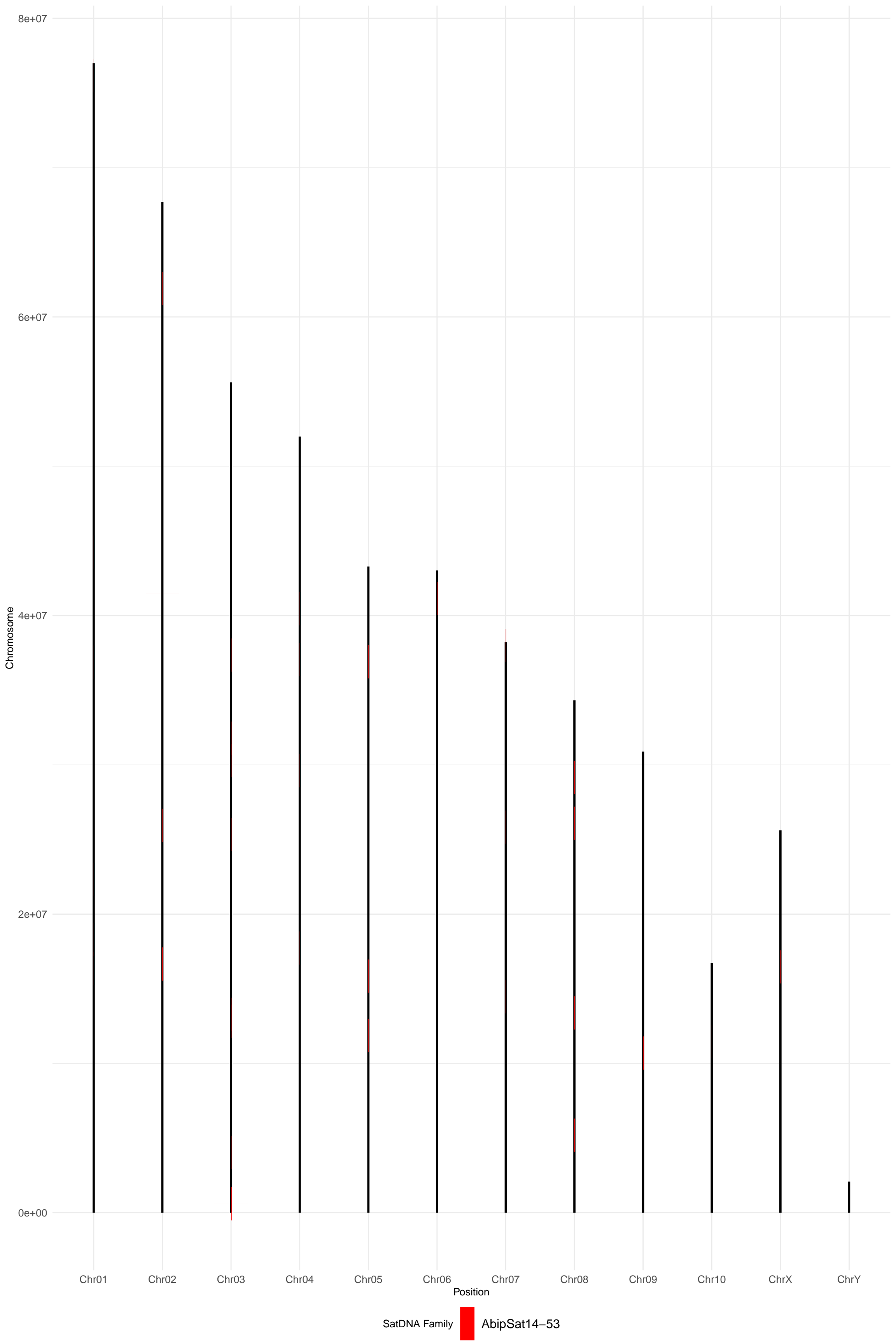

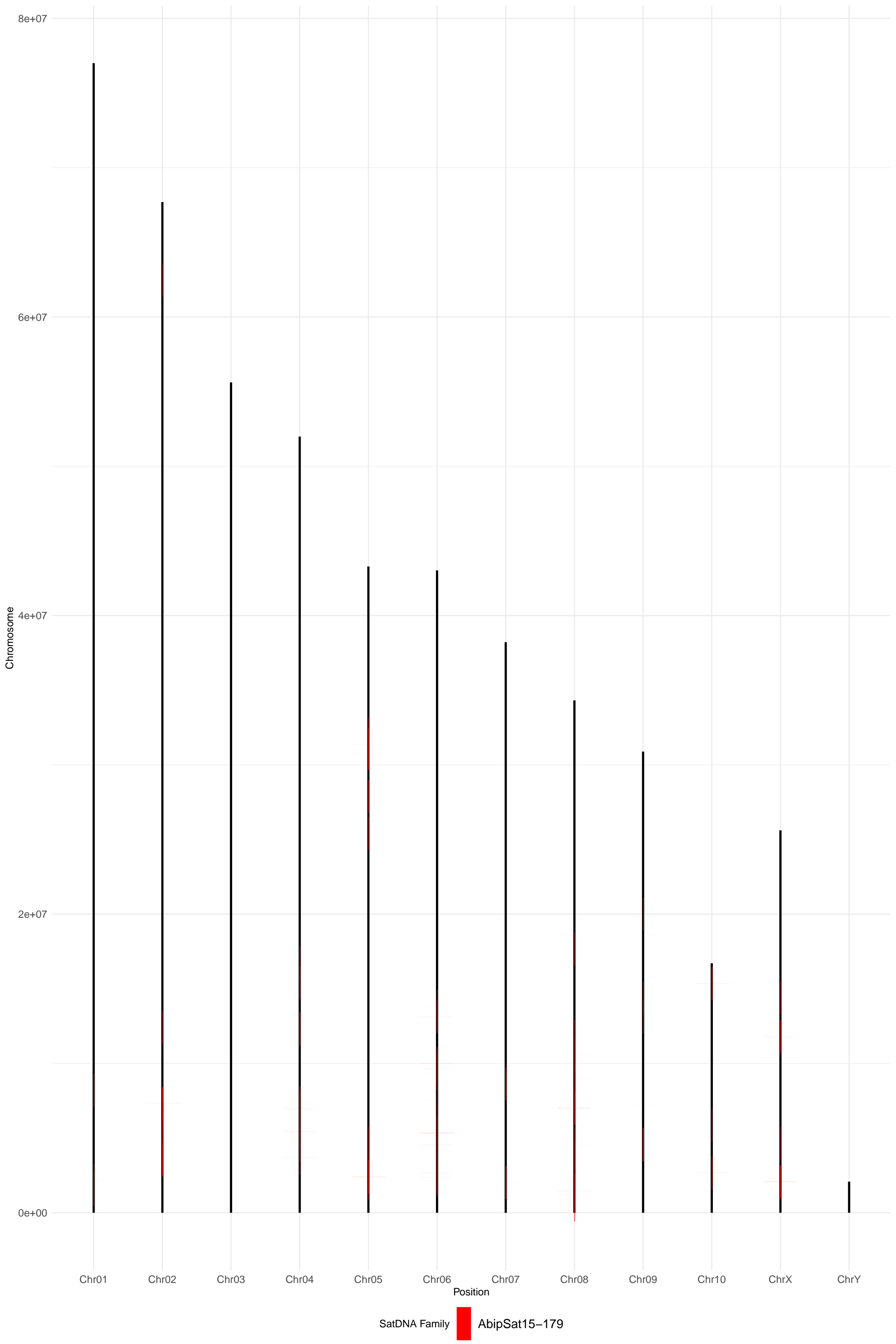

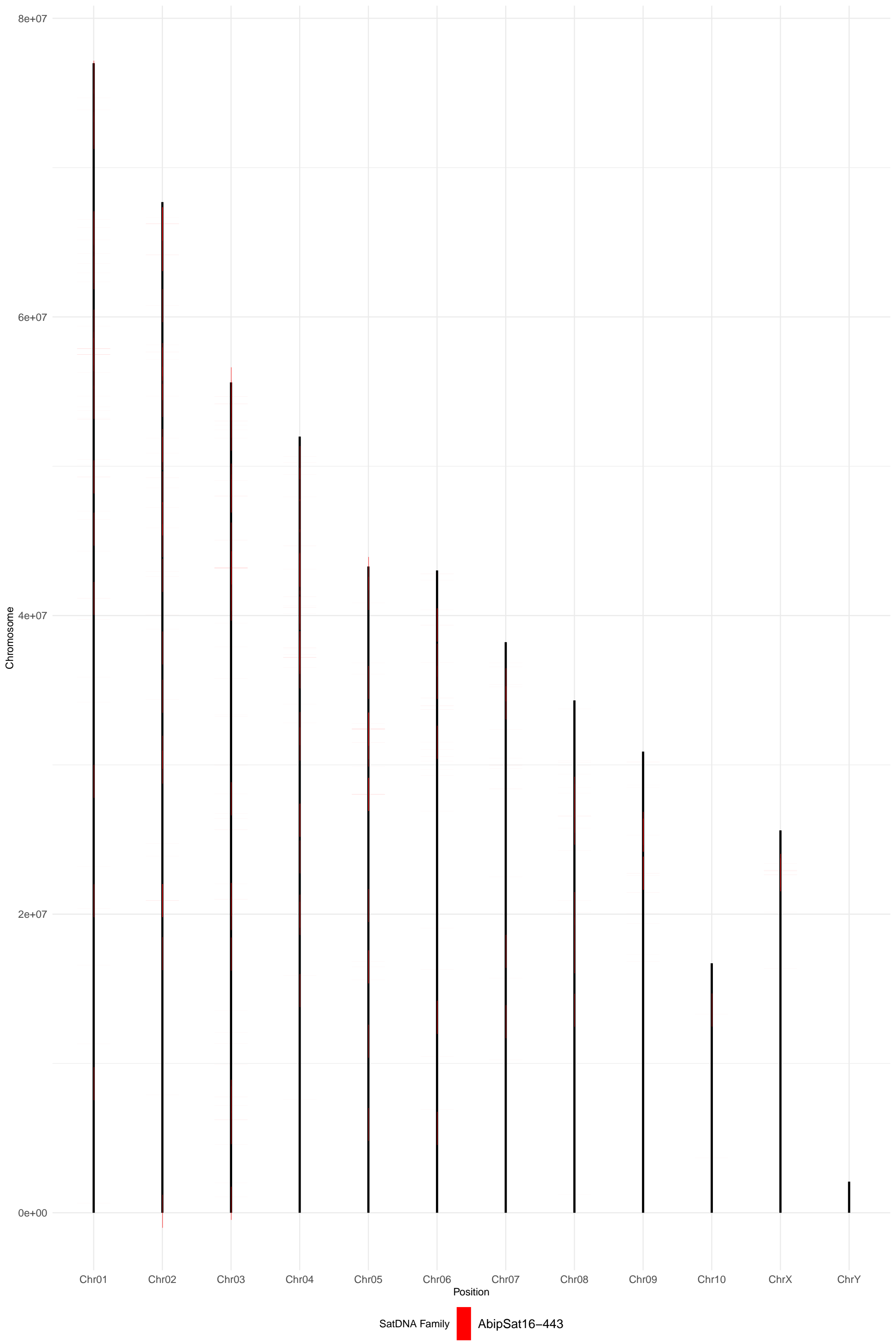

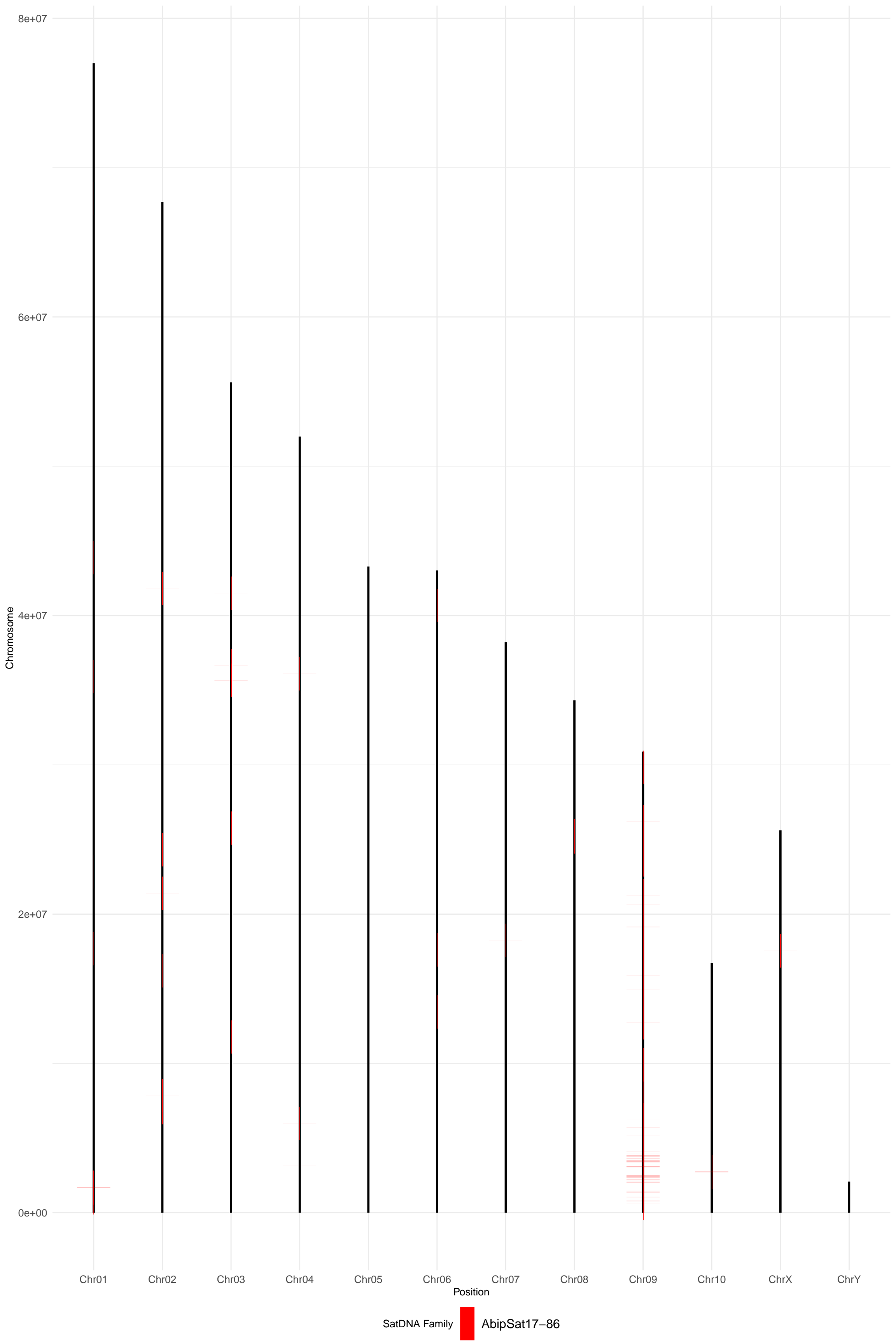

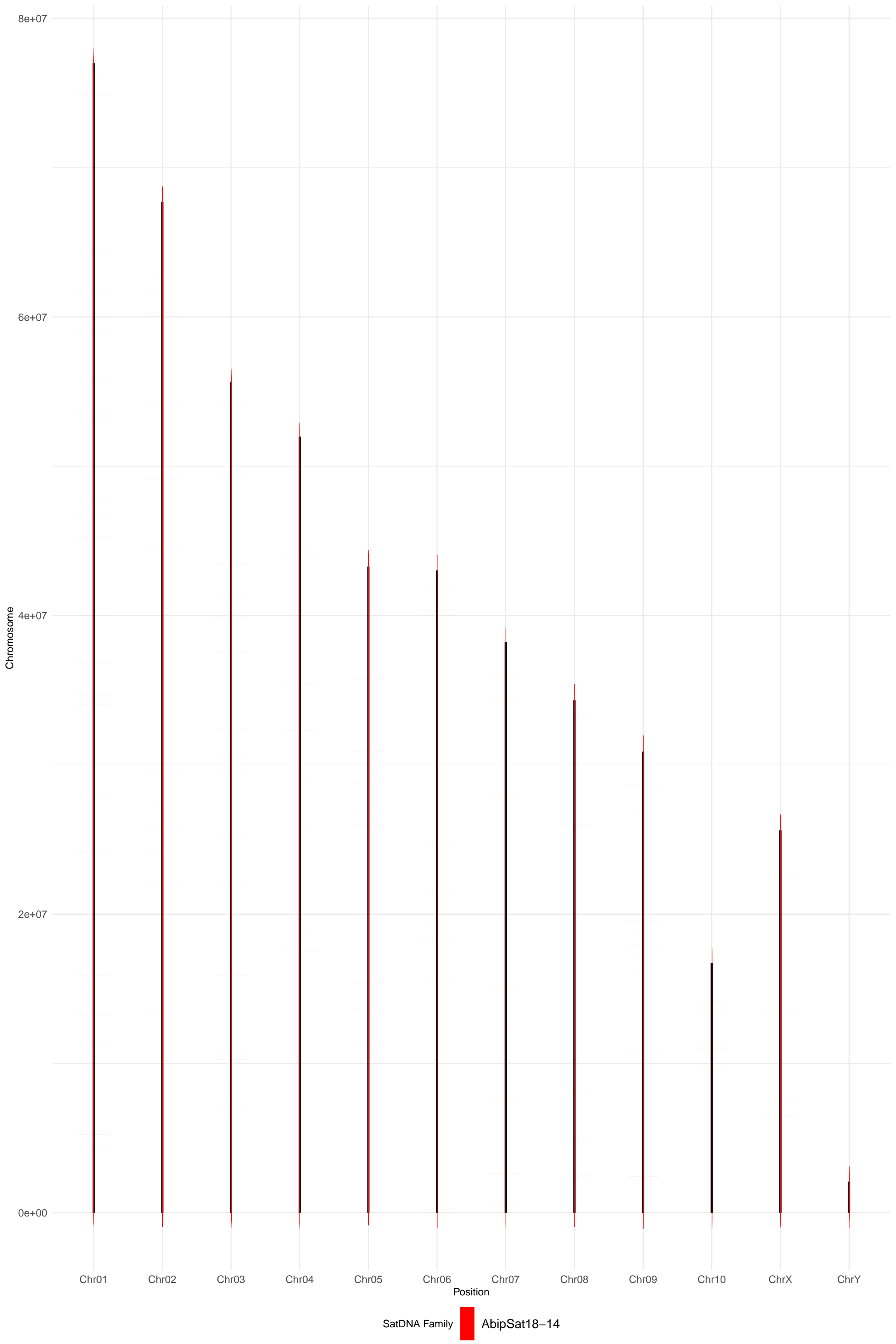

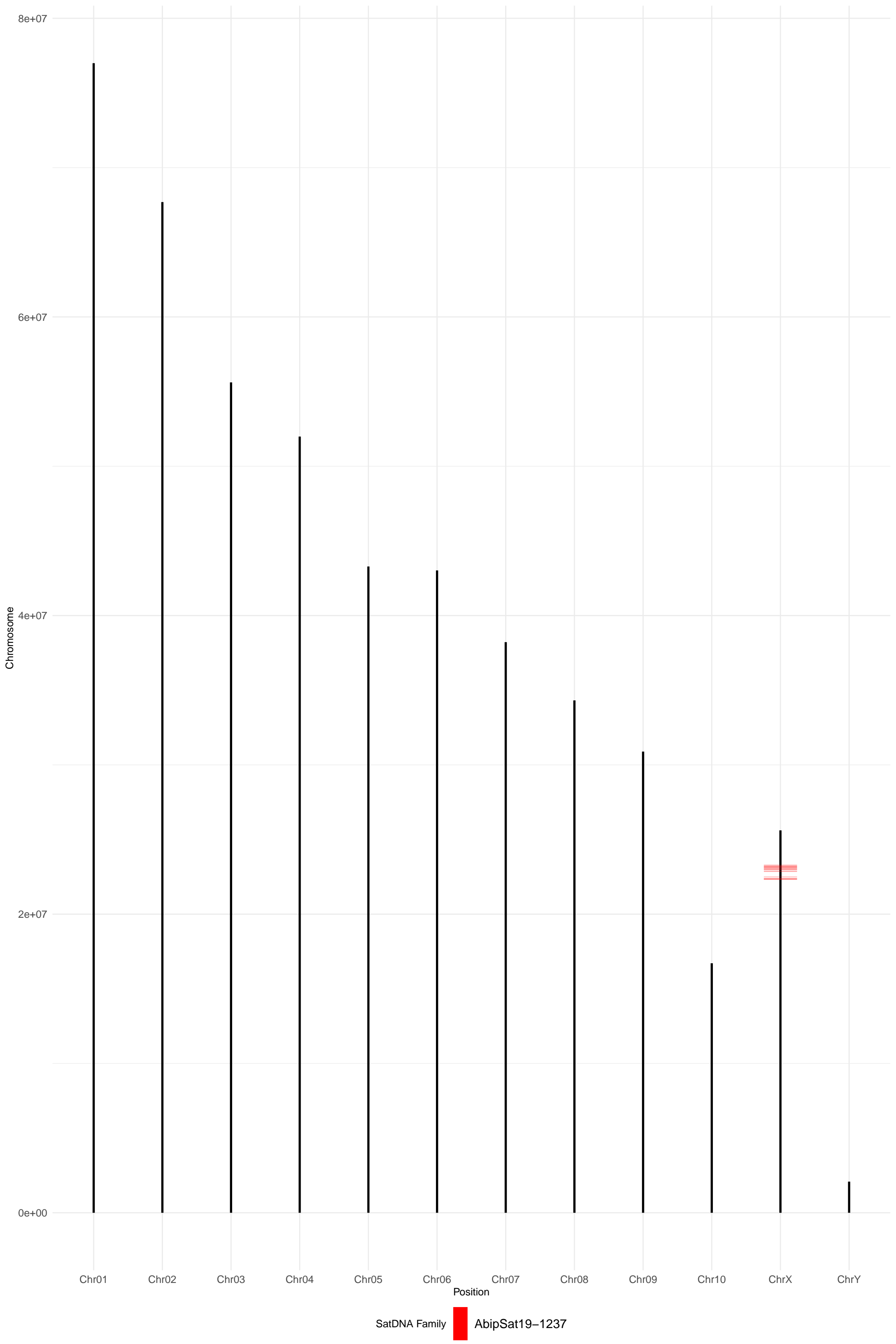

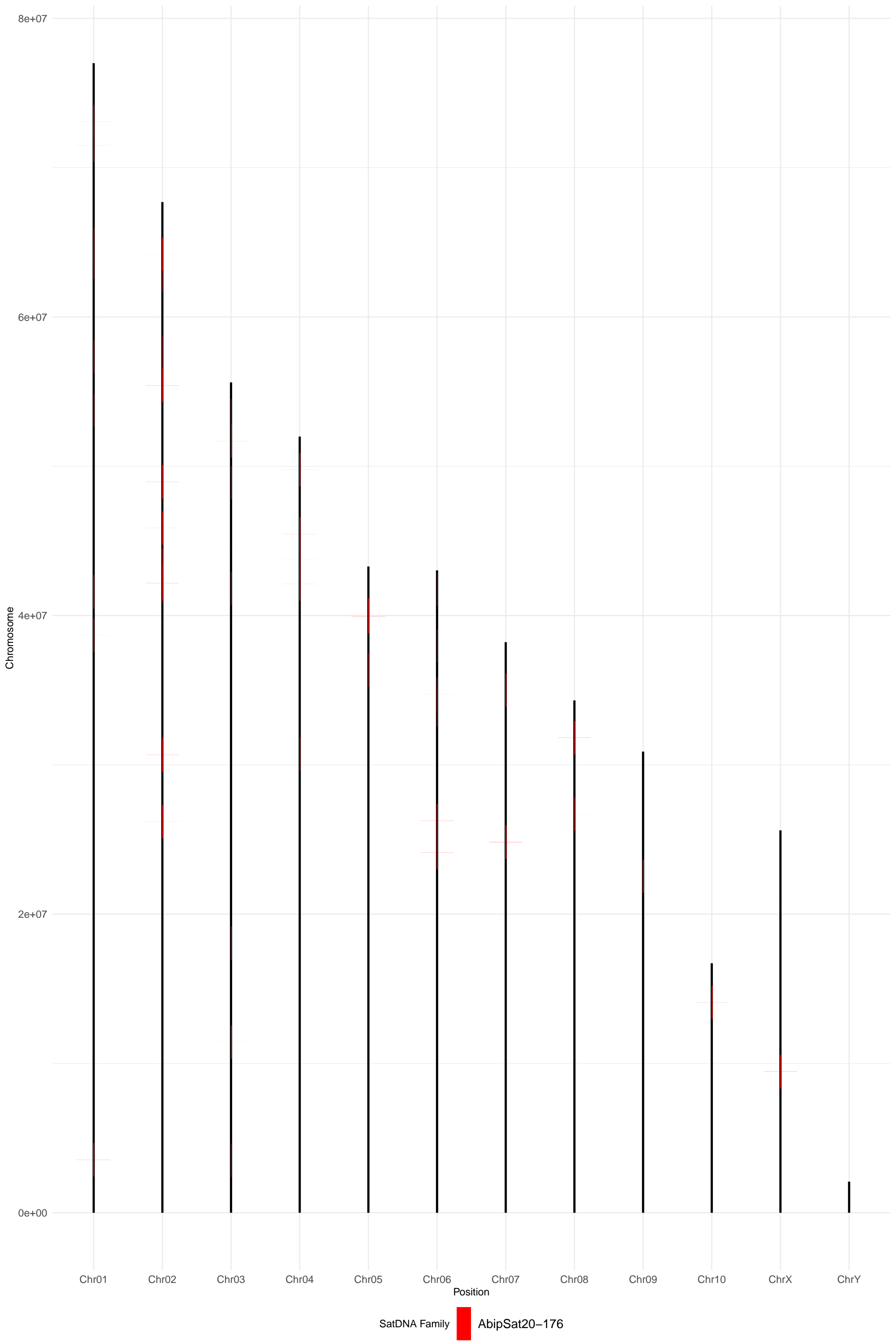

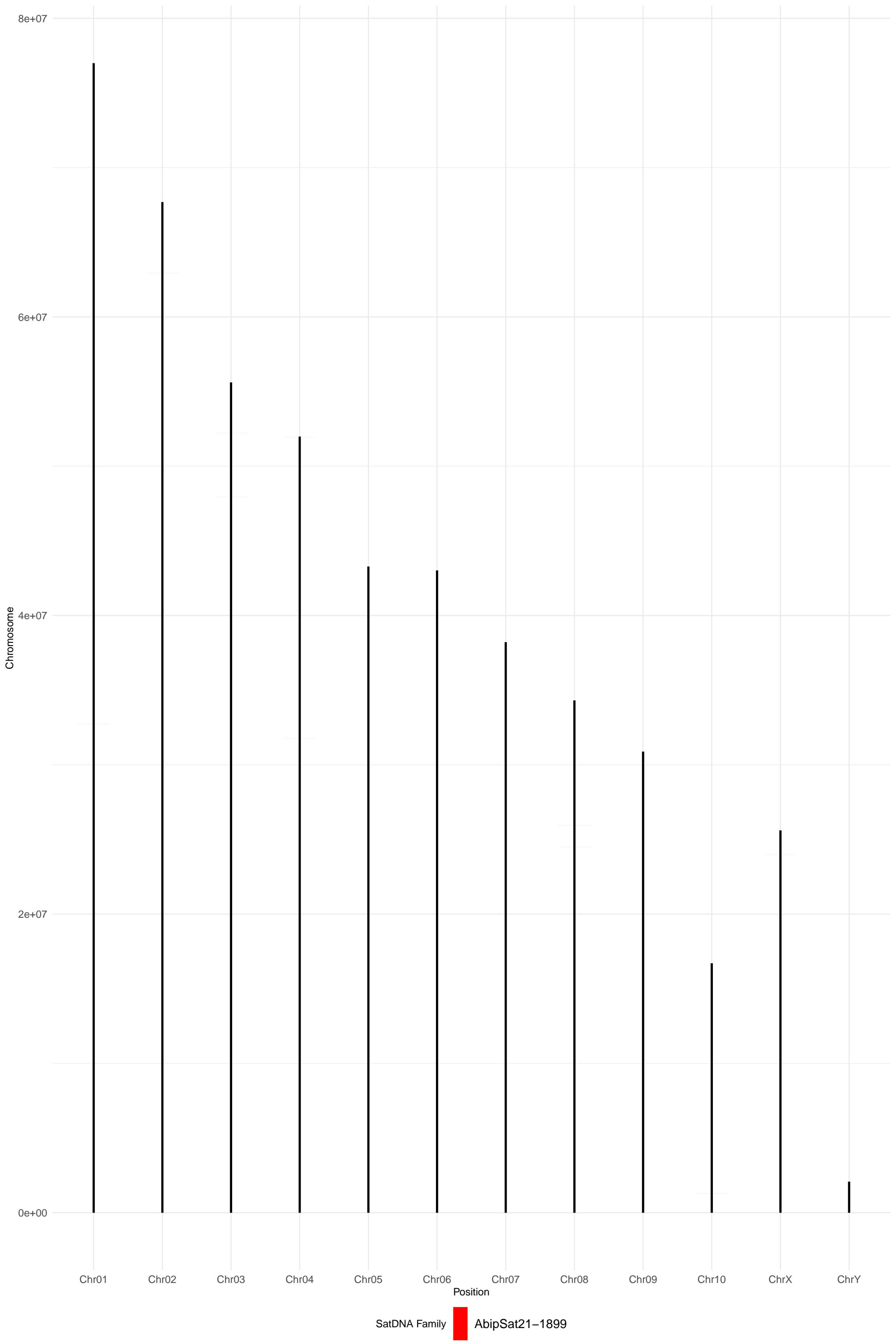

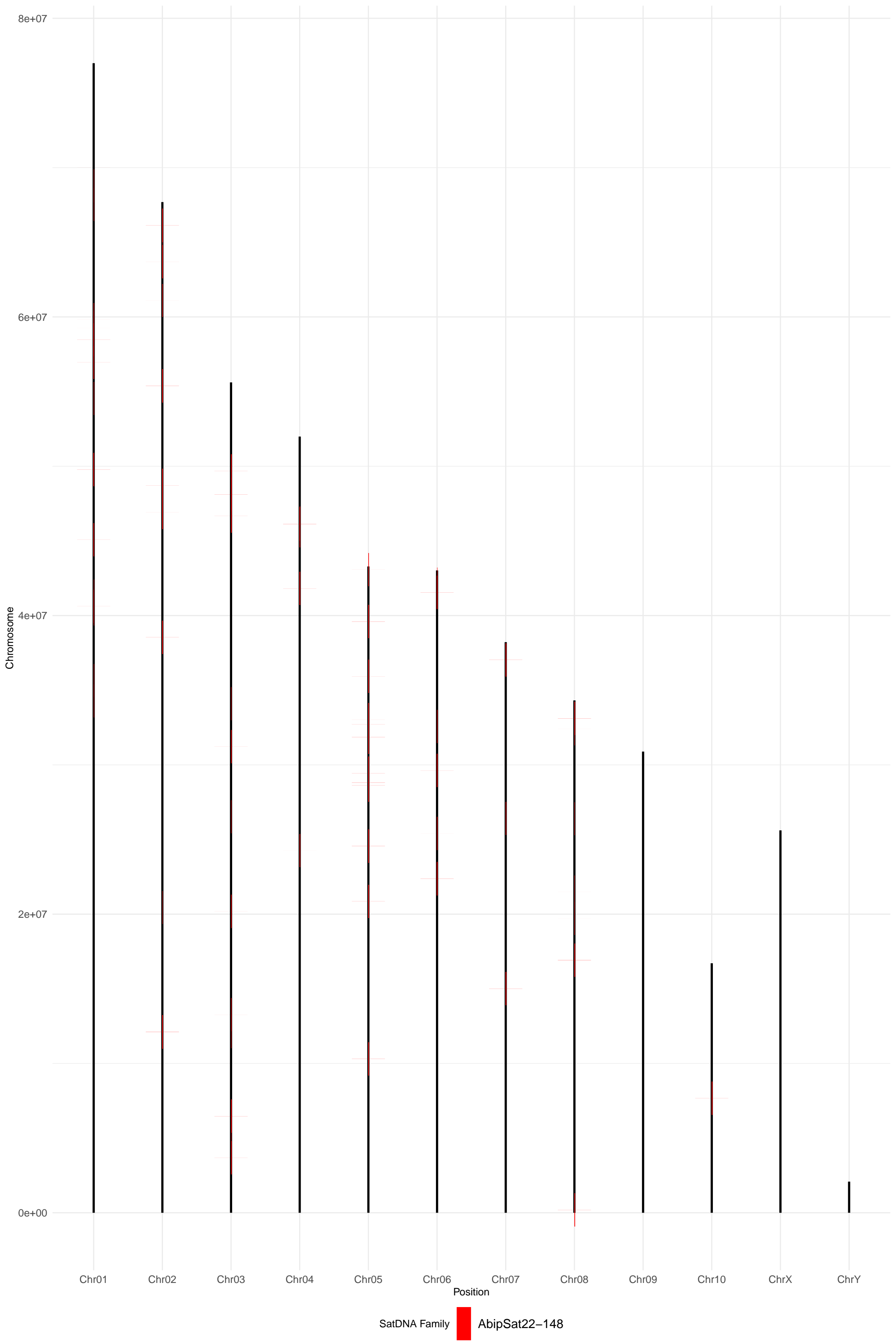

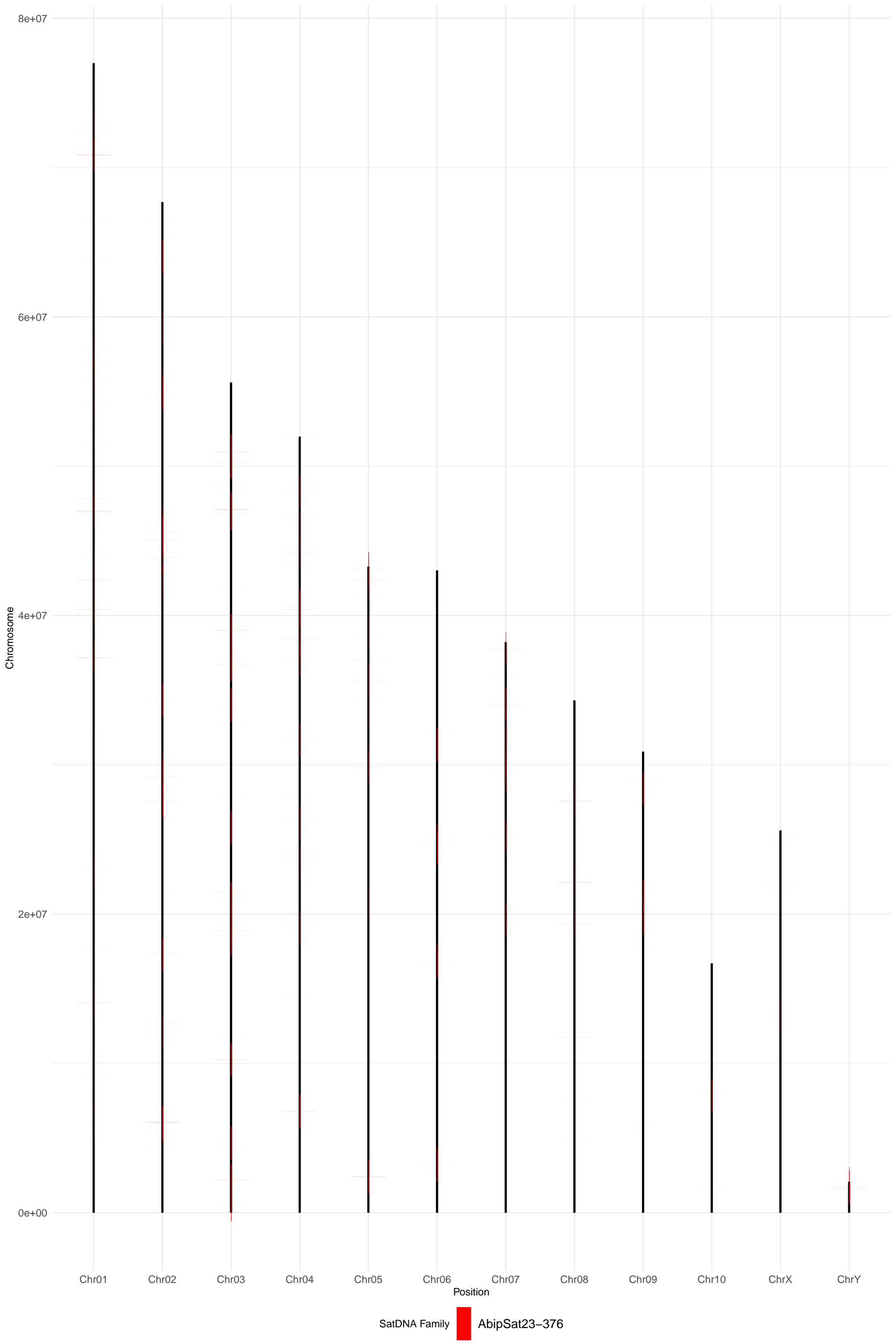

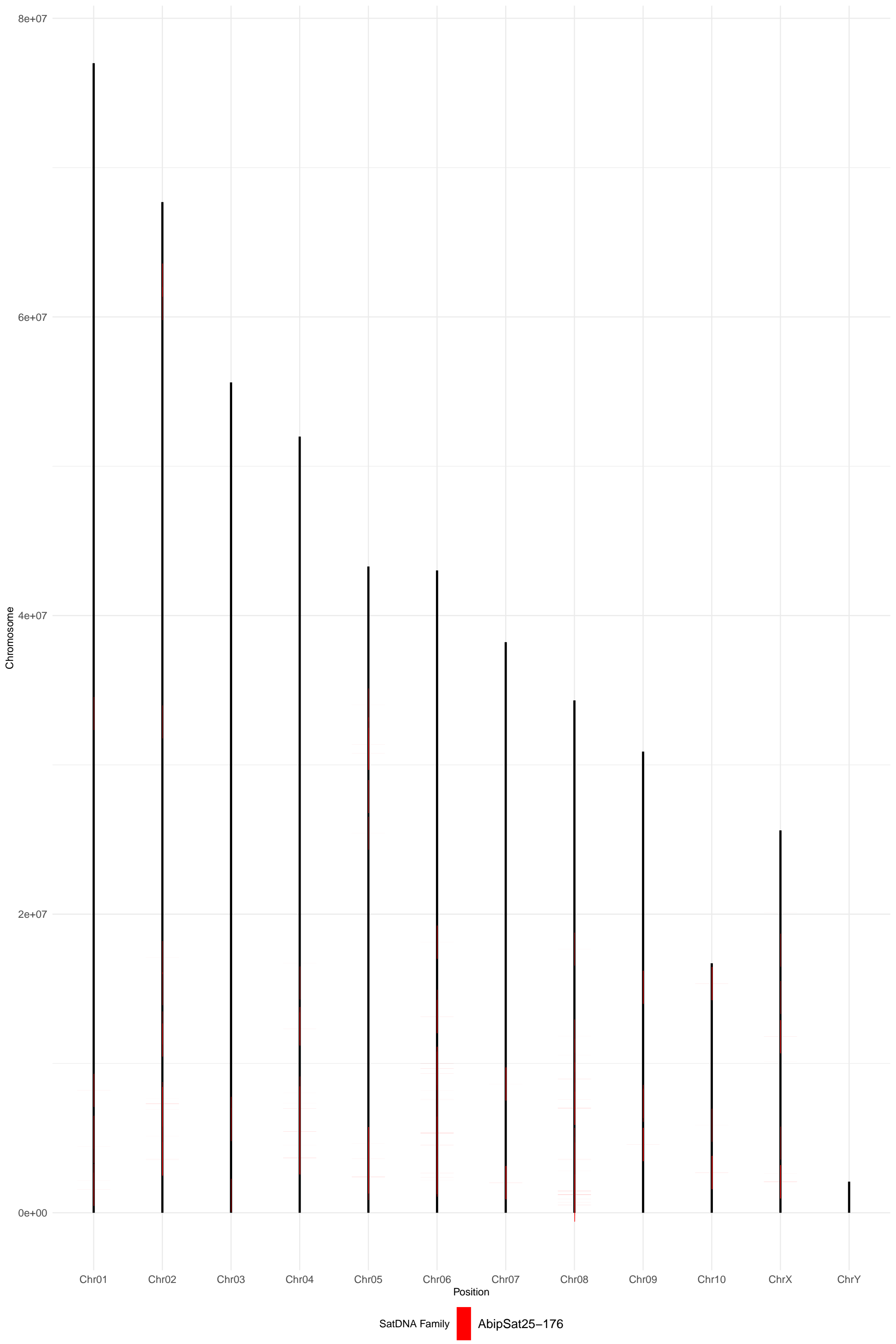

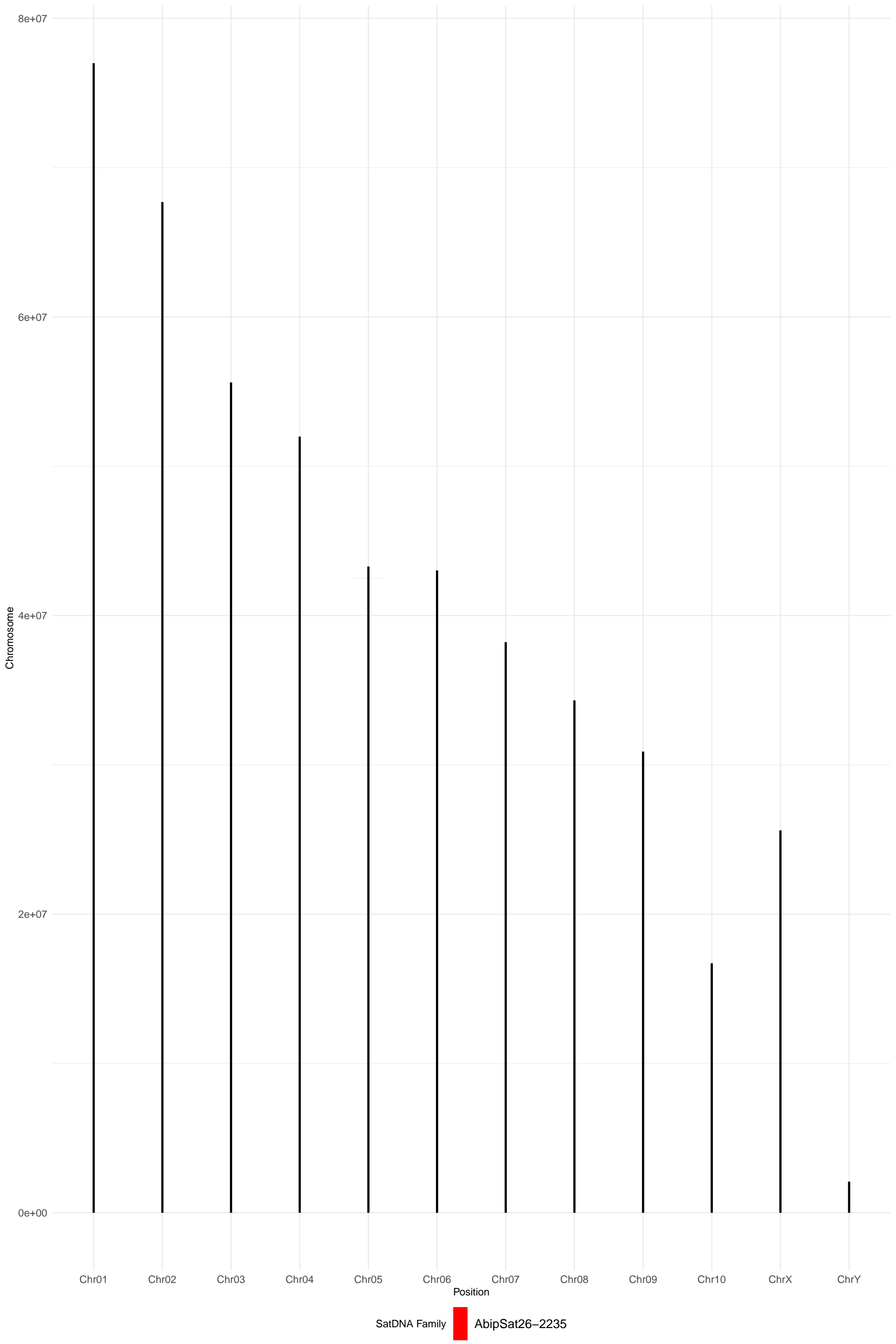

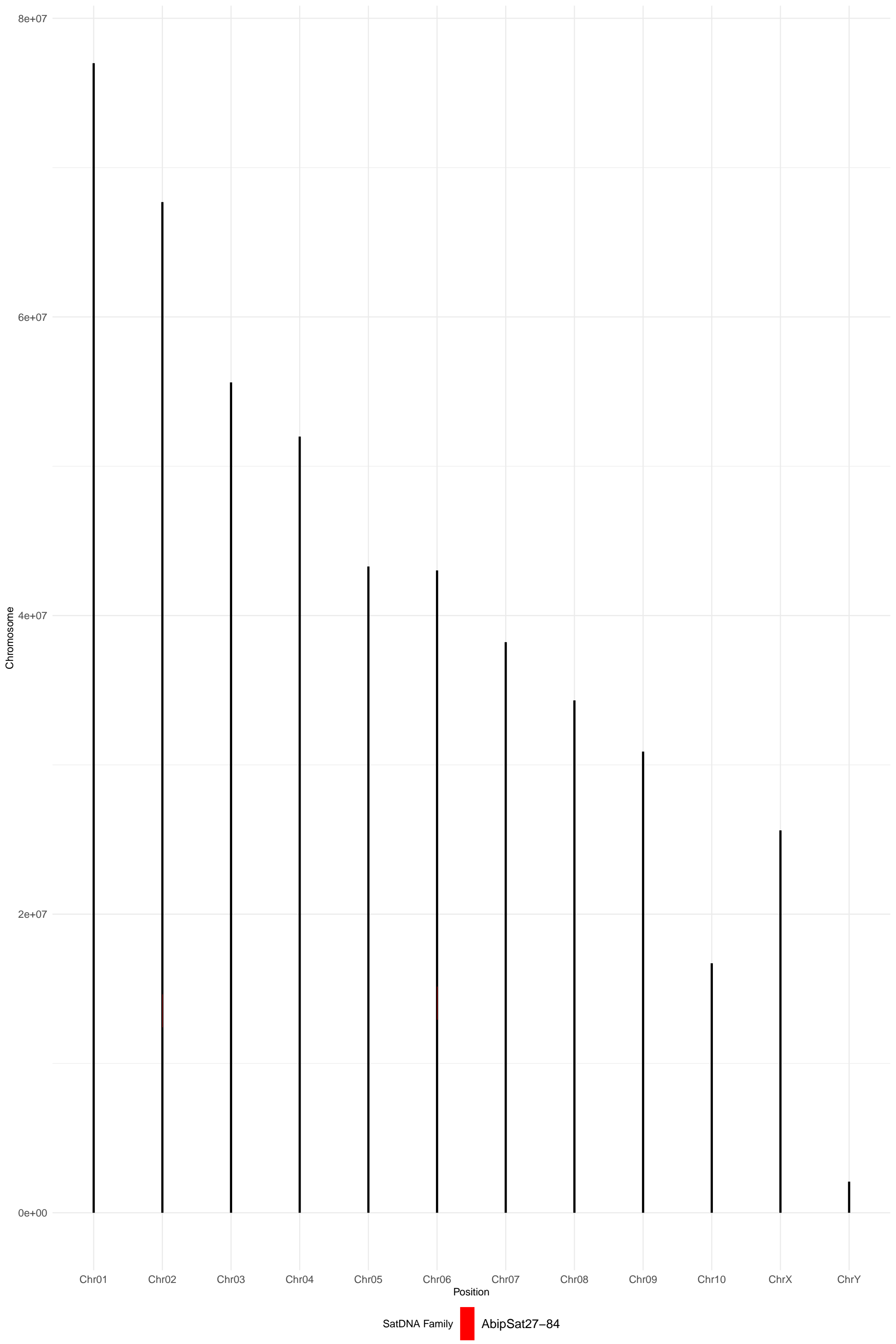

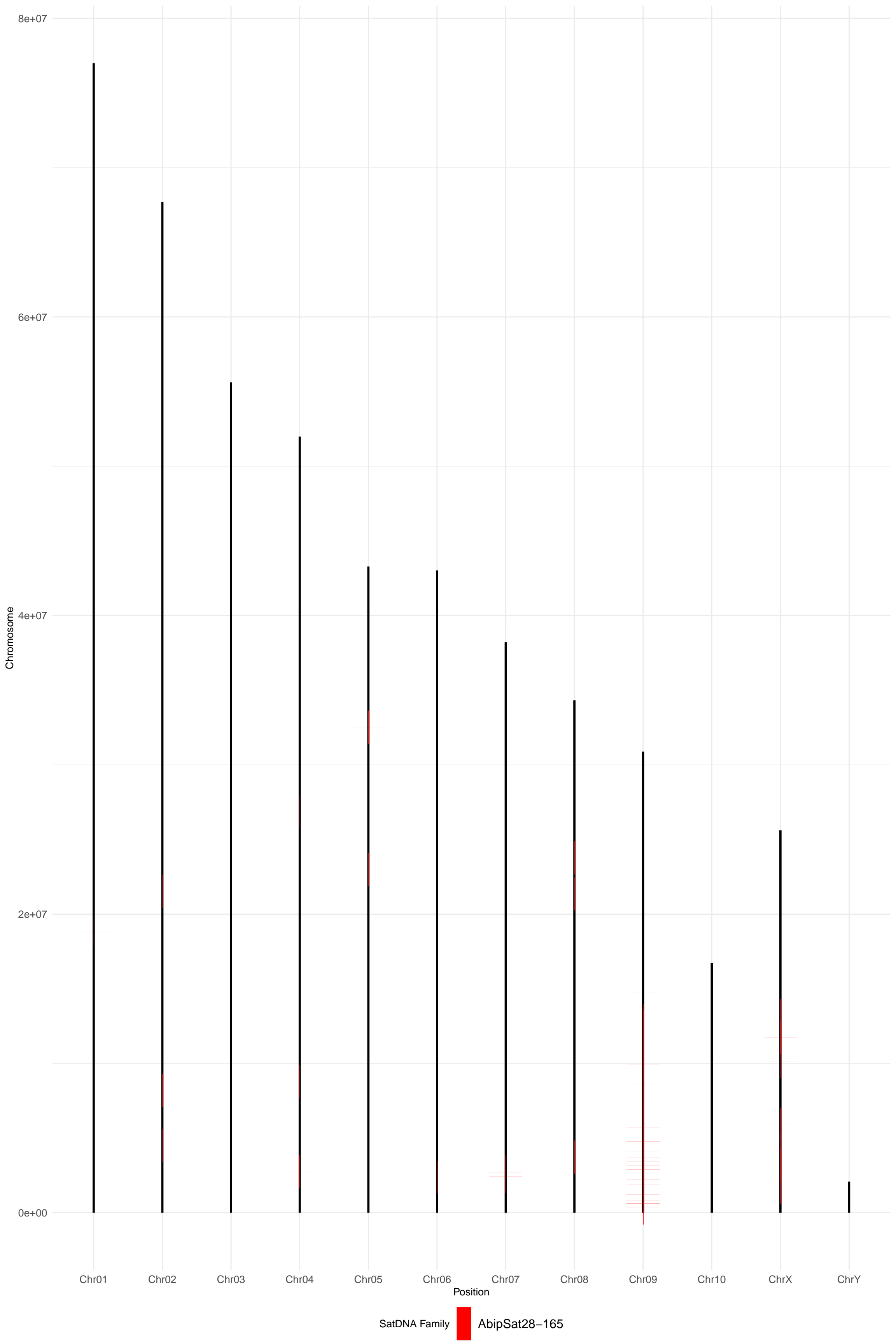

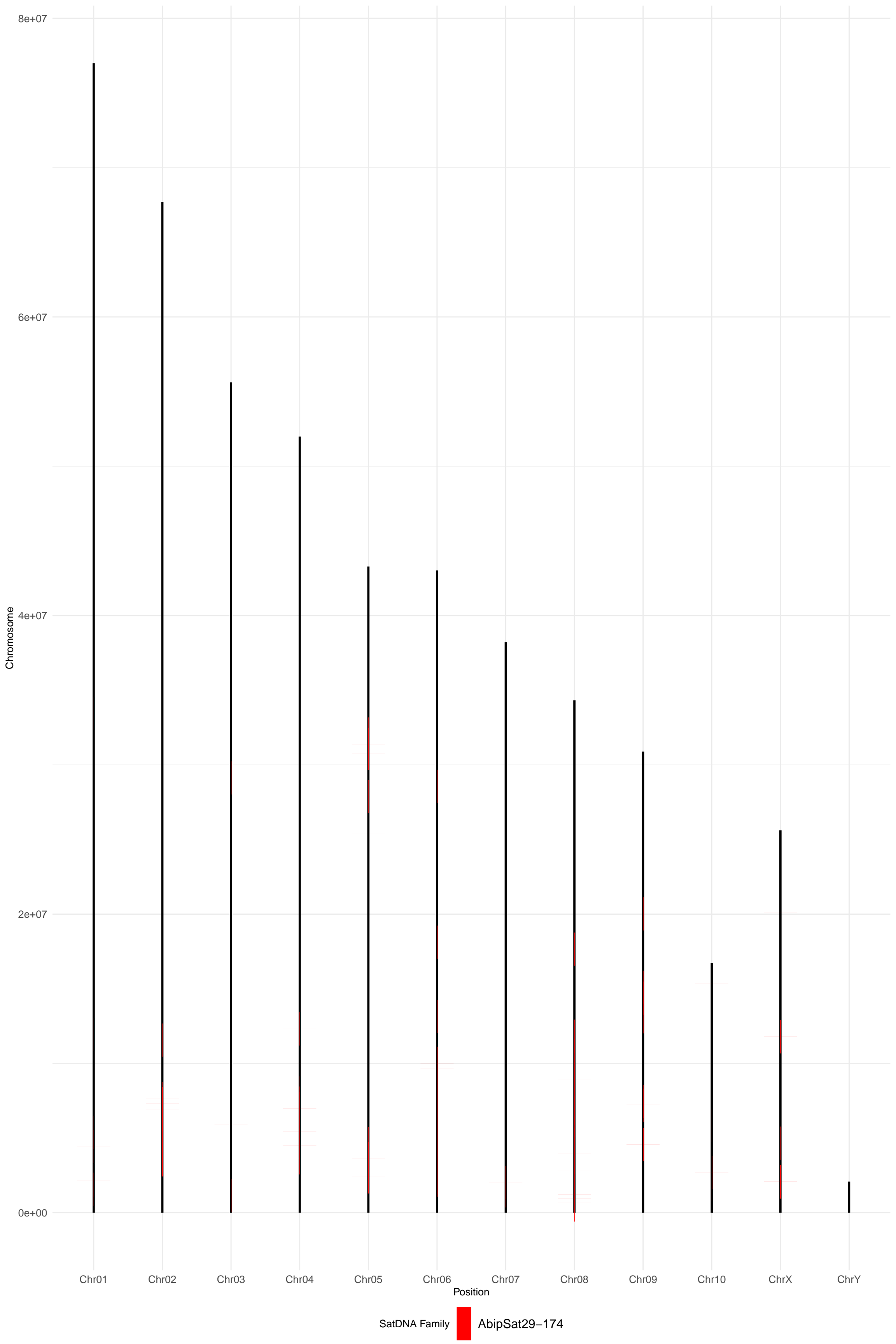

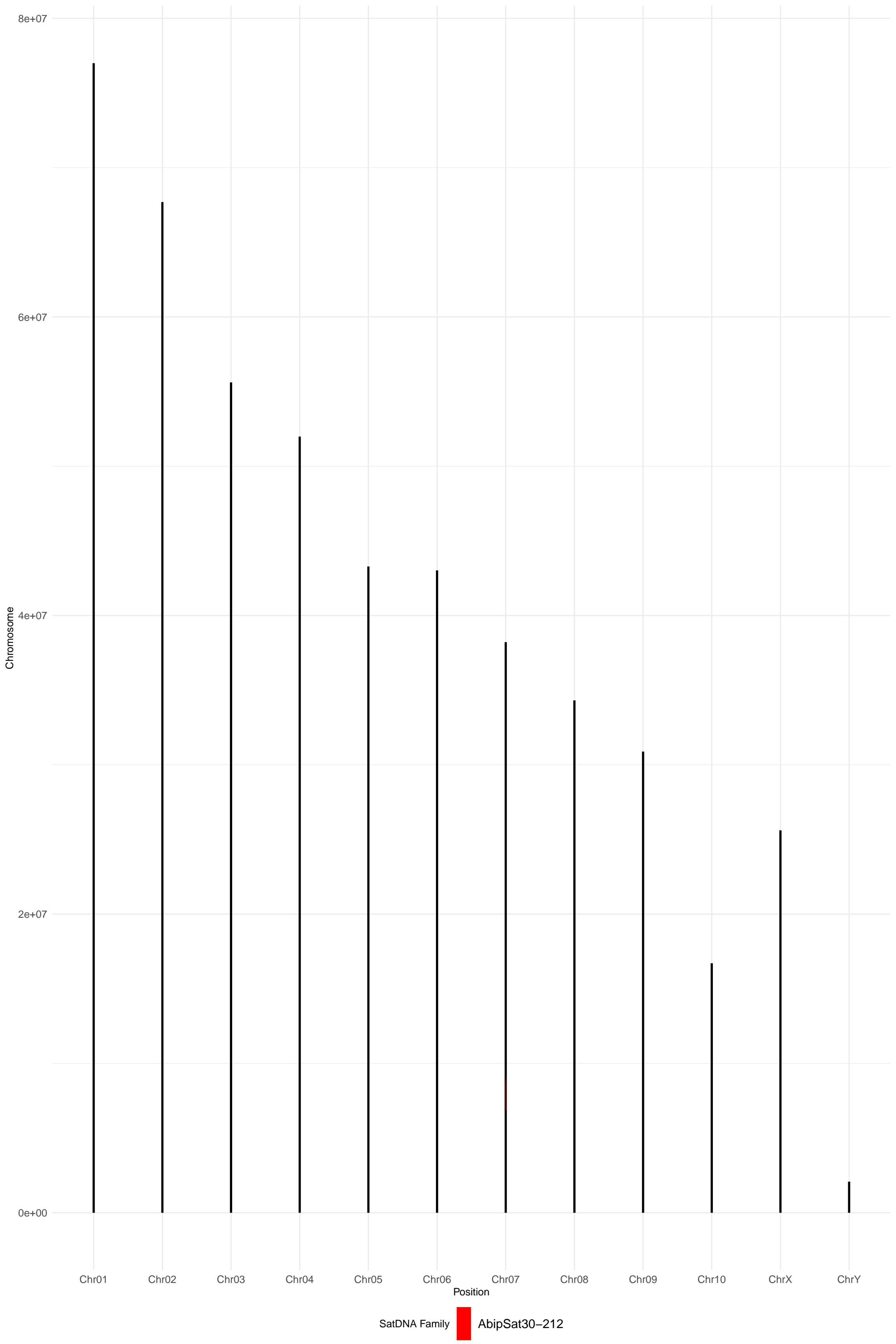

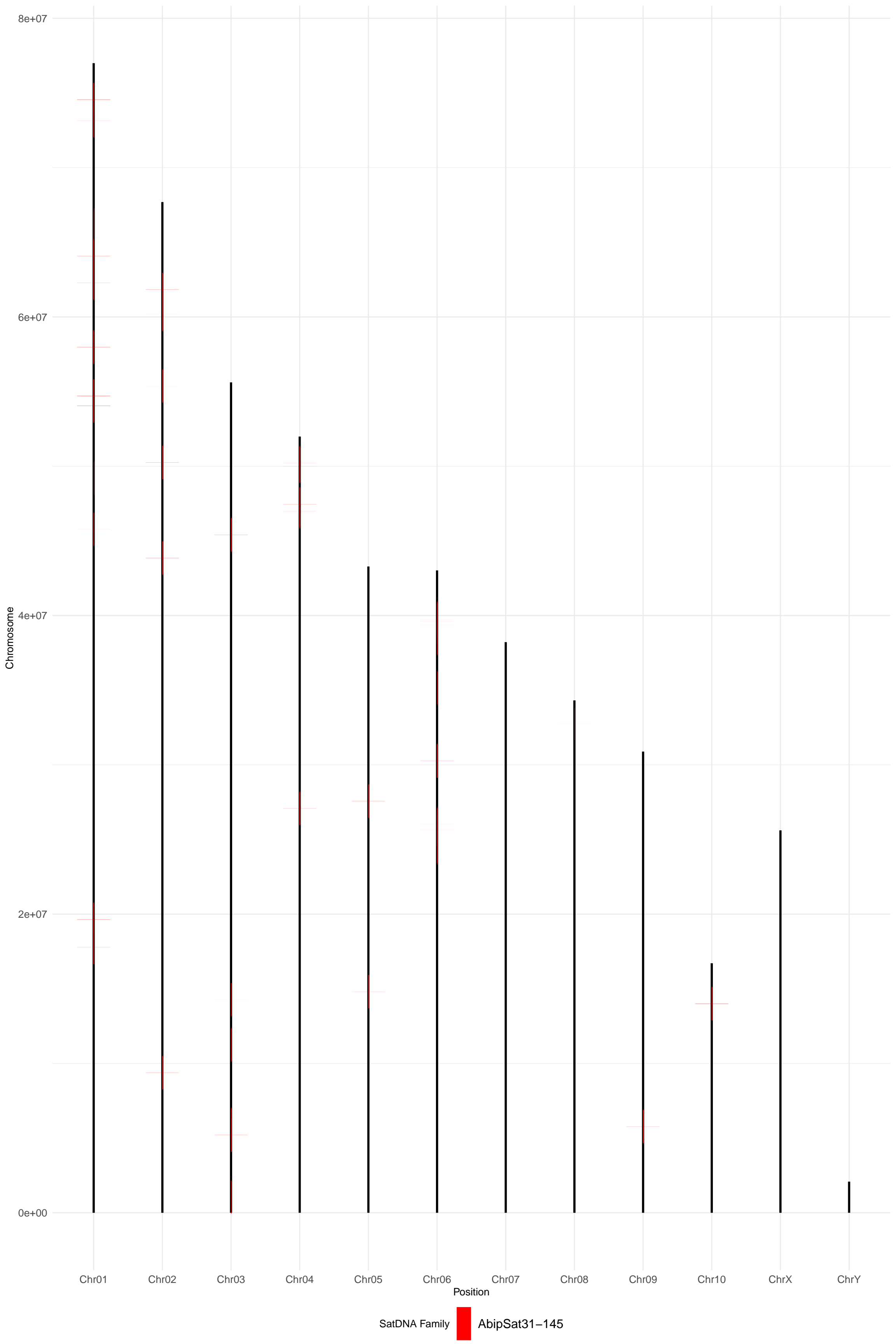

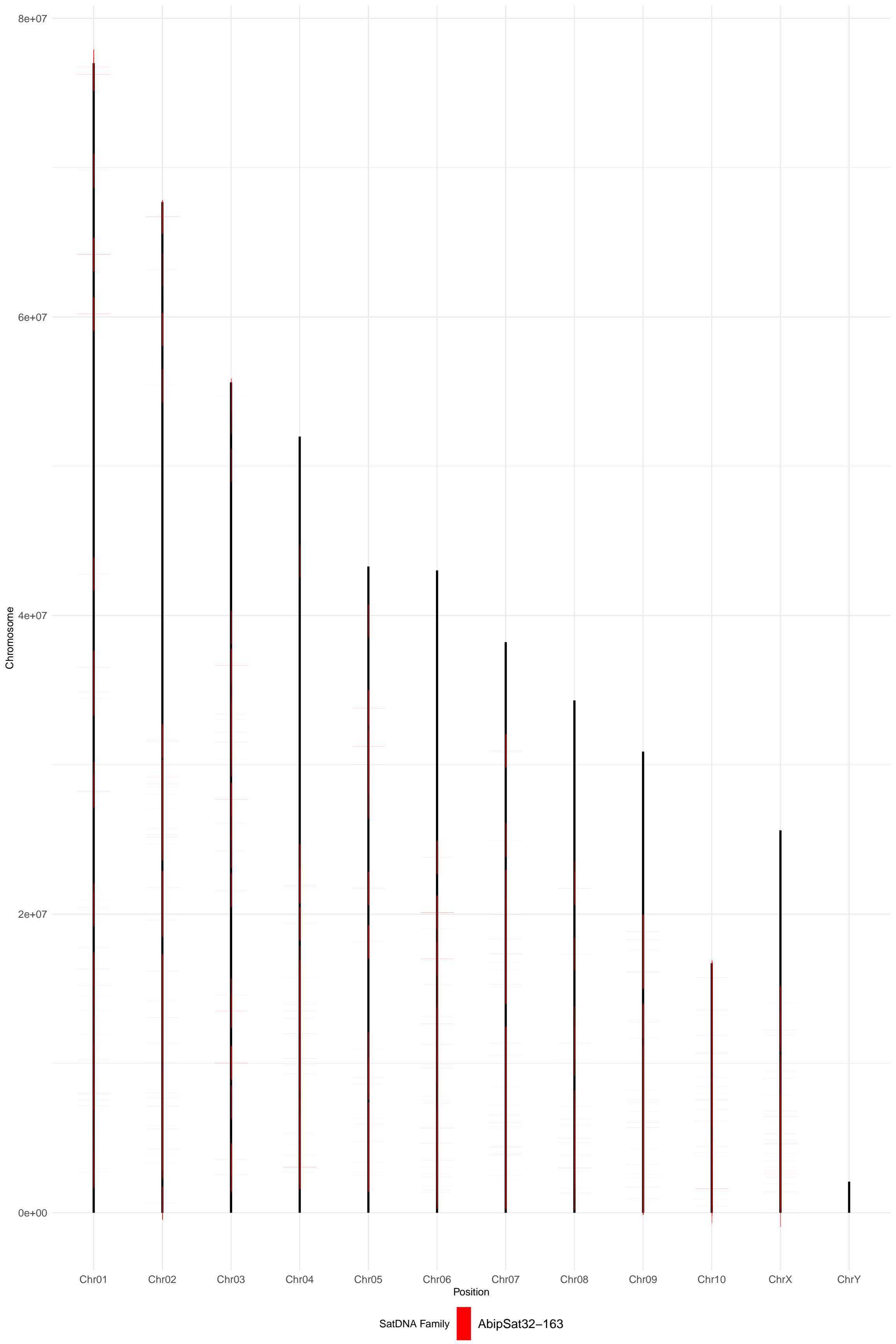

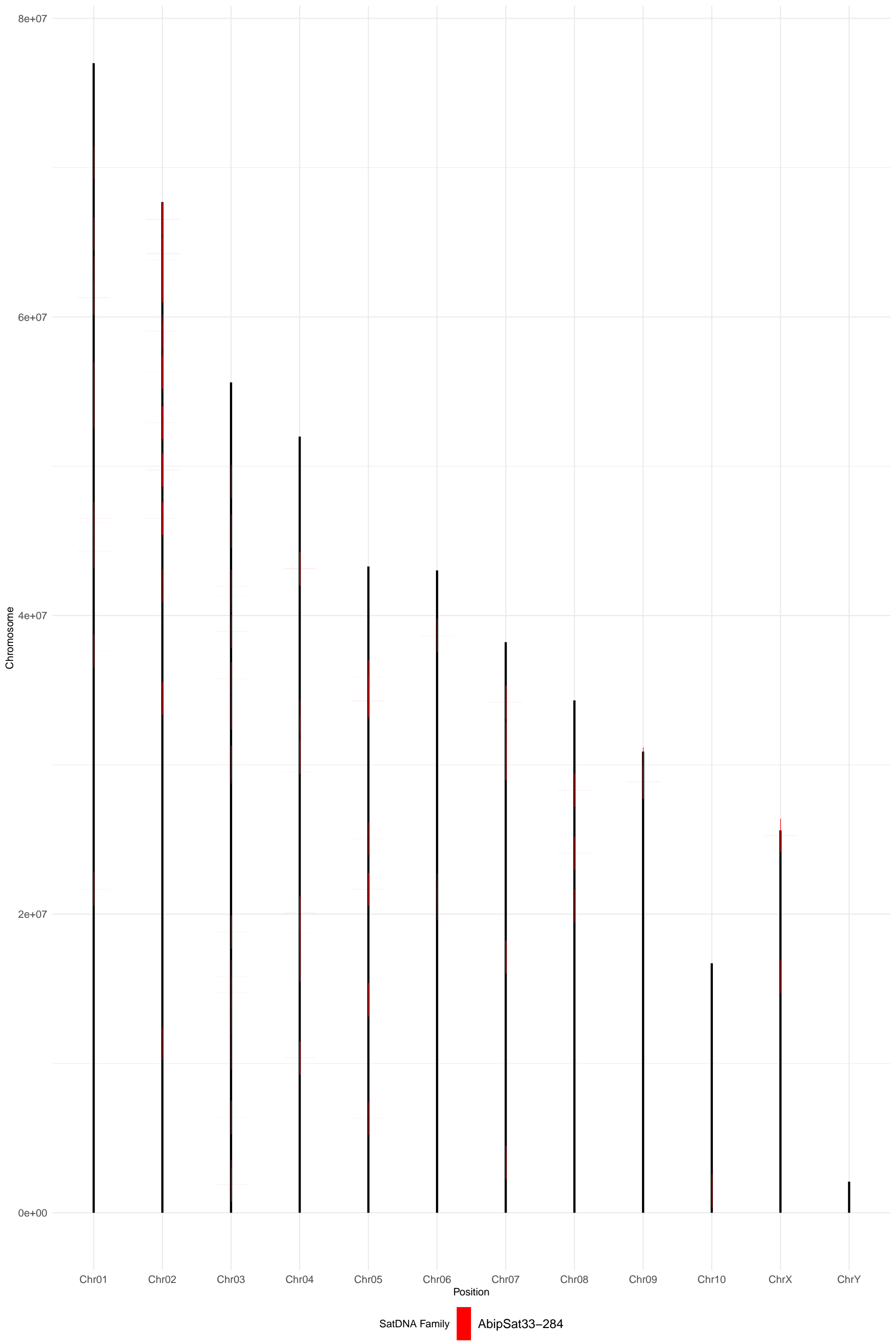

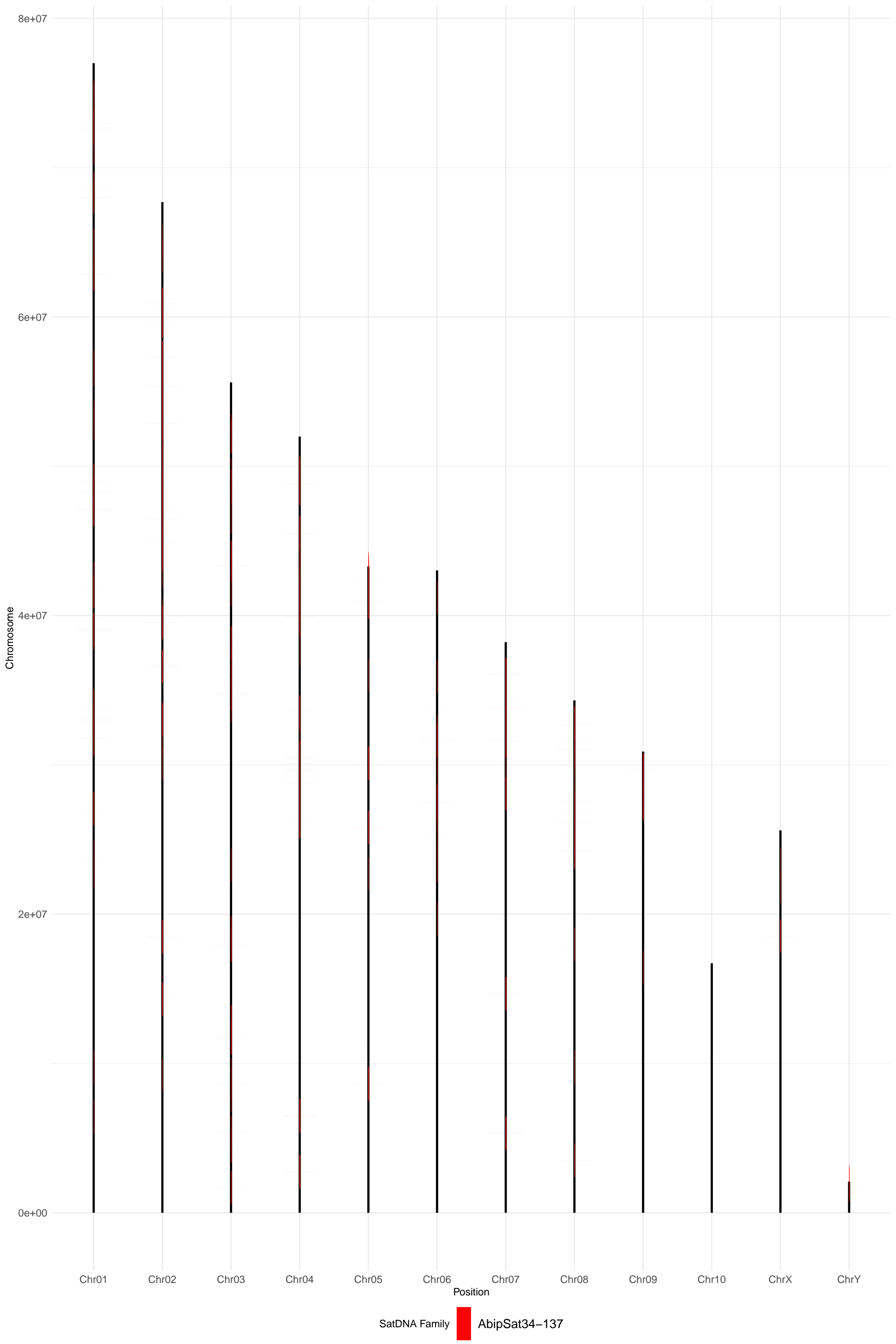

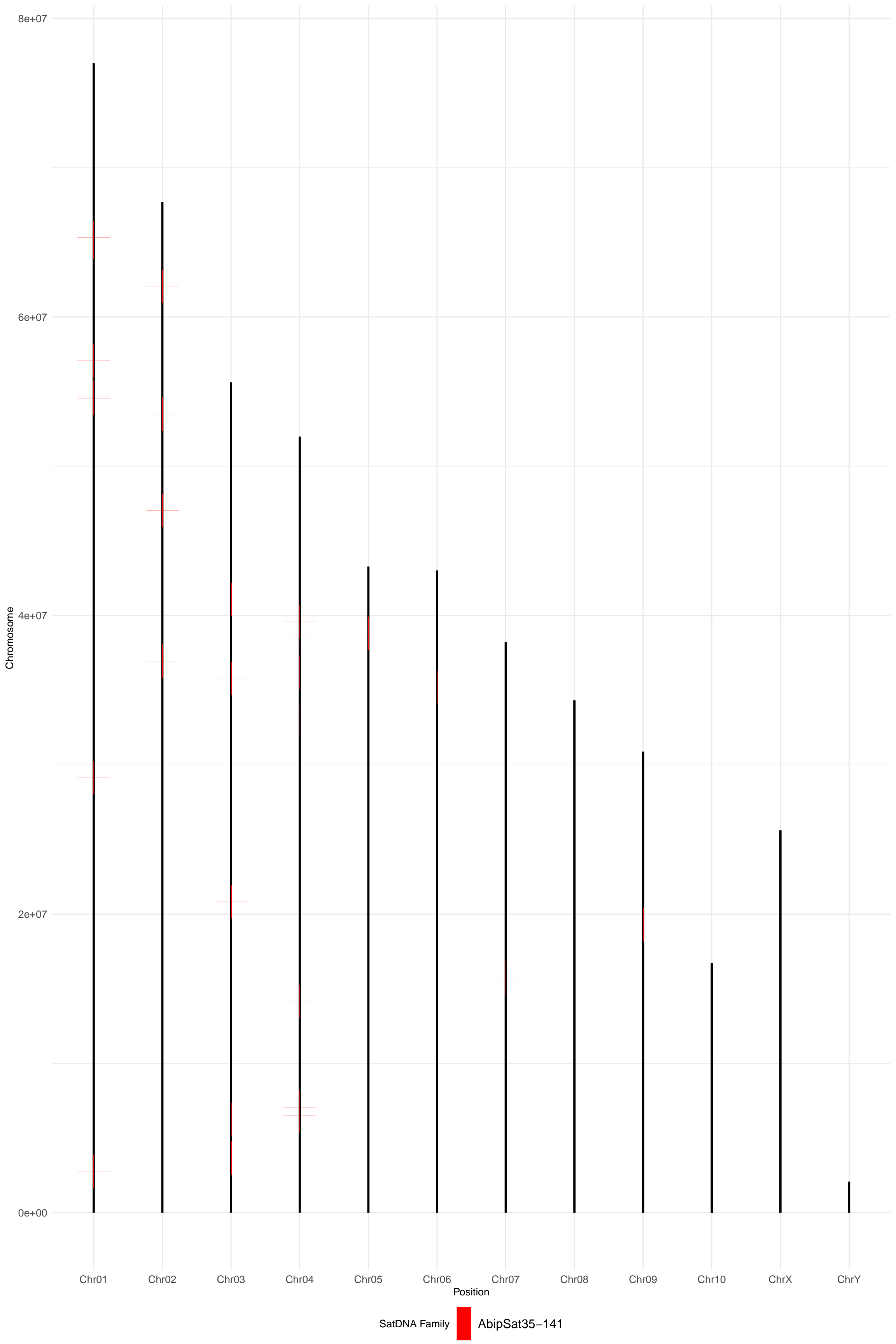

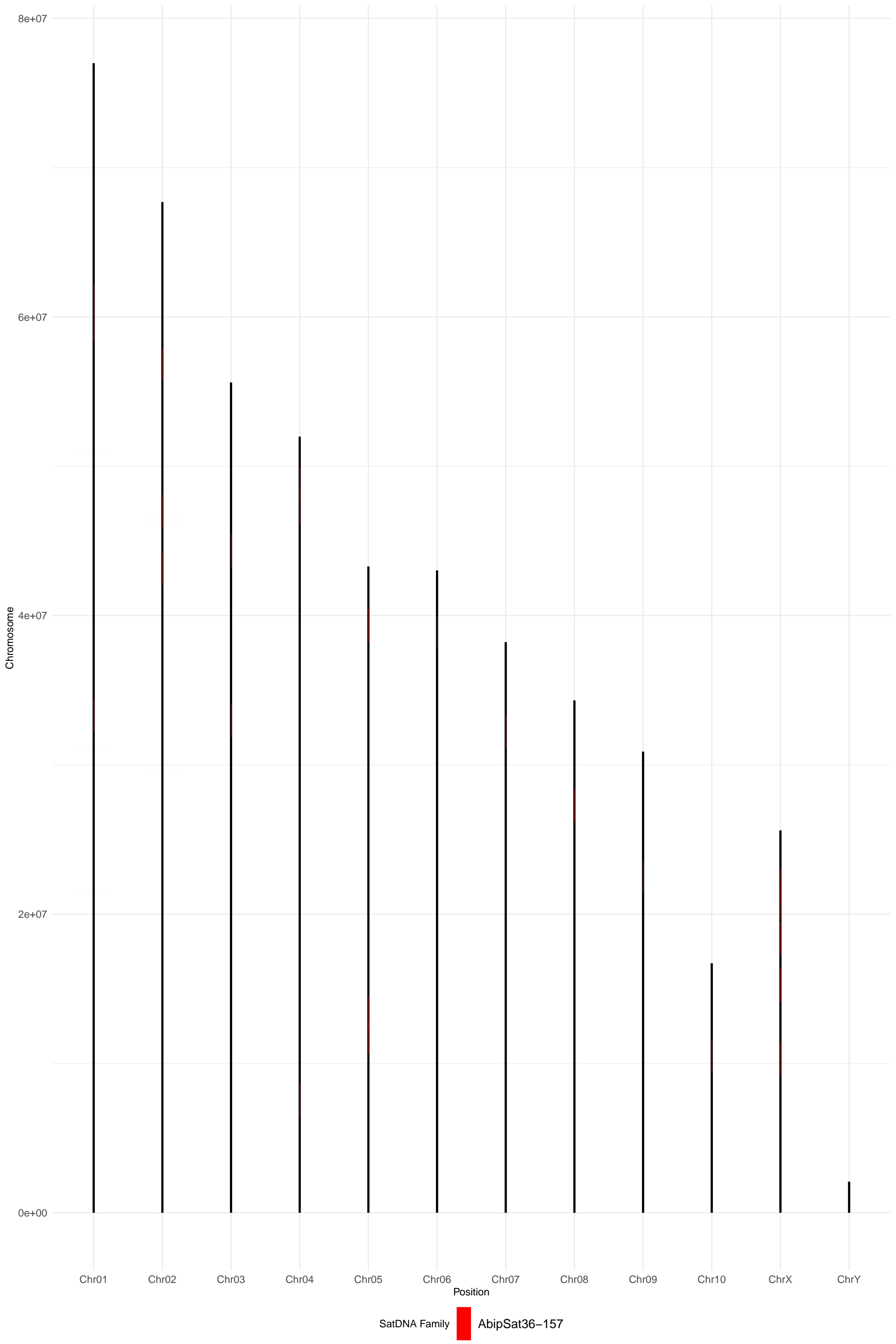

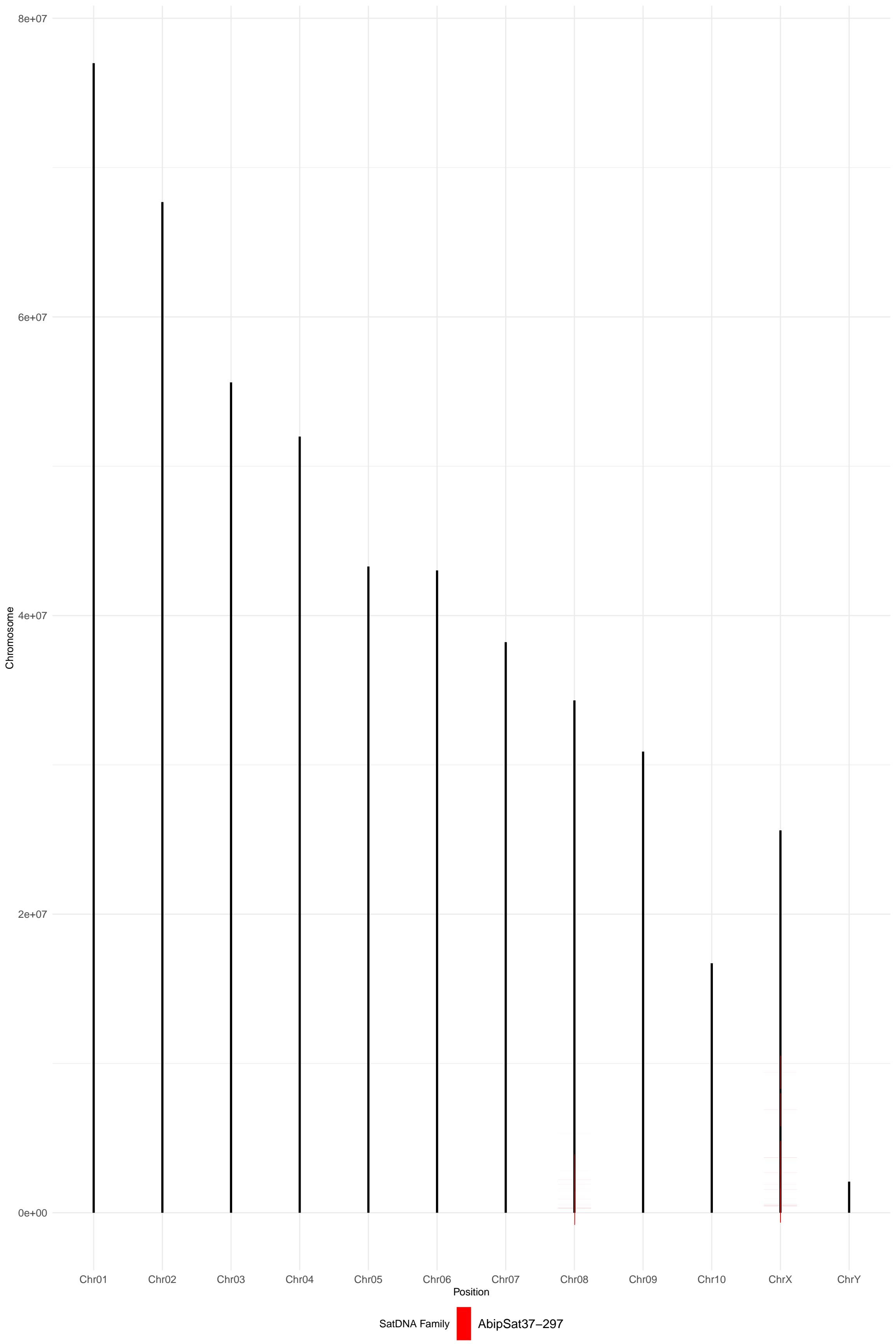

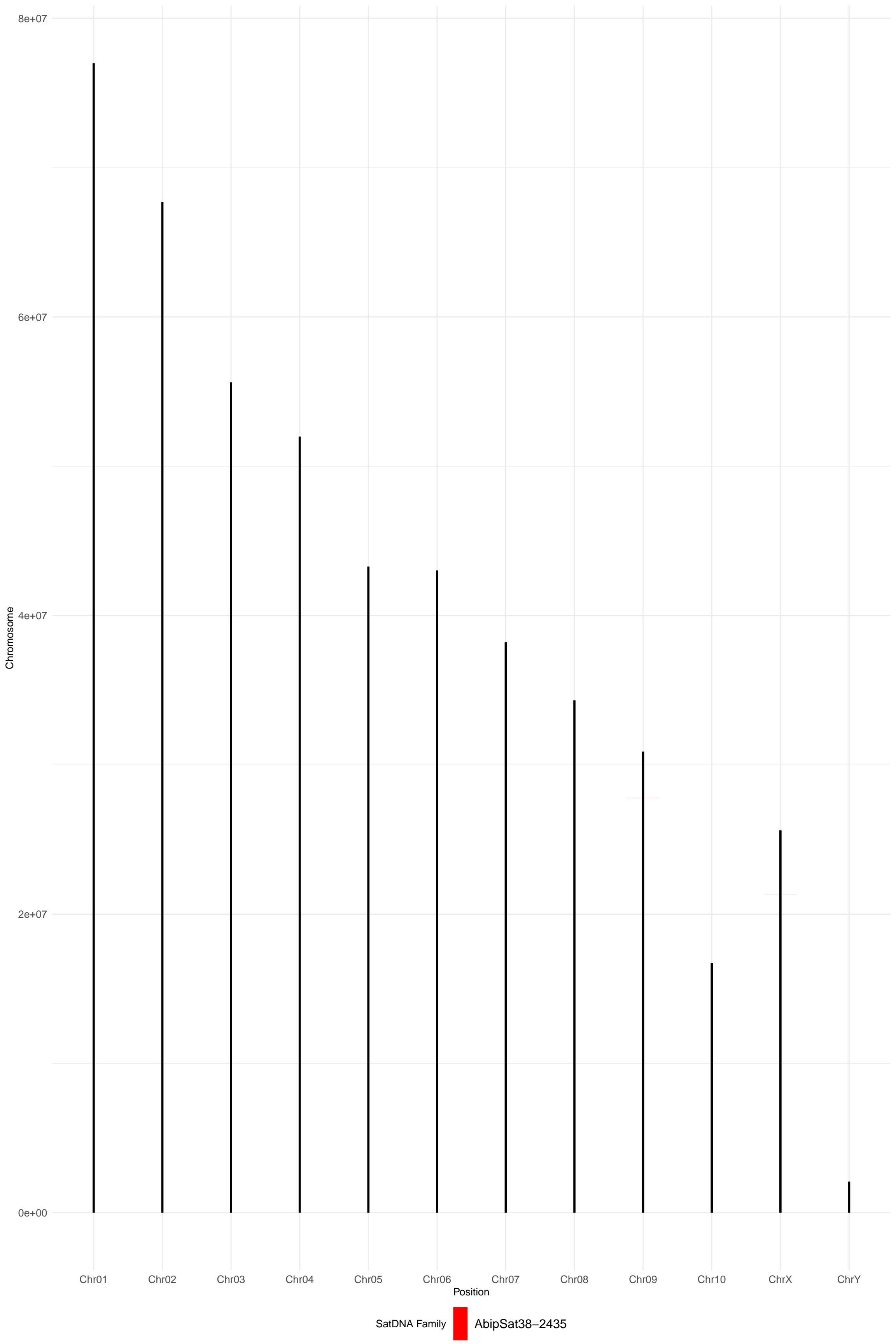

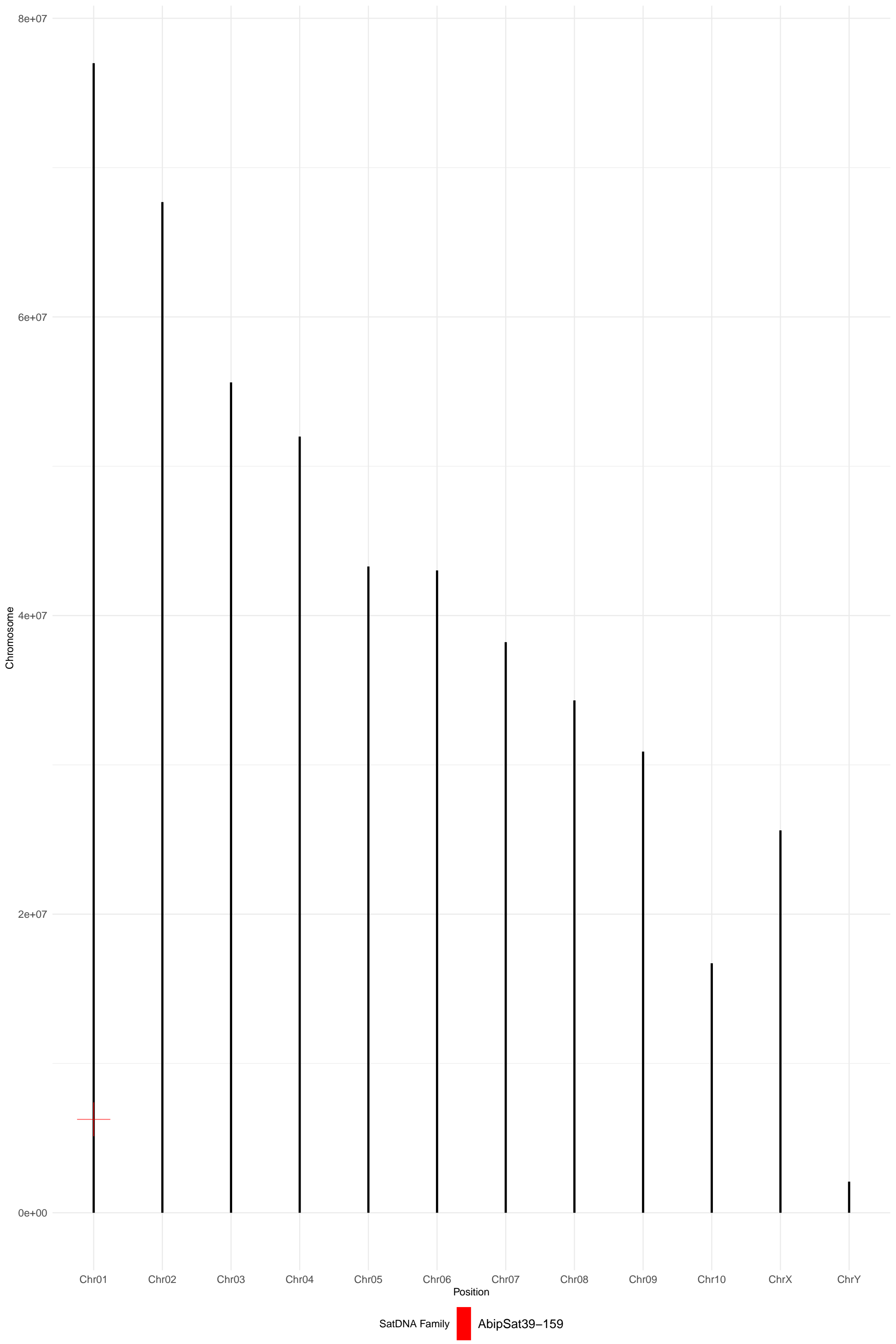

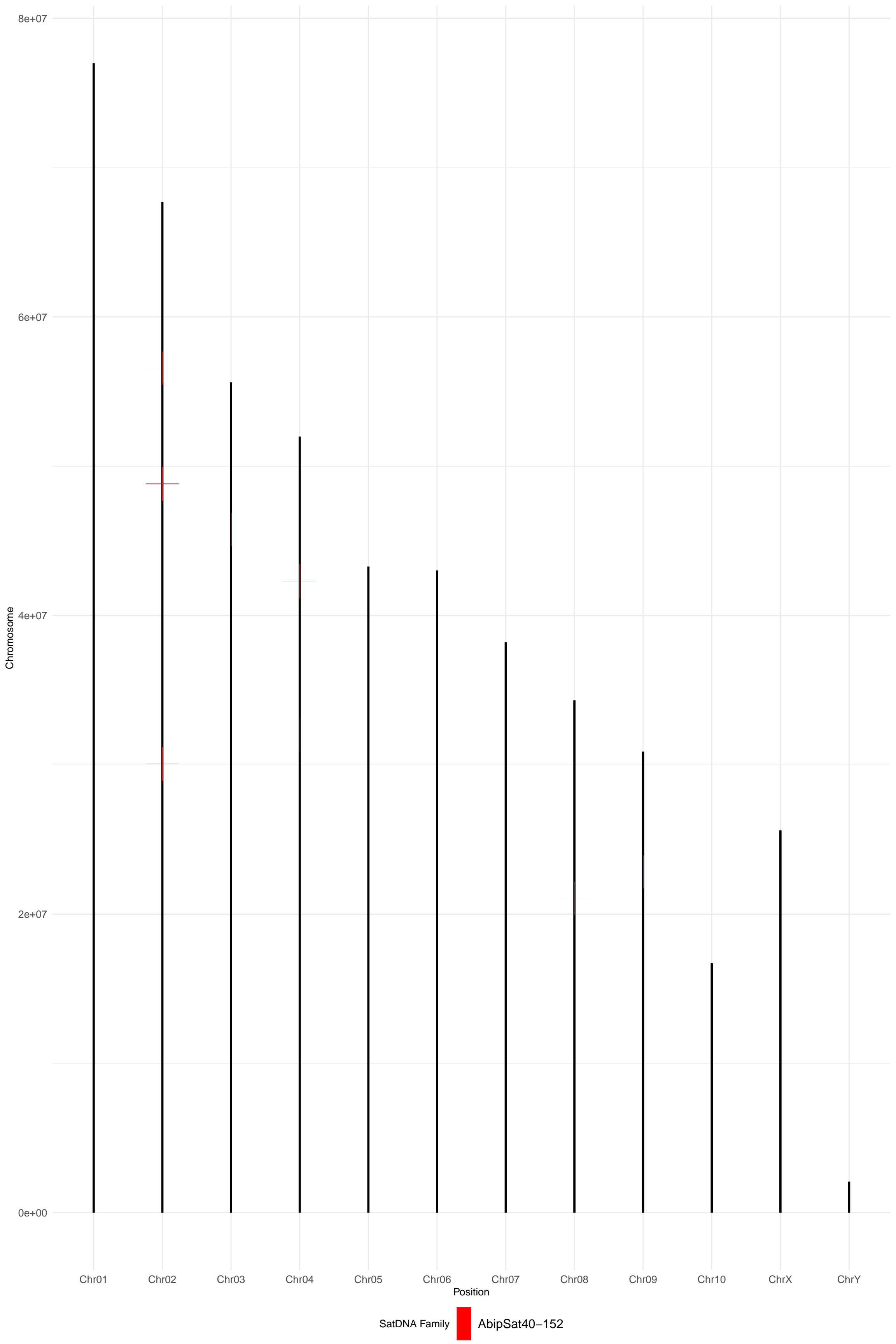

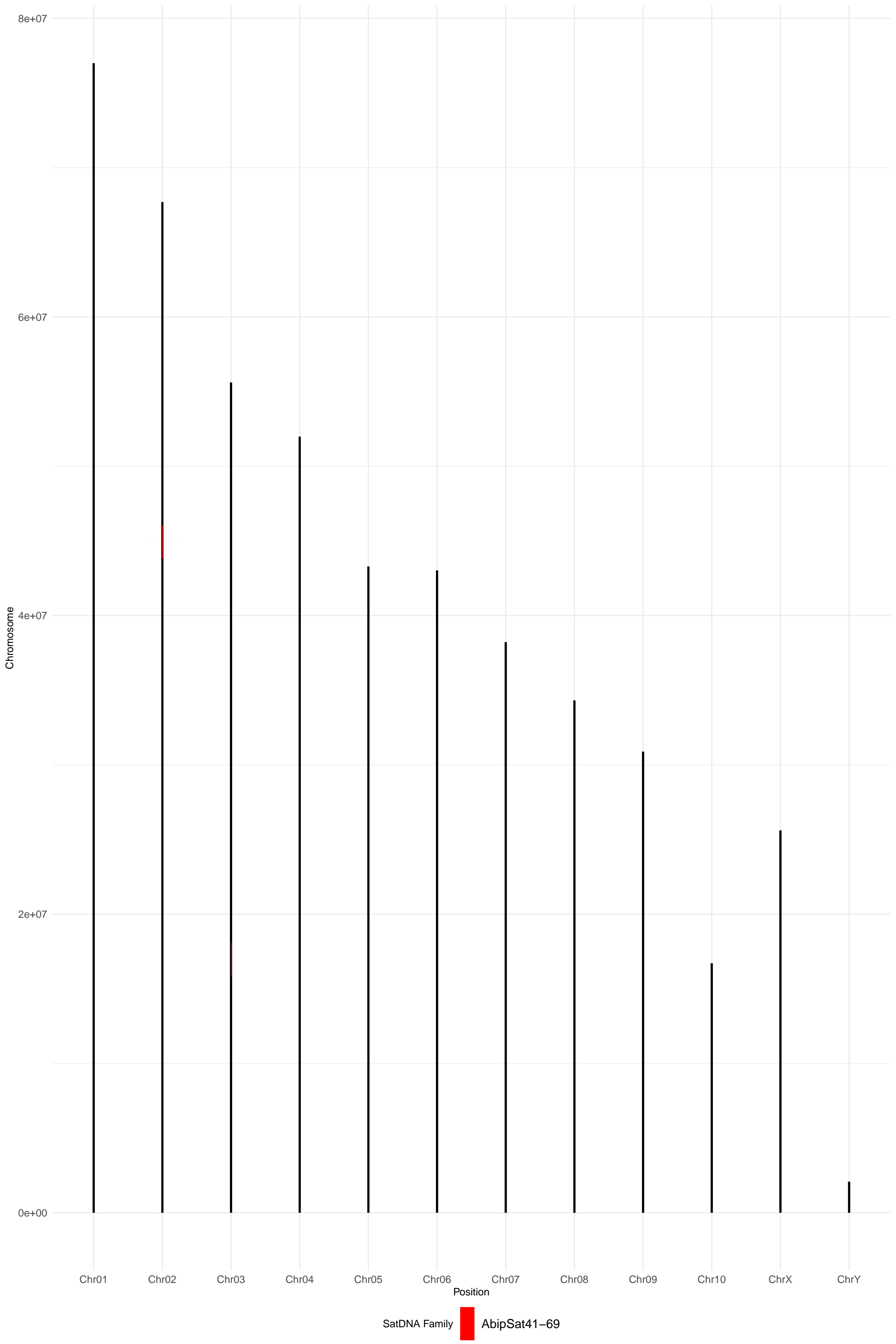

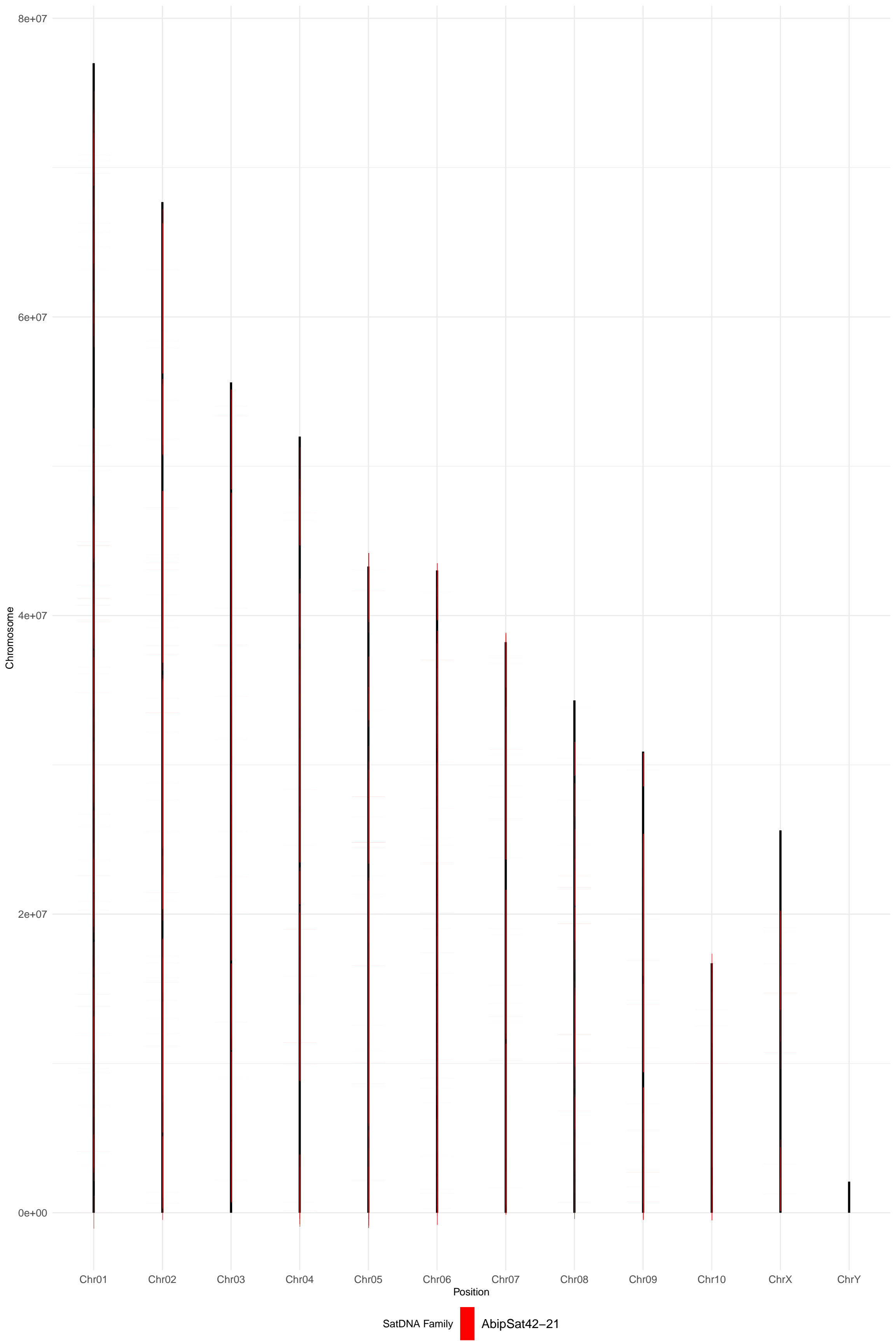

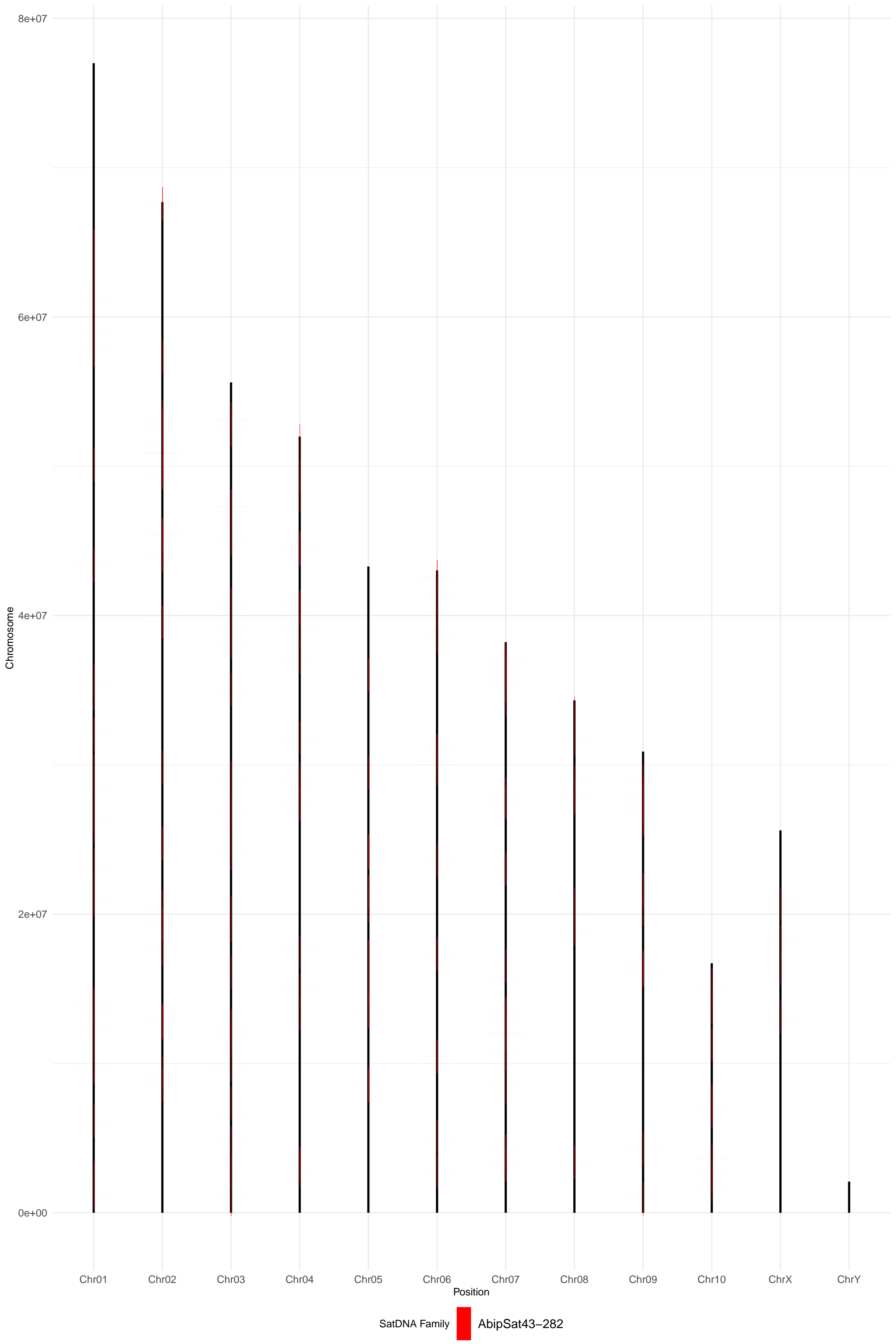

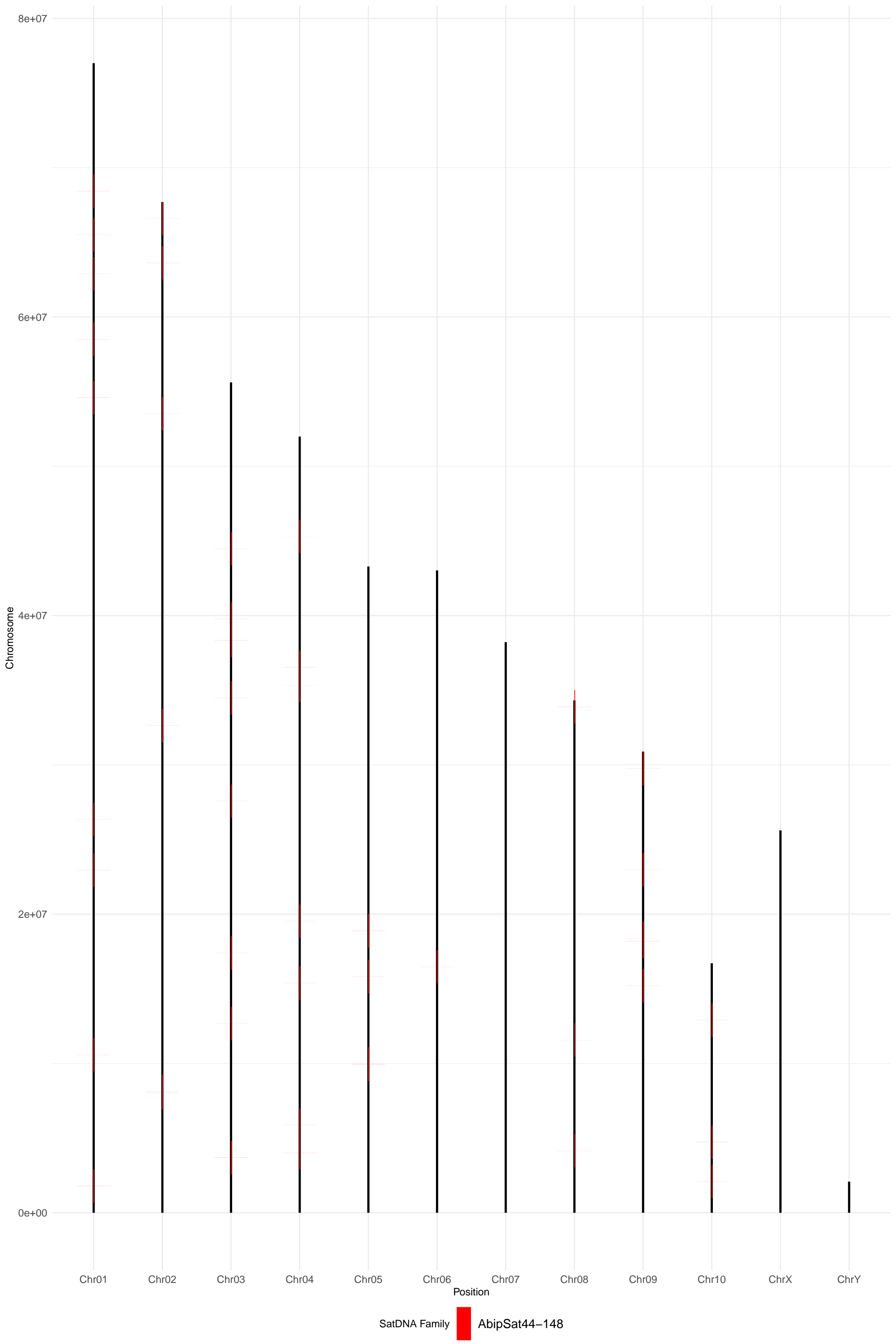

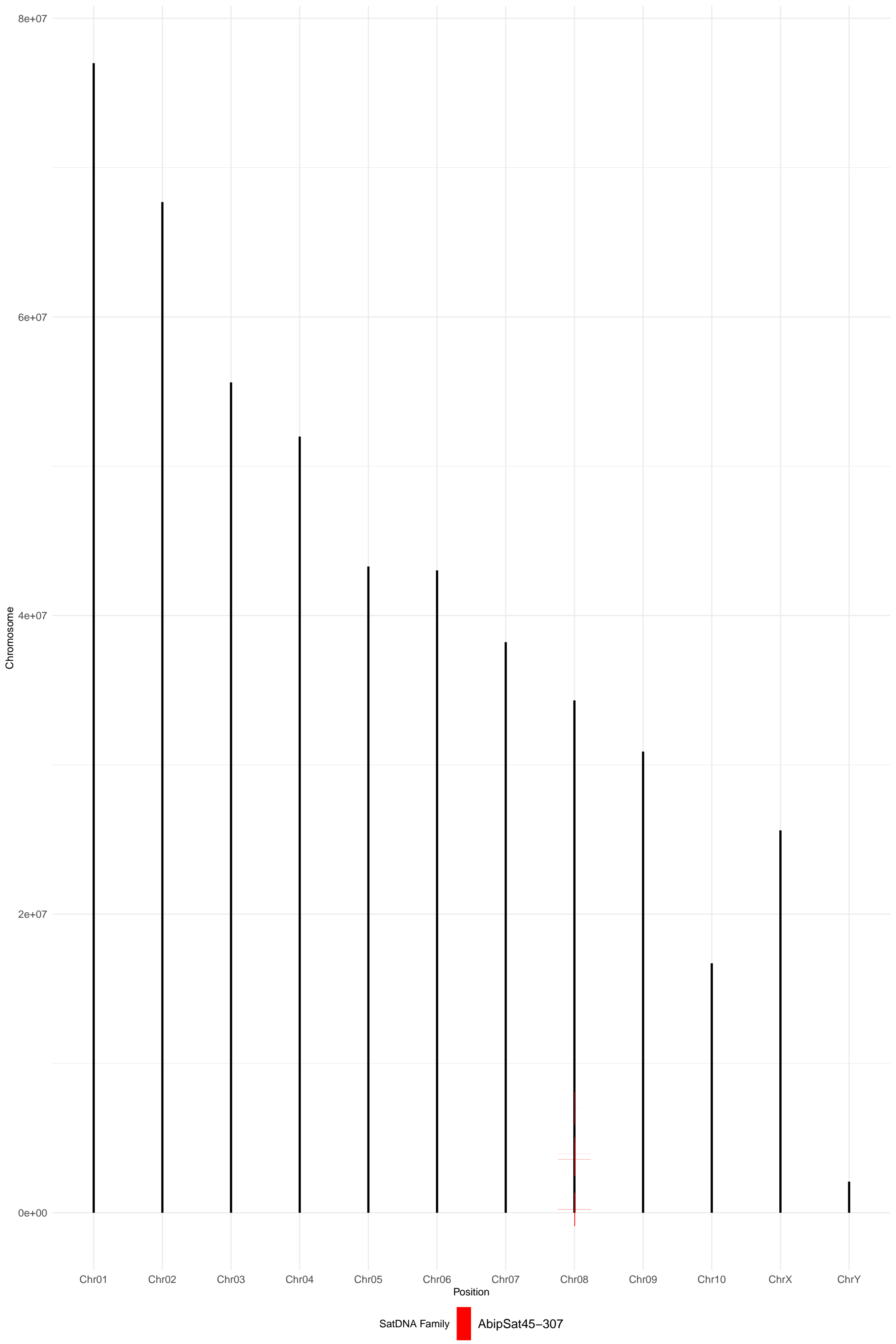

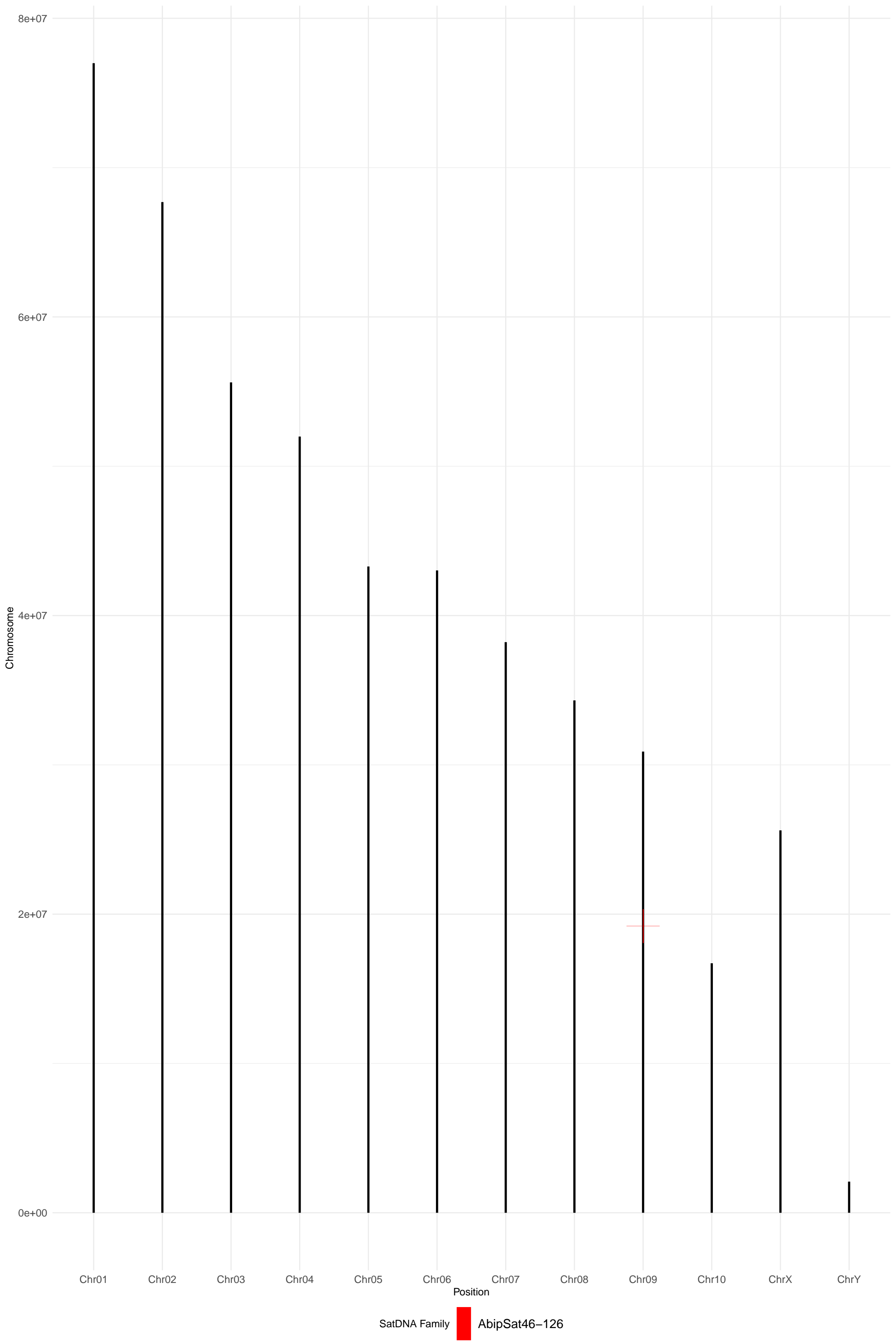

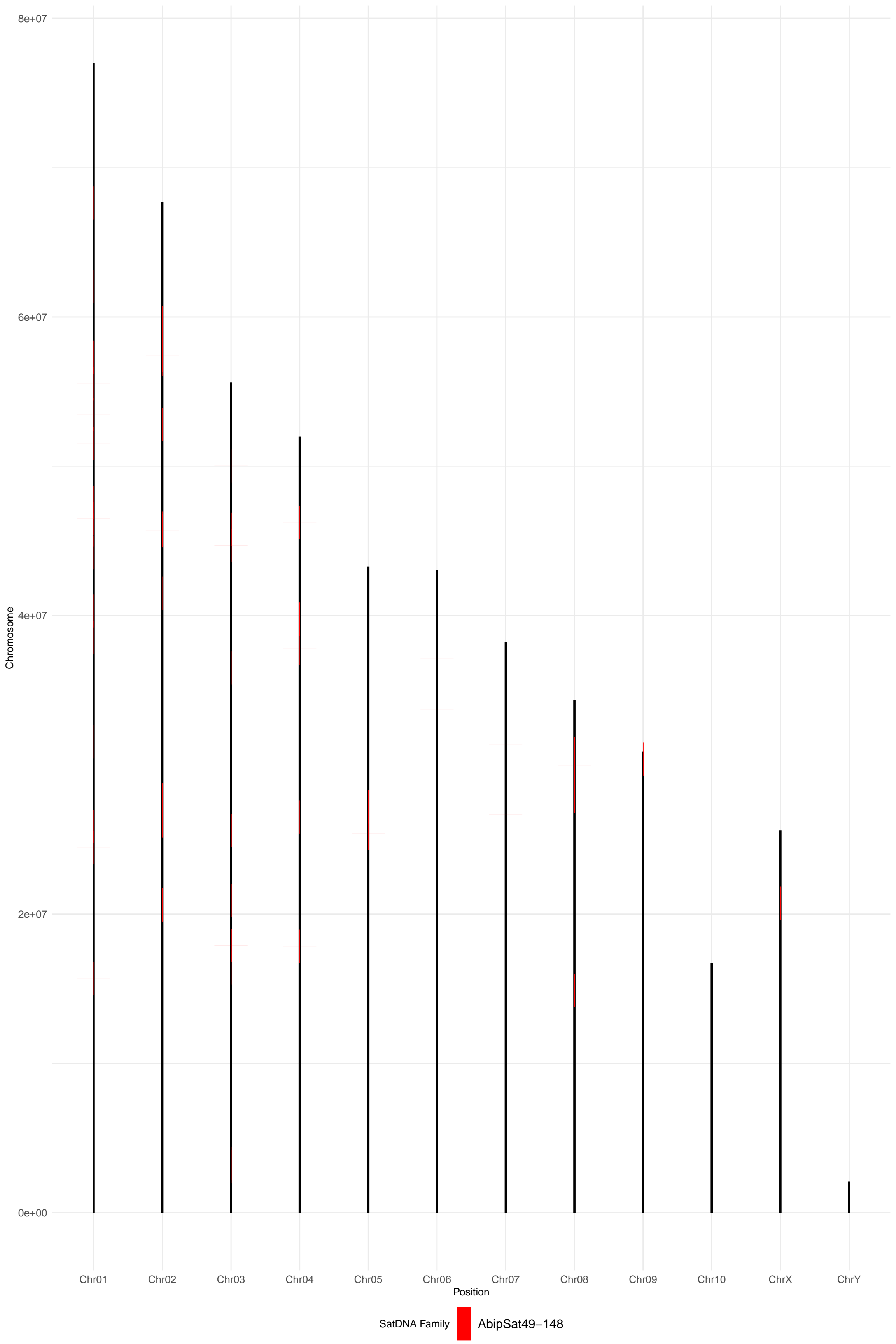

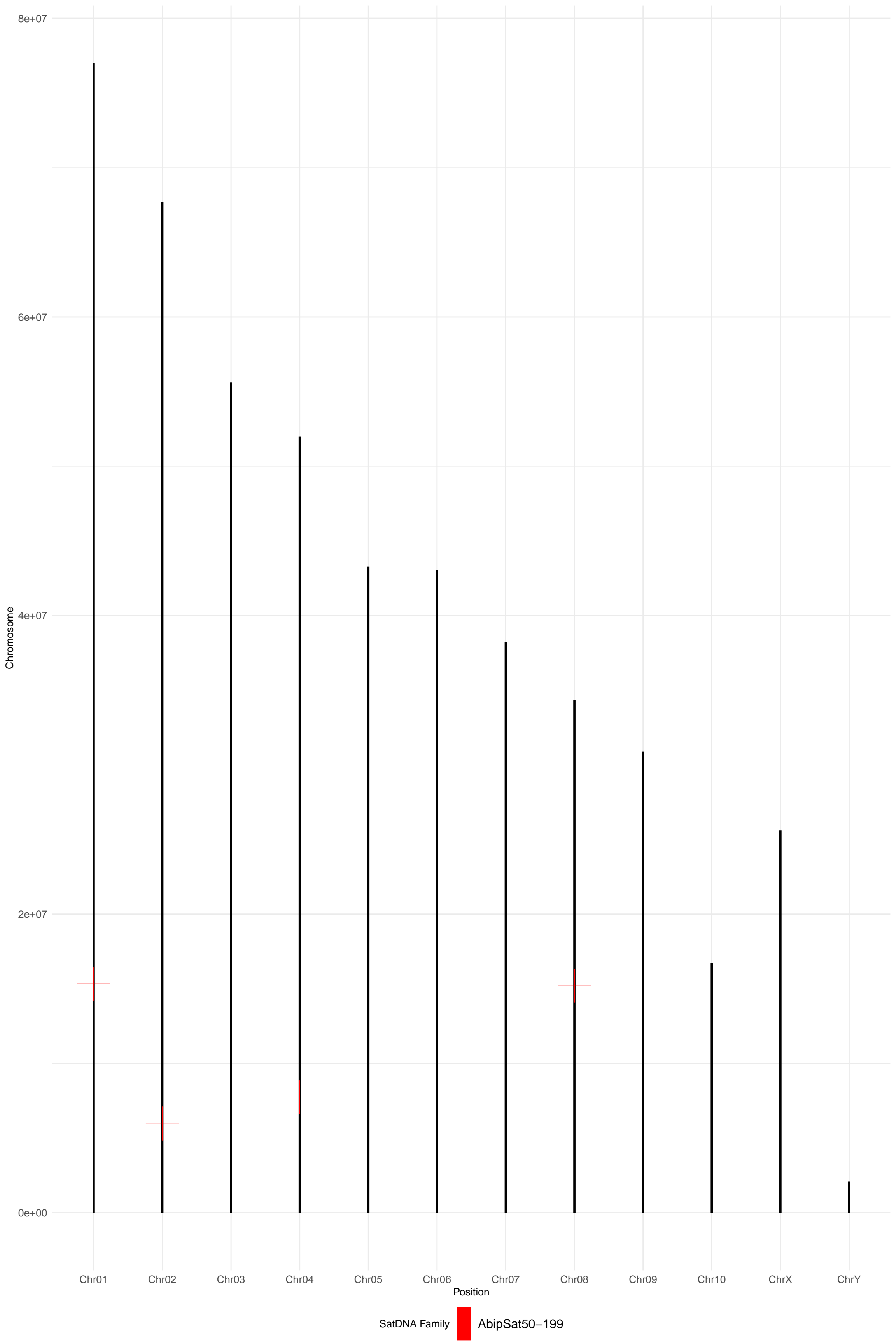

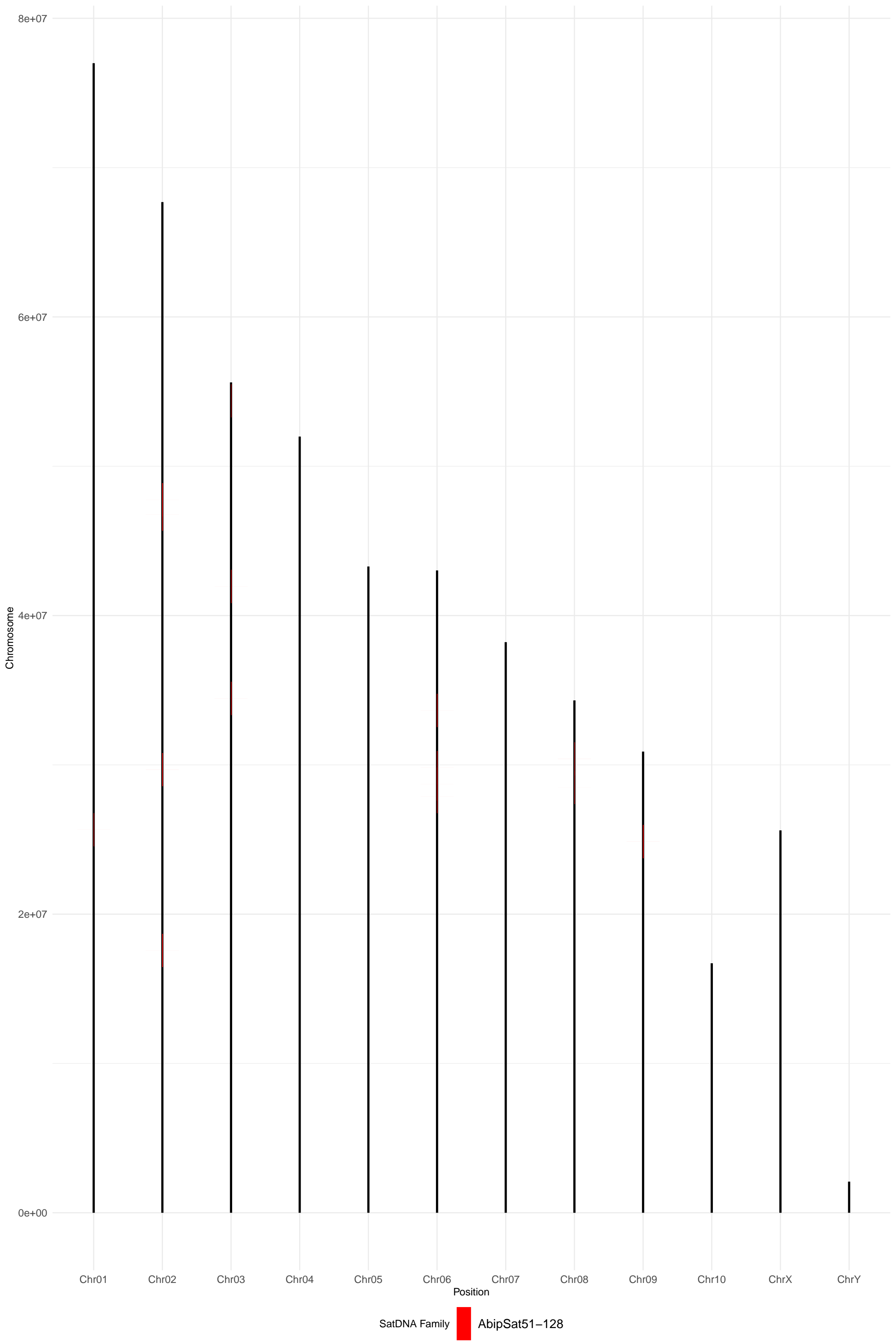

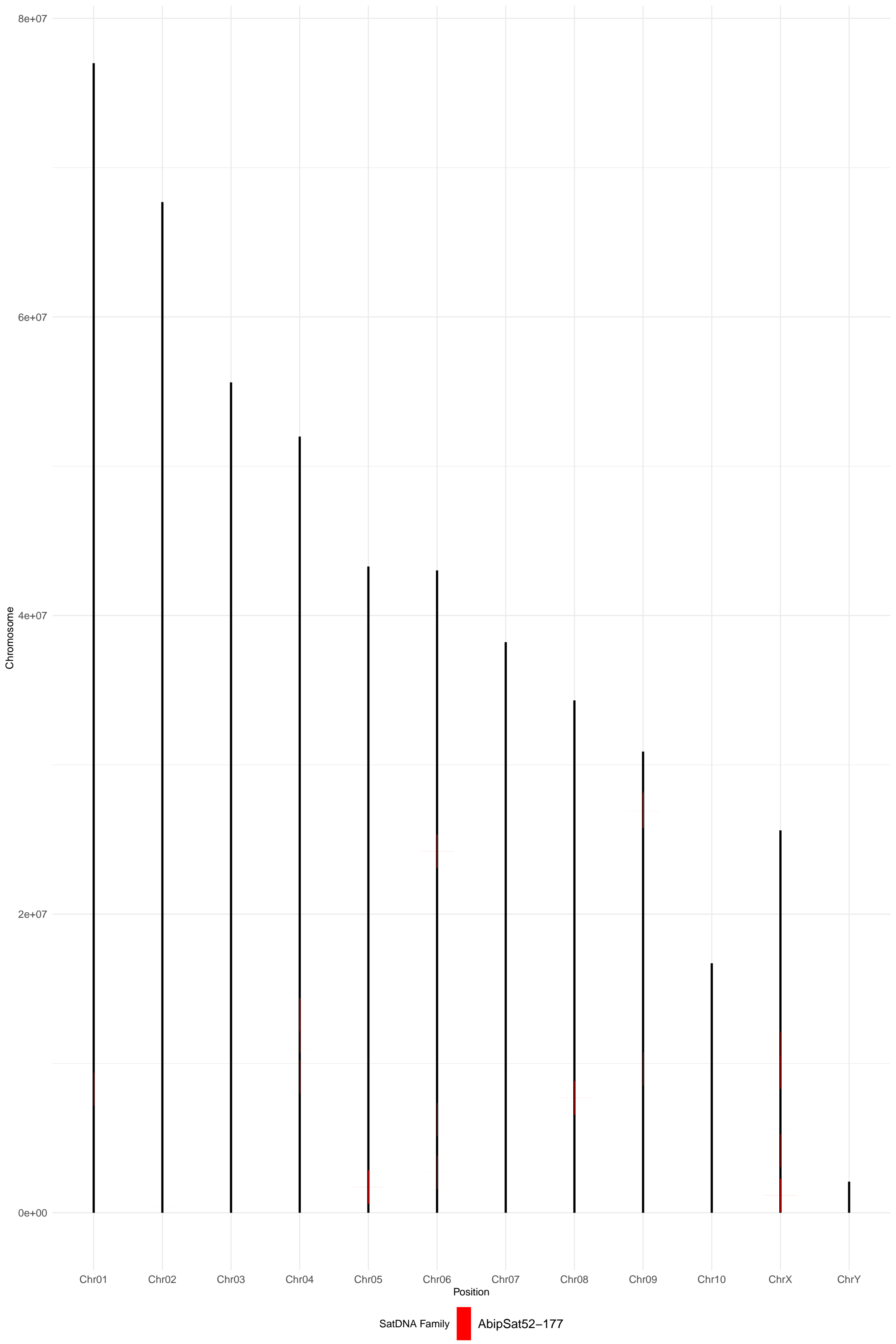

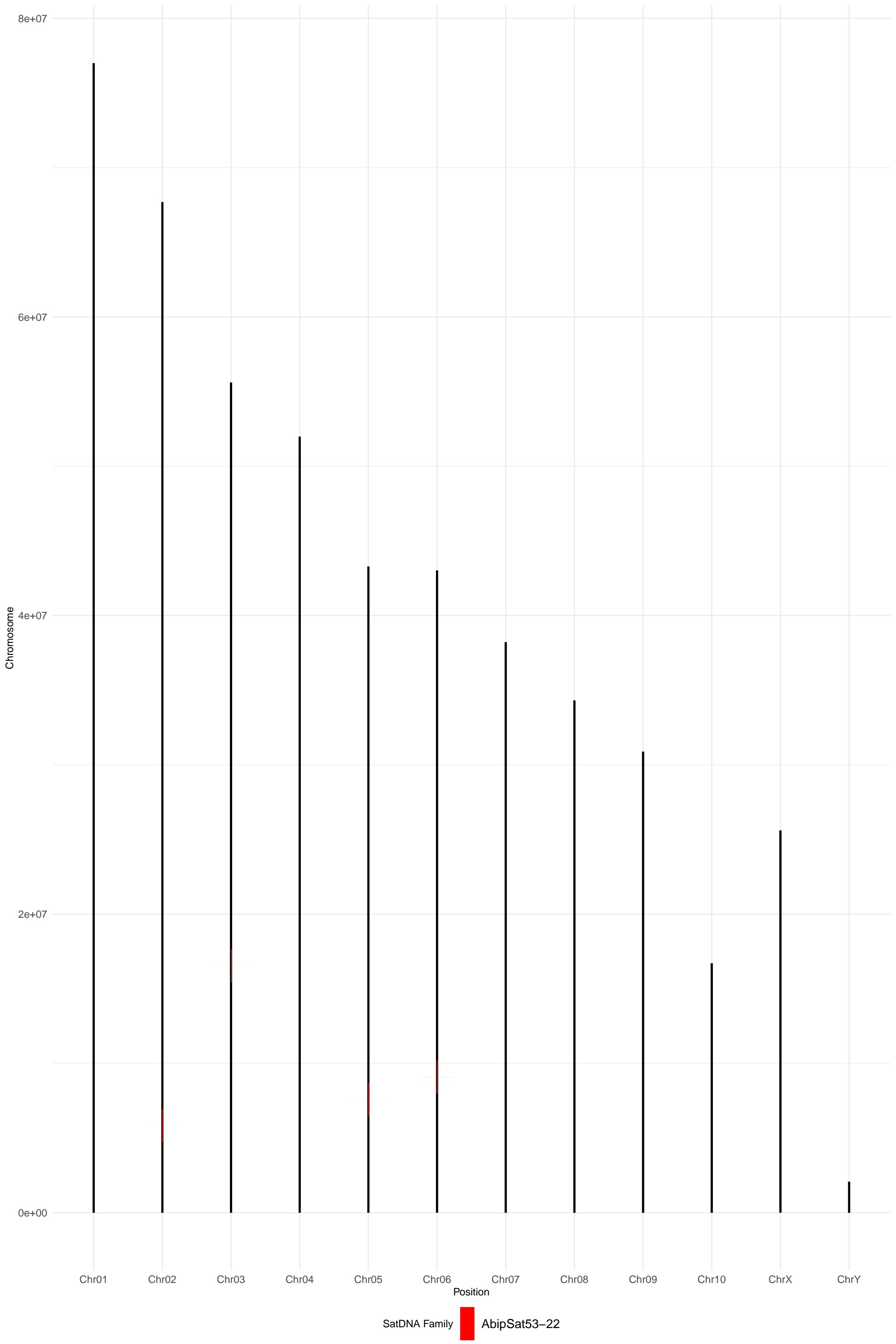

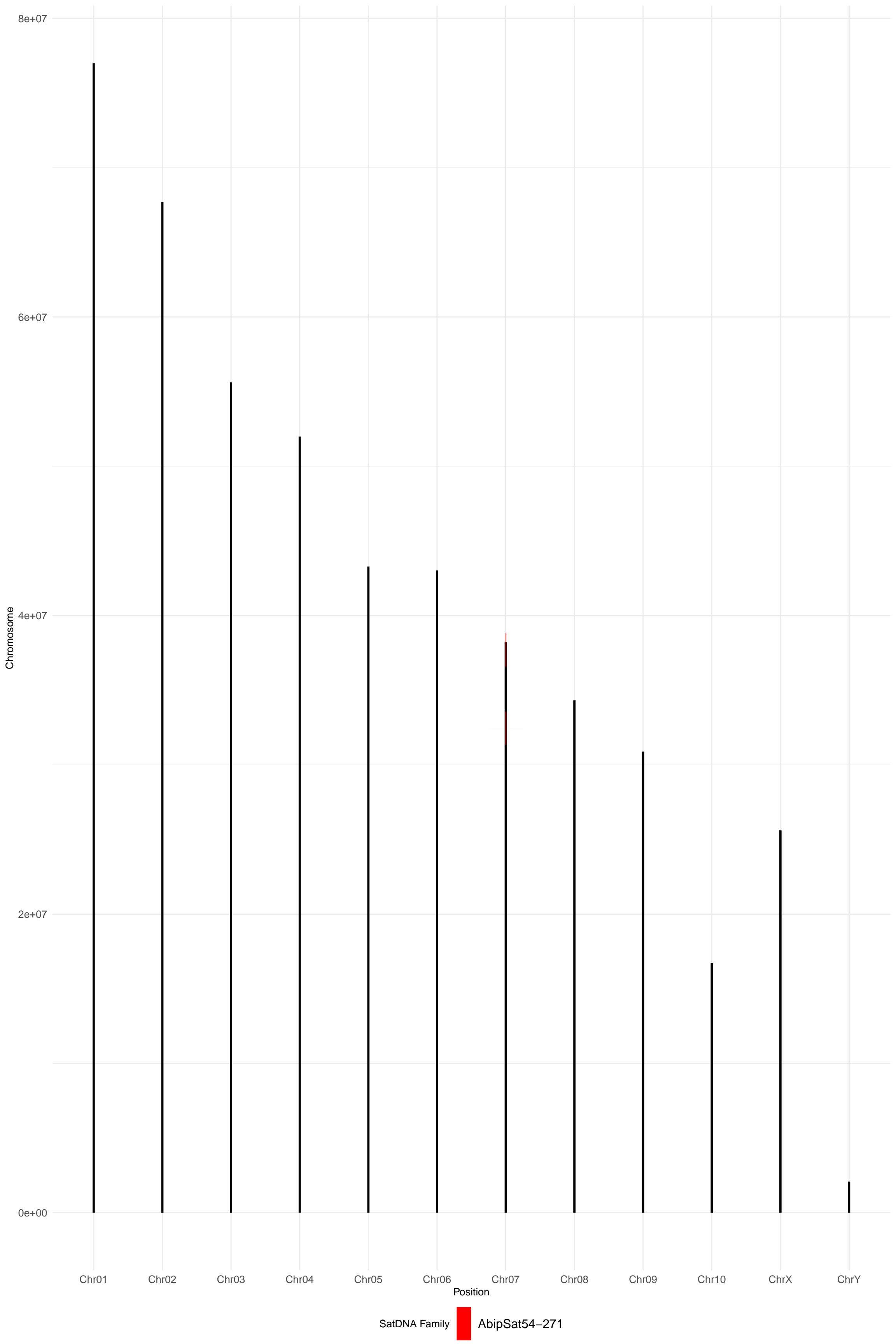

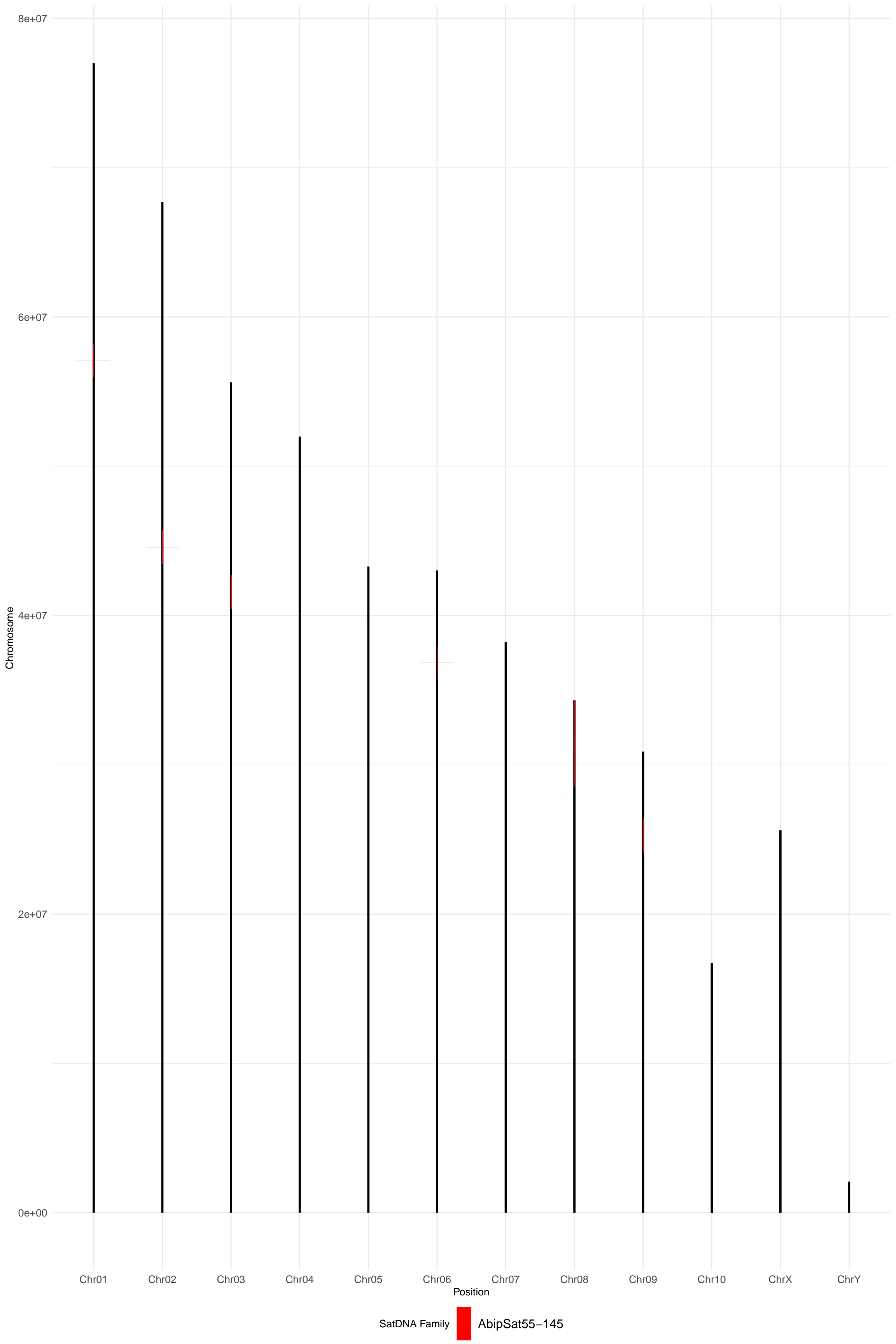

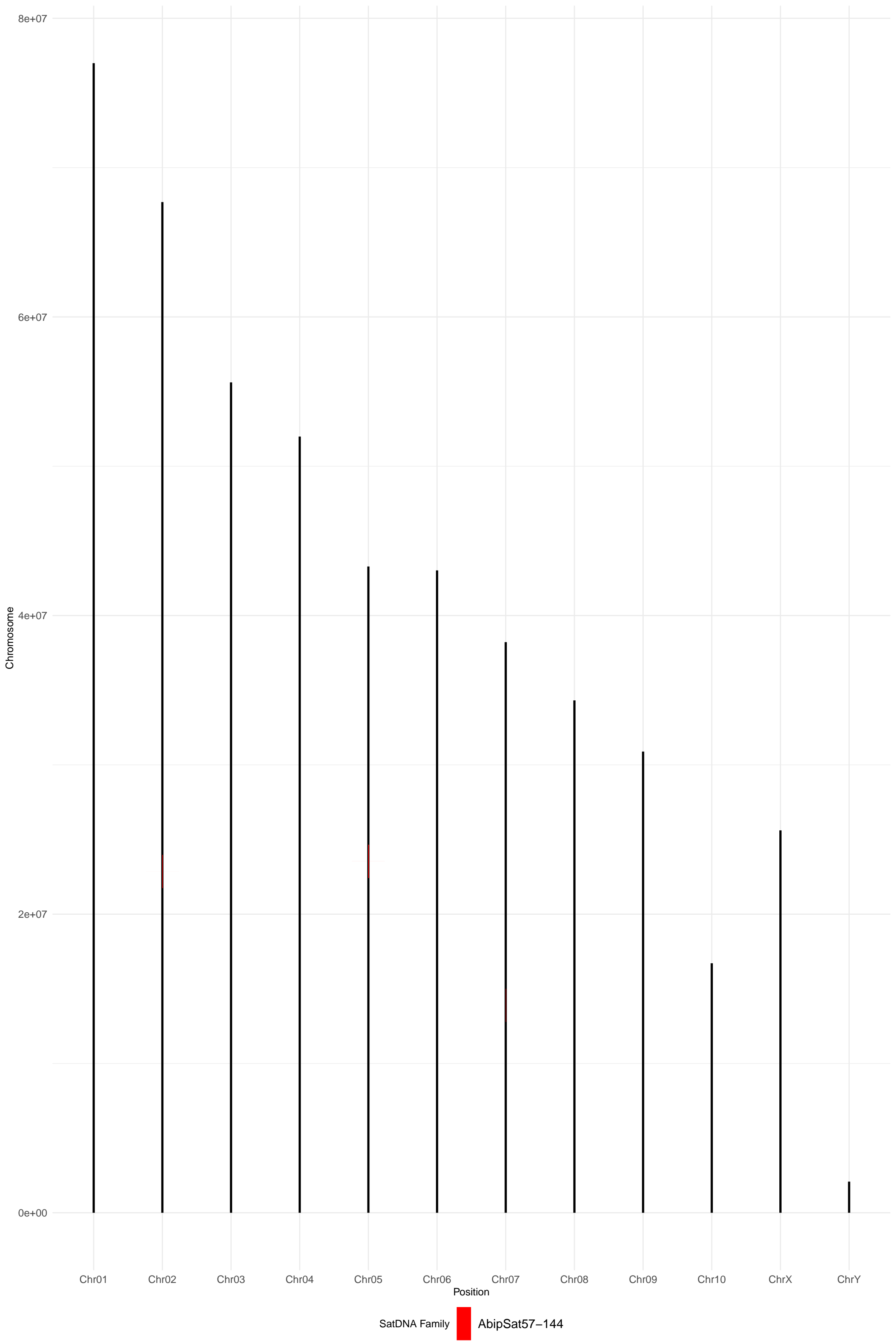

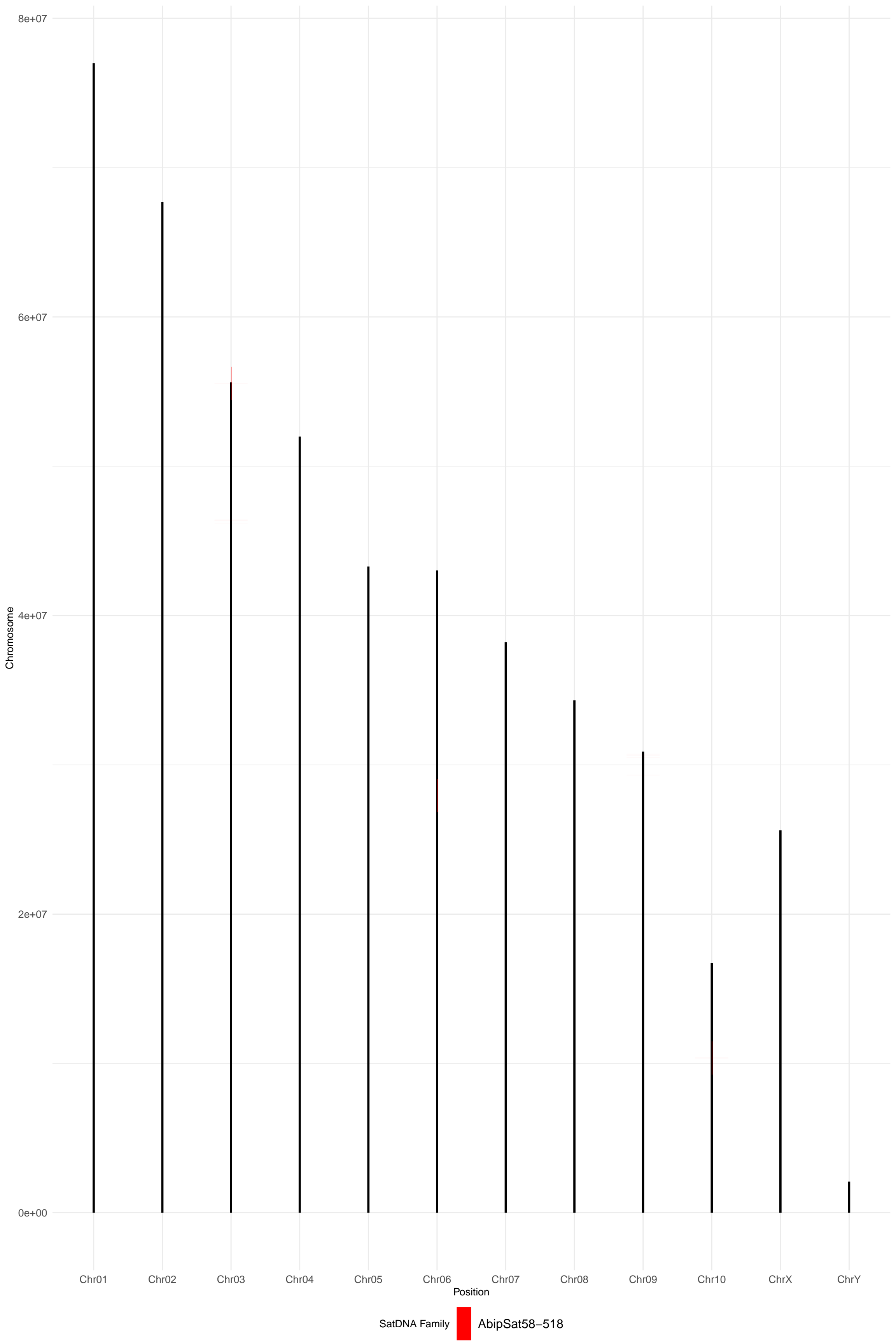

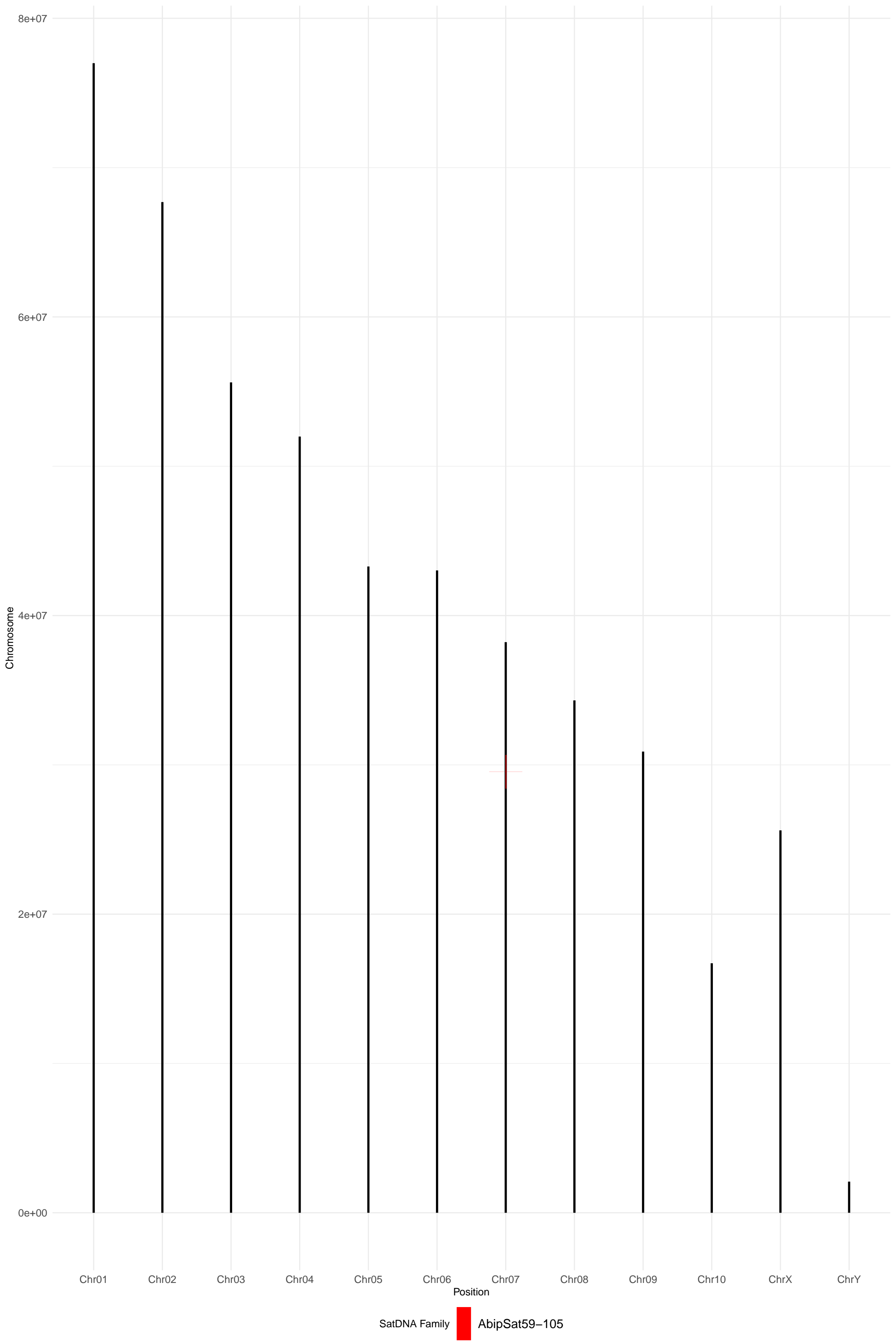

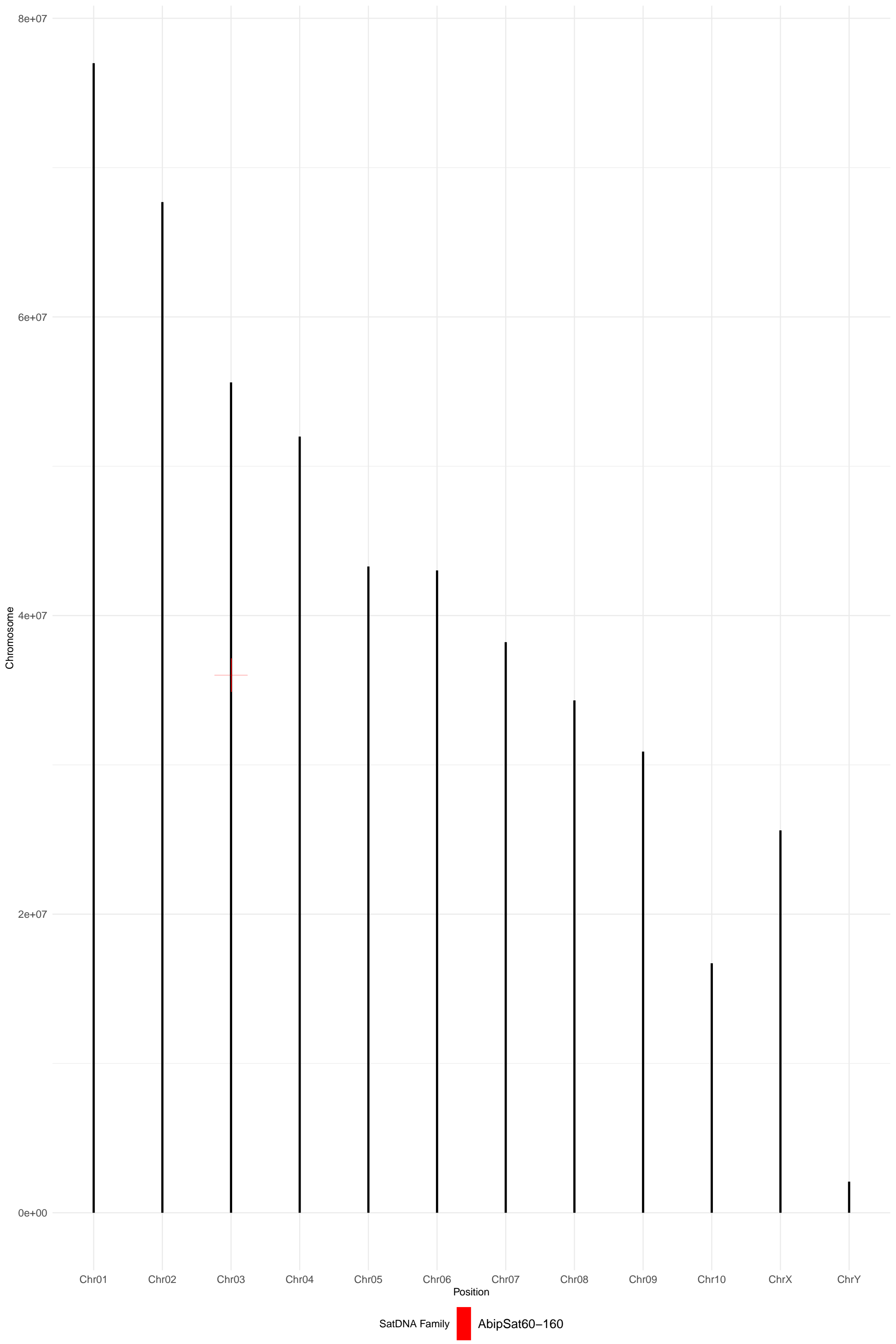

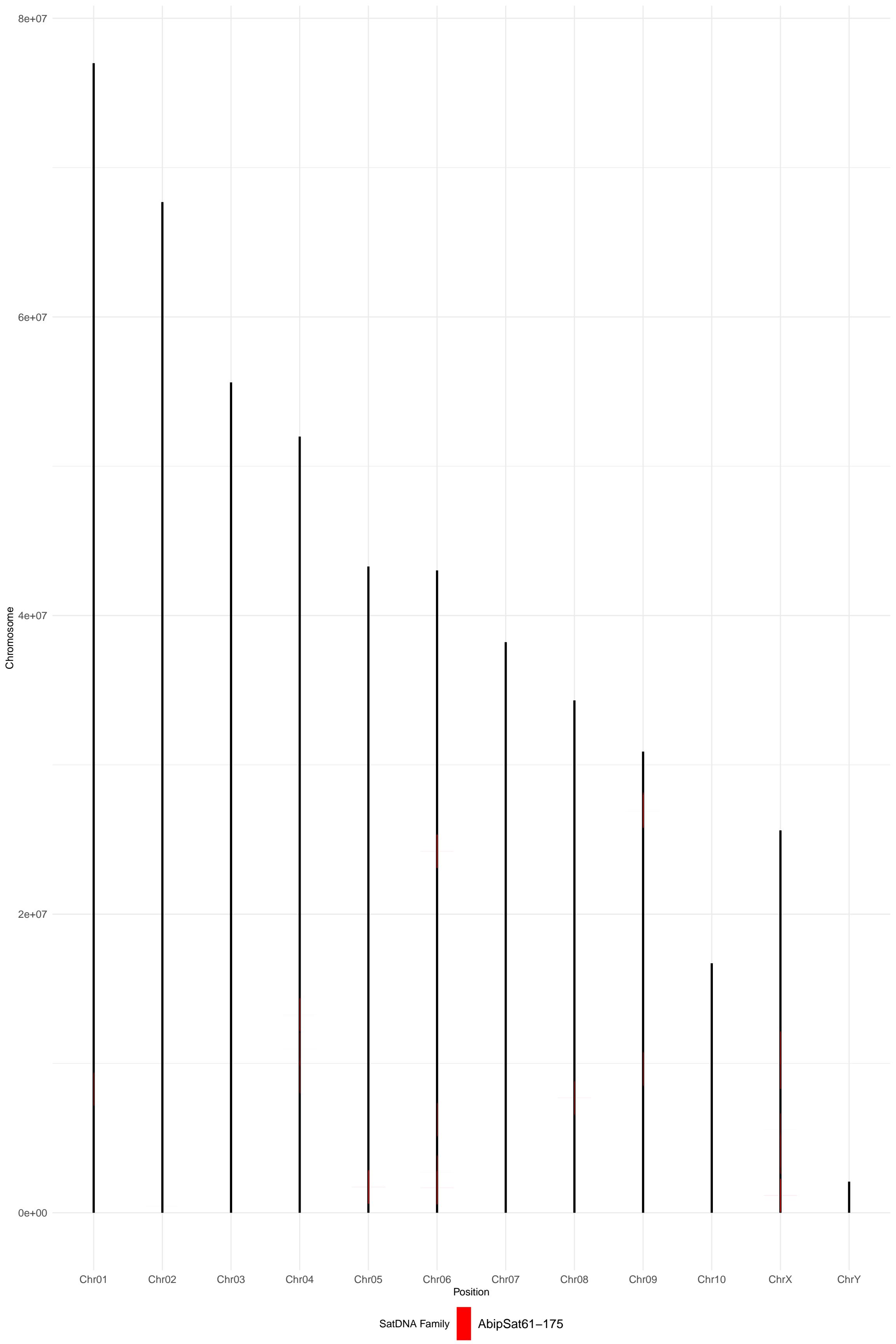

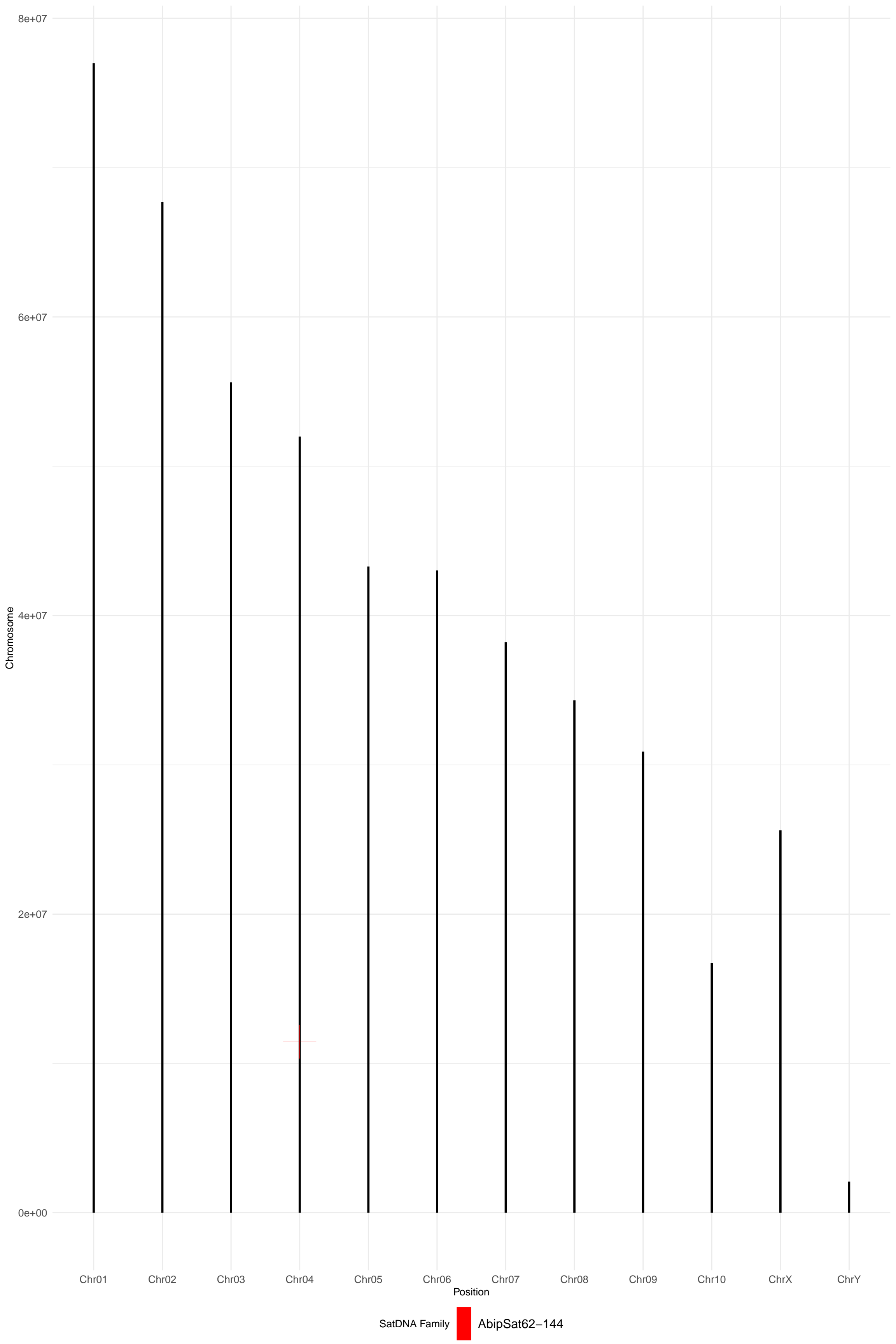

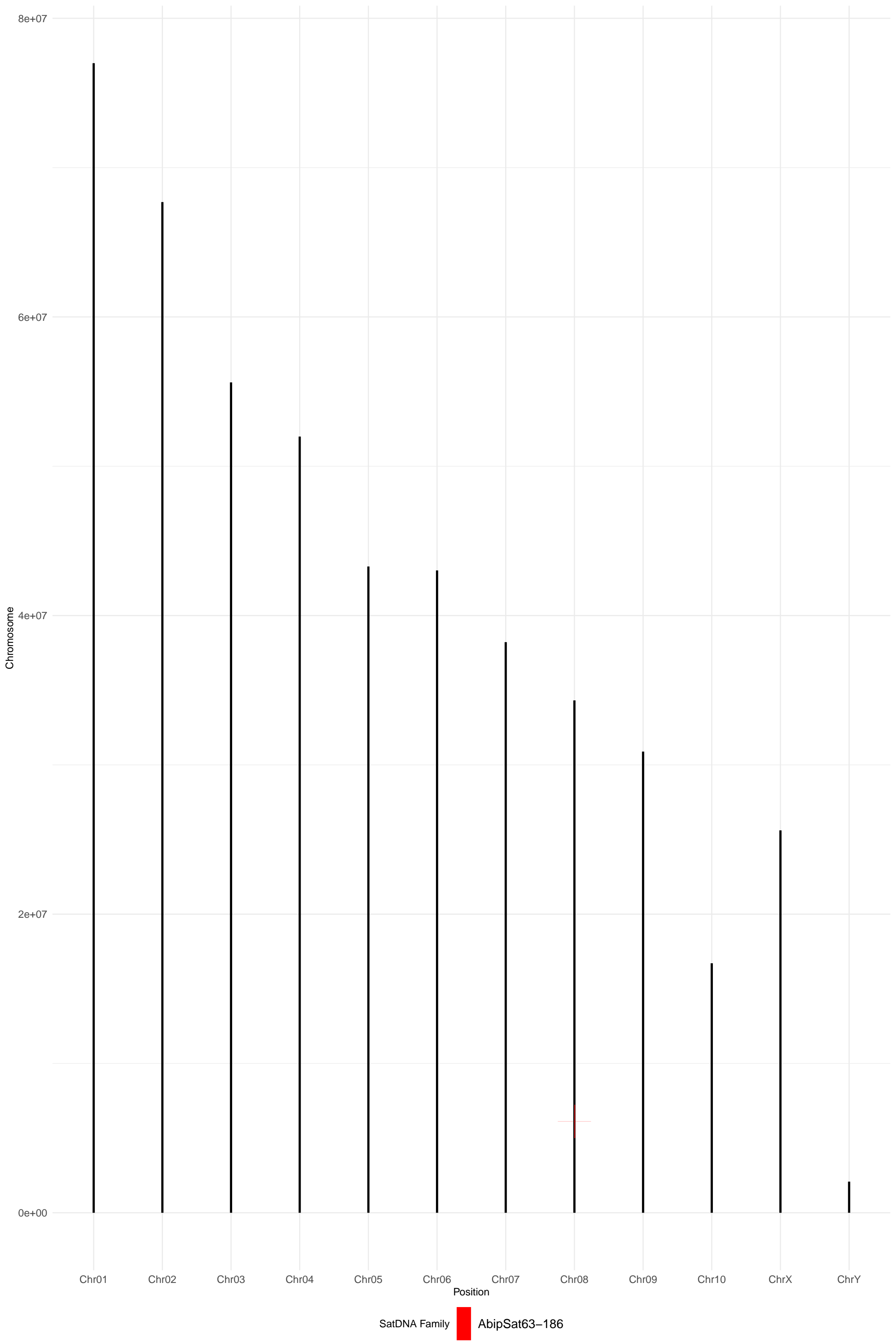

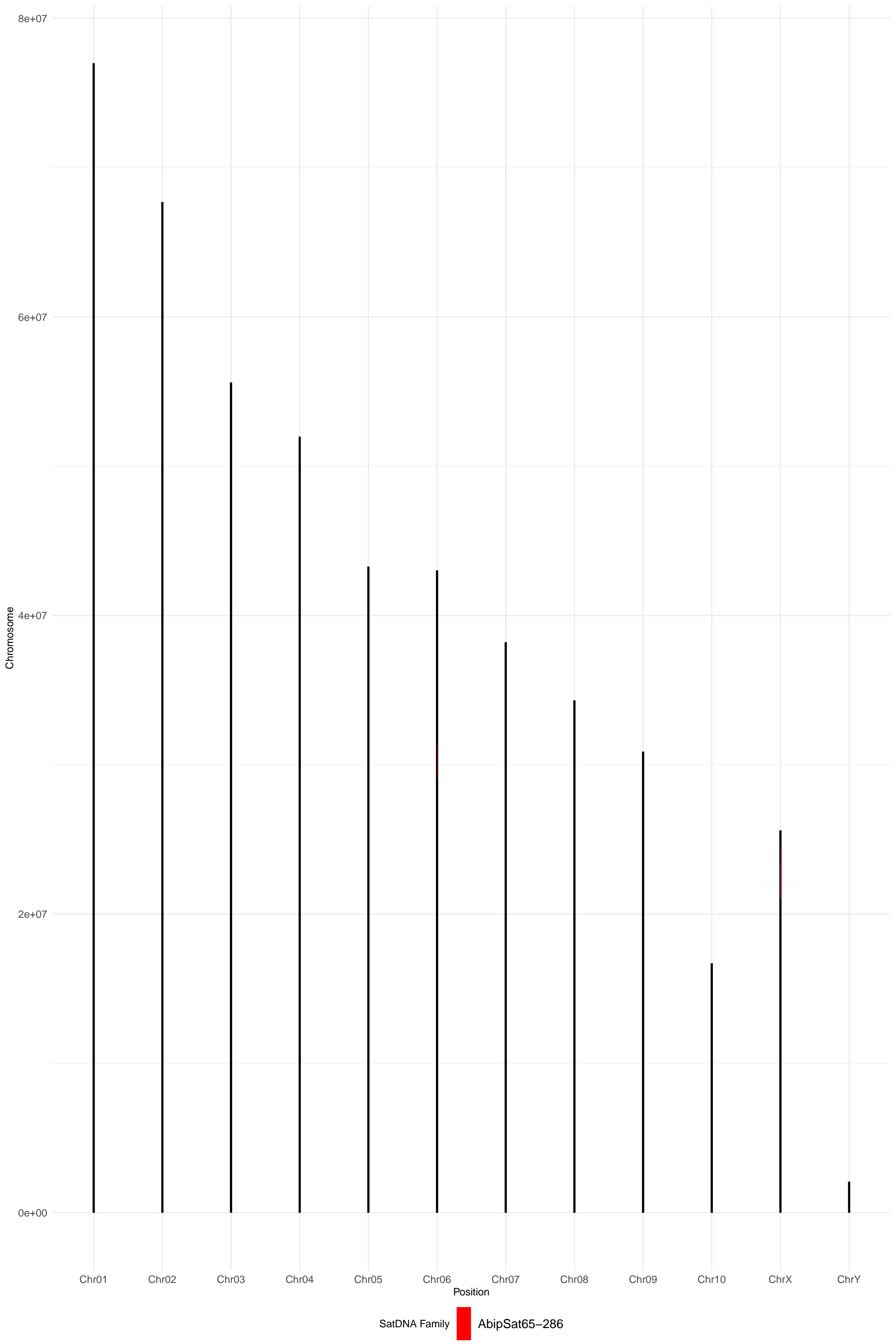

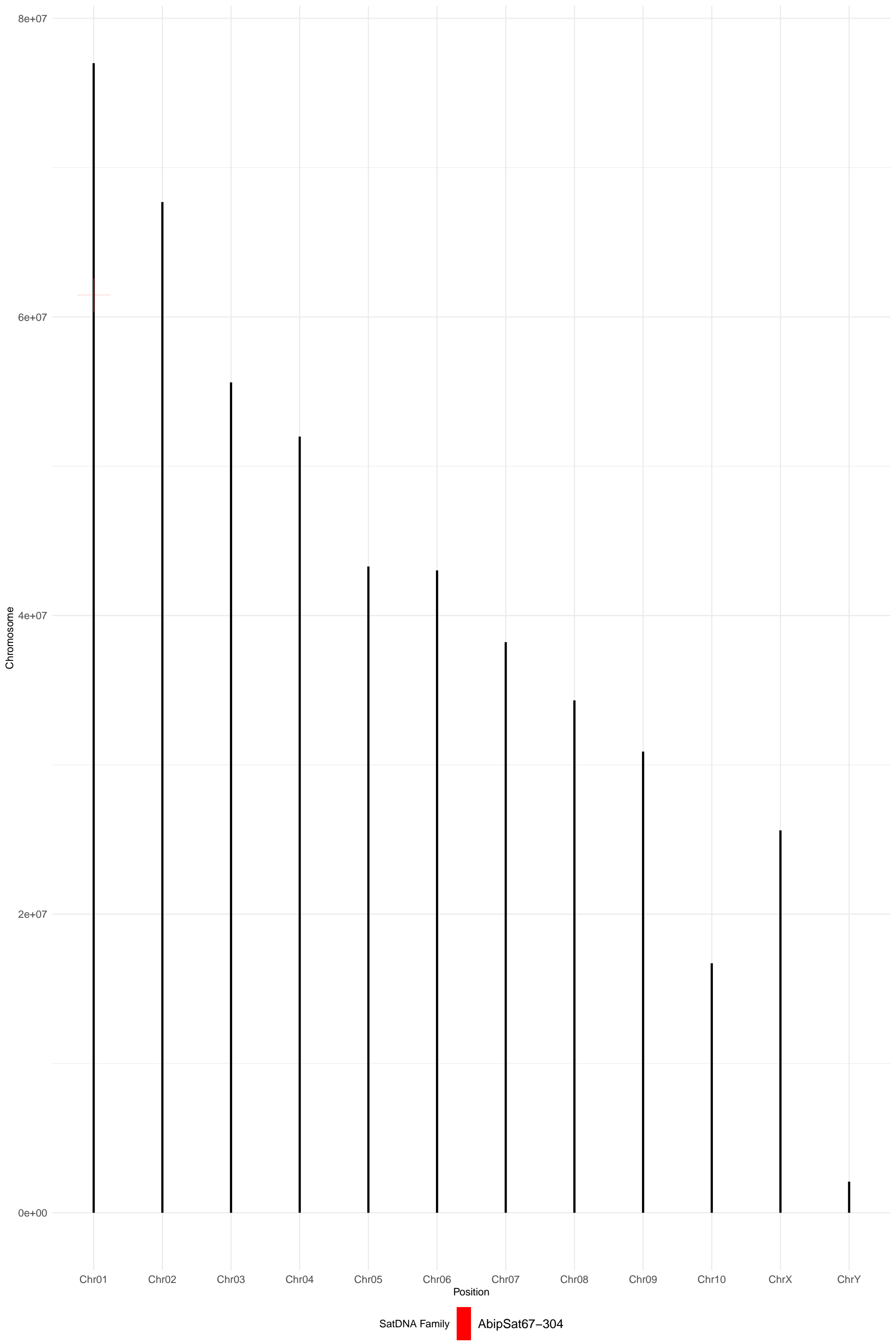

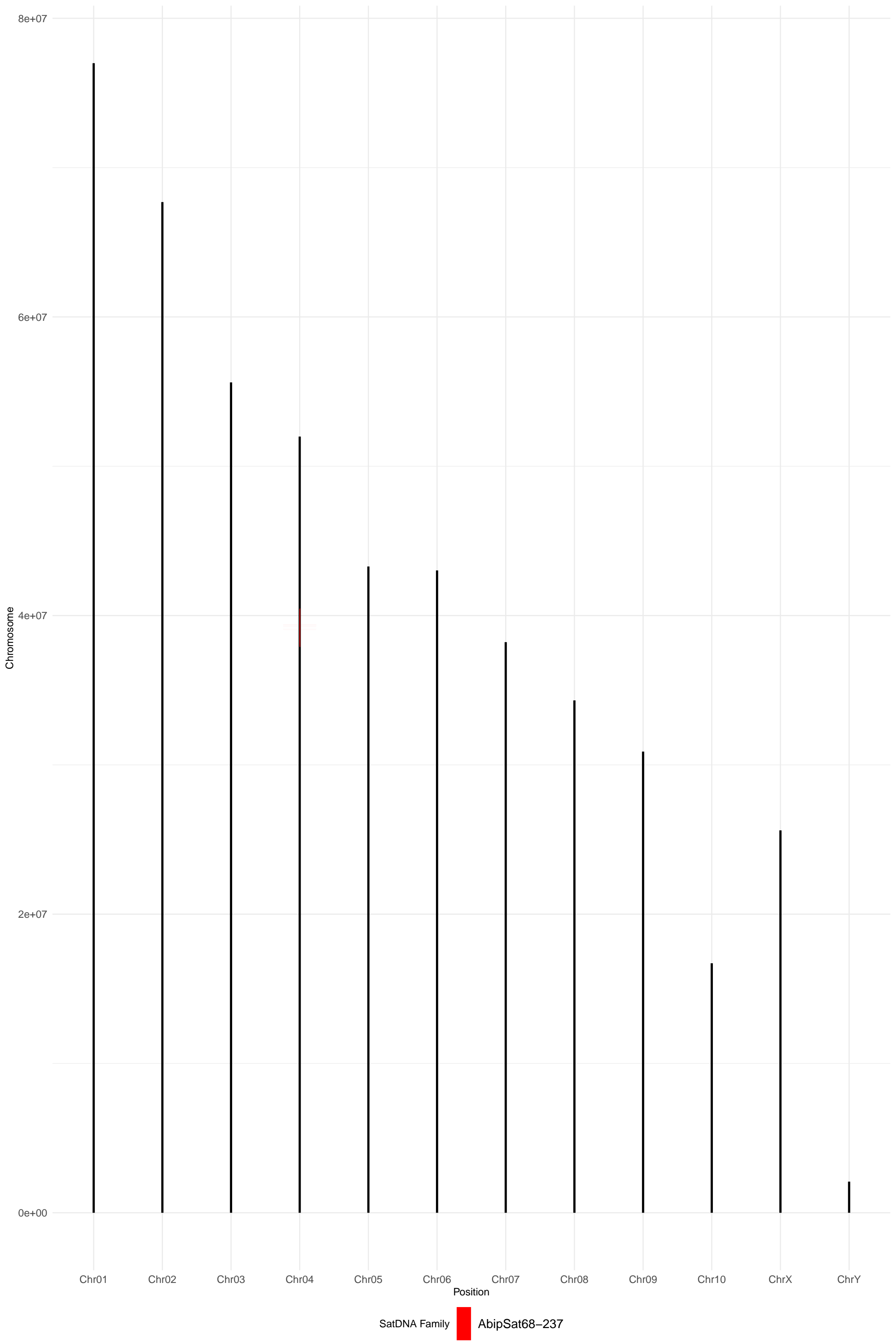

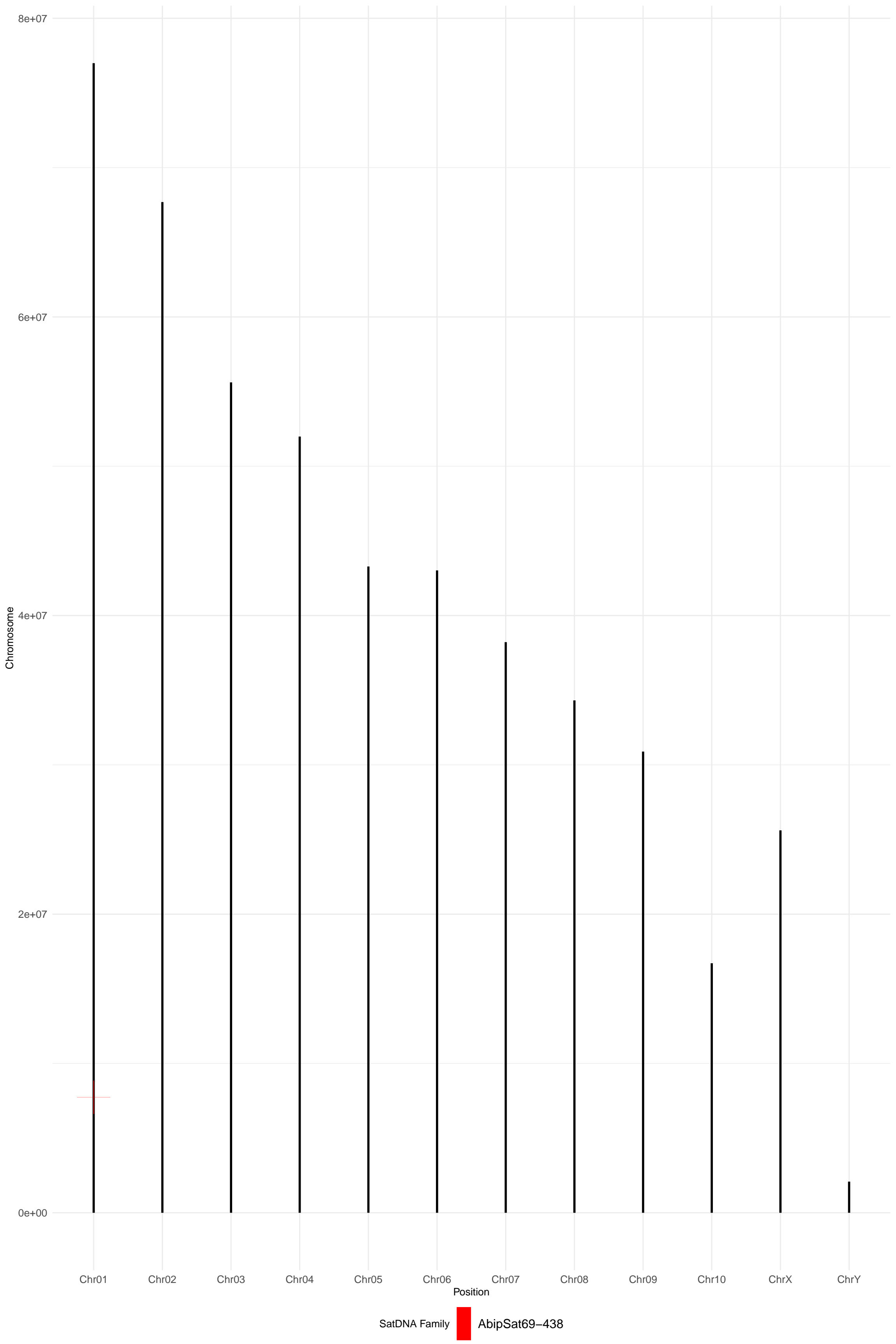

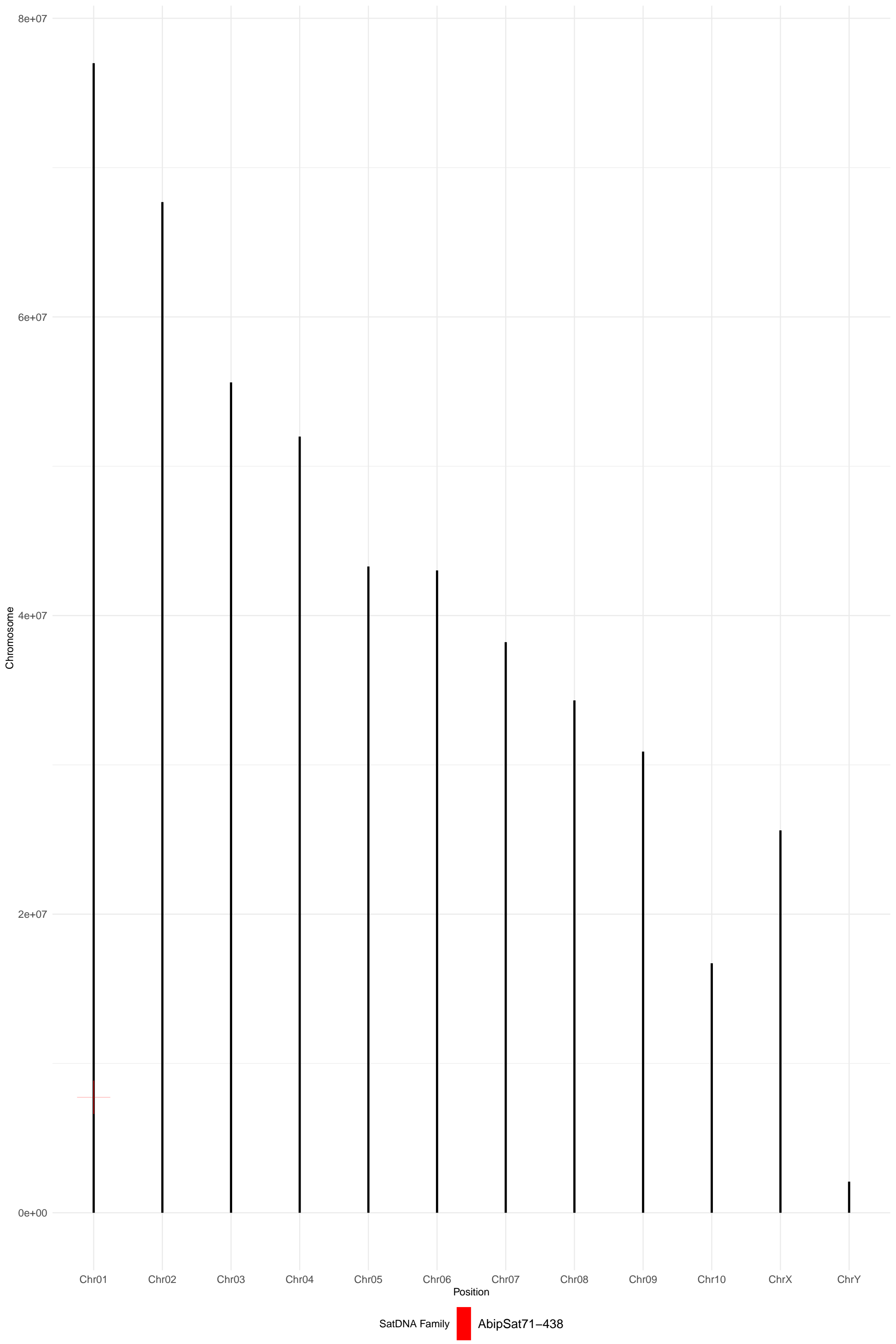

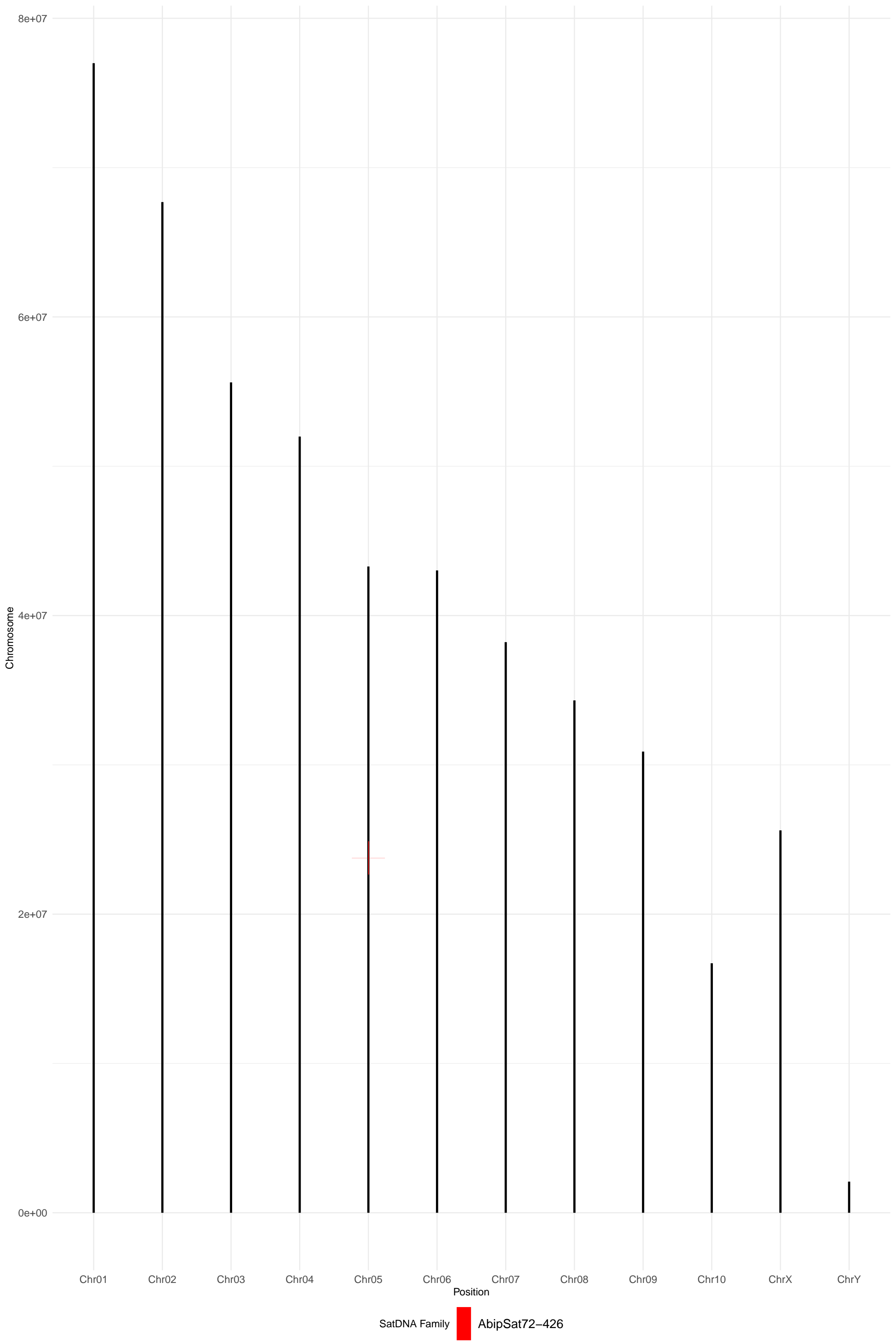

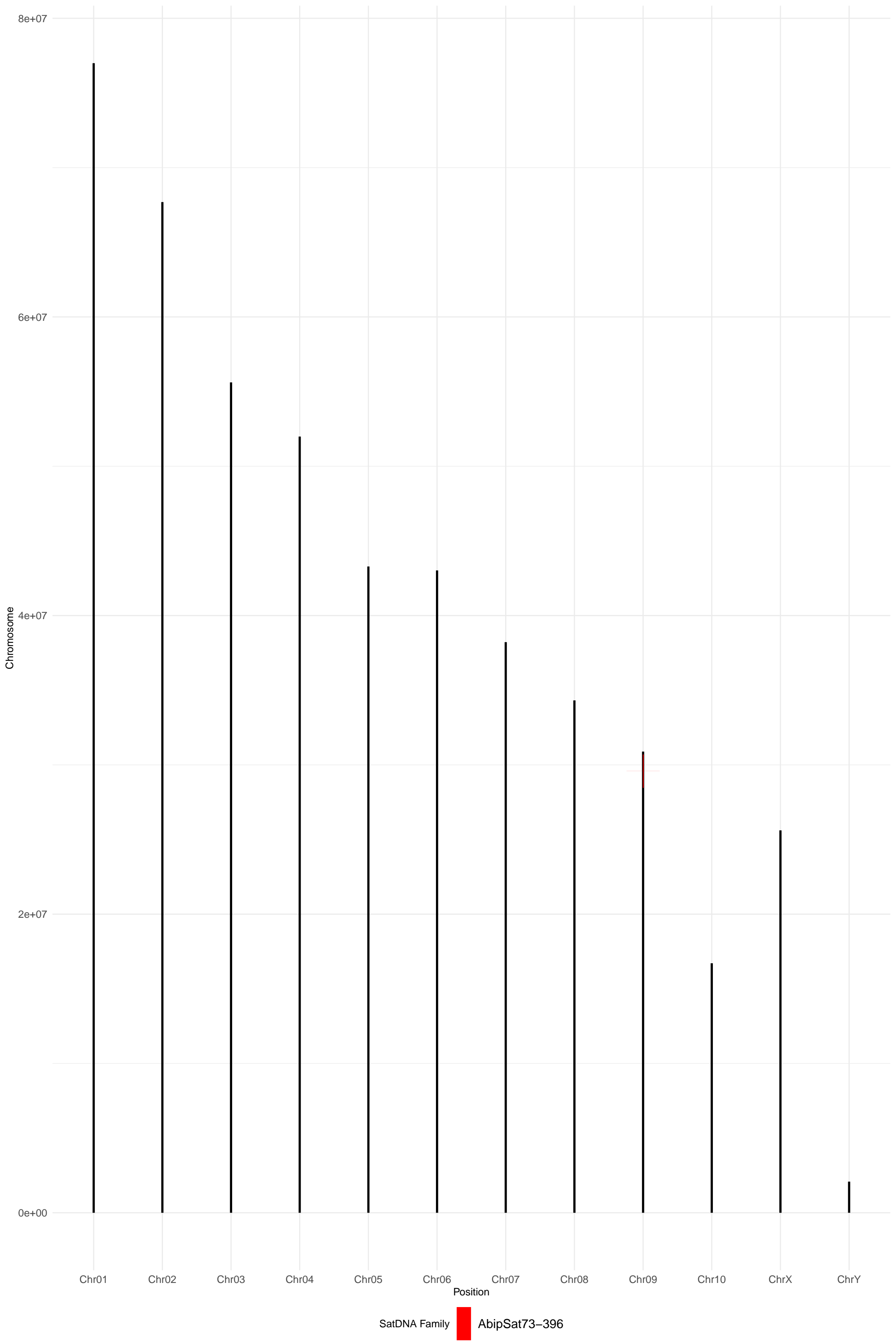

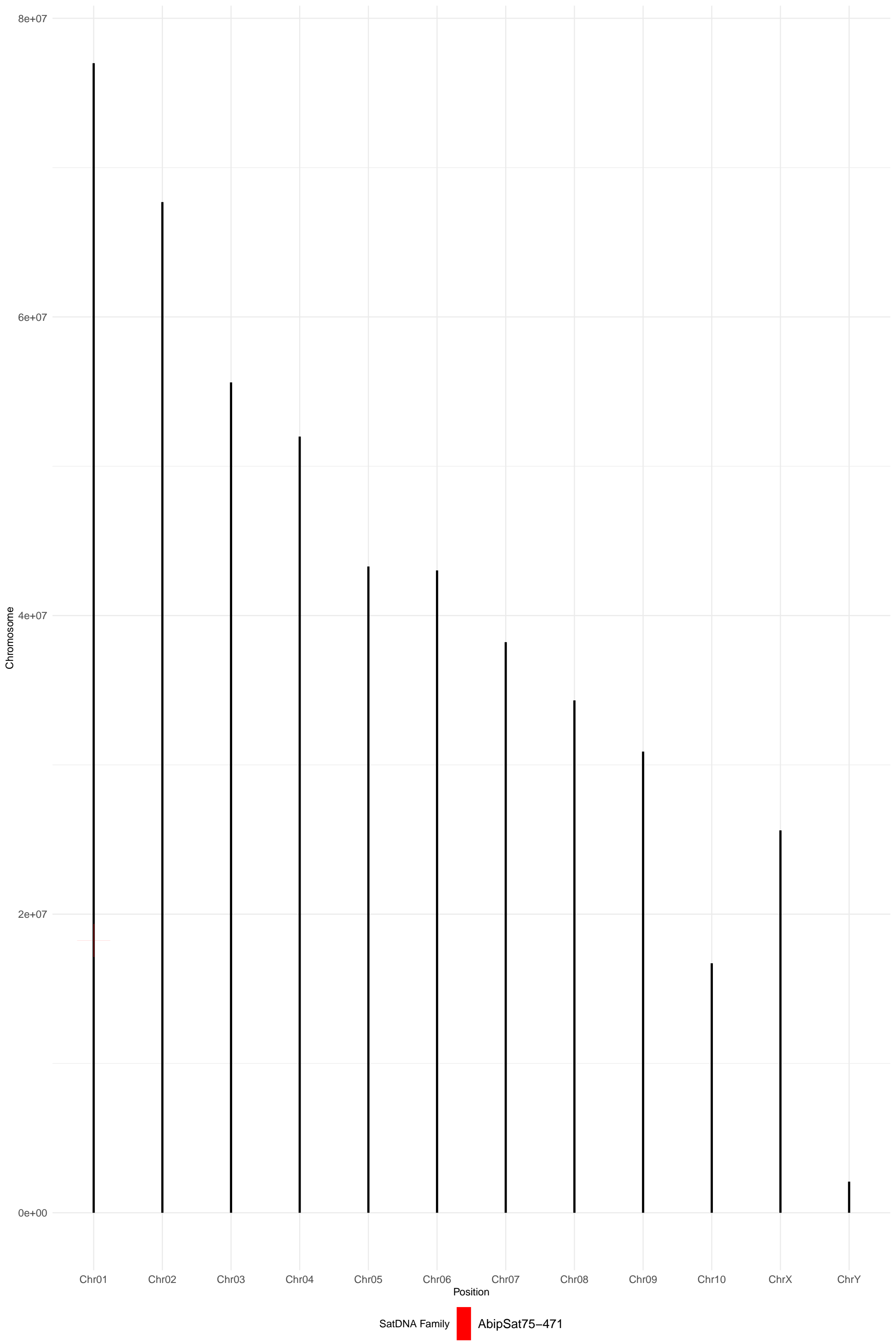

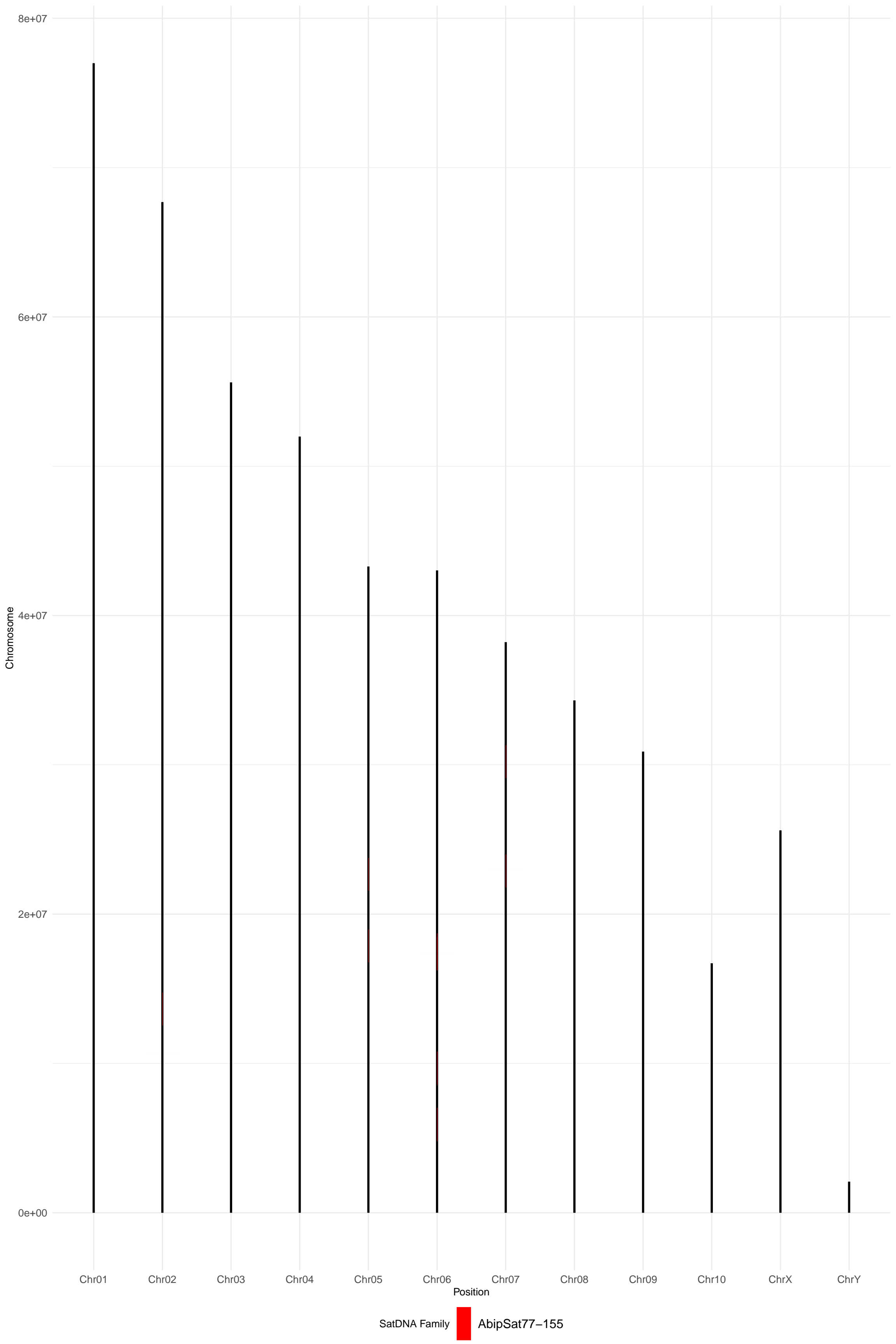

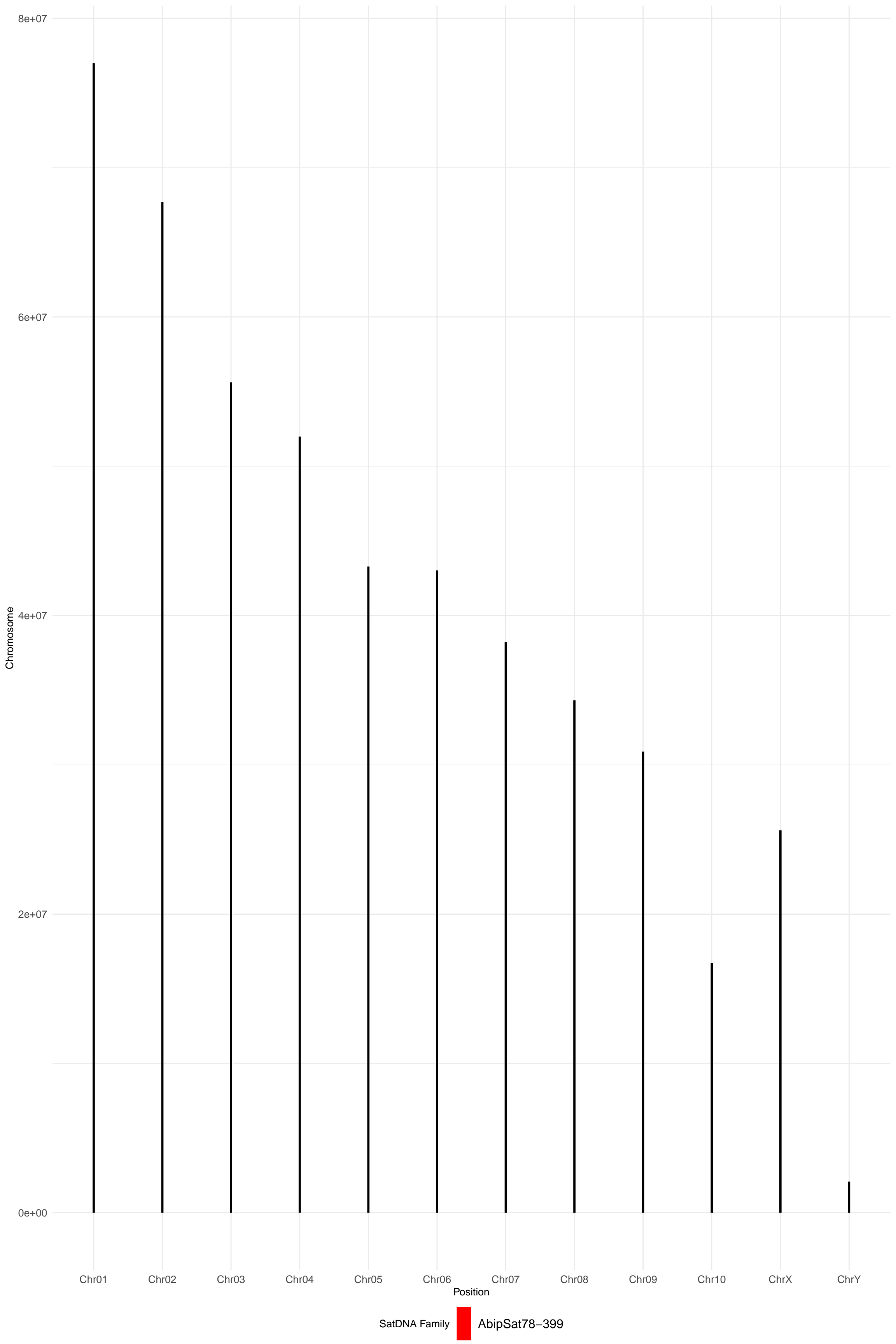

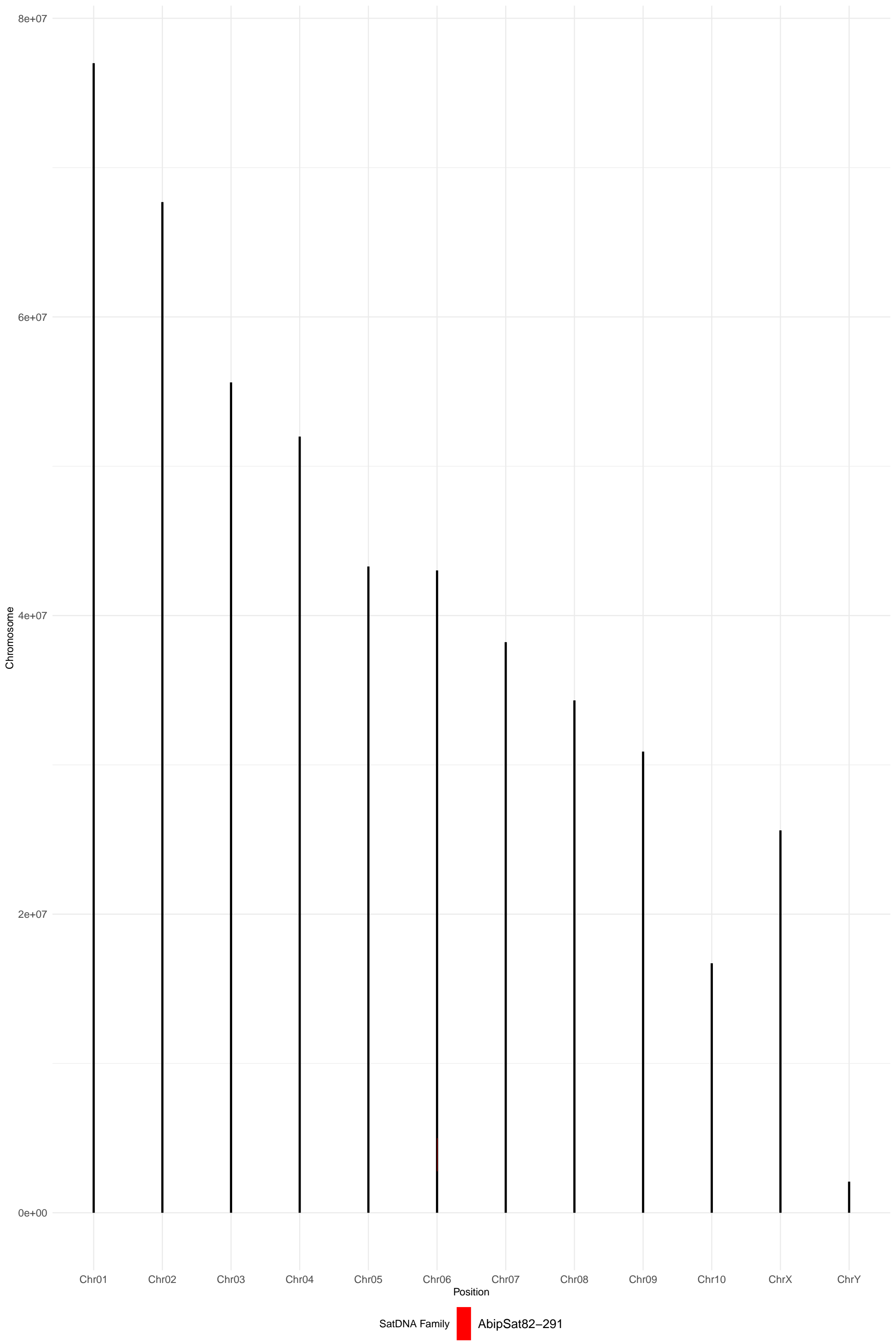

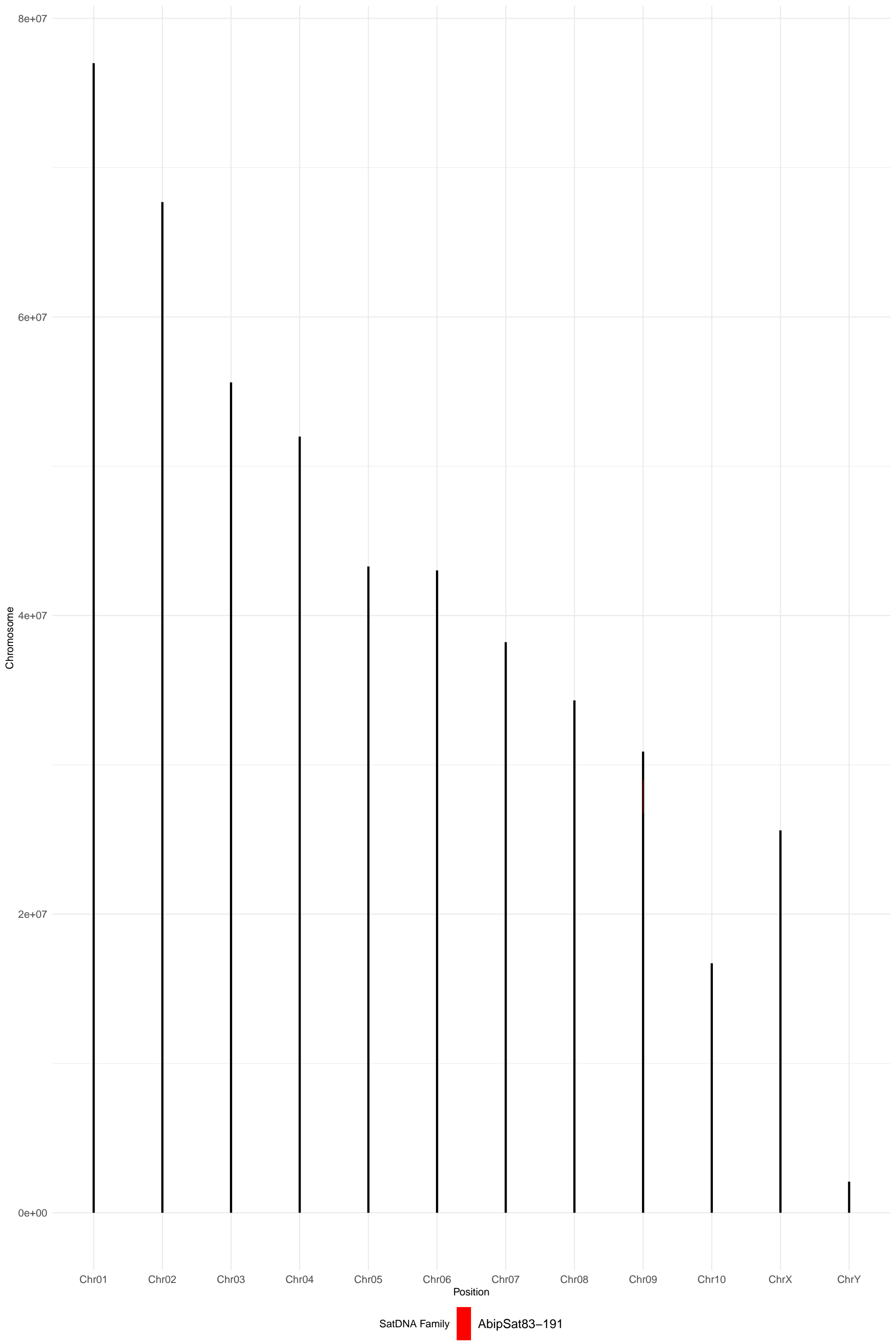

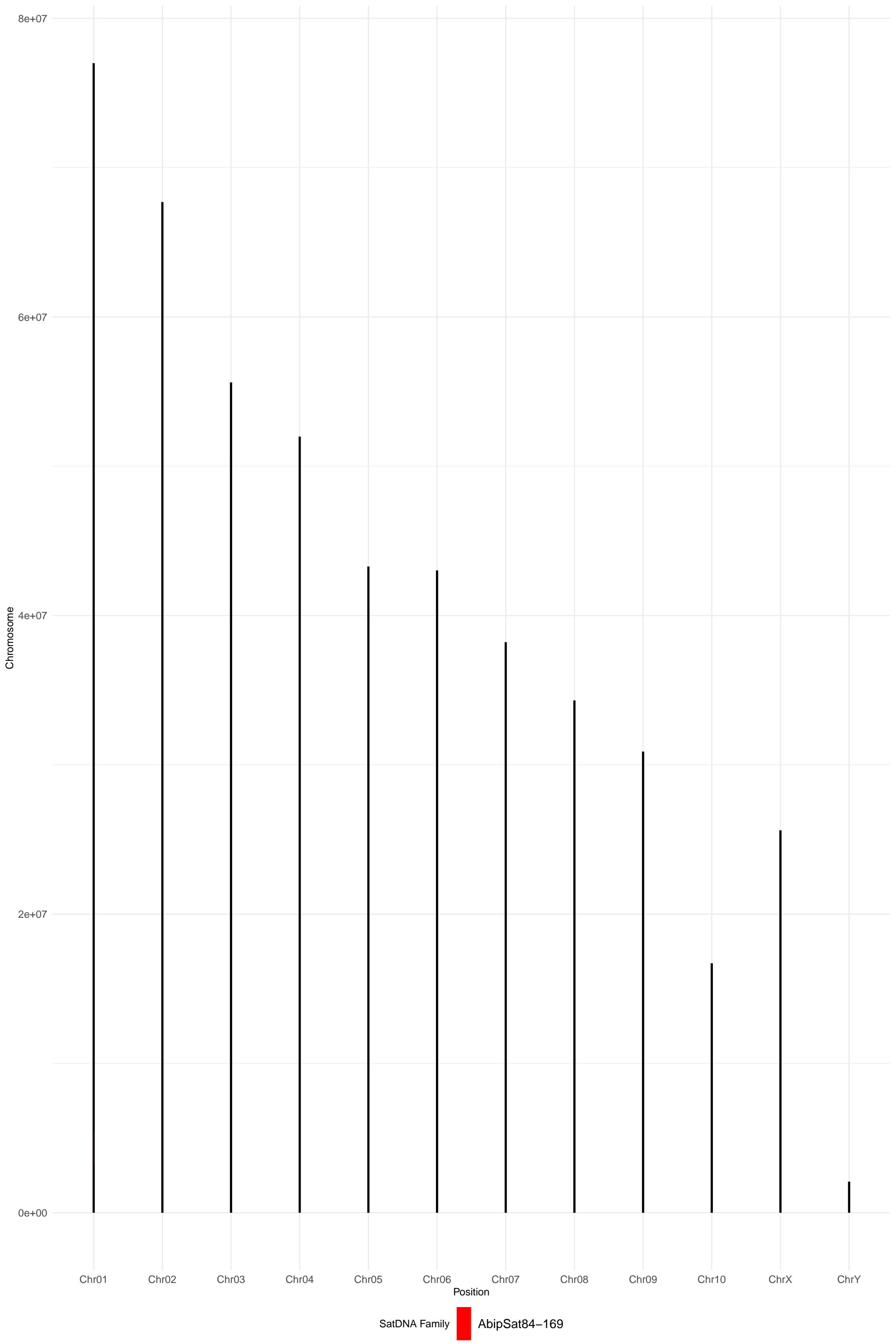

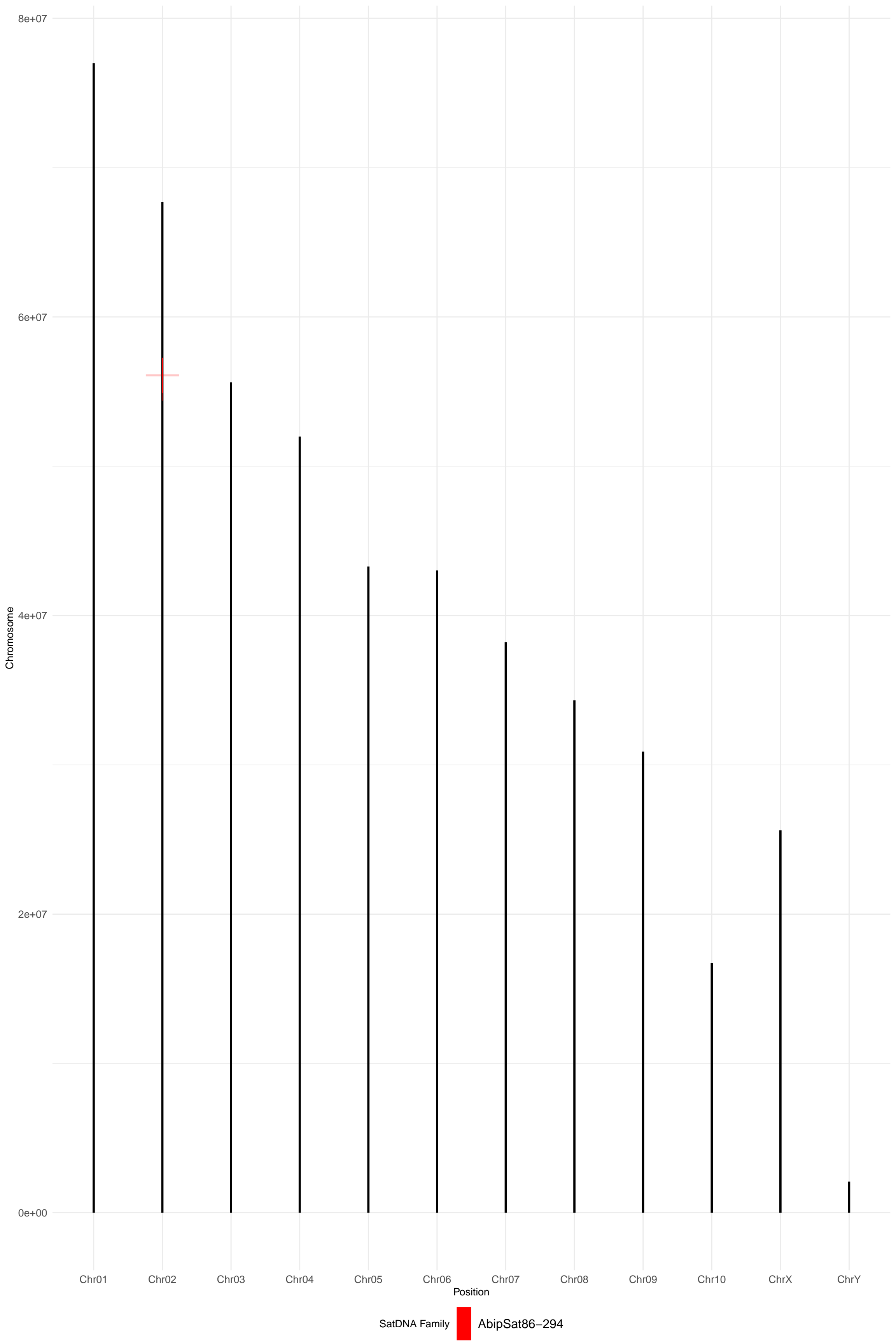

Supplement: Supplementary file 1 [file ijms-25-09214-s001.zip › Supplementary Figure S6 CHRISMAPP A_decempunctata.pdf]
